# High resolution analysis of proteolytic substrate processing

**DOI:** 10.1016/j.jbc.2024.107812

**Published:** 2024-09-21

**Authors:** Jasmin Schillinger, Michelle Koci, Kenny Bravo-Rodriguez, Geronimo Heilmann, Farnusch Kaschani, Markus Kaiser, Christine Beuck, Hartmut Luecke, Robert Huber, Doris Hellerschmied, Steven G. Burston, Michael Ehrmann

**Affiliations:** 1 Center of Medical Biotechnology, Faculty of Biology, University Duisburg-Essen, Essen, Germany; 2 Max-Planck-Institute of Molecular Physiology, Dortmund, Germany; 3 Nova School of Science and Technology, Lisbon, Portugal; 4 Department of Biophysics, University of California, Irvine, California, USA; 5 Max-Planck-Institute for Biochemistry, Martinsried, Germany; 6 School of Biochemistry, University of Bristol, Biomedical Sciences Building, Bristol, United Kingdom

**Keywords:** HTRA1, proteolysis, protein processing, protein degradation, bioinformatics, ANXA1, trypsin

## Abstract

Members of the widely conserved high temperature requirement A (HtrA) family of serine proteases are involved in multiple aspects of protein quality control. In this context, they have been shown to efficiently degrade misfolded proteins or protein fragments. However, recent reports suggest that folded proteins can also be native substrates. To gain a deeper understanding of how folded proteins are initially processed and subsequently degraded into short peptides by human HTRA1, we established an integrated and quantitative approach using time-resolved mass spectrometry, CD spectroscopy, and bioinformatics. The resulting data provide high-resolution information on up to 178 individual proteolytic sites within folded ANXA1 (consisting of 346 amino acids), the relative frequency of cuts at each proteolytic site, the preferences of the protease for the amino acid sequence surrounding the scissile bond, as well as the degrees of sequential structural relaxation and unfolding of the substrate that occur during progressive degradation. Our workflow provides precise molecular insights into protease-substrate interactions, which could be readily adapted to address other posttranslational modifications such as phosphorylation in dynamic protein complexes.

Members of the highly conserved high temperature requirement A (HtrA) family of serine proteases are implicated in protein quality control and cellular stress signaling. Deregulation of human HTRA1 is associated with severe pathologies such as age-related macular degeneration, Alzheimer’s disease, cancer, arthritis, and familial ischemic cerebral small vessel disease (1, 2, 3). HTRA1 is a homotrimer of approximately 147 kDa, in which each protomer consists of an N terminus corresponding to a partial insulin-like growth factor binding protein-7 domain of unknown function, an S1 serine protease domain and a C-terminal PDZ domain. High-resolution structures of each of these domains have been solved independently (4, 5, 6) and a low-resolution model of the entire protein has been proposed (7). The substrate binding site of HTRA1 is located in a groove (5). This architecture accommodates individual peptide chains in the active site, resulting in limited sequence but rather conformational selectivity (Fig. 1*A*).

**Figure 1:**
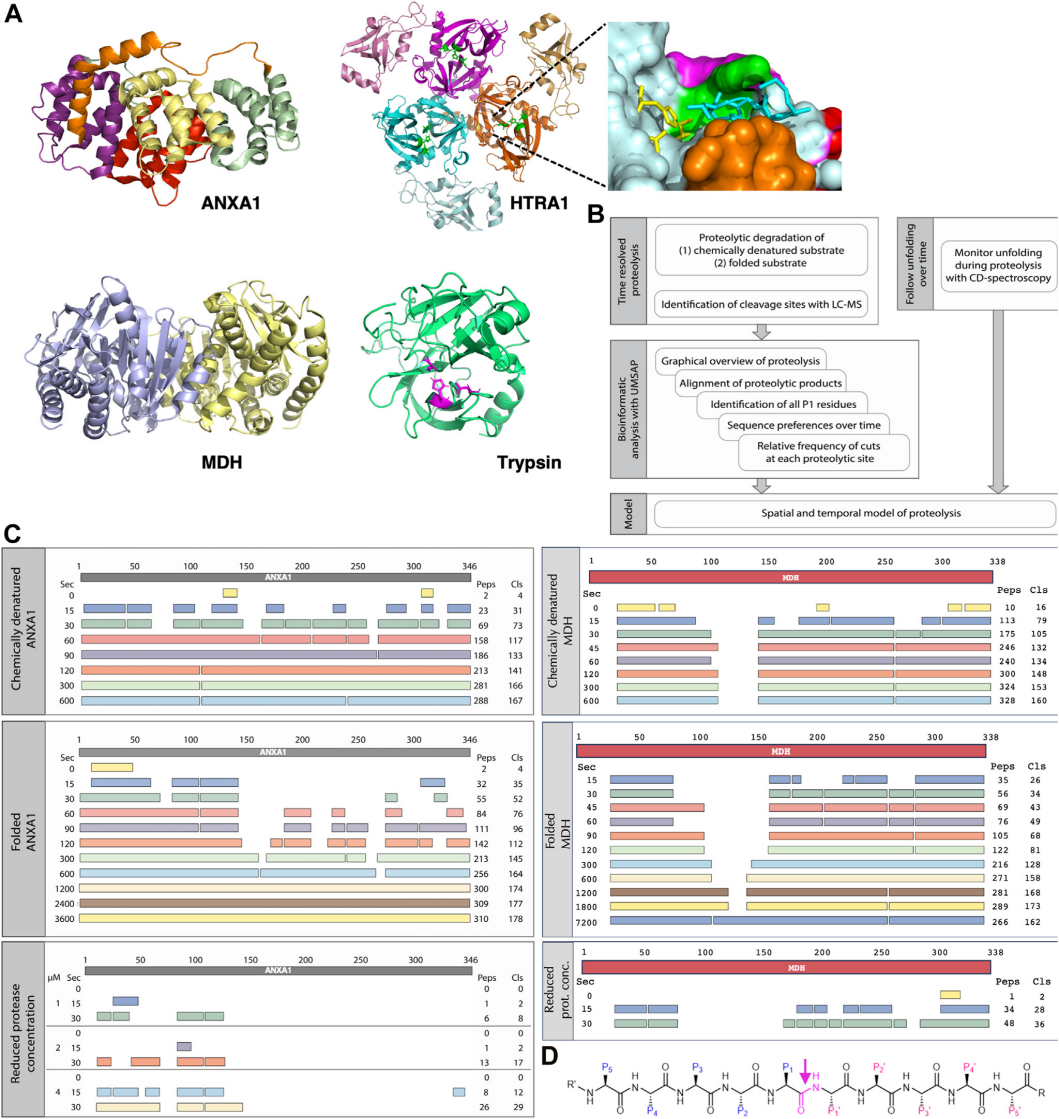
**Proteolytic degradation of ANXA1 by HTRA1 and of MDH by trypsin**. *A*, upper panel, structures of ANXA1 (pdb code: 1HM6) and HTRA1 (3NZI). ANXA1 consists of an N-terminal domain of 41 residues (*orange*) and a core domain consisting of four helical repeats of about 75 amino acid residues each (*purple*, *yellow*, *red*, and *green*). Model of the HTRA1 trimer. The protease domain was taken from 3NZI, the PDZ domains were modeled. The catalytic triad is shown in *green*. *Right*, close-up of one active site with bound DPMFKLV-boro (stick representation, *cyan*), catalytic triad (H220, D250, S328; stick representation, *yellow*) and the activation domain *i.e.*, loops L1 (*green*), L2 (*red*), L3 (*orange*), and LD (*magenta*). *Lower* panel, structures of MDH dimer (1MLD), individual protomers are color coded; and porcine trypsin (1AKS); residues of the catalytic triad His48, Asp92, and Ser185 are shown as *sticks* and in *magenta*. *B*, workflow, see text for details. *C*, *left*, proteolysis of chemically denatured and folded ANXA1 by HTRA1. *Top* bar: linear representation of the entire substrate protein. For each of the indicated time points (Sec), peptide sequences that align without gaps are grouped into so-called fragments, shown as *bars*. The number of peptides identified (Peps) and the total number of cleavage sites (Cls) are given at the right. The identified peptides aligned to the primary amino acid sequence of ANXA1 are provided in Supporting Data 1 (chemically denatured ANXA1), 3 (Folded ANXA1) and 4 (Folded ANXA1, reduced protease concentration). Right, proteolysis of chemically denatured and folded MDH by trypsin. The identified peptides aligned to the primary amino acid sequence of MDH are provided in Supporting Data 6 (chemically denatured MDH), 8 (Folded MDH) and 9 (Folded MDH, reduced protease concentration). *D*, model peptide and standard nomenclature. The residue of the scissile bond (*magenta*) is termed P1, which is often the major determinant of substrate specificity. Residues upstream to P1 are termed P2, P3, and so on. Accordingly, residues located downstream to P1 are termed P1′, P2', P3′ and so on (14). The proteolytic cleavage site is marked by an *arrow*. ANXA1, annexin A1; MDH, malate dehydrogenase; HTRA1, high temperature requirement A.

Recent proteomics and cell-based studies identified annexin A1 (ANXA1) as a native substrate of HTRA1 (8, 9) ANXA1 is a 39 kDa protein composed of 21 helices and connecting loops (Fig. 1*A*). The crystal structures of ANXA1 revealed that its N-terminal domain consists of 41 residues forming a helix and a loop, with the former inserted into the core domain. This core domain consists of 4 helical repeats of approximately 75 amino acid residues each (10, 11). In general, the annexin family of proteins is implicated in membrane biology including the repair of damaged cytoplasmic membranes (12). While several proteases perform N-terminal processing of ANXA1 (13), our previous data indicate that it can also be completely degraded by HTRA1 when mixing purified proteins (8). Therefore, ANXA1 and HTRA1 were chosen as a model to establish a simple experimental approach to study the degradation of a folded substrate at high temporal and spatial resolution (Fig. 1*B*). To demonstrate the general applicability of the workflow, an entirely different set of substrate and protease, *i.e.* porcine heart malate dehydrogenase (MDH) and trypsin (14), were used. While HTRA1 is a conformation specific protease with little substrate specificity, trypsin is a digestive protease with high substrate specificity. MDH is a widely conserved enzyme that catalyzes the conversion of oxaloacetate to malate (15) and is active as a homodimer (Fig. 1*A*) (16).

The key elements of this workflow are the mass spectrometry (MS)-based identification of proteolytic products, followed by bioinformatic characterization of proteolytic sites over a wide range of time points. Data analysis and presentation are supported by Utilities for Mass Spectrometry Analysis of Proteins (UMSAP), a software for the analysis of MS- data (17) (www.umsap.nl). In the context of targeted proteolysis, UMSAP uses a homogeneity of regression slope test to identify relevant proteolytic products whose sequences are aligned along the primary amino acid sequence of the substrate, calculates the relative frequency of cuts at each proteolytic site, as well as the preferences of the protease for the amino acid sequence surrounding the scissile bond (17). These data allow to postulate models of how progressive substrate processing causes substrate unfolding, which was verified by continuous CD spectroscopy. Therefore, this approach leads to a detailed understanding into the sequential events of initial substrate processing, concomitant unfolding, and final degradation into short peptides.

## Results

### Proteolysis of ANXA1 by HTRA1

The generation of a temporally and spatially resolved model of the proteolysis of folded ANXA1 requires a reference to identify all potential proteolytic sites and to distinguish those that are efficiently, poorly, or not at all cleaved, regardless of their accessibility in the folded substrate. Therefore, ANXA1 was chemically denatured in urea. The substrate was then diluted in buffer and incubated with stoichiometric amounts of HTRA1 at physiological temperature. Samples were taken at eight time points ranging from 0 to up to 600 s. Controls were denatured substrate and protease directly added to acetone. Four biological replicates of each sample were subjected to liquid chromatography-mass spectrometry (LC-MS) analyses to identify proteolytic products and the cleavage sites. The resulting datasets were analyzed using UMSAP software (17). For an initial graphical overview, UMSAP groups proteolytic products into fragments and provides the total number of cleavage sites at each time point (Fig. 1*C*). These data revealed 31 sites after 15 s and 167 sites after 600 s of incubation. The identified peptides span almost the entire sequence after 30 s except for short regions comprising residues 65 to 81, 146 to 160, 255 to 263, and 319 to 324. These gaps were not observed after the 90 s time point, indicating that substrate degradation was nearing completion (Fig. 1*C*, Supporting Data 1).

**Figure 2:**
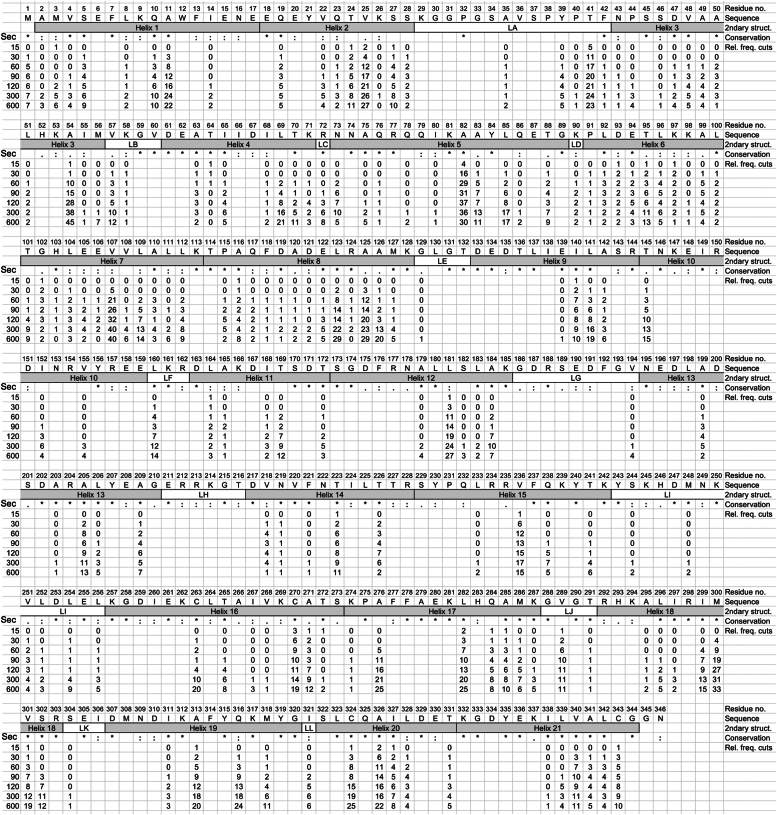
**Proteolysis of chemically denatured ANXA1 by HTRA1.** Proteolysis of chemically denatured ANXA1 was done as described in Fig. 1*B*. Samples were taken at the time points indicated (Sec) and proteolytic products of ANXA1 were identified by LC-MS. P1 residues identified in n = 4 experiments as significantly enriched compared to controls are represented by numbers. Structural elements *i.e.*, helices and loops (2ndary struct.) as detected in the crystal structure of folded ANXA1 (pdb:1HM6) are indicated. Amino acid sequence conservation (Conservation) is derived from a multiple sequence alignment (Supporting Data 2); ∗ = identical, : = conserved residues. All P1 residues identified in n = 4 independent experiments that were significantly enriched compared to controls are represented by numbers. Numbers below individual P1 positions indicate the relative frequency of cuts (Rel. freq. cuts) at each time point. No number indicates that no cleavage was detected at any of the time points investigated. ANXA1, annexin A1; HTRA1, high temperature requirement A; pdb, Protein Data Bank.

**Figure 3:**
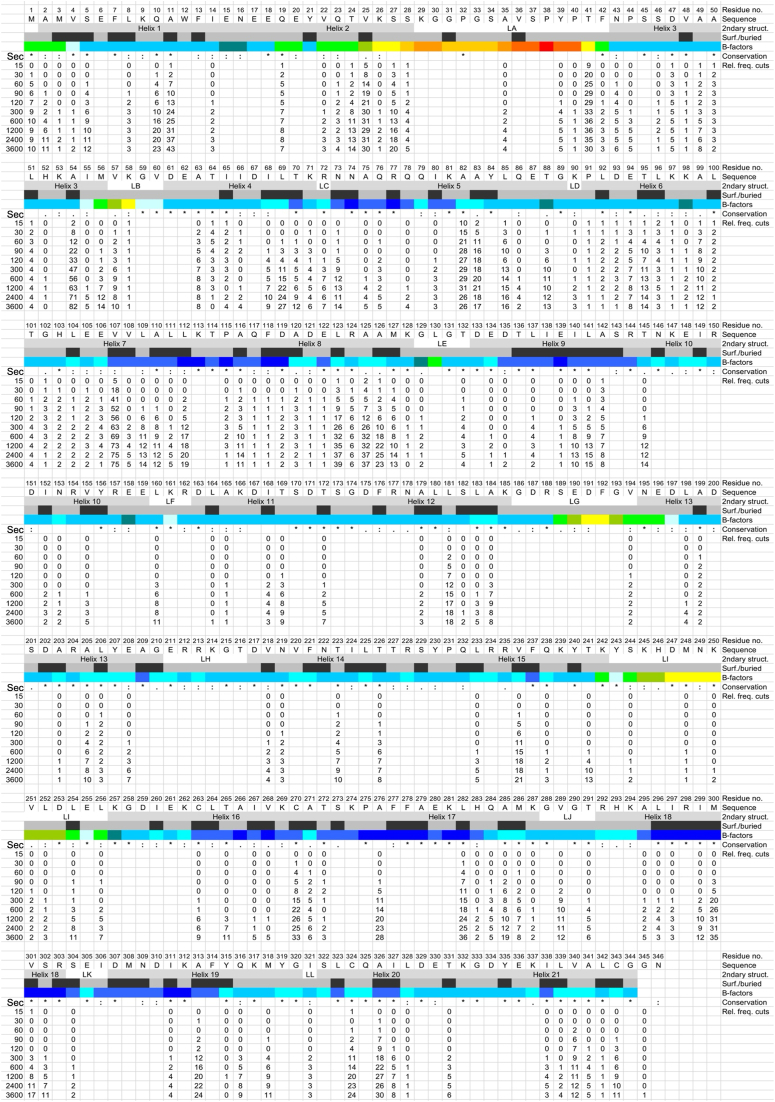
**Proteolysis of folded ANXA1 by HTRA1.** Proteolysis of folded ANXA1 was done as described in Fig. 1*B*. Samples were taken at the time points indicated (Sec) and proteolytic products of ANXA1 were identified by LC-MS. P1 residues identified in n = 4 experiments as significantly enriched compared to controls are represented by numbers. Structural elements *i.e.*, helices and loops (2ndary struct.), B-factors and surface accessibility (surf. (*gray*)/buried (*black*) as detected in the crystal structure of folded ANXA1 (pdb:1HM6) are indicated. The color code for B-factors indicates gradually structural rigidity (*blue*) to flexibility (*red*). Amino acid sequence conservation (Conservation) is derived from a multiple sequence alignment (Supporting Data 2); ∗ = identical, : = conserved residues. Numbers below individual P1 positions indicate the relative frequency of cuts (Rel. freq. cuts) at each time point. No number indicates that no cleavage was detected at any of the time points investigated. ANXA1, annexin A1; HTRA1, high temperature requirement A.

Common to the proteolysis of both unfolded and folded ANXA1 by HTRA1 is that, except for peptides generated from the extreme N- and C-terminal ends of the substrate, each proteolytic product is the result of two cuts, one at the N terminus, where the N-terminal residue represents the P1′ residue, and another at the C-terminus, representing the P1 residue (Fig. 1*D*). UMSAP also calculates the relative frequency of cuts at each P1 residue for each time point (see experimental procedures for details). These data provide quantitative information on how well HTRA1 cleaves the substrate at each position within its primary amino acid sequence and how proteolysis progresses over time (Figs 2 and 3). In addition, individual P1 residues can be grouped into several classes, based on histograms (Figs. S1 and S2). For denatured ANXA1, the first class included 17 P1 residues where the relative frequency of cuts reached ≥20 (Table S1). Among them, L181, A276, L282, M300, Q316, C324, and A326 are buried in the folded protein. Their efficient processing indicates that chemical denaturation has occurred. The second class consisted of 25 P1 residues where the relative frequency of cuts ranged from 11 to 19 (Fig. 2, Table S2). The third class consisted of residues where the relative frequency of cuts was between 1 and 10. These were considered poor sites at all time points, probably resulting from a low affinity to the active site. The fourth class consisted of residues for which no peptide products could be detected. This lack of detection could be explained by different models: (i) these sites did not bind to the active site, (ii) the resulting products were not detectable by MS or (iii) upon dilution of urea, this part of the substrate refolds into a stable conformation where the cleavage site remains inaccessible to the active site of HTRA1.

### Proteolysis of folded ANXA1 by HTRA1

Analysis of the degradation of folded substrate provides information on the surface accessibility of substrate binding sites and the degree of sequential structural relaxation and unfolding that occurs during progressive degradation. Therefore, ANXA1 was incubated with HTRA1, and samples were taken at 11 time points ranging from 0 s to up to 3600 s. The incubation time was extended because proteolysis of the folded substrate was slower than that of the chemically denatured substrate. These data revealed 35 cleavage sites after 15 s, 164 sites after 600 s, and 178 sites after 3600 s of incubation. In contrast to the degradation of denatured ANXA1, the identified peptides did not cover the entire sequence up to the 600 s time points. Complete coverage was only observed from the 1200 s time point onward (Fig. 1*C*, Supporting Data 3). When P1 sites were grouped into classes according to the maximum relative frequency of cuts performed at each site, class 1 (≥20 cuts) comprised 20 residues (Table S1) and class 2 (11–19 cuts) comprised 31 residues (Fig. 3, Table S2).

In contrast to the chemically denatured substrate, the first 35 cleavage sites at the 15 s time point, except for S302 and C324, were located exclusively in the N-terminal region of ANXA1 up to residue A142, comprising helices 1 to 9 (Fig. 3). The P1 sites with the highest relative cleavage frequencies at this time point are V25 in helix 2, T41 in loop LA, A82 in helix 5, and V107 in helix 7 (Fig. 3). The 178 cleavage sites identified across the entire ANXA1 sequence at 3600 s time point correlate with the 167 sites at the 600 s time point of the denatured substrate indicating that the extent of proteolysis of denatured and folded substrates was similar at these time points. The P1 sites with the highest relative frequencies of cuts are A11 in helix 1, A54 at the C terminus of helix 3, V107 in helix 7, L123 and A125 in helix 8, and L282 in helix 17.

These data suggest that early proteolytic events may destabilize the folded structure of the substrate, leading to accessibility of sites that were not exposed at the surface. Interestingly, the tendency for greater processing of the 9 N-terminal helices was also observed with chemically denatured protein (Fig. 2), suggesting that the C-terminal part of ANXA1 may have evolved to be more protease resistant, at least to HTRA1. The latter notion is supported by the larger gaps within the C-terminal half of ANXA1 (Figs. 2 and 3) as well as by the increased number of P1 sites that are cleaved more efficiently in denatured *versus* folded ANXA1 (Table S3).

In addition, we observed three features for folded and denatured substrate. The appearance of many products sharing only one identical cleavage site suggests that these products are the result of one high-affinity and several low-affinity binding sites (Supporting Data 1, 3, and 4). This model is supported by the observed tendency for longer proteolytic fragments, again sharing an identical cleavage site, to appear with higher frequency at later time points suggesting that these additional cleavage sites are of even lower affinity. In addition, sequences corresponding to the central parts of loops LA, LG, LH, and LI appear to be less well cleaved compared to helices. This may be due to their amino acid composition resulting in conformations that are inaccessible to the active site.

### Comparison of the amino acid sequences surrounding the cleavage sites in ANXA1

An important parameter that characterizes a protease is sequence specificity that is mediated by the specificity pockets of the active site. To obtain information on sequence specificity, UMSAP calculates the amino acid distribution around the scissile bond. When comparing the amino acid sequences surrounding the scissile bond (Fig. S2), the patterns of preferred residues at late time points (600 s for denatured and 3600 s for folded ANXA1) are very similar for the denatured and folded substrates *e.g.* at P1 sites, Ala (26.7 and 26.6%, respectively), Leu (16.8 and 16.4%), Val (14.0 and 13.6%), and Thr (11.9 and 12.2%) dominate, whereas Ala (13.5 and 13.5%) and Lys (12.0 and 11.5%) are enriched at P1′ sites. This feature is in agreement with published data (5). This similarity is also observed for the residues P2-P5, P2′and P5’ (Fig. S2). In contrast, the preferred residues differ at early time points and converge over time. These observations were also made when examining consensus sequences (Fig. S3). Here, the consensus sequences at each position were reached two to three time points earlier for denatured compared to folded ANXA1. These observations suggest that for the folded protein, the limited availability of the preferred small hydrophobic residues reflects conformational exclusion of these sites at early time points. This phenomenon is lost at later time points, probably because proteolytic digestion causes progressive substrate unfolding.

### Early events in the degradation of folded ANXA1 by HTRA1

Given the importance of initial events at early time points, we sought to increase the temporal resolution by reducing the number of initial cuts. We therefore reduced the concentration of protease 5-, 10-, and 20-fold, took samples at 0, 15, and 30 s and identified the proteolytic products by LC-MS (Fig. 1*C*, S4, Supporting Data 4). At 1 μM HTRA1 *i.e.*, at 20-fold molar access of substrate, a single peptide product was identified after 15 s of incubation, while six peptides were identified after 30 s, resulting from eight cuts within the N terminal 125 residues. At 2 μM HTRA1, again a single cut was identified after 15 s of incubation, while 13 peptides were identified after 30 s, resulting from 17 cuts within the N-terminal 126 residues. At 4 μM HTRA1, eight peptides were identified resulting from 12 cuts after 15 s, while 26 peptides were identified resulting from 29 cuts after 30 s of incubation. One additional product was identified located at the C terminus, resulting from cuts at P1 residues A326 and V340. Because this peptide was detected only at the 15 s but not at the 30 s time point and only from the 60 s time point onward at 20 μM HTRA1 concentration (Supporting Data 3), this peptide was omitted from the sequential substrate unfolding model described below.

The analysis of initial events must consider the P1 residues *per se*, but also the peptide products resulting from additional cuts occurring either upstream or downstream of the P1 residue. For example, cuts at A82 result in six products at 2 μM HTRA1 at 30 s that are generated by additional cuts at E94, L96, K97, A99, G102, and V107. At 4 μM HTRA1 and 30 s, cuts at A82 result in nine products that are generated from three additional cuts at D93, T95, and L100. Another example is residue T41. Here, additional cuts upstream, *i.e.*, at V25, K26, and K29 as well as downstream at A54, K58, and T64 produce six peptides. These events lead to the efficient degradation of the region from V25 to T64, with the cut at T41 being the main event (Supporting Data 4).

### Model of sequential unfolding of ANXA1 by progressive proteolysis

**Figure 4:**
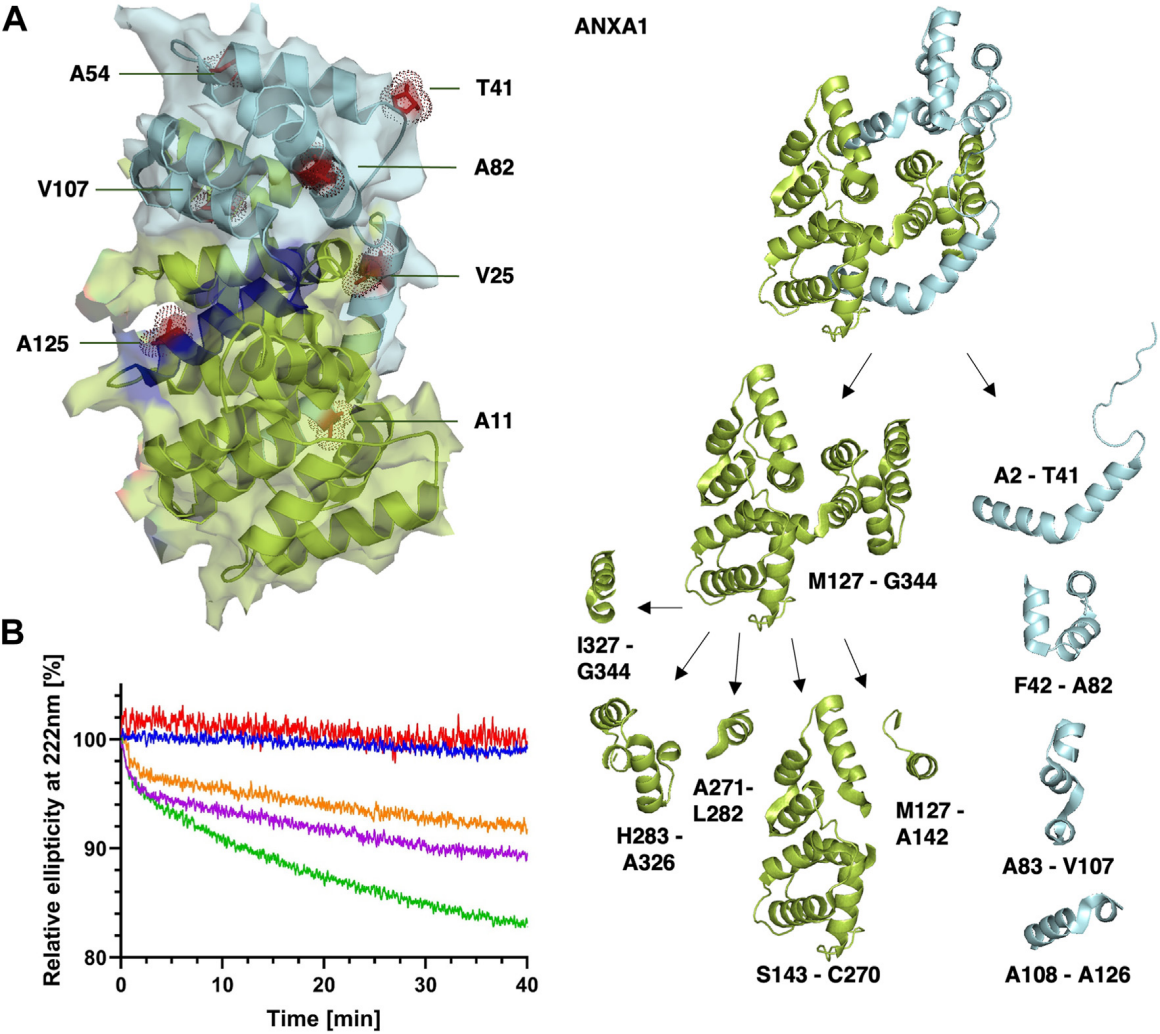
**Early cleavage events in folded ANXA1 and unfolding by fragmentation.***A*, *Left*, surface representation of ANXA1 (side view). Key P1 residues are shown as *stick* and *dot* representation. Surface exposed residues are shown in *red*, buried residues in *orange*. *Right*, model of how folded ANXA1 (top view) is first converted into 4 N-terminal fragments comprising residues A2-A126 (*marine*) generating a C-terminal fragment comprising M127-G344 (*limon*). Subsequently, the C-terminal fragment is further processed into five fragments. Helix 8 is highlighted in *dark blue*. Note that A11 is located in the background and therefore masked by the C-terminal domain. *B*, CD spectroscopy. HTRA1 and ANXA1 were mixed in a 1:1 (*green*), 1:10 (*magenta*) and 1:20 (*orange*) ratio and the ellipticity at 222 nm was monitored over time. To facilitate comparison between datasets, in each case the initial signal was set at 100% and the loss of the negative ellipticity at 222 nm is shown as a percentage of the overall signal. Briefly, 5 μM HTRA1 alone (*red*) or 5 μM ANXA1 alone (*blue*) control experiments are also shown. ANXA1, annexin A1; HTRA1, high temperature requirement A.

A model of sequential unfolding by proteolytic fragmentation can be postulated by considering the occurrence of cuts at low HTRA1 concentrations at early time points in combination with high relative frequency of cuts at individual P1 residues at higher HTRA1 concentrations. Taken together, our data suggest that the degradation of ANXA1 starts at its N terminus, resulting in fragmentation up to helix 8, followed by the degradation of the remaining C-terminal parts (Fig. 4*A*).

Residues T41, A82, and V107 are prime candidates for early and high frequency cleavages. Among these, the surface-exposed and nonconserved T41, located at the fusion junction of the N terminal and core domain of ANXA1, and at the C terminal end of the large loop LA, is likely to be the first P1 residue to be cleaved (Fig. 3). Subsequently, the site at V25, located at the C terminus of helix 2 immediately preceding loop LA, may be cleaved next. This cut is likely to be followed by cleavage at A11, an event that would require helix 1 (residues 2–17) to move away from the folded structure, which is likely to occur after the position of helix 2 (residues 8–28) has been destabilized by the cut at V25. In parallel, cuts after A82, located in the middle of helix 5, will cleave this helix in half. This event is likely to destabilize the positions of helices 3 and 4, allowing a cut at A54 near the end of helix 3. With respect to V107, this cut in the middle of helix 7 should allow for subsequent cuts at A125/A126 in helix 8 that are completing the removal of the N terminal 8 helices from the folded structure (Fig. 4*A*).

Subsequent processing of the remaining C-terminal part of ANXA1 involves early cuts at A142, C270, L282, and A326 producing five fragments, *i.e.*, M127-A142, S143-C270, A271-L282, H283-A326, and I327-G344. Subsequent proteolytic events involve I140, L181, T223, V236, A313, C324, and V340. Finally, all fragments are further processed by multiple cuts. The regions in which no cuts were observed at the latest time points examined comprise no more than nine residues *i.e.*, K185-G193 and A209-D217, suggesting that unfolding and degradation of the substrate is complete.

### CD spectroscopy during proteolysis of ANXA1

To independently follow substrate unfolding, CD spectroscopy was used (Figs. 4*B*, S5). The CD spectra of ANXA1 and HTRA1 alone exhibited the classic double minimum (208 nm/222 nm) allowing us to monitor the α-helical content at 222 nm. Since ANXA1 is composed only of α -helices and HTRA1 predominantly of β-sheets, the α-helical signal of HTRA1 was significantly lower than that of ANXA1. When ANXA1 was mixed with HTRA1 and subjected to CD spectroscopy after 5 h of digestion, a 75% reduction of the ANXA1 signal at 222 nm, but only a 50% signal reduction at 208 nm is observed, with the spectral minimum being shifted to the lower wavelength (Fig. S5). This suggests that proteolytic fragmentation is indeed associated with a loss of ANXA1 structure as indicated by the overall loss of signal intensity, while the shift to lower wavelengths indicates increased random coil content.

The decrease of α-helical content of the folded structure in the digest reaction was followed continuously by measuring the CD signal at 222 nm over time (Fig. 4*B*). Notably, an initial exponential burst phase during the first 60 s was observed, which correlates with the fast initial cuts mainly at P1 residues T41, A82, and V107. This relatively rapid loss of signal indicates that the peptide products resulting from cuts at these sites is accompanied by their rapid dissociation and unfolding, suggesting that both the tertiary contacts and the contiguity of the polypeptide chain is necessary to stabilize the secondary structure of the cleaved peptides within the intact protein. This fast initial phase is followed by a more slowly proceeding phase, indicating a likely scenario where the remainder of the undigested ANXA1 has sufficiently destabilized tertiary structure and core hydration that unfolding events take place. This results in a competition between refolding and proteolysis, which eventually leads to a progressive digestion of the whole ANXA1 at available sites along its linear sequence, although it is possible that some residual structure may remain and occlude sites leading to slower digestion. This is supported by the fact that as the substrate concentration is reduced by proteolytic degradation, the rate of the gradual loss of the CD signal following the initial burst phase decreases (Fig. 4*B*).

### Proteolysis of MDH by trypsin

**Figure 5:**
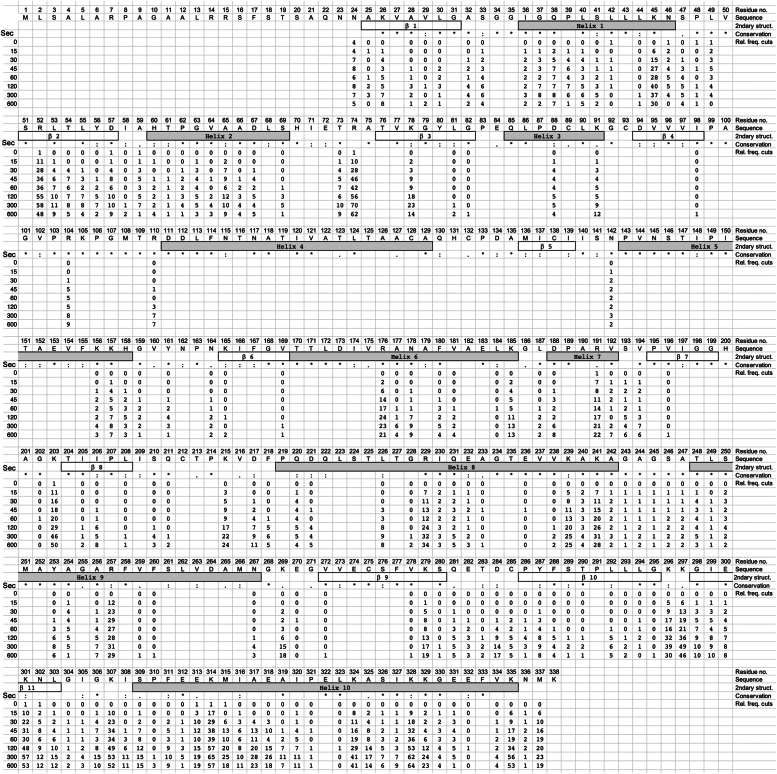
**Proteolysis of chemically denatured MDH by trypsin.** Proteolysis of chemically denatured MDH was done as described in Fig. 1*B*. Samples were taken at the time points indicated (Sec) and proteolytic products of MDH were identified by LC-MS. Structural elements *i.e.*, helices and loops (2ndary struct.) as detected in the crystal structure of folded MDH (pdb:1MLD) are indicated. Amino acid sequence conservation (Conservation) is derived from a multiple sequence alignment (Supporting Data 7); ∗ = identical, : = conserved residues. All P1 residues identified in n = 4 independent experiments that were significantly enriched compared to controls are represented by numbers. Numbers below individual P1 positions indicate the relative frequency of cuts (Rel. freq. cuts) at each time point. No number indicates that no cleavage was detected at any of the time points investigated. MDH, malate dehydrogenase.

To validate the approach, an additional substrate and protease set was analyzed. MDH and trypsin were chosen because, unlike the helical-only protein ANXA1, MDH contains helices and β-sheets and is a homodimer (Fig. 1*A*) (16). Trypsin was chosen because it is a sequence-specific, digestive protease, whereas HTRA1 is a conformation-specific protease (1, 14). To identify all cleavage sites, chemically denatured MDH was digested with trypsin. For this, MDH was chemically denatured in urea and then diluted into reaction buffer. Subsequently, 20 μM MDH was digested with 4 nM trypsin. Samples were taken at 8 time points ranging from 0 to up to 600 s. The initial graphical overview generated by the UMSAP software revealed 79 cleavage sites after 15 s and 160 sites after 600 s of incubation (Fig. 1*C*). The identified peptides span almost the entire sequence after 30 s except for short regions encompassing residues 92 to 103, 105 to 141 and 143 to 155 (Figs. 1*C* and 5). In contrast to the degradation of ANXA1 by HTRA1, these gaps remained at later time points, indicating that substrate degradation was nearing completion at 30 s of incubation (Figs. 1*C* and 5, Supporting Data 6). The gaps are best explained by the specificity of trypsin for Lys and Arg residues at P1 residue, none which are present in the sequences covered by the gaps (Fig. 5).

Furthermore, based on histograms (Fig. S6), individual P1 residues can be grouped into several classes. The first class included 13 P1 residues where the relative frequency of cuts reached ≥30 (Table S4). Among them, K45, R52, R74, and R229, for example, are buried in the folded dimer. Their efficient processing indicates that chemical denaturation has occurred. The second class consisted of 8 P1 residues with a relative cut frequency between 21 and 30 (Fig. 2, Table S5). It should be noted, however that although the majority of frequently cleaved sites had a Lys or Arg residue at P1 positions, as indicated by comparison of the amino acid sequences surrounding the scissile bond (Figs. S7 and S5), numerous minor cleavage sites containing other residues were also detected, suggesting that trypsin does not have an absolute specificity for positively charged residues at P1 position, as previously reported (18).

### Proteolysis of folded MDH

**Figure 6:**
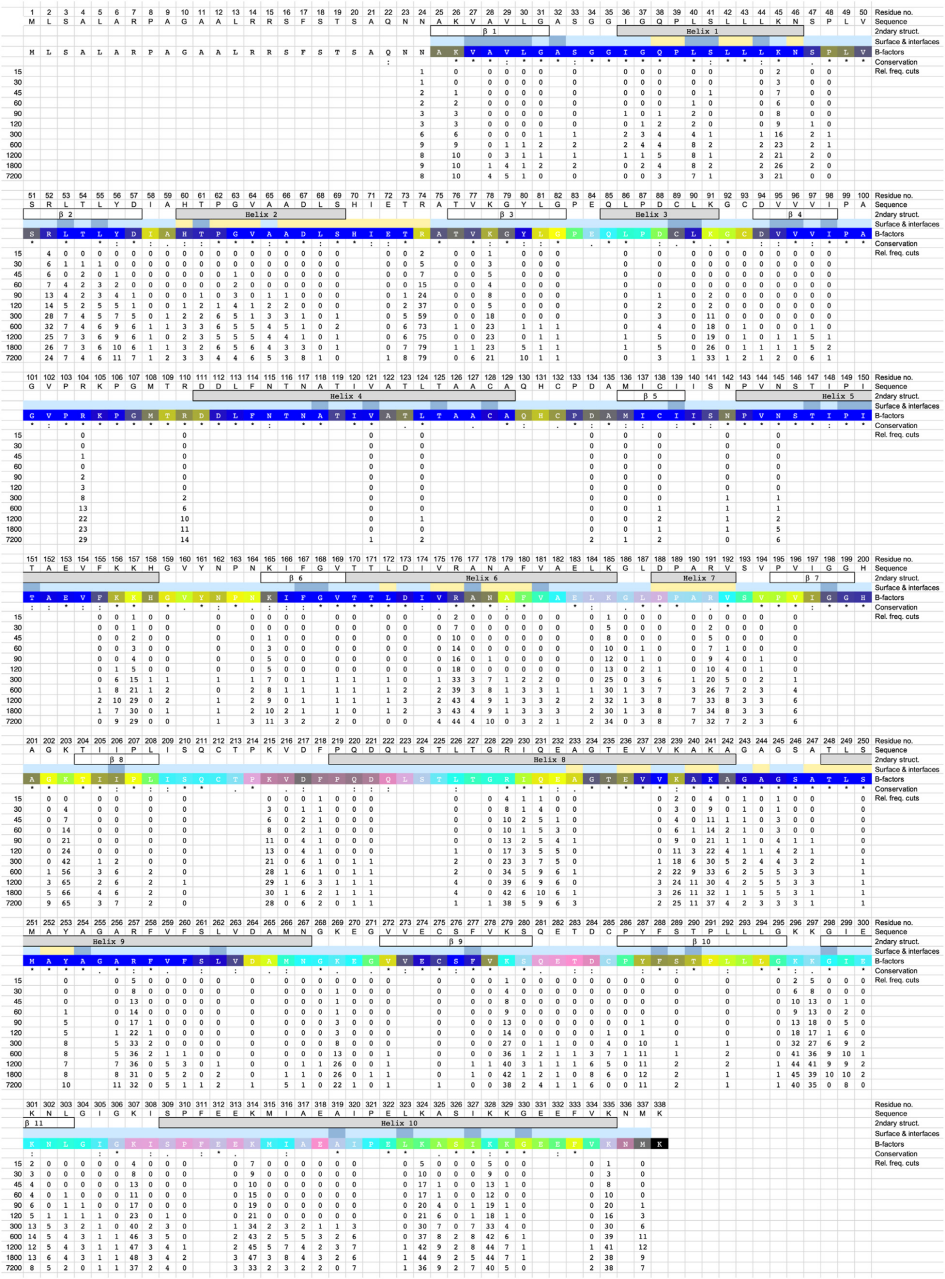
**Proteolysis of folded MDH by trypsin.** Proteolysis of folded MDH was done as described in Fig. 1*B*. Samples were taken at the time points indicated (Sec) and proteolytic products of MDH were identified by LC-MS. P1 residues identified in n = 4 experiments as significantly enriched compared to controls are represented by numbers. Structural elements *i.e.*, helices and loops (2ndary struct.). Surface accessibility (solvent accessible (*light blue*), solvent inaccessible (*dark blue*), and interfacing residues (*yellow*) as detected in the crystal structure of folded MDH (pdb:1MLD) are indicated. The color code for B-factors indicates gradually structural rigidity (*blue*) to flexibility (*lighter* colors). Amino acid sequence conservation (Conservation) is derived from a multiple sequence alignment (Supporting Data 7); ∗ = identical, : = conserved residues. Numbers below individual P1 positions indicate the relative frequency of cuts (Rel. freq. cuts) at each time point. No number indicates that no cleavage was detected at any of the time points investigated. MDH, malate dehydrogenase.

To analyze the sequential structural relaxation and unfolding that occur during progressive degradation, 20 μM of folded MDH was incubated with 4 nM trypsin at 37 °C and samples were taken at 12 time points ranging from 0 s to up to 7200 s. The graphical overview revealed 26 cleavage sites after 15 s, 158 sites after 600 s and between 163 and 173 sites at late time points *i.e.*, between 1200 and 7200 s of incubation (Fig. 1*C*). Up to the 600 s time points, the gaps observed with denatured substrate were also observed. Only at 7200 s of incubation, these gaps disappeared due to cuts at minor cleavage sites that did not contain Lys or Arg residue at P1 position, leading to complete degradation (Fig. 1*C*, Supporting Data 8). Based on histograms (Fig. S6), individual P1 residues can be grouped into several classes. The first class included 19 P1 residues where the relative frequency of cuts reached ≥30 (Fig. 6, Table S4). The second class consisted of 9 P1 residues where the relative frequency of cuts was between 21 and 30 (Fig. 6, Table S5).

A comparison of the number of cuts at major proteolytic sites at *e.g.* the 120 s time point in denatured and folded MDH, indicates that even though trypsin is a sequence-specific protease, the conformation of the substrate and the resulting surface accessibility of the cleavage sites is an important determinant of efficiency, as exemplified by P1 positions K45 (40 and 9 cuts in denatured and folded MDH, respectively), R52 (55 and 14), R74 (56 and 37), K239 (20 and 12), K301 (48 and 5), K328 (53 and 18), and R335 (34 and 16). At the 600 s time point, the P1 residues R74 (62 and 73 cuts in denatured and folded MDH, respectively), K203 (50 and 56), K328 (64 and 42), K307 (52 and 46), and K314 (57 and 43) exhibit the highest relative frequency of cuts (Table S4). In an extended analysis, 19 P1 residues corresponding to major cleavage sites were found to be cleaved more extensively at earlier but not at later time points in denatured compared to folded MDH (Table S6). Also, as observed for the denatured substrate, residues 111 to 155 of MDH were not or only rarely cleaved by trypsin because they do not contain positively charged residues (Fig. 6).

Of the various residues that are involved in substrate and cofactor NAD^+^ binding or catalysis (16), R104 and R176 involved in substrate binding are well cleaved, with R176 being cleaved early *i.e.* >30 times and <40 times at the 300 and 1200 s time point onward, respectively while R104 is cleaved >20 times at the 1200 s time point. In addition, R110, located in the substrate binding site, is also cleaved, but with about 2-fold lower efficiency compared to R104. All other residues of this class are not or poorly cleaved, probably because they are not positively charged (Fig. 6).

### Early events in the degradation of folded MDH

Given the importance of initial events for structural relaxation and unfolding of the substrate, we sought to increase the resolution by reducing the number of initial cuts by lowering the concentration of protease by 2-fold and taking samples at 0, 15, and 30 s. LC-MS detected 2, 28, and 36 cuts, respectively (Figs. 1*C* and S8, Supporting Data 9). As for ANXA1 digested by HTRA1, the analysis of the initial events must consider the P1 residues *per se* but also the peptide products resulting from additional cuts occurring either upstream or downstream of the main P1 residues. For example, at the 15 s time point, cuts at R52 resulted in four products that are generated by additional cuts at N24, K45, R74, and K78; or cuts at K241 resulted in three additional products that were also cleaved at Q231, I230, and R229, while cuts at R257 resulted in five products that are generated by additional cuts at K239, K241, G243, A244, and G245 (Supporting Data 9).

In addition to the previously identified major P1 sites R52 (4 and 6 cuts at the 15 and 30 s time points, respectively), R74 (2 and 6), K203 (1 and 3), R257 (5 and 6), K307 (5 and 6), K314 (8 and 18), and K328 (4 and 8), the P1 sites R176 (2 and 7), R191 (2 and 6), K215 (1 and 7), R229 (3 and 6), K241 (3 and 7) and K324 (5 and 9) were also cleaved. These additional 6 sites were also well cleaved at higher protease concentrations but missed the cut-off of > 10 and > 50 cuts at the 15 s and 600 s time points, respectively (Fig. S8).

### Model of sequential unfolding of MDH by progressive proteolysis

**Figure 7:**
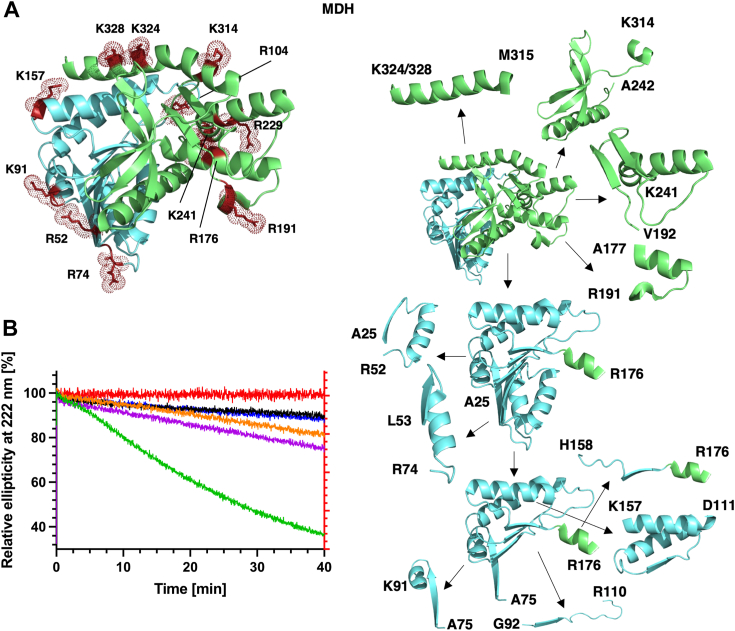
**Early cleavage events in folded MDH and unfolding by fragmentation**. *A*, *left*, cartoon representation of the MDH structure. Key P1 residues (*red*) are shown as *sticks* and *dot* representation. *Righ*t, model of the sequential fragmentation of folded MDH: First, the C-terminal part of MDH comprising residues A177-K338 (*green*) is detached by fragmentation. Second, the remaining N-terminal fragment (*cyan*) is reduced to A75-R176, by cuts at R52 and R74. Subsequently, A75-R176 is processed into 4 fragments. *B*, CD spectroscopy. Trypsin and MDH were mixed in a 1:500 (*green*), 1:5000 (*magenta*), 1:10,000 (*orange*), and 1:100,000 (*black*) ratio and the ellipticity at 222 nm was monitored over time. To facilitate comparison between datasets, in each case the initial signal was set at 100% and the loss of the negative ellipticity at 222 nm is shown as a percentage of the overall signal. 0.01 μM trypsin alone (*red*) or 5 μM MDH alone (*blue*) control experiments are also shown. The right *Y* axis is used for the trypsin only control because, due to its low concentration, the signal was much lower compared to the MDH signals. MDH, malate dehydrogenase.

As with ANXA1, a model of sequential unfolding by proteolytic fragmentation of MDH is derived from cuts at low protease concentrations at early time points in combination with high relative frequency of cuts at P1 residues at higher trypsin concentrations. This model predicts that the degradation of MDH occurs in three sequential steps. Initially, the C-terminal part comprising residues A177-K338 is detached by fragmentation. Simultaneously, the remaining N-terminal fragment comprising A25-R176 is reduced to A75-R176, which is then further processed into four fragments. All relevant cuts occur at major sites containing positively charged residues (Fig. 7*A*).

Specifically, the degradation of MDH begins at the C terminus with cuts at K314, K324, and K328. These cuts remove helix 10 from the folded structure. The removal of the long helix 10 suggests several consequences. First, its N terminus interacts with the surface accessible helix 8. Relaxation of the position of helix 8 should result in subsequent processing of R229 and K241 and thus loss of helix 8. Second, since helix 10 also interacts with β-strand 8, its absence is expected to weaken the interaction of β-strands 7 and 8, and therefore the position of the adjacent short helix 7, allowing cuts at the major proteolytic site R191. This cut should relax the position of the buried helix 6, allowing the processing at the major proteolytic site R176. Third, at the N-terminal end of helix 10, a loop containing K307 is also expected to be cleaved, followed by cuts at K296 and K297. These residues form a short loop connecting β-strands 10 and 11. Separation of β-strands 10 and 11 allows processing at K279 in β-strand 9 as well as of R257 which is located in the middle of helix 9, completing the digestion of the C terminal half of MDH. Fourth, the absence of helix 10 is also expected to destabilize and relax the position of helix 5, which is expected to affect helix 4, which interacts with helix 5. Further digestion of helices 4 and 5 does not occur because they are located within the gap that is not proteolyzed before the 300 s time point (Figs. 6, 7*A*, and S8).

The concomitant degradation of the N terminal part of MDH is expected to be initiated later as it is more buried and/or involved in interprotomer interactions. Initial processing of the major proteolytic site R52, located on β-strand 2 may allow processing of the downstream major proteolytic site R74, located in a loop connecting helix 2 and β-strand 3, and the minor proteolytic site K78 in β-strand 3, resulting in destabilization of the N-terminal portion of MDH. The following region consisting of residues G79-N164 containing helices 3 to 5 and β-strands 4 and 5, helix 4, is not cleaved at early time points at lower trypsin concentration. This region is digested at later time points and higher protease concentration, *i.e.*, by cuts at K91 (helix 3), R104 and R110 (both in the loop connecting β-strand 4 and helix 4, and K157 in helix 5. At later time points, cleavage at K45 (in helix 1) completes processing of the N terminal part of MDH. The level of proteolysis required to cause dissociation of the oligomer is unknown but it could be speculated that early cuts at K241 in helix 8 and R176 in helix 6, followed by cuts R74 in a loop connecting helix 2 and β-strand 3, R191 in helix 7 at later time points *i.e.* 60 s or later, are the major contributors to oligomer dissociation (Figs. 6, 7*A*, and S8). Considering that helices 10 are located at opposite ends of the MDH dimer, it appears that proteolytic degradation is initiated at the surfaces furthest away from the protomer interface and progresses sequentially toward the dimer core (Fig. 1*A*).

### CD spectroscopy during proteolysis of MDH

To independently follow substrate unfolding, CD spectroscopy was used (Figs. 7*B* and S9). The CD spectra of MDH and trypsin alone exhibited the classic double minimum (208 nm/222 nm) allowing us to monitor the α-helical content at 222 nm. Since MDH consists of 10 α -helices (and 11 β-strands) and trypsin predominantly of β-sheets, the α-helical signal of trypsin was significantly lower than that of MDH. When MDH was mixed with trypsin and subjected to CD spectroscopy after 5 h digestion, > 80% reduction of the MDH signal at 222 nm was observed, but only a 45% signal reduction at 208 nm, with the spectral minimum being shifted to the lower wavelength (Fig. S9). This suggests that proteolytic fragmentation is indeed associated with loss of MDH structure as indicated by the overall loss of signal intensity while the shift to lower wavelengths indicates increased random coil content.

The decrease in the α-helical content of the folded structure in the digestion reaction was continuously followed by measuring the CD signal at 222 nm over time (Fig. 7*B*). In contrast to ANXA1/HTRA1, an initial exponential burst phase was not observed. Instead, following a slight lag-phase of 5 to 10 min, a rather continuous loss of signal was observed suggesting a progressive digestion of the folded protein. Furthermore, as with ANXA1/HTRA1, the degree of signal loss over time correlates with the amount of protease.

## Discussion

The HtrA family of proteases, including HTRA1, plays a crucial role in maintaining proteostasis by degrading misfolded, damaged, or fragmented proteins. This process is essential for preventing the accumulation of potentially toxic protein aggregates, which can lead to various diseases. The present study explores how HTRA1 not only targets misfolded proteins but also degrades proteins in their folded conformation. Understanding this broader substrate range can provide insights into the physiological roles of HTRA1 and its involvement in various cellular processes. The additional biological relevance of this work is the advanced understanding of proteolysis by providing a detailed, high-resolution view of how proteins are degraded in sequential steps, which is critical for understanding protein turnover and maintenance. The quantification of cleavage events at each proteolytic site over time offers a new perspective on the relationship between structural flexibility, sequence conservation, and proteolytic processing. In addition, unexpected cleavage patterns observed in regions of structural flexibility and low sequence conservation in the substrate suggest evolutionary adaptations to protease activity, which could impact protein function and stability.

Our approach combines time-resolved MS identification of peptide products, the corresponding protease cleavage sites, and the relative frequency of cuts at each specific site. Furthermore, correlating the temporal occurrence and the location of the observed cuts in the folded substrate reveals the sequential degradation of the substrates at high temporal and spatial resolution. These data provide detailed information on substrate degradation that does not require prior modification and ATP-driven unfolding and proteolysis as exemplified by the ubiquitin, p97, proteasome system (19).

Our data provide insights into how a folded substrate is degraded in sequential steps *i.e.*, from initial processing followed by local structural relaxation to stepwise unfolding of the substrate and subsequent complete degradation of the initially produced large fragments into short peptide products. This approach could be applied to hetero-oligomeric complexes and other structures such as disease-related amyloid fibrils, whose dissociation and degradation is not yet understood at high resolution (20, 21, 22). The resulting datasets might serve as a basis for further improvements of downstream computational processing such as the development of predictive algorithms regarding structural changes caused by proteolytic processing as well as for conformational and amino acid sequence selectivity of the proteases under investigation. The generated data will also allow to postulate experimentally testable hypotheses about the structural and functional consequences of missense mutations identified in pathological events such as cancer and other genetic diseases. In addition, the data reveal proteolytic hotspots, where proteolysis is initiated, leading to substrate unfolding. This knowledge could be used to eliminate these sites in a designed or engineered protein to confer a degree of protease resistance.

One limitation of the workflow concerns peptides from poorly cleaved sites, as their reproducibility of detection is not as robust as for well-cleaved sites. We have therefore focused our data interpretation on well-cleaved sites. A related limitation is due to MS constraints *i.e.*, the inability to detect specific peptide fragments in an unpredictable manner. However, this problem is likely to soften given the continuous development of new instruments with improved resolution and method development toward optimized sample preparation.

Our data provide additional insights. Interestingly, B-factors and evolutionary conservation of amino acid sequences, both typically used to distinguish between regions of high and low structural rigidity and relevance, respectively, can be directly correlated with the efficiency of proteolytic processing. That is, regions of high structural flexibility and low sequence conservation as observed in ANXA1 for loops LA, LB, LG, and LI would suggest efficient proteolysis, whereas structurally rigid and conserved parts such as helices 5, 8, and 9 would suggest less efficient proteolysis. Our data did not show the expected correlation, as these loops were less processed compared to these helices (Fig. 3). Similar observations were made in an N-terminomics study involving the proteases caspase 3 and glutamyl endopeptidase (23). This curiosity is best explained by an evolutionary adaptation toward sequences with low affinity to the active site of the protease or conformational rigidity, thereby allowing the protein to retain regions of surface-exposed structural flexibility. The opposite of this feature is observed, for example, in natural serine protease inhibitors (serpins) where a conserved surface-exposed loop has evolved to be a substrate of proteases that are inhibited by efficient serpin processing because one proteolytic product remains covalently bound to the catalytic Ser residue (24).

Another rather unexpected feature was the generation of multiple proteolytic products resulting from one identical site that was cleaved with high efficiency and several additional secondary cleavage sites processed with lower efficiency. A related phenomenon was that larger peptide products of this class were produced at later time points in contrast to the expectation that larger products would be produced early to be further degraded over time. Again, these larger products are likely the result of a high-affinity cleavage site at one end and a secondary site of lower and rate-limiting affinity.

The wider implications of our workflow are that in addition to studying protein degradation, it could be adapted to study other posttranslational modifications such as phosphorylation in dynamic protein complexes. In this case, MS would identify phosphorylated residues, rather than proteolytic products. In analogy to the current study, the incubation of the chemically denatured proteins with a protein kinase would identify all potential phosphorylation sites. The subsequent mapping of these sites over time on the intact protein complex in different functional states would provide valuable information about the conformational differences and the resulting changes in phosphorylation patterns, the functional implications of which can be addressed experimentally.

## Experimental procedures

### Purification of HTRA1 and ANXA1

As the activity of HTRA1 does not depend on its N-terminal domain (4), we used a derivative composed of the protease and PDZ domain in this study. Human HTRA1 comprised residues 158 to 480 and was purified as published previously (21) with minor alterations: HTRA1 carried an N-terminal StrepII-tag. Therefore, affinity-chromatography was performed with a strep-tactin resin (IBA Lifesciences). Human ANXA1 was expressed in *Escherichia coli* (BL21 Rosetta 2) grown in LB medium. Protein production was induced with 300 μM IPTG for 3 h at 37 °C. ANXA1 was affinity purified *via* a Ni-NTA superflow column (Qiagen), eluted stepwise with increasing concentrations of imidazole (12.5, 25, 50, 100, and 250 mM) and further purified by size exclusion chromatography using a Superdex 200 preparation grade column (GE HealthCare) in 20 mM Hepes, 50 mM NaCl, pH 7.5. Protein concentrations were determined *via* Bradford assays and SDS-PAGE.

### Time-resolved proteolysis of ANXA1 by HTRA1

Proteolysis of folded recombinant ANXA1 was done by mixing 20 μM ANXA1 with 20 μM HTRA1 in 150 mM NaH_2_PO_4_, 380 mM NaCl, pH 8. Samples were taken at the time points indicated. Alternatively, for the proteolysis of chemically denatured substrate, ANXA1 was denatured in 8 M urea. Denatured ANXA1 was diluted 13-fold into 150 mM NaH_2_PO_4_, 380 mM NaCl, pH 8 containing 0.6 M urea before adding HTRA1. For MS-analysis 5 μl of each sample were added to 30 μl ice-cold acetone for precipitation at −80 °C overnight. Precipitated proteins were sedimented (20,000g, 4 °C, 1 h) and the supernatant was lyophilized in a SpeedVac centrifuge (Eppendorf) at 30 °C for 90 min.

### Time-resolved proteolysis of MDH by trypsin

Time-resolved proteolysis of MDH (Roche, LMDH-RO) by trypsin (Sigma-Aldrich, T-1426) was performed as described for ANXA1 and HTRA1, except for the buffer composition (50 mM Tris, pH 8) and the concentration of trypsin (4 or 2 nM). For proteolysis of denatured substrate, MDH was diluted 20-fold into 50 mM Tris, pH 8 before adding trypsin.

### LC/MS/MS

Experiments were performed on an Orbitrap Elite or Fusion Lumos mass spectrometer (Thermo Fisher Scientific) that was coupled to an Evosep One liquid chromatography (LC) system (Evosep Biosystems). Analysis on the Evosep One was performed on a commercially available EV-1064 Analytical Column–60 & 100 samples/day (Length (LC) 8 cm; ID 100 μm; OD 360 mm; emitter EV-1086 Stainless steel emitter). The LC system was equipped with two mobile phases: solvent A (0.1% formic acid, in water) and solvent B (0.1% formic acid in acetonitrile). All solvents were of UHPLC (ultra-high-performance liquid chromatography) grade (Honeywell). For analysis with the Evosep One, samples were first loaded onto Evotips by following the manufacturer's guidelines. For peptide separation, we used 60 samples per day gradient which has an effective gradient of 21 min.

The mass spectrometers were operated using Xcalibur software (Elite: v2.2 SP1.48 or Lumos: v4.5.445.18). The mass spectrometers were set in the positive ion mode. Precursor ion scanning (MS1) was performed in the Orbitrap analyzer (Fourier transform mass spectrometry with the internal lock mass option turned on (lock mass was 445.120025 m/z, polysiloxane) (25). MS2 product ion spectra were recorded only from ions with a charge greater +1 and in a data dependent fashion in the ion trap mass spectrometry. All relevant MS settings (Resolution, scan range, AGC, ion acquisition time, charge states isolation window, fragmentation type and details, cycle time, number of scans performed, and various other settings) for the individual experiments can be found in Supporting Data 10.

### Peptide and protein identification using MaxQuant

RAW spectra were submitted to an Andromeda (26) search in MaxQuant (version 2.0.2.0 or 2.0.3.0) (www.maxquant.org) using the default settings *e.g.* search for peptides between 8 and 25 residues (27). Label-free quantification (28) and the match between runs was activated. Normalization in MaxQuant was switched off. MS/MS spectra data were searched against the ACE_0653_UP000000625_83333.fasta (4450 entries) custom database. The search database contains the Uniprot reference database for *E. coli* supplemented by the sequences of HTRA1 (Q92743) and ANXA1 (P04083, plus additional N-term His-tag). The searches for MDH (P00346) were performed with the custom database ACE_0793_SOI_plus_con.fasta (248 entries). This search database contains the sequences of MDH (P00346), trypsin (P00761) and already contains 246 known contaminants. The contaminant sequences were included in both searches to estimate the level of contamination. Andromeda searches allowed oxidation of methionine residues (16 Da) and acetylation of the protein N terminus (42 Da). No static modifications were set. Enzyme specificity was set to “unspecific”. The instrument type in Andromeda searches was set to Orbitrap and the precursor mass tolerance was set to ±20 ppm (first search) and ±4.5 ppm (main search). The MS/MS match tolerance was set to ±20 ppm. The peptide spectrum match false discovery rate and the protein false discovery rate were set to 0.01 (based on target-decoy approach). Minimum peptide length was 7 amino acids. For protein quantification, unique and razor peptides were allowed. In addition to unmodified peptides, modified peptides with dynamic modifications were allowed for quantification. The minimum score for modified peptides was set to 40.

### UMSAP

MS-data were evaluated with the targeted proteolysis module of UMSAP 2.2.1 (17). MS-data of the ANXA1 alone samples served as reference for all UMSAP calculations. The significance level was set to 0.05 and the minimum score value to 50. A log2 transformation was applied to the data before the analysis. The amino acid distribution around the cleavage sites included 5 residues in each direction. Chain A of the Protein Data Bank (PDB) file 1HM6 was used for mapping of the cleavage sites to the ANXA1 structure. The native and recombinant sequences used for this calculation can be found in Supporting Data 1. Individual samples that were below the cutoff of 0.4 in Pearson correlation analyses were excluded (17).

### Calculation of the relative frequency of cuts by UMSAP

The calculation of the frequency of cuts is performed in two steps. First, UMSAP groups all MS-detected peptides that share the same P1-P1′ bond. Subsequently, the relative frequency of cuts is calculated as follows. For each peptide and experiment the average intensities are calculated. Subsequently, for each peptide the average intensity ratios are calculated taking as reference the first average intensity greater than zero along the time points for each peptide. Finally, the relative frequency of cuts for a P1 site at a time point is calculated as the sum of the average intensity ratios of all peptides that share the same P1 site. If the peptide was not detected at a time point or the intensity values are not significantly different to the control experiments the average intensity is set to zero for this time point. A numeric example is provided in Supporting Data 5.

### CD spectroscopy

CD spectra were recorded on a Jasco J-170 CD spectrometer in a 1 mm quartz cuvette at 37 °C from 190-260 nm, averaging 5 scans. For the proteolysis of ANXA1, samples consisted of 200 μl with 5 μM ANXA1, 5 μM HTRA1 or 5 μM ANXA1 and 5 μM HTRA1 together in 150 mM NaPi, pH 8, 380 mM NaCl. For the proteolysis of MDH, 5 μM MDH, 0.01 μM trypsin or 5 μM MDH and 0.01 μM trypsin were mixed in 200 μl 50 mM Tris, pH 8. The secondary structure composition was analyzed using DichroWeb (http://dichroweb.cryst.bbk.ac.uk) using the CDSSTR algorithm (29, 30). Kinetic measurements were performed with either 5 μM ANXA1 and varying ratios of HTRA1 (ratio of HTRA1:ANXA1 in the range of 1:1–1:10) or 5 μM MDH and varying ratios of trypsin (ratio of MDH:trypsin in the range of 1:500–1:100,000) as well as each protein separately (5 μM for ANXA1, HTRA1, and MDH, respectively, and 0.01 μM for trypsin) as control. The CD signal was monitored at 222 nm, which is characteristic for α-helices. Measurements over the time frame of 5 h or 2 h were collected with a data pitch of 5 s and a response time of 1 s, while measurements over the course of 60 min were collected with a data pitch of 0.5 s and response time of 0.5 s. The data were normalized to represent the relative ellipticity at 222 nm and smoothed using GraphPad Prism software version 8.4.1 and 9.2.0 (www.graphpad.com).

## Data availability

All data are contained within the manuscript, except the mass spectrometry data for the digestions have been deposited to the ProteomeXchange Consortium *via* the PRIDE partner repository (https://www.ebi.ac.uk/pride/archive/) with the dataset identifier PXD031534 and PXD047437 (31).

## Supporting information

This article contains supporting information.

## Conflict of interest

The authors declare that they have no conflicts of interest with the contents of this article.
