# Supplementary material for: High resolution analysis of proteolytic substrate processing
Source: J Biol Chem. 2024 Sep 21;300(11):107812. doi: 10.1016/j.jbc.2024.107812 (PMC11513451; doi:10.1016/j.jbc.2024.107812)
Supplement: Supporting Information [file mmc1.pdf]

Supporting Information

Supporting Figures 1 - 9

Fig. S1. Histogram of relative cleavage efficiencies of ANXA1 digests.

Fig. S2. Relative amino acid distribution at sites P1-P5 and P1'-P5' of ANXA1 after various times of incubation.

Fig. S3. Consensus sequences derived from relative amino acid distribution at sites P1-P5 and P1'-P5' after various times of incubation.

Fig. S4. Proteolysis of folded ANXA1 at low HTRA1 concentrations.

Fig. S5. Circular dichroism spectroscopy of ANXA1 incubated with HTRA1.

Fig. S6. Histogram of relative cleavage efficiencies of MDH digests.

Fig. S7. Relative amino acid distribution at sites P1-P5 and P1'-P5' of MDH after various times of incubation.

Fig. S8. Proteolysis of folded MDH at 0.002 μM trypsin concentration.

Fig. S9. Circular dichroism spectroscopy of MDH incubated with trypsin.

Supporting Tables 1-6

Table S1
List of P1 residues of ANXA1 with the highest relative numbers of cuts

Table S2
List of P1 residues of ANXA1 with second highest relative numbers of cuts

Table S3
P1 sites that are cleaved more efficiently in denatured vs folded ANXA1

Table S4.
List of P1 residues of MDH with the highest relative numbers of cuts

Table S5.
List of P1 residues of MDH with the second highest relative numbers of cuts

Table S6.
P1 sites that are cleaved more efficiently in denatured vs folded ANXA1

Supporting Data 1-10

Supporting data 1
Sequences of native and recombinant ANXA1
Alignment of proteolytic products of denatured substrate to the primary amino acid sequence of ANXA1

Supporting data 2
Multiple sequence alignment of ANXA1 proteins

Supporting data 3
Alignment of proteolytic products of folded substrate to the primary amino acid sequence of ANXA1

Supporting data 4
Alignment of proteolytic products of folded ANXA1 obtained with 1, 2 and 4 μM HTRA1 to the primary amino acid sequence of ANXA1

Supporting data 5
Numeric example for how UMSAP calculates the relative frequency of cleavages

Supporting data 6
Sequences of native MDH
Alignment of proteolytic products of denatured substrate to the primary amino acid sequence of MDH

Supporting data 7
Multiple sequence alignment of MDH proteins

Supporting data 8
Alignment of proteolytic products of folded substrate to the primary amino acid sequence of MDH

Supporting data 9
Alignment of proteolytic products of folded MDH obtained with 2 nM trypsin to the primary amino acid sequence of MDH

Supporting data 10
Overview of MS samples

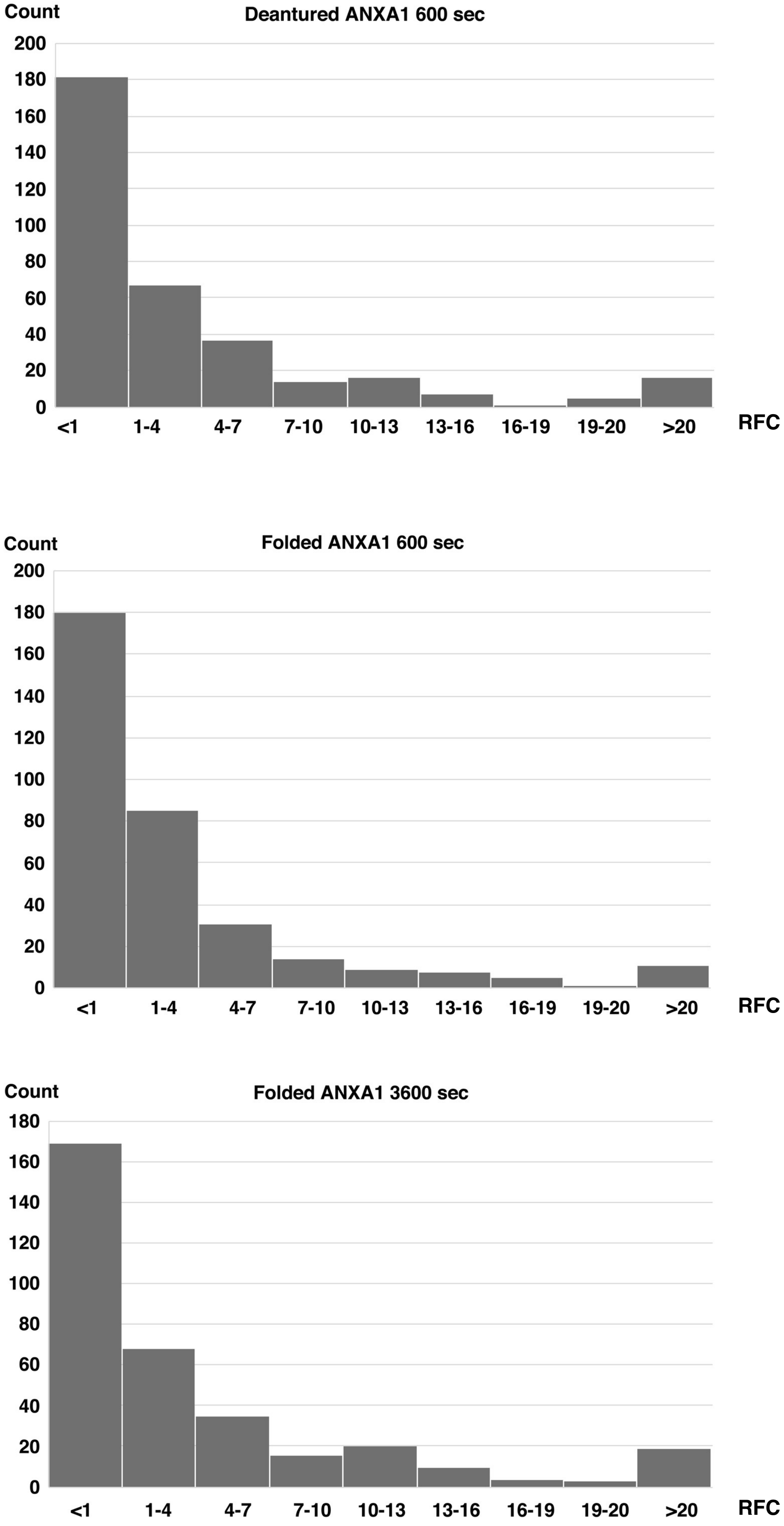

Fig. S1. Histogram of relative cleavage efficiencies in ANXA1 digests. To classify cleavage sites, the relative cleavage efficiencies after each residue was calculated by UMSAP. The histogram was generated by Xcel using width of interval: 3, underflow bin: 0.99; overflow bin: 20 as parameters. RFC, relative frequency of cuts.

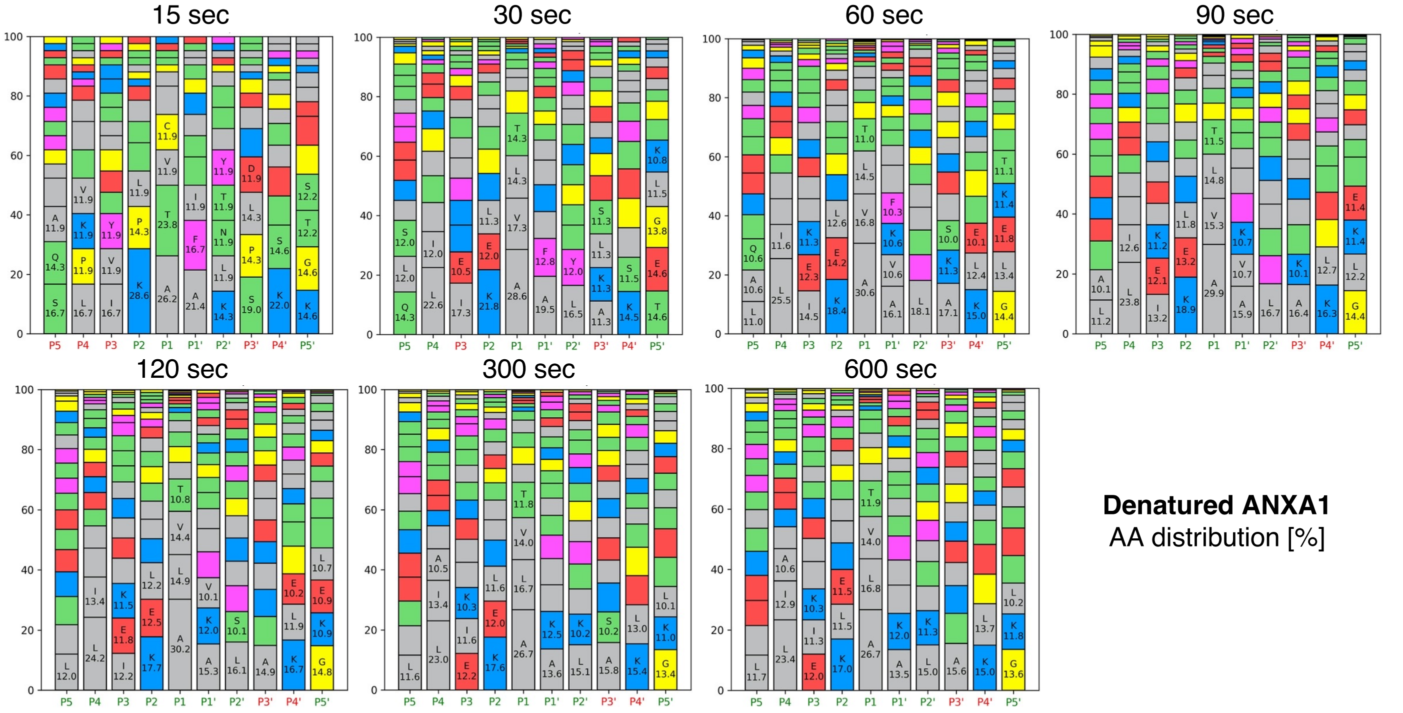

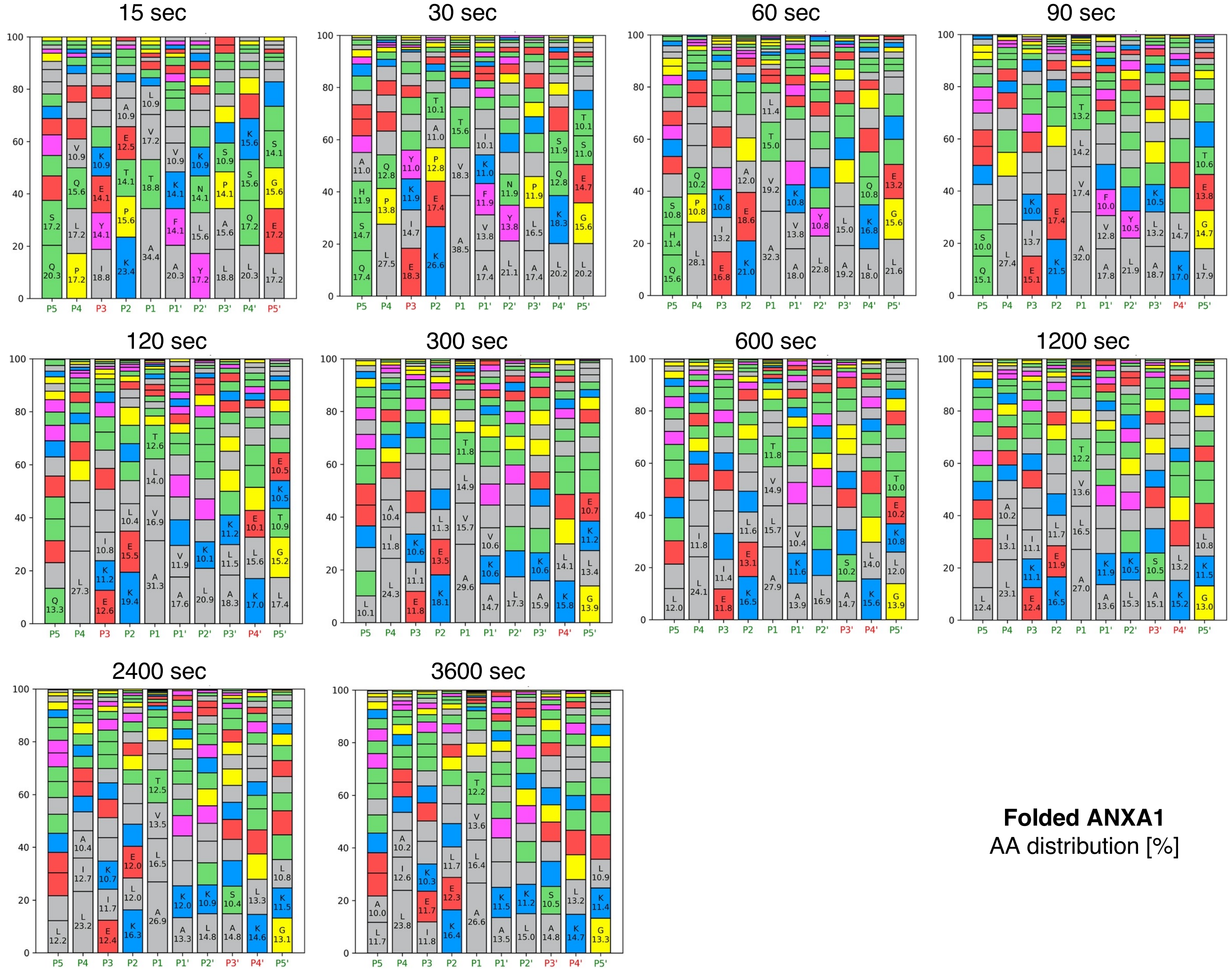

Fig. S2. Relative amino acid distribution at P1-P5 and P1'-P5' sites of ANXA1 after various times of incubation. X-axis, red letter, no protease selectivity toward specific amino acids at this position; green letter, protease selectivity toward certain amino acids at this position.

Denatured ANXA1

P5	P4	P3	P2	P1	P1'	P2'	P3'	P4'	P5'
			K 29<br>P 14<br>L 12	A 26<br>T 24<br>V 12<br>C 12	A 21<br>F 17<br>I 12	K 14<br>L 12<br>N 12<br>T 12<br>Y 12			K 15<br>G 15<br>T 12<br>S 12
Q 14<br>L 12<br>S 12	L 23<br>I 12		K 22<br>E 12<br>L 11	A 29<br>V 17<br>L 14<br>T 14	A 19<br>F 13	L 17<br>Y 12			T 15<br>E 15<br>G 14<br>L 12<br>K 11
L 11<br>A 11<br>Q 11	L 26<br>I 12	I 15<br>E 12<br>K 11	K 18<br>E 14<br>L 13	A 31<br>V 17<br>L 15<br>T 11	A 16<br>V 11<br>K 11<br>F 10	L 18			G 14<br>L 13<br>E 12<br>K 11<br>T 11
L 11<br>A 10	L 24<br>I 13	I 13<br>E 12<br>K 11	K 19<br>E 13<br>L 12	A 30<br>L 15<br>V 15<br>T 12	A 16<br>V 11<br>K 11	L 17			G 14<br>L 12<br>K 11<br>E 11
L 12	L 24<br>I 13	I 12<br>E 12<br>K 12	K 18<br>E 13<br>L 12	A 30<br>L 15<br>V 15<br>T 11	A 15<br>K 12<br>V 10	L 16<br>S 10			G 15<br>K 11<br>E 11<br>L 11
L 12	L 23<br>I 13<br>A 11	E 12<br>I 12<br>K 10	K 18<br>E 12<br>L 12	A 27<br>L 17<br>V 14<br>T 12	A 14<br>K 13	L 15<br>K 10			G 13<br>K 11<br>L 10
L 12	L 23<br>I 13<br>A 11	E 12<br>I 11<br>K 10	K 18<br>E 12<br>L 12	A 27<br>L 17<br>V 14<br>T 12	A 14<br>K 12	L 15<br>K 11			G 14<br>K 12<br>L 10
L	L,I,A	E,I,K	K,E,L	A,L,V,T	A,K	L,K			G,K,L

Folded ANXA1

Time sec	P5	P4	P3	P2	P1	P1'	P2'	P3'	P4'	P5'
15	Q 20<br>S 17	P 17<br>L 17<br>Q 16<br>V 11		K 24<br>P 16<br>T 14<br>E 13<br>A 11	A 34<br>T 19<br>V 17<br>L 11	A 20<br>F 14<br>K 14<br>V 11	Y 17<br>L 16<br>N 14<br>K 11	L 19<br>A 16<br>P 11<br>S 11	L 20<br>Q 17<br>S 16<br>K 16	L 17<br>E 17<br>G 16<br>S 14
30	Q 17<br>S 15<br>H 12<br>A 11	L 28<br>P 14<br>Q 13		K 27<br>E 17<br>P 13<br>A 11<br>T 10	A 39<br>V 18<br>T 16	A 17<br>V 14<br>F 12<br>K 11<br>I 10	L 21<br>Y 14<br>N 12	A 17<br>L 17<br>P 12	L 20<br>K 18<br>Q 13<br>S 12	L 20<br>G 16<br>E 15<br>S 11<br>T 10
60	Q 16<br>H 11<br>S 11	L 28<br>P 11<br>Q 10	E 17<br>I 13<br>K 11	K 21<br>E 19<br>A 12	A 32<br>V 19<br>T 15<br>L 11	A 18<br>V 14<br>K 11	L 23<br>Y 11	A 19<br>L 15	L 18<br>K 17<br>Q 11	L 22<br>G 16<br>E 13
90	Q 15<br>S 10	L 27	E 15<br>I 14<br>K 10	K 22<br>E 17	A 32<br>V 17<br>L 14<br>T 13	A 18<br>V 13<br>F 10	L 22<br>Y 11	A 19<br>L 13<br>K 11		L 18<br>G 15<br>E 14<br>T 11
120	Q 13	L 27		K 19<br>E 16<br>L 10	A 31<br>V 17<br>L 14<br>T 13	A 18<br>V 12	L 21<br>K 10	A 18<br>L 12<br>K 11		L 17<br>G 15<br>T 11<br>K 11<br>E 11
300	L 10	L 24<br>I 12<br>A 10	E 12<br>I 11<br>K 11	K 18<br>E 14<br>L 11	A 30<br>V 16<br>L 15<br>T 12	A 15<br>K 11<br>V 11	L 17	A 16<br>K 11		G 14<br>L 13<br>K 11<br>E 11
600	L 12	L 24<br>I 12	E 12<br>I 11	K 17<br>E 13<br>L 12	A 28<br>L 16<br>V 15<br>T 12	A 14<br>K 12<br>V 10	L 17			G 14<br>L 12<br>K 11<br>E 11<br>T 10
1200	L 12	L 23<br>I 13<br>A 10	E 12<br>K 11<br>I 11	K 17<br>E 12<br>L 12	A 27<br>L 17<br>V 14<br>T 12	A 14<br>K 12	L 15<br>K 11			G 13<br>K 12<br>L 11
2400	L 12	L 23<br>I 13<br>A 10	E 12<br>I 12<br>K 11	K 16<br>E 12<br>L 12	A 27<br>L 17<br>V 14<br>T 13	A 13<br>K 12	L 15<br>K 11			G 13<br>K 12<br>L 11
3600	L 12<br>A 10	L 24<br>I 17<br>A 10	I 12<br>E 12<br>K 10	K 16<br>E 12<br>L 12	A 27<br>L 16<br>V 14<br>T 12	A 14<br>K 12	L 15<br>K 11			G 13<br>K 11<br>L 11
	L	L,I,A	E,I,K	K,E,L	A,L,V,T	A,K	L,K			G,K,L

Fig. S3. Consensus sequences derived from relative amino acid distribution at P1-P5 and P1'-P5' sites after various times of incubation. Left, denatured ANXA1; right, folded ANXA1 were used as substrates. Numbers were taken from SI Fig. 2 and represent % of occurrence of the residue indicated at the given position. Only residues found at >10% are shown. Red letters indicate that consensus sequences given at the bottom of a cell are reached. Empty fields, no protease selectivity toward specific amino acids at this position.

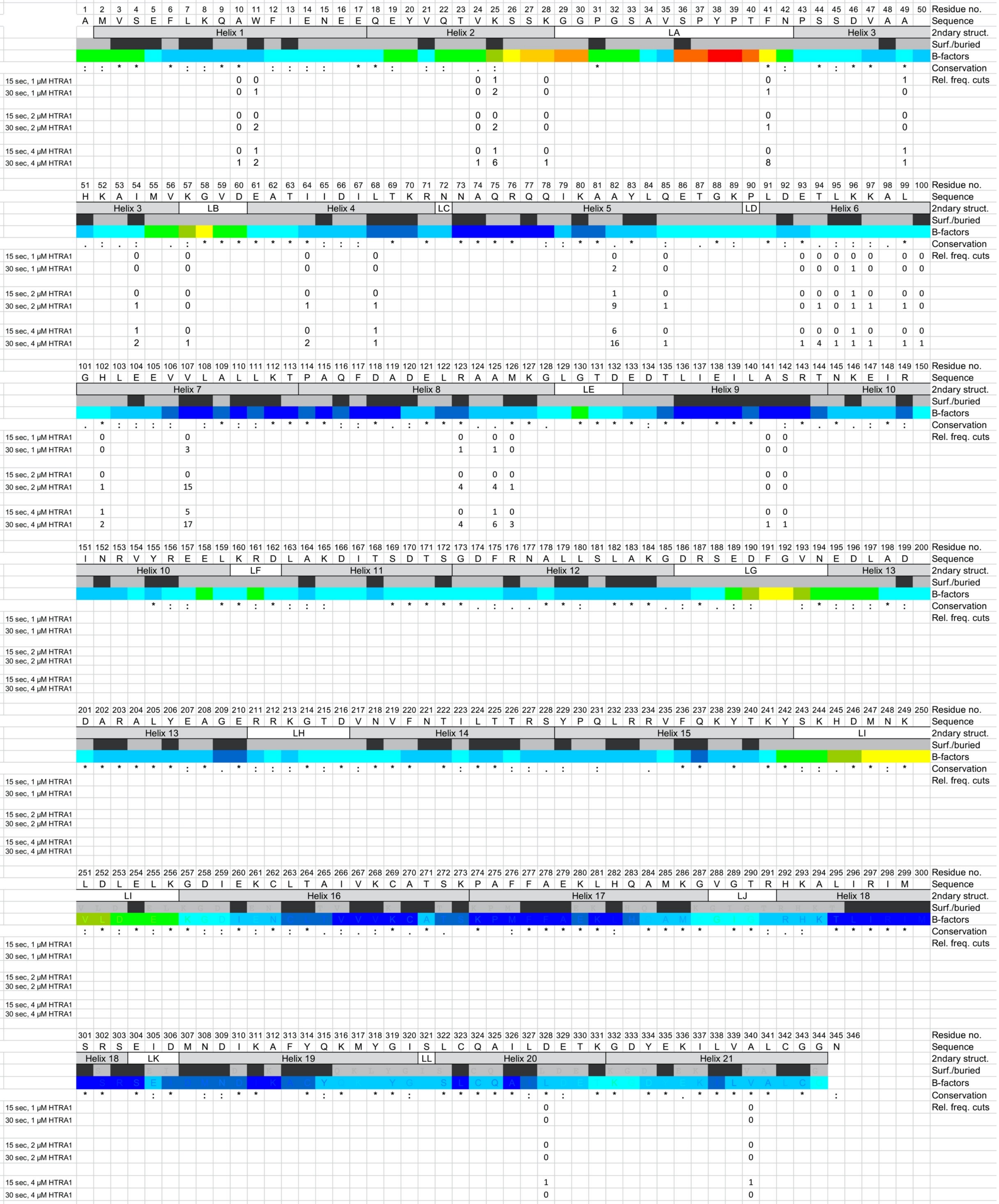

Fig. S4. Proteolysis of folded ANXA1 at low HTRA1 concentrations. 20 µM ANXA1 was incubated with 1, 2 or 4 µM HTRA1 at 37°C. Samples were taken for MS analysis at the timepoint indicated. The identified peptides aligned to the primary amino acid sequence of ANXA1 are provided in Supporting data 4. P1 residues identified in n=4 independent experiments significantly enriched compared to controls are shown. Structural elements i.e. helices and loops (2ndary struct.), B-factors and surface accessibility (surf. (grey)/buried (black) as detected in the crystal structure of folded ANXA1 (pdb:1HM6) are indicated. The colour code for B-factors indicates gradually structural rigidity (blue) to flexibility (red). Amino acid sequence conservation (Conservation) is derived from a multiple sequence alignment (Supporting data 2); * = identical, : = conserved residues. Numbers of ANXA1 below individual P1 positions indicate the relative frequency of cuts (Rel. freq. cuts) at each timepoint. No number indicates that no cleavage was detected at any of the timepoints investigated.

A

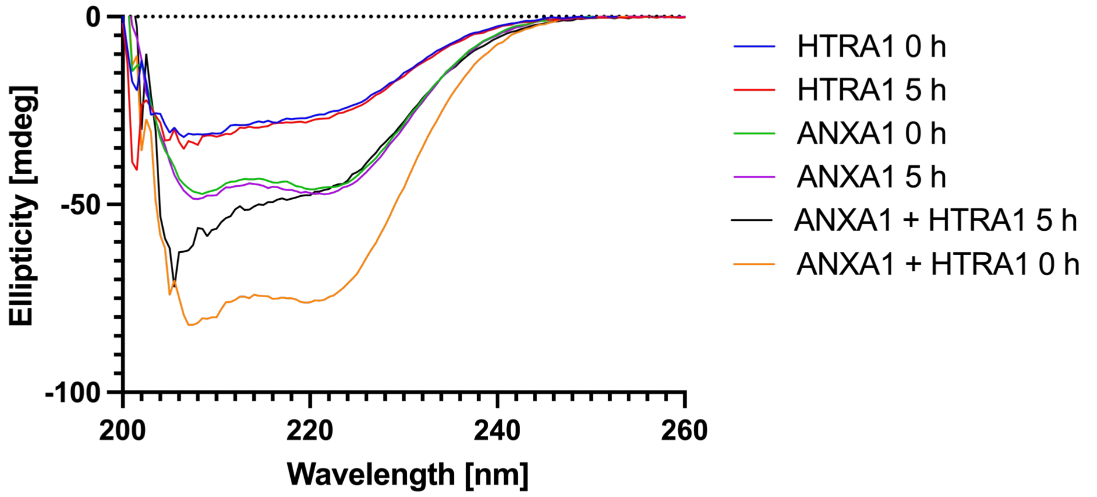

B

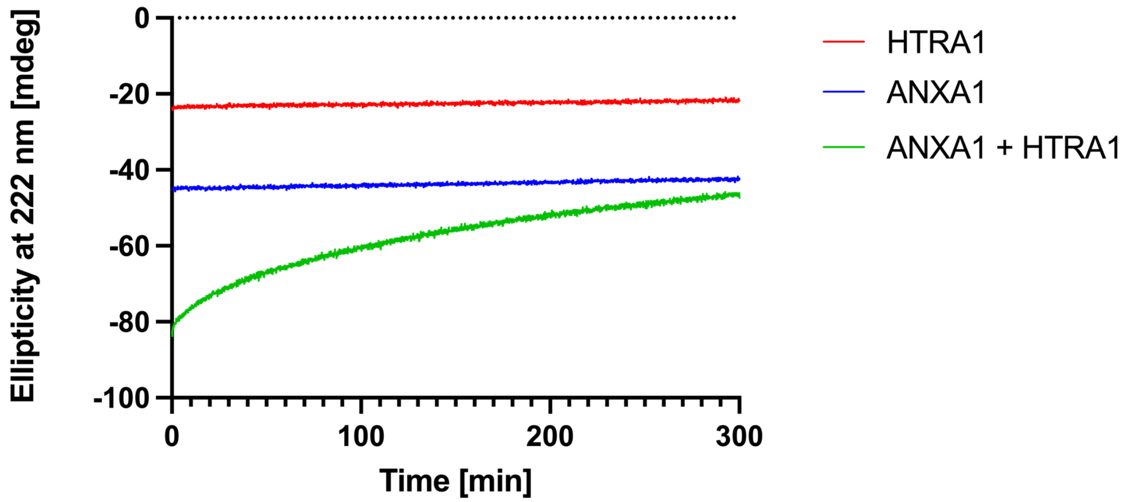

Fig. S5. Circular dichroism spectroscopy of ANXA1 incubated with HTRA1. 5 μM ANXA1, 5 μM HTRA1 or a mix of 5 μM ANXA1 and 5 μM HTRA1 were subjected to CD spectroscopy A. CD spectra of HTRA1, ANXA1 or a mix of the two proteins at the times indicated. The large loss of the α-helical minimum at 222 nm when ANXA1 has been proteolytically digested for 5 hours by HTRA1 can be observed. For each spectrum, the photomultiplier HT voltage was less than 600 V throughout the scan. B. Time course showing the gain in ellipticity at 222 nm over 5 hours as ANXA1 is proteolytically digested after mixing with HTRA1 (*green*). ANXA1 alone (*blue*) or HTRA1 alone (*red*) control experiments are also shown and show no change.

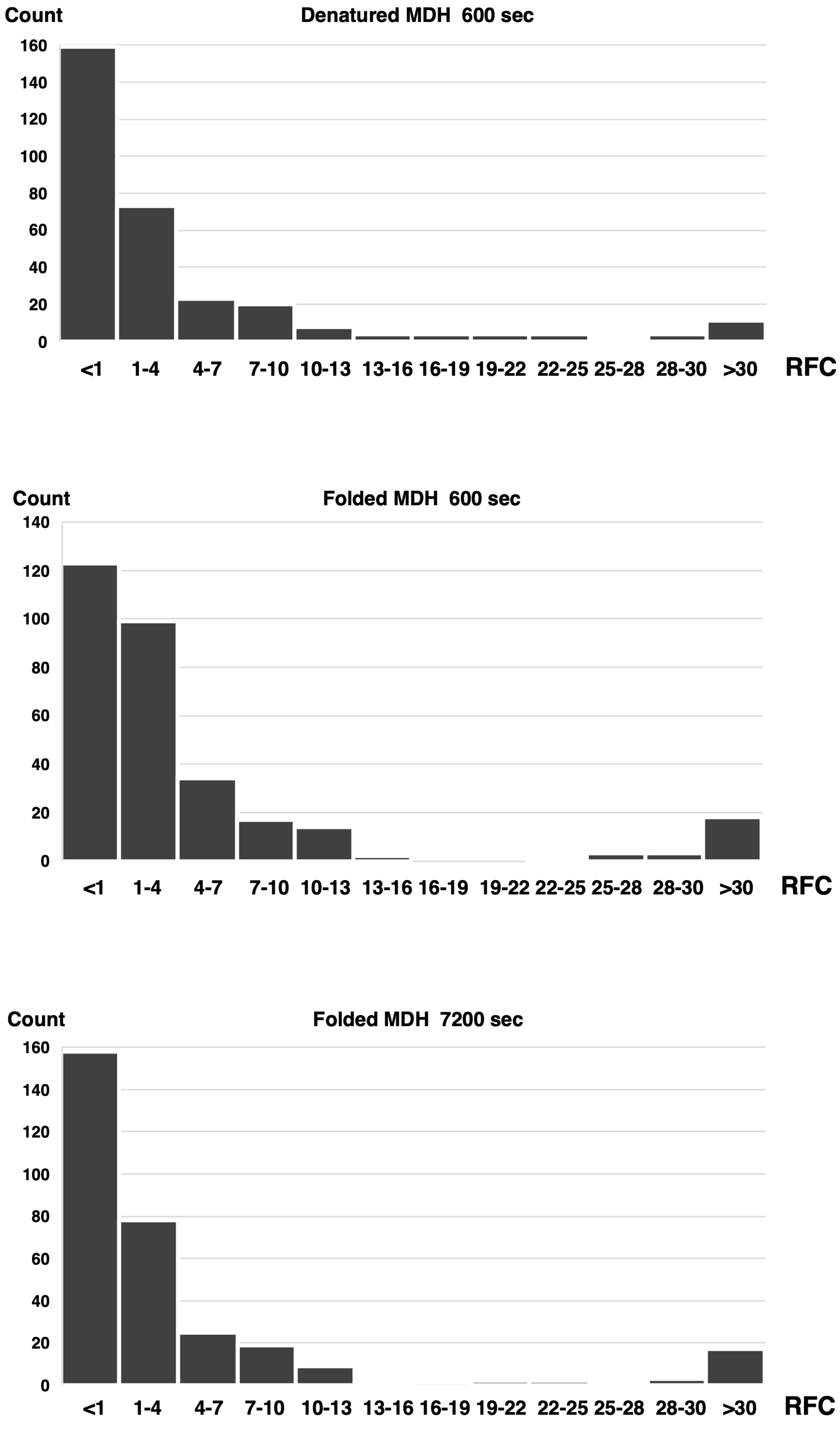

Fig. S6. Histogram of relative cleavage efficiencies in MDH digests. To classify cleavage sites, the relative cleavage efficiencies after each residue was calculated by UMSAP. The histogram was generated by Xcel using width of interval: 3, underflow bin: 0.99; overflow bin: 30 as parameters. RFC, relative frequency of cuts.

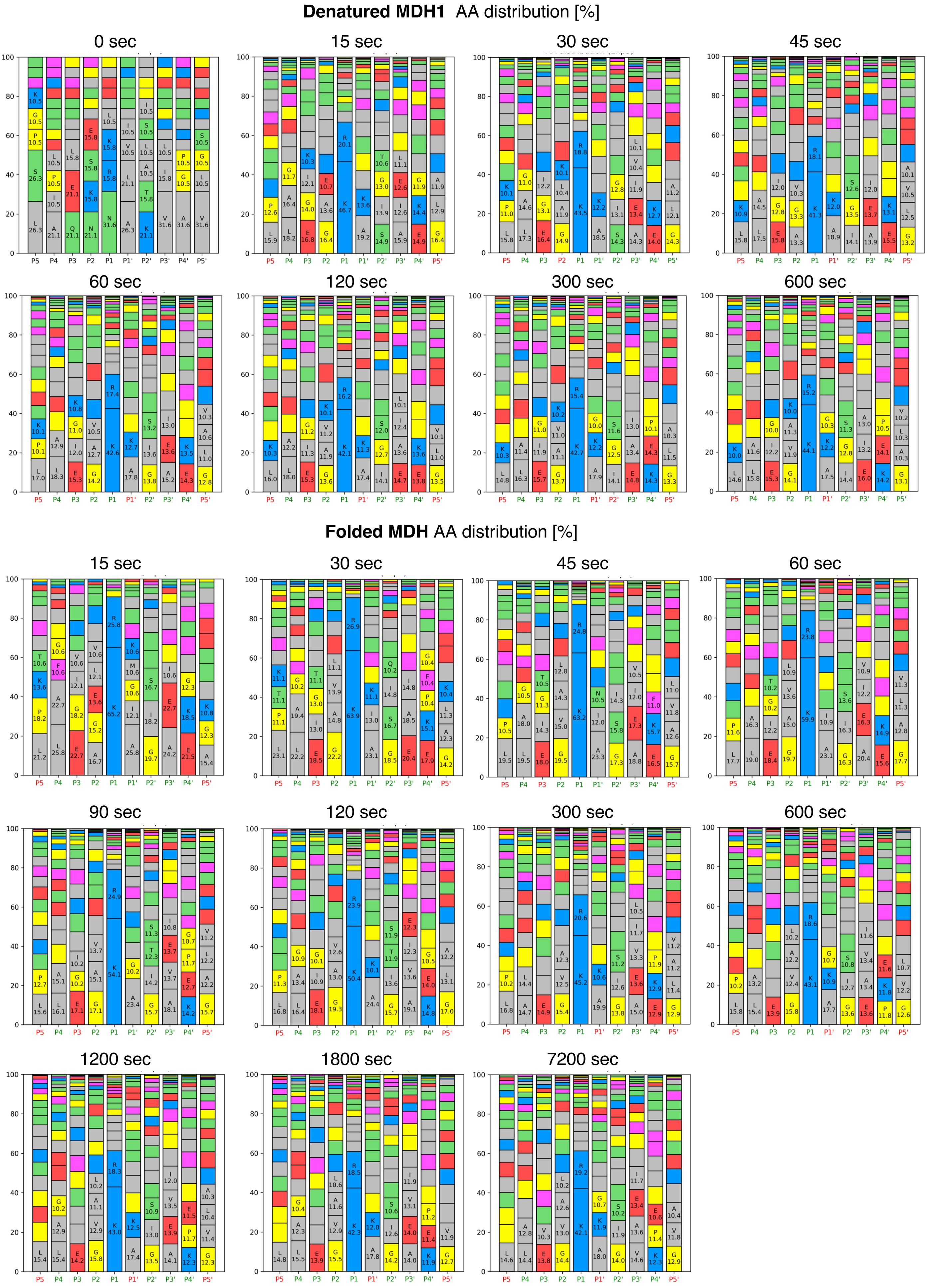

Fig. S7. Relative amino acid distribution at P1-P5 and P1'-P5' sites of MDH after various times of incubation. X-axis, red letter, no protease selectivity toward specific amino acids at this position; green letter, protease selectivity toward certain amino acids at this position.

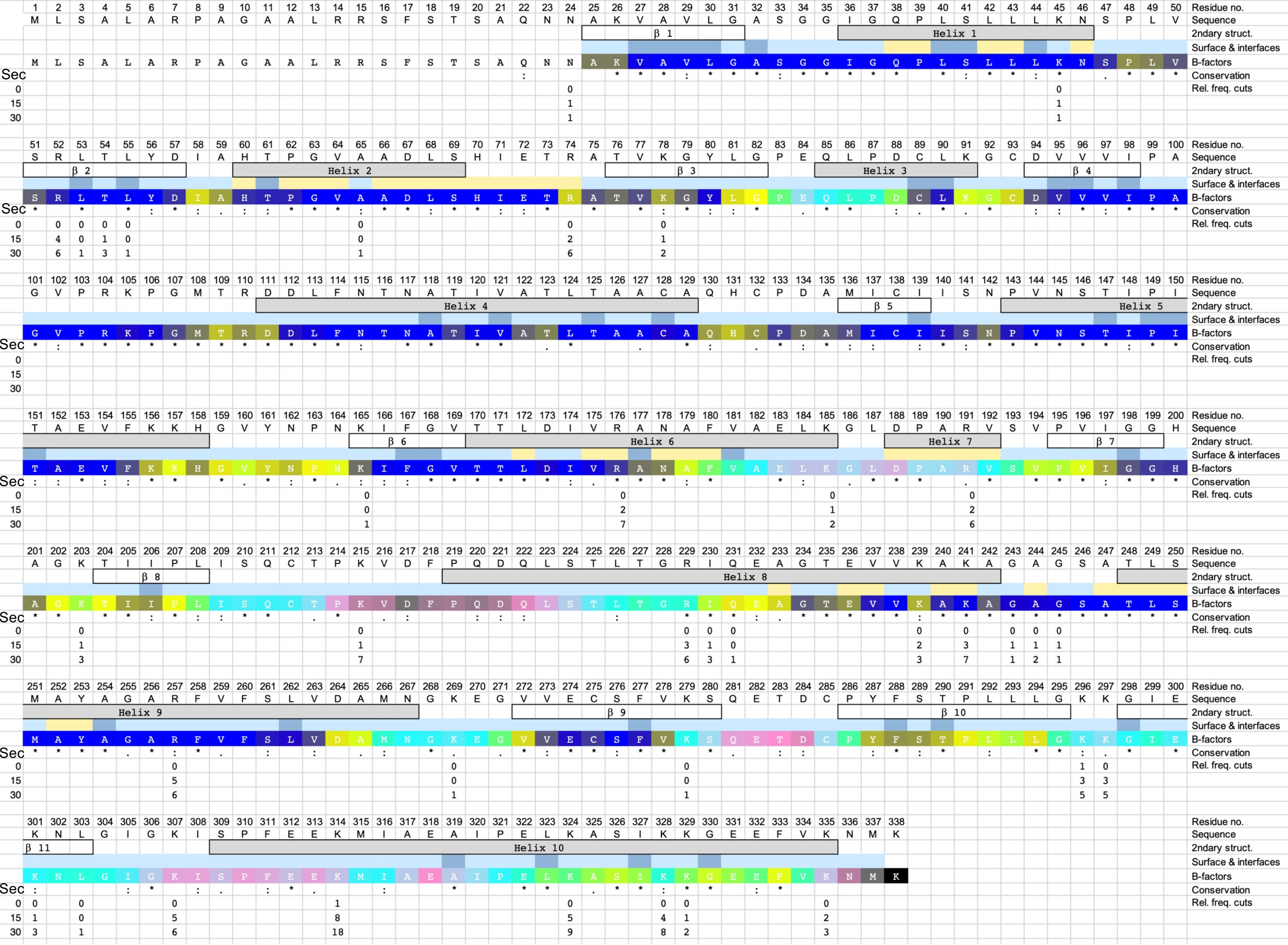

Fig. S8. Proteolysis of folded MDH by 2 nM trypsin. 20 μM folded MDH was incubated with 2 nM trypsin. Samples were taken for MS analysis at the timepoints indicated (Sec). The identified peptides aligned to the primary amino acid sequence of ANXA1 are provided in Supporting data 9. P1 residues identified in n=4 experiments as significantly enriched compared to controls are represented by numbers. Structural elements i.e. helices and loops (2ndary struct.). Surface accessibility (solvent accessible (light blue), solvent inaccessible (dark blue), and interfacing residues (yellow) as detected in the crystal structure of folded MDH (pdb:1MLD) are indicated. The colour code for B-factors indicates gradually structural rigidity (blue) to flexibility (lighter colors). Amino acid sequence conservation (Conservation) is derived from a multiple sequence alignment (Supporting data 7); * = identical, : = conserved residues. Numbers below individual P1 positions indicate the relative frequency of cuts (Rel. freq. cuts) at each timepoint. No number indicates that no cleavage was detected at any of the timepoints investigated.

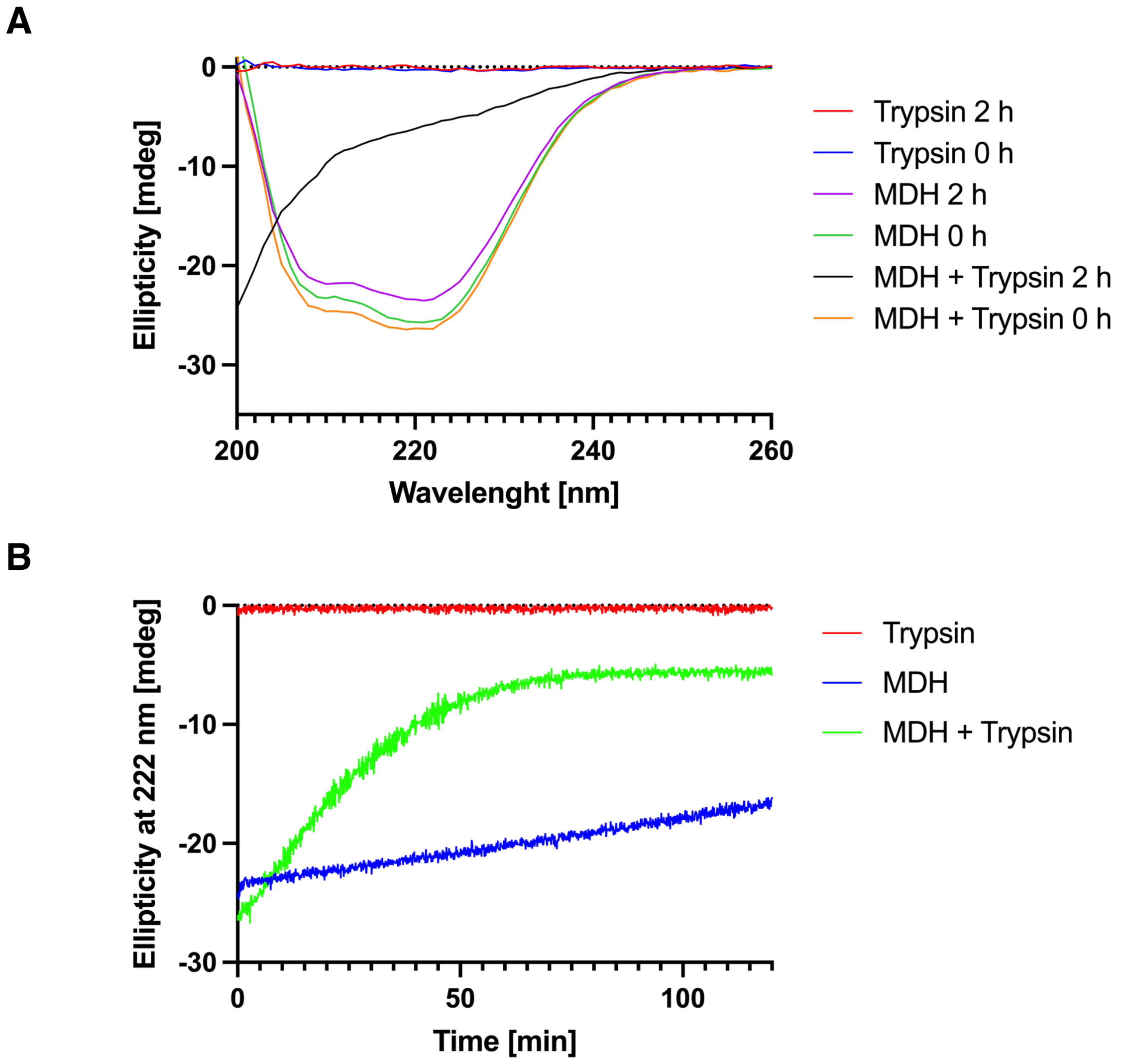

Fig. S9. CD spectroscopy of MDH incubated with trypsin. 5 μM MDH or 0.01 μM trypsin or a mix of 5 μM MDH and 0.01 μM trypsin were subjected to CD spectroscopy. A. CD spectra at the times indicated. The large loss of the α-helical minimum at 222 nm when MDH was proteolytically digested by trypsin can be observed. For each spectrum, the photomultiplier HT voltage was less than 600 V throughout the scan. B. A time course showing the gain in ellipticity at 222 nm over 2 hours as MDH is proteolytically digested after mixing with trypsin (*green*). MDH alone (*blue*) or trypsin alone (*red*) control experiments are also shown and show less or no change, respectively.

Table S1. List of P1 residues of ANXA1 with the highest relative numbers of cuts

Residue	Denatured 600 sec	Folded 600 sec	Folded 3600 sec
Q 10			x
A 11	x		x
V 25	x	x	x
T 41	x	x	x
A 54	x	x	x
L 69	x		x
A 82	x	x	x
A 83			x
V107	x	x	x
L123	x	x	x
A125	x	x	x
A126	x		x
L181	x		x
V236			x
C270		x	x
A276	x		x
L282	x		x
M300	x	x	x
A313			x
Q316	x		x
C324	x		x
A326	x	x	x

Class I P1 residues (> 20 cuts) detected in proteolysis experiments with denatured and folded ANXA1 at the timepoints indicated. x = detected

Table S2. List of P1 residues of ANXA1 with the second highest relative numbers of cuts

Residue	Denatured 600 sec	Folded 600 sec	Folded 3600 sec
A 2			x
S 5			x
Q 10		x	
T 24	x	x	x
S 27		x	x
M 56			x
V 57	x		
L 69			
T 70			x
N 73		x	x
A 83	x	x	
L 85	x	x	x
T 88		x	x
T 95	x	x	
A 99			x
L109	x		x
L112		x	x
A116			x
A126		x	
M127			x
I140			x
L141	x		x
T145	x		x
L160	x		x
T169	x		
L181		x	x
A184	x		
A205	x		
T223	x		
V236	x	x	
T241			x
L254			x
C263	x		
T265			x
C270	x		
A271	x		
A276			
V289	x		x
I299	x		x
V301	x		x
S302	x		x
A313	x	x	x
M318	x		
C324		x	x
A326	x		x
V340		x	x
C343			x

Class II P1 residues (11 - 20 cuts) detected in proteolysis experiments with denatured and folded ANXA1 at the timepoints indicated. x = detected

Table S3: P1 sites that are cleaved more efficiently in denatured vs folded ANXA1

Res	0	15	30	60	90	120	300	600	1200	2400	3600
L69	0	0	0	2	4	8	16	21			
L69	0	0	0	1	3	4	11	15	22	24	27
T70	0	0	0	1	1	2	5	11			
T70	0	0	0	1	3	4	5	5	6	9	12
N73	0	0	0	2	6	7	10	5			
N73	0	0	1	1	1	5	9	12	13	11	14
T101	0	0	0	1	1	4	9	9			
T101	0	0	0	1	1	2	4	4	4	4	4
I140	1	1	2	7	6	8	9	10			
I140	0	0	0	1	3	3	5	8	10	13	10
L141	0	0	1	3	6	8	16	19			
L141	0	0	0	0	1	2	5	9	13	15	15
I152	0	0	0	0	1	3	6	4			
I152	0	0	0	0	0	0	0	2	2	3	2
V155	0	0	0	0	0	0	3	4			
V155	0	0	0	0	0	0	0	1	3	3	5
L160	0	0	1	4	3	7	12	14			
L160	0	0	0	0	0	0	3	6	8	8	11
L164	0	1	1	1	2	2	2	3			
L164	0	0	0	0	0	0	0	0	0	0	1
A165	0	0	0	1	2	1	1	1			
A165	0	0	0	0	0	0	1	1	1	1	1
I168	0	0	0	1	1	2	3	2			
I168	0	0	0	0	0	0	2	4	4	4	3
T169	0	0	0	2	2	7	9	12			
T169	0	0	0	0	0	1	3	6	8	9	9
T172	0	0	0	1	0	2	5	3			
T172	0	0	0	0	0	0	1	2	5	5	7

A179	0	0	0	0	0	0	2	4			
A179	0	0	0	0	0	0	0	2	2	2	3
L181	0	1	3	11	14	19	24	27			
L181	0	0	0	2	5	7	12	15	17	18	18
S182	0	0	0	0	0	0	1	3			
S182	0	0	0	0	0	0	0	0	0	1	2
A184	0	0	0	2	1	7	10	7			
A184	0	0	0	0	0	0	3	7	9	8	8
A205	0	0	2	8	6	9	11	13			
A205	0	0	0	0	1	2	4	6	7	8	10
A209	0	0	1	2	4	6	5	7			
A209	0	0	0	0	0	0	1	2	3	6	7
V218	0	0	1	4	3	4	4	1			
V218	0	0	0	0	0	0	1	2	3	4	4
N219	0	0	1	1	1	1	1	1			
N219	0	0	0	0	0	1	2	2	3	3	3
T223	0	1	2	6	6	8	9	11			
T223	0	0	0	1	2	2	4	5	7	9	10
T226	0	0	2	3	4	7	6	2			
T226	0	0	0	0	1	1	3	6	7	7	8
V236	0	1	6	12	13	15	17	15			
V236	0	0	0	1	5	6	11	15	18	18	21
Q238	0	0	0	0	1	5	7	5			
Q238	0	0	0	0	0	0	0	1	2	1	3
T241	0	0	0	0	1	1	4	6			
T241	0	0	0	0	0	0	0	1	4	10	13
S244	0	0	0	0	0	0	1	2			
S244	0	0	0	0	0	0	0	0	1	1	2
V251	0	0	1	2	3	3	4	4			
V251	0	0	0	0	0	1	2	2	2	2	2

L252	0	0	0	1	1	1	2	3			
L252	0	0	0	0	0	0	1	1	2	2	3
L254	0	0	1	1	1	1	4	9			
L254	0	0	0	0	1	1	1	3	5	8	11
L256	0	0	0	1	1	1	3	5			
L256	0	0	0	0	1	0	0	2	5	3	7
C263	0	0	1	2	1	4	10	20			
C263	0	0	0	0	0	0	1	1	6	6	9
T265	0	0	0	0	1	4	6	8			
T265	0	0	0	0	0	0	0	0	3	7	11
I267	0	0	0	0	0	0	1	3			
I267	0	0	0	0	0	0	0	0	1	1	5
V268	0	0	0	0	0	0	1	1			
V268	0	0	0	0	0	0	0	0	1	0	5
C270	0	3	6	9	10	11	14	19			
C270	0	0	1	4	5	8	15	22	26	25	33
A271	0	1	2	3	3	7	9	12			
A271	0	0	0	1	2	2	5	4	5	6	6
A276	0	0	0	5	11	16	21	25			
A276	0	0	0	0	1	5	11	14	20	23	28
L282	0	2	3	7	10	13	20	25			
L282	0	0	1	4	7	11	15	18	24	25	36
Q284	0	1	1	3	4	5	8	8			
Q284	0	0	0	0	1	1	3	4	5	5	5
A285	0	1	1	3	4	6	8	10			
A285	0	0	0	0	2	6	8	8	10	12	19
K287	0	0	0	0	0	1	3	5			
K287	0	0	0	0	0	0	0	1	1	2	2
V289	0	1	2	6	10	11	11	11			

V289	0	0	0	0	0	2	9	10	11	11	12
T291	0	0	0	1	1	1	1	1			
T291	0	0	0	0	0	0	1	4	5	5	6
A295	0	0	0	0	1	1	1	2			
A295	0	0	0	0	0	0	1	2	2	2	5
L296	0	0	0	0	1	2	5	5			
L296	0	0	0	0	0	0	1	2	4	4	3
I299	0	0	0	4	7	9	13	15			
I299	0	0	0	0	0	0	2	5	10	9	12
M300	0	0	4	9	19	27	31	33			
M300	0	0	0	0	3	5	20	26	31	31	35
V301	1	1	1	3	7	8	12	19			
V301	0	1	0	0	0	0	3	4	8	11	17
S302	0	0	0	0	3	7	11	12			
S302	0	0	0	0	0	0	1	3	5	7	11
I311	0	0	0	0	1	2	3	3			
I311	0	0	0	0	0	0	1	2	4	4	4
A313	1	1	2	5	9	12	18	20			
A313	0	0	1	0	2	2	12	16	20	22	24
Q316	0	0	1	3	9	13	18	24			
Q316	0	0	0	0	0	0	3	5	7	8	9
M318	0	0	1	1	2	4	6	11			
M318	0	0	0	0	1	0	4	6	9	9	11
I321	0	0	0	0	2	5	6	6			
I321	0	0	0	0	0	0	2	3	3	3	3
C324	0	1	3	8	8	15	19	25			
C324	0	1	0	1	2	4	11	14	20	23	24
A326	0	2	6	11	14	16	16	22			
A326	0	0	1	1	7	9	18	22	27	26	30

I327	0	1	2	4	5	6	7	8			
I327	0	0	0	0	0	1	6	5	7	8	8
L328	0	0	1	2	4	3	4	4			
L328	0	0	0	0	0	0	0	1	1	1	1
T331	0	0	1	1	1	3	4	5			
T331	0	0	0	0	0	0	2	3	5	5	6
V340	0	0	3	7	10	9	11	11			
V340	0	0	0	2	6	7	9	11	11	12	12
A341	0	0	1	3	4	4	4	5			
A341	0	0	0	0	0	1	2	4	5	5	5
L342	0	0	1	3	4	4	3	4			
L342	0	0	0	0	0	0	1	1	1	1	1
C343	0	1	3	5	5	8	9	10			
C343	0	0	0	0	1	3	6	6	9	10	11

Left column = P1 residues. Numbers in top row represent seconds (sec) of incubation of ANXA1 with HTRA1; Numbers in all other lanes below represent the relative numbers of cuts at each P1 residue. For each P1 residue shown, the top and bottom lanes represent data obtained by digesting denatured or folded HTRA1, respectively. Res = amino acid residue and amino acid number in ANXA1.

Table S4. List of P1 residues of MDH with the highest relative numbers of cuts

Residue	Denatured 600 sec	Folded 600 sec	Folded 3600 sec
K 45	x		
R 52	x	x	
R 74	x	x	x
K 91			x
R176		x	x
K185		x	x
R191			x
K203	x	x	x
R229	x	x	x
K241		x	x
R257		x	x
K279		x	x
K296	x	x	x
K297	x	x	x
K301	x		
K307	x	x	x
K314	x	x	x
K324	x	x	x
K328	x	x	x
K335	x	x	x

Class I P1 residues (> 30 cuts) detected in proteolysis experiments with denatured and folded MDH at the timepoints indicated. x = detected

Table S5. List of P1 residues of MDH with the second highest relative numbers of cuts

Residue	Denatured 600 sec	Folded 600 sec	Folded 3600 sec
K 45		x	x
R 52			x
K 78		x	x
R104			x
K157		x	x
R176	x		
R191	x	x	
K215	x	x	x
K239	x	x	x
K241	x		
R257	x		
K269			x
A317	x		
K329	x		

Class II P1 residues (21 - 30 cuts) detected in proteolysis experiments with denatured and folded ANXA1 at the timepoints indicated. x = detected

Table S6: P1 sites that are cleaved more efficiently in denatured vs folded ANXA1

Res	15	30	45	60	120	300	600	1200	1800	7200
K45	6	15	27	28	40	37	30			
K45	0	2	3	7	9	16	23	21	26	21
R52	11	28	36	36	55	58	48			
R52	0	4	6	6	14	28	32	25	26	24
R74	10	28	46	42	56	70	62			
R74	0	2	5	7	37	59	73	75	79	79
K91	1	3	4	5	5	9	12			
K91	0	0	0	0	2	11	18	19	26	33
R191	7	8	11	14	17	21	22			
R191	0	0	2	5	10	20	26	33	34	32
K203	11	16	18	20	29	46	50			
K203	0	0	4	7	24	42	56	65	66	65
K215	3	5	9	9	17	22	24			
K215	0	3	6	8	13	21	28	29	30	28
R229	7	11	13	12	24	32	34			
R229	0	4	8	10	17	23	34	39	42	38
K239	5	8	11	13	20	25	25			
K239	2	3	4	6	11	18	22	24	26	25
K241	7	11	15	20	26	31	28			
K241	0	4	9	11	22	30	33	30	32	37
R257	12	23	29	27	28	31	29			
R257	0	5	8	13	22	33	36	36	31	32
K269	0	2	2	3	6	15	18			
K269	0	0	1	1	3	8	13	26	26	22
K296	5	9	17	16	32	39	30			
K296	0	2	6	10	18	32	41	44	45	40
K297	6	13	19	21	36	49	46			
K297	0	5	8	13	17	27	36	41	39	35

K301	10	22	31	30	48	57	53			
K301	0	2	3	4	5	13	14	12	13	8
K307	10	23	34	34	49	53	52			
K307	0	4	8	13	23	40	46	47	48	37
K314	17	29	38	39	57	65	57			
K314	0	7	9	10	21	34	43	45	47	33
K328	9	18	32	36	53	62	64			
K328	0	5	9	13	18	33	42	44	44	40
K335	6	9	17	19	34	56	53			
K335	1	3	8	10	16	30	39	41	38	38

Left column = P1 residues. Numbers in top row represent seconds (sec) of incubation of MDH with trypsin; Numbers in all other lanes represent the relative numbers of cuts at each P1 residue. For each P1 residue shown, the top and bottom lanes represent data obtained by digesting denatured or folded MDH, respectively. Res = amino acid residue and amino acid number in MDH.

Supporting data 1

Native ANXA1 sequence

MAMVSEFLKQAWFIENEEQEYVQTVKSSKGGPGSAVSPYPTFNPSSDVAALHKAIMVKGVDEATIIDILTKRNNA
QRQQIKAAYLQETGKPLDETLKKALTGHLEEVVLALLKTPAQFDADELRAAMKGLGTDEDTLIEILASRTNKEIR
DINRVYREELKRDLAKDITSDTSGDFRNALLSLAKGDRSEDFGVNEDLADSDARALYEAGERRKGTDVNVFNTIL
TTRSYPQLRRVFQKYTKYSKHDMNKVLDLELKGDIEKCLTAIVKCATSKPAFFAEKLHQAMKGVGTRHKALIRIM
VSRSEIDMNDIKAFYQKMYGISLCQAILDETKGDYEKILVALCGGN

Denatured ANXA1 0 sec

```
1        10        20        30        40        50
MAMVSEFLKQAWFIENEEQEYVQTVKSSKGGPGSAVSPYPTFNPSSDVAA

51       60        70        80        90        100
LHKAIMVKGVDEATIIDILTKRNNAQRQQIKAAYLQETGKPLDETLKKAL

101      110       120       130       140       150
TGHLEEVVLALLKTPAQFDADELRAAMKGLGTDEDTLIEILASRTNKEIR
                          MKGLGTDEDTLIEI

151      160       170       180       190       200
DINRVYREELKRDLAKDITSDTSGDFRNALLSLAKGDRSEDFGVNEDLAD

201      210       220       230       240       250
SDARALYEAGERRKGTDVNVFNTILTTRSYPQLRRVFQKYTKYSKHDMNK

251      260       270       280       290       300
VLDLELKGDIEKCLTAIVKCATSKPAFFAEKLHQAMKGVGTRHKALIRIM

301      310       320       330       340   346
VSRSEIDMNDIKAFYQKMYGISLCQAILDETKGDYEKILVALCGGN
 SRSEIDMNDIKA
```

Denatured ANXA1 15 sec

```
1        10        20        30        40        50
MAMVSEFLKQAWFIENEEQEYVQTVKSSKGGPGSAVSPYPTFNPSSDVAA
   VSEFLKQAWFIENEEQEYVQTV
                        VKSSKGGPGSAVSPYPT
                         KSSKGGPGSAVSPYPT
                           SKGGPGSAVSPYPT
                                         FNPSSDVAA
                                         FNPSSDVAA

51       60        70        80        90        100
LHKAIMVKGVDEATIIDILTKRNNAQRQQIKAAYLQETGKPLDETLKKAL
LHKA
LHKAIMVKGVDEAT
                                AYLQETGKPLD
                                AYLQETGKPLDET
                                AYLQETGKPLDETLK
                                AYLQETGKPLDETLKKAL

101      110       120       130       140       150
TGHLEEVVLALLKTPAQFDADELRAAMKGLGTDEDTLIEILASRTNKEIR
TG
                QFDADELRAAMKGLGTDEDTLIEI

151      160       170       180       190       200
DINRVYREELKRDLAKDITSDTSGDFRNALLSLAKGDRSEDFGVNEDLAD
              AKDITSDTSGDFRNALL

201      210       220       230       240       250
SDARALYEAGERRKGTDVNVFNTILTTRSYPQLRRVFQKYTKYSKHDMNK
                       ILTTRSYPQLRRV

251      260       270       280       290       300
VLDLELKGDIEKCLTAIVKCATSKPAFFAEKLHQAMKGVGTRHKALIRIM
                    ATSKPAFFAEKLHQ
                    ATSKPAFFAEKLHQA
                    ATSKPAFFAEKLHQAMKGV
                     TSKPAFFAEKL
                      SKPAFFAEKL

301      310       320       330       340   346
VSRSEIDMNDIKAFYQKMYGISLCQAILDETKGDYEKILVALCGGN
 SRSEIDMNDIKA
                        QAILDETKGDYEKILVALCGGN
                          ILDETKGDYEKILVALC
                          ILDETKGDYEKILVALCGGN
                           LDETKGDYEKILVALCGGN
```

Denatured ANXA1 30 sec

```
1        10        20        30        40        50
MAMVSEFLKQAWFIENEEQEYVQTVKSSKGGPGSAVSPYPTFNPSSDVAA
 AMVSEFLKQA
     EFLKQAWFIENEEQEYVQTV
          AWFIENEEQEYVQTV
           WFIENEEQ
           WFIENEEQEYVQTV
                        VKSSKGGPGSAVSPYPT
                         KSSKGGPGSAVSPYPT
                           SKGGPGSAVSPYPT
                            KGGPGSAVSPYPT
                                         FNPSSDVAA
                                         FNPSSDVAA
                                         FNPSSDVAA
                                         FNPSSDVAA
                                         FNPSSDVAA

51       60        70        80        90        100
LHKAIMVKGVDEATIIDILTKRNNAQRQQIKAAYLQETGKPLDETLKKAL
LHKA
LHKAIMV
LHKAIMVK
LHKAIMVKGVDEA
LHKAIMVKGVDEAT
                                AYLQETGKP
                                AYLQETGKPL
                                AYLQETGKPLD
                                AYLQETGKPLDE
                                AYLQETGKPLDET
                                AYLQETGKPLDETL
                                AYLQETGKPLDETLK
                                AYLQETGKPLDETLKKAL
                                AYLQETGKPLDETLKKAL
                                AYLQETGKPLDETLKKAL
                                AYLQETGKPLDETLKKAL
                                 YLQETGKPLDETLKKA
                                   QETGKPLDETLKKAL
                                      GKPLDETLKKAL

101      110       120       130       140       150
TGHLEEVVLALLKTPAQFDADELRAAMKGLGTDEDTLIEILASRTNKEIR
TG
TGHL
TGHLEEV
TGHLEEV
TGHLEEV
       VLALLKTPA
       VLALLKTPAQFDADELRAA
                       RAAMKGLGTDEDTLIEI
                       RAAMKGLGTDEDTLIEIL
                         AMKGLGTDEDTLIEI
                         AMKGLGTDEDTLIEILA
                         AMKGLGTDEDTLIEILASRT

151      160       170       180       190       200
DINRVYREELKRDLAKDITSDTSGDFRNALLSLAKGDRSEDFGVNEDLAD
          KRDLAKDITSDTSGDFRNALL
              AKDITSDTSGDFRNALL
                               SLAKGDRSEDFGVNEDLAD
```

```
201      210       220       230       240       250
SDARALYEAGERRKGTDVNVFNTILTTRSYPQLRRVFQKYTKYSKHDMNK
SDARA
     LYEAGERRKGTDVNVFNT
         GERRKGTDVNVFNTILT
                  NVFNTILTTRSYPQLRRV
                   VFNTILTTRSYPQLRRV
                       ILTTRSYPQLRRV
                          TRSYPQLRRV
                                    FQKYTKYSKHDMNK
                                    FQKYTKYSKHDMNK


251      260       270       280       290       300
VLDLELKGDIEKCLTAIVKCATSKPAFFAEKLHQAMKGVGTRHKALIRIM
V
VLDL
             LTAIVKCA
                    ATSKPAFFAEKL
                    ATSKPAFFAEKLHQ
                    ATSKPAFFAEKLHQA
                    ATSKPAFFAEKLHQAM
                    ATSKPAFFAEKLHQAMKGV
                     TSKPAFFAEKL
                                HQAMKGVGTRHKALIRIM

301      310       320       330       340   346
VSRSEIDMNDIKAFYQKMYGISLCQAILDETKGDYEKILVALCGGN
VSRSEIDMNDIKA
VSRSEIDMNDIKAFYQ
VSRSEIDMNDIKAFYQKM
 SRSEIDMNDIKA
                        QAILDETKGDYEKILVALC
                        QAILDETKGDYEKILVALCGGN
                          ILDETKGDYEKILV
                          ILDETKGDYEKILVA
                          ILDETKGDYEKILVAL
                          ILDETKGDYEKILVALC
                          ILDETKGDYEKILVALCGGN
                           LDETKGDYEKILV
                           LDETKGDYEKILVALCGGN
                            DETKGDYEKILV
                               KGDYEKILVALCGGN
```

Denatured ANXA1 60 sec

```
1        10        20        30        40        50
MAMVSEFLKQAWFIENEEQEYVQTVKSSKGGPGSAVSPYPTFNPSSDVAA
 AMVSEFLKQ
 AMVSEFLKQA
     EFLKQAWFIENEEQEYVQTV
        KQAWFIENEEQEYVQTV
          AWFIENEEQEYVQTV
           WFIENEEQ
           WFIENEEQEYVQTV
           WFIENEEQEYVQTVKS
           WFIENEEQEYVQTVKSS
           WFIENEEQEYVQTVKSSKGGPGSA
                   EYVQTVKSSKGGPGSAVSPYPT
                       TVKSSKGGPGSAVSPYPT
                        VKSSKGGPGSAVSPYPT
                        VKSSKGGPGSAVSPYPTFNPSSDVA
                         KSSKGGPGSAVSPYPT
                         KSSKGGPGSAVSPYPTFNPSSD
                         KSSKGGPGSAVSPYPTFNPSSDV
                         KSSKGGPGSAVSPYPTFNPSSDVA
                         KSSKGGPGSAVSPYPTFNPSSDVAA
                           SKGGPGSAVSPYPT
                            KGGPGSAVSPYPT
                                       PTFNPSSDVAA
                                         FNPSSDVAA
                                         FNPSSDVAA
                                         FNPSSDVAA
                                         FNPSSDVAA
                                         FNPSSDVAA
                                         FNPSSDVAA
                                         FNPSSDVAA
                                          NPSSDVAA
51       60        70        80        90        100
LHKAIMVKGVDEATIIDILTKRNNAQRQQIKAAYLQETGKPLDETLKKAL
LHKA
L
LHKA
LHKAIMV
LHKAIMVK
LHKAIMVKGVDEA
LHKAIMVKGVDEAT
LHKA
    IMVKGVDEATI
    IMVKGVDEATIIDIL
    IMVKGVDEATIIDILT
    IMVKGVDEATIIDILTK
    IMVKGVDEATIIDILTKRN
    IMVKGVDEATIIDILTKRNNAQ
       KGVDEATIIDILTKRNNAQRQQIKA
                   TKRNNAQRQQIKAAYLQETGKPLDE
                       NAQRQQIKAAYL
                                AYLQETGKP
                                AYLQETGKPL
                                AYLQETGKPLD
                                AYLQETGKPLDE
                                AYLQETGKPLDET
                                AYLQETGKPLDETL
                                AYLQETGKPLDETLK
                                AYLQETGKPLDETLKKA
                                AYLQETGKPLDETLKKAL
```

```
                                AYLQETGKPLDETLKKAL
                                AYLQETGKPLDETLKKAL
                                AYLQETGKPLDETLKKAL
                                AYLQETGKPLDETLKKAL
                                AYLQETGKPLDETLKKAL
                                AYLQETGKPLDETLKKAL
                                AYLQETGKPLDETLKKAL
                                 YLQETGKPLDETL
                                 YLQETGKPLDETLKKA
                                 YLQETGKPLDETLKKAL
                                 YLQETGKPLDETLKKAL
                                   QETGKPLDETLKKAL
                                      GKPLDETLKKAL
                                        PLDETLKKAL
                                             LKKAL
                                                 L
                                                 L

101      110       120       130       140       150
TGHLEEVVLALLKTPAQFDADELRAAMKGLGTDEDTLIEILASRTNKEIR
T
TG
TGH
TGHL
TGHLE
TGHLEE
TGHLEEV
TG
TGHLEEV
TGHLEEV
TGHLEEV
TGHLEEV
TGHLEEV
TGHLEEV
TGHLEEV
TGHLEEVVLA
       VLALLKTP
       VLALLKTPA
       VLALLKTPAQ
       VLALLKTPAQF
       VLALLKTPAQFD
       VLALLKTPAQFDA
       VLALLKTPAQFDAD
       VLALLKTPAQFDADE
       VLALLKTPAQFDADEL
       VLALLKTPAQFDADELR
       VLALLKTPAQFDADELRA
       VLALLKTPAQFDADELRAA
       VLALLKTPAQFDADELRAAM
         ALLKTPAQFDADEL
         ALLKTPAQFDADELRA
          LLKTPAQFDADEL
          LLKTPAQFDADELRA
            KTPAQFDADEL
            KTPAQFDADELRA
                QFDADELRAAMKGLGTDEDTLIEI
                       RAAMKGLGTDEDTLIEI
                       RAAMKGLGTDEDTLIEIL
                       RAAMKGLGTDEDTLIEILASRT
                         AMKGLGTDEDTLIEI
                         AMKGLGTDEDTLIEIL
                         AMKGLGTDEDTLIEILA
                         AMKGLGTDEDTLIEILASRT
```

```
                                        LASRTNKEIR

151      160       170       180       190       200
DINRVYREELKRDLAKDITSDTSGDFRNALLSLAKGDRSEDFGVNEDLAD
DINRVYREEL
          KRDLAKDITSDTSGDFRNALL
              AKDITSDTSGDFRNALL
               KDITSDTSGDFRNALL
                  TSDTSGDFRNALL
                   SDTSGDFRNALL
                   SDTSGDFRNALLSLA
                      SGDFRNALLSLA
                               SLAKGDRSEDFGVNEDLA
                               SLAKGDRSEDFGVNEDLAD

201      210       220       230       240       250
SDARALYEAGERRKGTDVNVFNTILTTRSYPQLRRVFQKYTKYSKHDMNK
SDARA
     LYEAGERRKGTDVNVFNT
     LYEAGERRKGTDVNVFNTILT
         GERRKGTDVNVFNT
         GERRKGTDVNVFNTILT
                  NVFNTILTTRSYPQLRRV
                   VFNTILTTRSYPQLRRV
                       ILTTRSYPQLRRV
                          TRSYPQLRRV
                                    FQKYTKYSKHDMNK
                                    FQKYTKYSKHDMNK
                                    FQKYTKYSKHDMNK
                                    FQKYTKYSKHDMNK

251      260       270       280       290       300
VLDLELKGDIEKCLTAIVKCATSKPAFFAEKLHQAMKGVGTRHKALIRIM
V
VL
VLDL
VLDLEL
             LTAIVKCA
             LTAIVKCATSKPAFFAEKL
                    ATSKPAFFAEKL
                    ATSKPAFFAEKLHQ
                    ATSKPAFFAEKLHQA
                    ATSKPAFFAEKLHQAM
                    ATSKPAFFAEKLHQAMKGV
                    ATSKPAFFAEKLHQAMKGVGT
                     TSKPAFFAEKL
                     TSKPAFFAEKLHQAMKGV
                          FFAEKLHQ
                          FFAEKLHQA
                          FFAEKLHQAMKGV
                          FFAEKLHQAMKGVGTRHKALIRI
                          FFAEKLHQAMKGVGTRHKALIRIM
                                HQAMKGVGTRHKALIRI
                                HQAMKGVGTRHKALIRIM
                                HQAMKGVGTRHKALIRIM
                                  AMKGVGTRHKALIRIM
                                   MKGVGTRHKALIRIM
                                       GTRHKALIRIM
                                                 M
                                                 M

301      310       320       330       340   346
```

```
VSRSEIDMNDIKAFYQKMYGISLCQAILDETKGDYEKILVALCGGN
V
VSRSEIDMNDIKA
VSRSEIDMNDIKAFYQ
VSRSEIDMNDIKA
VSRSEIDMNDIKAFYQ
VSRSEIDMNDIKAFYQKM
VSRSEIDMNDIKAFYQKMYGISLC
 SRSEIDMNDIKA
 SRSEIDMNDIKAFYQ
             FYQKMYGISLC
             FYQKMYGISLCQA
                        QAILDETKGDYEKILV
                        QAILDETKGDYEKILVA
                        QAILDETKGDYEKILVALC
                        QAILDETKGDYEKILVALCGGN
                          ILDETKGDYEKILV
                          ILDETKGDYEKILVA
                          ILDETKGDYEKILVAL
                          ILDETKGDYEKILVALC
                          ILDETKGDYEKILVALCGGN
                           LDETKGDYEKILV
                           LDETKGDYEKILVALCGGN
                            DETKGDYEKILV
                               KGDYEKILVALC
                               KGDYEKILVALCGGN
```

Denatured ANXA1 90 sec

```
1        10        20        30        40        50
MAMVSEFLKQAWFIENEEQEYVQTVKSSKGGPGSAVSPYPTFNPSSDVAA
 AMVSEFLKQ
 AMVSEFLKQA
    SEFLKQAWFIENEEQEYVQTV
     EFLKQAWFIENEEQEYVQTV
        KQAWFIENEEQEYVQTV
          AWFIENEEQ
          AWFIENEEQEYVQTV
           WFIENEEQ
           WFIENEEQEYVQT
           WFIENEEQEYVQTV
           WFIENEEQEYVQTVKS
           WFIENEEQEYVQTVKSS
           WFIENEEQEYVQTVKSSKGGPGSA
                   EYVQTVKSSKGGPGSAVSPYPT
                       TVKSSKGGPGSAVSPYPT
                        VKSSKGGPGSAVSPYPT
                        VKSSKGGPGSAVSPYPTFNPSSDVA
                         KSSKGGPGSAVSPYPT
                         KSSKGGPGSAVSPYPTFNPSSD
                         KSSKGGPGSAVSPYPTFNPSSDV
                         KSSKGGPGSAVSPYPTFNPSSDVA
                         KSSKGGPGSAVSPYPTFNPSSDVAA
                           SKGGPGSAVSPYPT
                            KGGPGSAVSPYPT
                                     PTFNPSSDVAA
                                         FNPSSDVAA
                                         FNPSSDVAA
                                         FNPSSDVAA
                                         FNPSSDVAA
                                         FNPSSDVAA
                                         FNPSSDVAA
                                          NPSSDVAA
                                           PSSDVAA
51       60        70        80        90        100
LHKAIMVKGVDEATIIDILTKRNNAQRQQIKAAYLQETGKPLDETLKKAL
LHKA
L
LHKA
LHKAIMV
LHKAIMVK
LHKAIMVKGVDEA
LHKA
LHKA
    IMVKGVDEA
    IMVKGVDEATI
    IMVKGVDEATIIDI
    IMVKGVDEATIIDIL
    IMVKGVDEATIIDILT
    IMVKGVDEATIIDILTKR
    IMVKGVDEATIIDILTKRN
    IMVKGVDEATIIDILTKRNNAQ
       KGVDEATIIDILTKRNNAQRQQIKA
               IDILTKRNNAQRQQIKA
                   TKRNNAQRQQIKAAYLQET
                   TKRNNAQRQQIKAAYLQETGKPLDE
                       NAQRQQIKAAYL
                       NAQRQQIKAAYLQET
                                AYLQETGKP
```

```
                                AYLQETGKPL
                                AYLQETGKPLD
                                AYLQETGKPLDE
                                AYLQETGKPLDET
                                AYLQETGKPLDETL
                                AYLQETGKPLDETLK
                                AYLQETGKPLDETLKKA
                                AYLQETGKPLDETLKKAL
                                AYLQETGKPLDETLKKAL
                                AYLQETGKPLDETLKKAL
                                AYLQETGKPLDETLKKAL
                                AYLQETGKPLDETLKKAL
                                AYLQETGKPLDETLKKAL
                                AYLQETGKPLDETLKKAL
                                AYLQETGKPLDETLKKAL
                                 YLQETGKPLDET
                                 YLQETGKPLDETL
                                 YLQETGKPLDETLKKA
                                 YLQETGKPLDETLKKAL
                                   QETGKPLDETLKKAL
                                      GKPLDETLKKAL
                                        PLDETLKKAL
                                             LKKAL
                                                 L
                                                 L

101      110       120       130       140       150
TGHLEEVVLALLKTPAQFDADELRAAMKGLGTDEDTLIEILASRTNKEIR
T
TG
TGH
TGHL
TGHLE
TGHLEE
TGHLEEV
TGHLEEV
TG
TGHLEEV
TGHLEEV
TGHLEEV
TGHLEEV
TGHLEEVVLA
       VLALLKTP
       VLALLKTPA
       VLALLKTPAQ
       VLALLKTPAQF
       VLALLKTPAQFD
       VLALLKTPAQFDA
       VLALLKTPAQFDAD
       VLALLKTPAQFDADE
       VLALLKTPAQFDADEL
       VLALLKTPAQFDADELR
       VLALLKTPAQFDADELRA
       VLALLKTPAQFDADELRAA
       VLALLKTPAQFDADELRAAM
        LALLKTPAQFDADELRA
         ALLKTPAQFDADEL
         ALLKTPAQFDADELRA
          LLKTPAQFDADEL
          LLKTPAQFDADELRA
           LKTPAQFDADELRAA
            KTPAQFDADEL
            KTPAQFDADELRA
```

```
                QFDADELRA
                       RAAMKGLGTDEDTLIEI
                       RAAMKGLGTDEDTLIEIL
                       RAAMKGLGTDEDTLIEILASRT
                         AMKGLGTDEDTLIEI
                         AMKGLGTDEDTLIEIL
                         AMKGLGTDEDTLIEILA
                         AMKGLGTDEDTLIEILASRT
                                         ASRTNKEIR
                                             NKEIR
                                             NKEIR

151      160       170       180       190       200
DINRVYREELKRDLAKDITSDTSGDFRNALLSLAKGDRSEDFGVNEDLAD
DI
DINRVYREEL
DINRVYREELKRDLAKDIT
          KRDLAKDITSDTSGDFRNALL
              AKDITSDTSGDFRNALL
               KDITSDTSGDFRNALL
                  TSDTSGDFRNALL
                   SDTSGDFRNALL
                   SDTSGDFRNALLSLA
                               SLAKGDRSEDFGVNEDLA
                               SLAKGDRSEDFGVNEDLAD
                               SLAKGDRSEDFGVNEDLAD

201      210       220       230       240       250
SDARALYEAGERRKGTDVNVFNTILTTRSYPQLRRVFQKYTKYSKHDMNK
SDARA
SDARAL
     LYEAGERRKGTDVNVFNT
     LYEAGERRKGTDVNVFNTILT
         GERRKGTDVNVFNT
         GERRKGTDVNVFNTILT
                  NVFNTILTTRSYPQLRRV
                   VFNTILTTRSYPQLRRV
                       ILTTRSYPQLRRV
                          TRSYPQLRRV
                          TRSYPQLRRVFQ
                                    FQKYTKYSKHDMNK
                                    FQKYTKYSKHDMNK
                                    FQKYTKYSKHDMNK
                                    FQKYTKYSKHDMNK
                                        KYSKHDMNK

251      260       270       280       290       300
VLDLELKGDIEKCLTAIVKCATSKPAFFAEKLHQAMKGVGTRHKALIRIM
V
VL
VLDL
VLDLEL
VLDLELKGDIEKC
             LTAIVKCATSKPAFFAEKL
               AIVKCATSKPAFFAEKL
                    ATSKPAFFAEKL
                    ATSKPAFFAEKLHQ
                    ATSKPAFFAEKLHQA
                    ATSKPAFFAEKLHQAM
                    ATSKPAFFAEKLHQAMKGV
                    ATSKPAFFAEKLHQAMKGVGT
                    ATSKPAFFAEKLHQAMKGVGTRHKA
                     TSKPAFFAEKL
```

```
                     TSKPAFFAEKLHQAMKGV
                        PAFFAEKLHQA
                          FFAEKLHQ
                          FFAEKLHQA
                          FFAEKLHQAM
                          FFAEKLHQAMKGV
                          FFAEKLHQAMKGVGTRHKALIRI
                          FFAEKLHQAMKGVGTRHKALIRIM
                                HQAMKGVGTRHKALIRI
                                HQAMKGVGTRHKALIRIM
                                HQAMKGVGTRHKALIRIM
                                HQAMKGVGTRHKALIRIM
                                  AMKGVGTRHKALIRIM
                                   MKGVGTRHKALIRIM
                                       GTRHKALIRIM
                                              IRIM
                                                 M
                                                 M

301      310       320       330       340   346
VSRSEIDMNDIKAFYQKMYGISLCQAILDETKGDYEKILVALCGGN
V
VS
VSRSEIDMNDIKA
VSRSEIDMNDIKA
VSRSEIDMNDIKAFYQ
VSRSEIDMNDI
VSRSEIDMNDIKA
VSRSEIDMNDIKAFYQ
VSRSEIDMNDIKAFYQKM
VSRSEIDMNDIKAFYQKMYGI
VSRSEIDMNDIKAFYQKMYGISLC
 SRSEIDMNDIKA
 SRSEIDMNDIKAFYQ
  RSEIDMNDIKA
  RSEIDMNDIKAFYQ
             FYQKMYGI
             FYQKMYGISLC
             FYQKMYGISLCQA
                KMYGISLC
                        QAILDETKGDYEKIL
                        QAILDETKGDYEKILV
                        QAILDETKGDYEKILVA
                        QAILDETKGDYEKILVALC
                        QAILDETKGDYEKILVALCGGN
                          ILDETKGDYEKILV
                          ILDETKGDYEKILVA
                          ILDETKGDYEKILVAL
                          ILDETKGDYEKILVALC
                          ILDETKGDYEKILVALCGGN
                           LDETKGDYEKILV
                           LDETKGDYEKILVALCGGN
                            DETKGDYEKILV
                               KGDYEKILVALC
                               KGDYEKILVALCGGN
```

Denatured ANXA1 120 sec

```
1        10        20        30        40        50
MAMVSEFLKQAWFIENEEQEYVQTVKSSKGGPGSAVSPYPTFNPSSDVAA
 AMVSEFLKQ
 AMVSEFLKQA
   VSEFLKQA
   VSEFLKQAWFIENEEQEYVQTV
    SEFLKQAWFIENEEQEYVQTV
     EFLKQAWFIENEEQEYVQTV
     EFLKQAWFIENEEQEYVQTVKS
        KQAWFIENEEQEYVQTV
          AWFIENEEQ
          AWFIENEEQEYVQTV
           WFIENEEQ
           WFIENEEQEYV
           WFIENEEQEYVQT
           WFIENEEQEYVQTV
           WFIENEEQEYVQTVKS
           WFIENEEQEYVQTVKSS
           WFIENEEQEYVQTVKSSKGGPGSA
              ENEEQEYVQTV
                   EYVQTVKSSKGGPGSAVSPYPT
                       TVKSSKGGPGSAVSPYPT
                        VKSSKGGPGSAVSPYPT
                        VKSSKGGPGSAVSPYPTFNPSSDVA
                         KSSKGGPGSAVSPYPT
                         KSSKGGPGSAVSPYPTFNPSSD
                         KSSKGGPGSAVSPYPTFNPSSDV
                         KSSKGGPGSAVSPYPTFNPSSDVA
                         KSSKGGPGSAVSPYPTFNPSSDVAA
                           SKGGPGSAVSPYPT
                            KGGPGSAVSPYPT
                                       PTFNPSSDVAA
                                         FNPSSDVAA
                                         FNPSSDVAA
                                         FNPSSDVAA
                                         FNPSSDVAA
                                         FNPSSDVAA
                                         FNPSSDVAA
                                          NPSSDVAA
                                           PSSDVAA
                                            SSDVAA
                                                 A


51       60        70        80        90        100
LHKAIMVKGVDEATIIDILTKRNNAQRQQIKAAYLQETGKPLDETLKKAL
LHKA
L
LHKA
LHKAIMV
LHKAIMVK
LHKAIMVKGVDEA
LHKA
LHKA
LHKA
LHKAIMVKGVDEATIIDIL
    IMVKGVDEA
    IMVKGVDEATI
    IMVKGVDEATIIDI
    IMVKGVDEATIIDIL
```

```
    IMVKGVDEATIIDILT
    IMVKGVDEATIIDILTK
    IMVKGVDEATIIDILTKR
    IMVKGVDEATIIDILTKRN
    IMVKGVDEATIIDILTKRNNA
    IMVKGVDEATIIDILTKRNNAQ
       KGVDEATIIDIL
       KGVDEATIIDILTKRNNAQRQQIKA
               IDILTKRNNAQRQQIKA
                   TKRNNAQRQQIKAAYL
                   TKRNNAQRQQIKAAYLQETGKPLDE
                       NAQRQQIKAAYL
                       NAQRQQIKAAYLQET
                                AYLQETGKP
                                AYLQETGKPL
                                AYLQETGKPLD
                                AYLQETGKPLDE
                                AYLQETGKPLDET
                                AYLQETGKPLDETL
                                AYLQETGKPLDETLK
                                AYLQETGKPLDETLKK
                                AYLQETGKPLDETLKKA
                                AYLQETGKPLDETLKKAL
                                AYLQETGKPLDETLKKAL
                                AYLQETGKPLDETLKKAL
                                AYLQETGKPLDETLKKAL
                                AYLQETGKPLDETLKKAL
                                AYLQETGKPLDETLKKAL
                                AYLQETGKPLDETLKKAL
                                AYLQETGKPLDETLKKAL
                                 YLQETGKPLDET
                                 YLQETGKPLDETL
                                 YLQETGKPLDETLKKA
                                 YLQETGKPLDETLKKAL
                                 YLQETGKPLDETLKKAL
                                 YLQETGKPLDETLKKAL
                                 YLQETGKPLDETLKKAL
                                   QETGKPLDETLKKAL
                                   QETGKPLDETLKKAL
                                      GKPLDETLKKAL
                                        PLDETLKKAL
                                             LKKAL
                                                AL
                                                 L
101      110       120       130       140      150
TGHLEEVVLALLKTPAQFDADELRAAMKGLGTDEDTLIEILASRTNKEIR
T
TG
TGH
TGHL
TGHLE
TGHLEE
TGHLEEV
T
TG
TGHLEEV
T
TGHLEEV
TGHLEEV
TGHLEEV
TGHLEEV
TGHLEEV
TGHLEEV
```

```
       VLALLKTP
       VLALLKTPA
       VLALLKTPAQ
       VLALLKTPAQF
       VLALLKTPAQFD
       VLALLKTPAQFDA
       VLALLKTPAQFDAD
       VLALLKTPAQFDADE
       VLALLKTPAQFDADEL
       VLALLKTPAQFDADELR
       VLALLKTPAQFDADELRA
       VLALLKTPAQFDADELRAA
       VLALLKTPAQFDADELRAAM
        LALLKTPAQFDADELRA
         ALLKTPAQFDADEL
         ALLKTPAQFDADELRA
            KTPAQFDADEL
            KTPAQFDADELRA
            KTPAQFDADELRAA
                       RAAMKGLGTDEDTLIEI
                       RAAMKGLGTDEDTLIEIL
                       RAAMKGLGTDEDTLIEILASRT
                         AMKGLGTDEDTLIEI
                         AMKGLGTDEDTLIEIL
                         AMKGLGTDEDTLIEILA
                         AMKGLGTDEDTLIEILASRT
                                        LASRTNKEIR
                                        LASRTNKEIR
                                         ASRTNKEIR
                                         ASRTNKEIR
                                             NKEIR
                                             NKEIR


151      160       170       180       190       200
DINRVYREELKRDLAKDITSDTSGDFRNALLSLAKGDRSEDFGVNEDLAD
DI
DINRVYREEL
DI
DINRVYREEL
DINRVYREEL
DINRVYREELKRDLAKDIT
          KRDLAKDITSDTSGDFRNALL
              AKDITSDTSGDFRNALL
               KDITSDTSGDFRNALL
                  TSDTSGDFRNALL
                  TSDTSGDFRNALLSLA
                   SDTSGDFRNALL
                   SDTSGDFRNALLSLA
                      SGDFRNALLSLA
                               SLAKGDRSEDFGVNEDLA
                               SLAKGDRSEDFGVNEDLAD
                               SLAKGDRSEDFGVNEDLAD
                                  KGDRSEDFGVNEDLAD
                                  KGDRSEDFGVNEDLAD

201      210       220       230       240       250
SDARALYEAGERRKGTDVNVFNTILTTRSYPQLRRVFQKYTKYSKHDMNK
SDARA
SDARAL
SDARA
SDARALYEA
     LYEAGERRKGTDVNVFNT
```

```
     LYEAGERRKGTDVNVFNTILT
         GERRKGTDVNVFNT
         GERRKGTDVNVFNTILT
                  NVFNTILTTRSYPQLRRV
                   VFNTILTTRSYPQLRRV
                       ILTTRSYPQLRRV
                          TRSYPQLRRV
                          TRSYPQLRRVFQ
                                    FQKYTKYSKHDMNK
                                    FQKYTKYSKHDMNK
                                    FQKYTKYSKHDMNK
                                    FQKYTKYSKHDMNK
                                      KYTKYSKHDMNK
                                         KYSKHDMNK

251      260       270       280       290       300
VLDLELKGDIEKCLTAIVKCATSKPAFFAEKLHQAMKGVGTRHKALIRIM
V
VL
VLDL
VLDLEL
VLDLELKGDIEKC
VLDLELKGDIEKCLT
             LTAIVKCA
             LTAIVKCATSKPA
               AIVKCATSKPAFFAEKL
                    ATSKPAFFAEKL
                    ATSKPAFFAEKLHQ
                    ATSKPAFFAEKLHQA
                    ATSKPAFFAEKLHQAM
                    ATSKPAFFAEKLHQAMKGV
                    ATSKPAFFAEKLHQAMKGVGT
                    ATSKPAFFAEKLHQAMKGVGTRHKA
                     TSKPAFFAEKL
                     TSKPAFFAEKLHQ
                     TSKPAFFAEKLHQAMKGV
                        PAFFAEKLHQA
                          FFAEKLHQ
                          FFAEKLHQA
                          FFAEKLHQAM
                          FFAEKLHQAMKGV
                          FFAEKLHQAMKGVGTRHKALIRI
                          FFAEKLHQAMKGVGTRHKALIRIM
                                HQAMKGVGTRHKALI
                                HQAMKGVGTRHKALIRI
                                HQAMKGVGTRHKALIRIM
                                HQAMKGVGTRHKALIRIM
                                HQAMKGVGTRHKALIRIM
                                  AMKGVGTRHKALIRIM
                                   MKGVGTRHKALIRIM
                                   MKGVGTRHKALIRIM
                                     GVGTRHKALIRIM
                                       GTRHKALIRIM
                                              IRIM
                                                 M
                                                 M

301      310       320       330       340   346
VSRSEIDMNDIKAFYQKMYGISLCQAILDETKGDYEKILVALCGGN
V
VS
VS
VSRSEIDMNDIKA
```

```
VSRSEIDMNDIKA
VSRSEIDMNDIKAFYQ
VSRSEIDMNDI
VSRSEIDMNDIKA
VSRSEIDMNDIKAFYQ
VSRSEIDMNDIKAFYQKM
VSRSEIDMNDIKAFYQKMYGI
VSRSEIDMNDIKAFYQKMYGISLC
 SRSEIDMNDIKA
 SRSEIDMNDIKAFYQ
  RSEIDMNDIKA
  RSEIDMNDIKAFYQ
             FYQKMYGI
             FYQKMYGISLC
             FYQKMYGISLCQA
                KMYGISLC
                        QAILDETKGDYEKIL
                        QAILDETKGDYEKILV
                        QAILDETKGDYEKILVA
                        QAILDETKGDYEKILVALC
                        QAILDETKGDYEKILVALCGGN
                          ILDETKGDYEKIL
                          ILDETKGDYEKILV
                          ILDETKGDYEKILVA
                          ILDETKGDYEKILVAL
                          ILDETKGDYEKILVALC
                          ILDETKGDYEKILVALCGGN
                           LDETKGDYEKILV
                           LDETKGDYEKILVALC
                           LDETKGDYEKILVALCGGN
                            DETKGDYEKILV
                               KGDYEKILVALC
                               KGDYEKILVALCGGN
```

Denatured ANXA1 300 sec

```
1        10        20        30        40        50
MAMVSEFLKQAWFIENEEQEYVQTVKSSKGGPGSAVSPYPTFNPSSDVAA
 AMVSEFLKQ
 AMVSEFLKQA
  MVSEFLKQ
  MVSEFLKQA
   VSEFLKQA
   VSEFLKQAWFIENEEQEYVQTV
    SEFLKQAWFIENEEQEYVQTV
     EFLKQAWFIENEEQEYVQTV
     EFLKQAWFIENEEQEYVQTVKS
        KQAWFIENEEQEYVQTV
          AWFIENEEQ
          AWFIENEEQEYVQT
          AWFIENEEQEYVQTV
          AWFIENEEQEYVQTVKS
           WFIENEEQ
           WFIENEEQEYV
           WFIENEEQEYVQT
           WFIENEEQEYVQTV
           WFIENEEQEYVQTVKS
           WFIENEEQEYVQTVKSS
           WFIENEEQEYVQTVKSSKGGPGSA
              ENEEQEYVQTV
                   EYVQTVKSSKGGPGSAVSPYPT
                      QTVKSSKGGPGSAVSPYPT
                       TVKSSKGGPGSAVSPYPT
                        VKSSKGGPGSAVSPYPT
                        VKSSKGGPGSAVSPYPTFNPSSD
                        VKSSKGGPGSAVSPYPTFNPSSDVA
                         KSSKGGPGSAVSPYPT
                         KSSKGGPGSAVSPYPTFNPSS
                         KSSKGGPGSAVSPYPTFNPSSD
                         KSSKGGPGSAVSPYPTFNPSSDV
                         KSSKGGPGSAVSPYPTFNPSSDVA
                         KSSKGGPGSAVSPYPTFNPSSDVAA
                          SSKGGPGSAVSPYPT
                           SKGGPGSAVSPYPT
                            KGGPGSAVSPYPT
                                       PTFNPSSDVAA
                                        TFNPSSDVAA
                                         FNPSSDVAA
                                         FNPSSDVAA
                                         FNPSSDVAA
                                         FNPSSDVAA
                                         FNPSSDVAA
                                         FNPSSDVAA
                                          NPSSDVAA
                                           PSSDVAA
                                            SSDVAA
                                                 A

51       60        70        80        90        100
LHKAIMVKGVDEATIIDILTKRNNAQRQQIKAAYLQETGKPLDETLKKAL
LHKA
LHKA
L
LHKA
LHKAIMV
```

```
LHKAIMVK
LHKAIMVKGVDEA
LHKA
LHKA
LHKA
LHKAIMVKGVDEATIIDIL
    IMVKGVDEA
    IMVKGVDEATI
    IMVKGVDEATIIDI
    IMVKGVDEATIIDIL
    IMVKGVDEATIIDILT
    IMVKGVDEATIIDILTK
    IMVKGVDEATIIDILTKR
    IMVKGVDEATIIDILTKRN
    IMVKGVDEATIIDILTKRNNA
    IMVKGVDEATIIDILTKRNNAQ
     MVKGVDEATIIDIL
      VKGVDEATIIDIL
       KGVDEATIIDIL
       KGVDEATIIDILT
       KGVDEATIIDILTKRN
       KGVDEATIIDILTKRNNAQRQQIKA
               IDILTKRNNAQRQQIKA
                   TKRNNAQRQQIKAAYL
                   TKRNNAQRQQIKAAYLQET
                   TKRNNAQRQQIKAAYLQETGKPLDE
                      NNAQRQQIKAAYL
                      NNAQRQQIKAAYLQET
                       NAQRQQIKAAYL
                       NAQRQQIKAAYLQET
                              KAAYLQET
                                AYLQETGKP
                                AYLQETGKPL
                                AYLQETGKPLD
                                AYLQETGKPLDE
                                AYLQETGKPLDET
                                AYLQETGKPLDETL
                                AYLQETGKPLDETLK
                                AYLQETGKPLDETLKKA
                                AYLQETGKPLDETLKKAL
                                AYLQETGKPLDETLKKAL
                                AYLQETGKPLDETLKKAL
                                AYLQETGKPLDETLKKAL
                                AYLQETGKPLDETLKKAL
                                AYLQETGKPLDETLKKAL
                                AYLQETGKPLDETLKKAL
                                AYLQETGKPLDETLKKAL
                                 YLQETGKPLDE
                                 YLQETGKPLDET
                                 YLQETGKPLDETL
                                 YLQETGKPLDETLKKA
                                 YLQETGKPLDETLKKAL
                                 YLQETGKPLDETLKKAL
                                 YLQETGKPLDETLKKAL
                                   QETGKPLDET
                                   QETGKPLDETLKKAL
                                   QETGKPLDETLKKAL
                                    ETGKPLDETLKKAL
                                      GKPLDETLKKAL
                                        PLDETLKKAL
                                             LKKAL
                                              KKAL
                                                AL
```

```
                                                 L


101      110       120       130       140       150
TGHLEEVVLALLKTPAQFDADELRAAMKGLGTDEDTLIEILASRTNKEIR
T
TG
TGH
TGHL
TGHLE
TGHLEE
TGHLEEV
T
TG
TGHLEEV
T
TGHLEEV
TGHLEEV
TGHLEEV
TGHLEEV
TGHLEEV
TGHLEEV
TGHLEEV
TGHLEEV
       VLALLKTP
       VLALLKTPA
       VLALLKTPAQ
       VLALLKTPAQF
       VLALLKTPAQFD
       VLALLKTPAQFDA
       VLALLKTPAQFDAD
       VLALLKTPAQFDADE
       VLALLKTPAQFDADEL
       VLALLKTPAQFDADELR
       VLALLKTPAQFDADELRA
       VLALLKTPAQFDADELRAA
       VLALLKTPAQFDADELRAAM
        LALLKTPAQFDADEL
        LALLKTPAQFDADELRA
         ALLKTPAQFDADEL
         ALLKTPAQFDADELRA
         ALLKTPAQFDADELRAA
         ALLKTPAQFDADELRAAM
          LLKTPAQFDADEL
          LLKTPAQFDADELRA
          LLKTPAQFDADELRAAM
           LKTPAQFDADEL
           LKTPAQFDADELRA
            KTPAQFDADEL
            KTPAQFDADELRA
            KTPAQFDADELRAA
                QFDADELRA
                       RAAMKGLGTDEDTLIEI
                       RAAMKGLGTDEDTLIEIL
                       RAAMKGLGTDEDTLIEILA
                       RAAMKGLGTDEDTLIEILASRT
                         AMKGLGTDEDTLIEI
                         AMKGLGTDEDTLIEIL
                         AMKGLGTDEDTLIEILA
                         AMKGLGTDEDTLIEILASRT
                          MKGLGTDEDTLIEI
                          MKGLGTDEDTLIEIL
                          MKGLGTDEDTLIEILASRT
```

```
                           KGLGTDEDTLIEIL
                                        LASRTNKEIR
                                        LASRTNKEIR
                                         ASRTNKEIR
                                         ASRTNKEIR
                                             NKEIR
                                             NKEIR
                                             NKEIR

151      160       170       180       190       200
DINRVYREELKRDLAKDITSDTSGDFRNALLSLAKGDRSEDFGVNEDLAD
DI
DINRVYREEL
DI
DINRVYREEL
DINRV
DINRVYREEL
DINRVYREELKRDLAKDIT
     YREELKRDLAKDITSDT
     YREELKRDLAKDITSDTSGDFRNA
          KRDLAKDITSDT
          KRDLAKDITSDTSGDFRNALL
          KRDLAKDITSDTSGDFRNALLSLA
              AKDITSDTSGDFRNALL
               KDITSDTSGDFRNALL
                  TSDTSGDFRNALL
                  TSDTSGDFRNALLSLA
                   SDTSGDFRNA
                   SDTSGDFRNALL
                   SDTSGDFRNALLSL
                   SDTSGDFRNALLSLA
                      SGDFRNALLSL
                      SGDFRNALLSLA
                               SLAKGDRSEDFGV
                               SLAKGDRSEDFGVNEDLA
                               SLAKGDRSEDFGVNEDLAD
                               SLAKGDRSEDFGVNEDLAD
                               SLAKGDRSEDFGVNEDLAD
                                LAKGDRSEDFGVNEDLAD
                                  KGDRSEDFGVNEDLAD
                                  KGDRSEDFGVNEDLAD

201      210       220       230       240       250
SDARALYEAGERRKGTDVNVFNTILTTRSYPQLRRVFQKYTKYSKHDMNK
SDA
SDARA
SDARAL
SDARA
SDARA
SDARALYEA
     LYEAGERRKGTDVNVFNT
     LYEAGERRKGTDVNVFNTILT
         GERRKGTDVNVFNT
         GERRKGTDVNVFNTILT
                  NVFNTILTTRSYPQLRRV
                   VFNTILTTRSYPQLRRV
                     NTILTTRSYPQLRRV
                       ILTTRSYPQL
                       ILTTRSYPQLRRV
                          TRSYPQLRRV
                          TRSYPQLRRVFQ
                                    FQKYTKYSKHDM
                                    FQKYTKYSKHDMNK
```

```
                                    FQKYTKYSKHDMNK
                                    FQKYTKYSKHDMNK
                                    FQKYTKYSKHDMNK
                                      KYTKYSKHDMNK
                                         KYSKHDMNK
                                         KYSKHDMNK
                                         KYSKHDMNK
                                            KHDMNK

251      260       270       280       290       300
VLDLELKGDIEKCLTAIVKCATSKPAFFAEKLHQAMKGVGTRHKALIRIM
V
VL
VLDL
VLDLEL
VLDLELKGDIEKC
VLDL
VLDLELKGDIEKC
VLDLELKGDIEKCLT
VLDLELKGDIEKC
    ELKGDIEKC
    ELKGDIEKCLTAIVKC
      KGDIEKCLTAIVKC
             LTAIVKCA
             LTAIVKCATSKPA
             LTAIVKCATSKPAFFAEKL
               AIVKCATSKPAFFAEKL
                 VKCATSKPAFFAEKL
                  KCATSKPAFFAEKL
                    ATSKPAFFAEKL
                    ATSKPAFFAEKLHQ
                    ATSKPAFFAEKLHQA
                    ATSKPAFFAEKLHQAM
                    ATSKPAFFAEKLHQAMKGV
                    ATSKPAFFAEKLHQAMKGVGT
                    ATSKPAFFAEKLHQAMKGVGTRHKA
                     TSKPAFFAEKL
                     TSKPAFFAEKLHQ
                     TSKPAFFAEKLHQAMKGV
                      SKPAFFAEKL
                        PAFFAEKLHQA
                          FFAEKLHQ
                          FFAEKLHQA
                          FFAEKLHQAM
                          FFAEKLHQAMKGV
                          FFAEKLHQAMKGVGTRHKALIRI
                          FFAEKLHQAMKGVGTRHKALIRIM
                          FFAEKLHQAMKGVGTRHKALIRIM
                                HQAMKGVGTRHKALI
                                HQAMKGVGTRHKALIRI
                                HQAMKGVGTRHKALIRIM
                                HQAMKGVGTRHKALIRIM
                                HQAMKGVGTRHKALIRIM
                                  AMKGVGTRHKALIRIM
                                   MKGVGTRHKALIRIM
                                   MKGVGTRHKALIRIM
                                     GVGTRHKALIRIM
                                       GTRHKALIRIM
                                              IRIM
                                               RIM
                                                 M
                                                 M
```

```
                                                 M
                                                 M
                                                 M

301      310       320       330       340   346
VSRSEIDMNDIKAFYQKMYGISLCQAILDETKGDYEKILVALCGGN
V
V
VS
VS
VSRSEIDMNDIKA
VSRSEIDMNDIKA
VSRSEIDMNDI
VSRSEIDMNDIKA
VSRSEIDMNDIKAFYQ
VSRSEIDMNDIKAFYQKM
VSRSEIDMNDIKAFYQKMYGISLC
VSRSEIDMNDI
VSRSEIDMNDIKA
VSRSEIDMNDIKAFYQ
VSRSEIDMNDIKAFYQKM
VSRSEIDMNDIKAFYQKMYGI
VSRSEIDMNDIKAFYQKMYGISLC
 SRSEIDMNDIKA
 SRSEIDMNDIKAFYQ
 SRSEIDMNDIKAFYQKM
 SRSEIDMNDIKAFYQKMYGISLC
  RSEIDMNDIKA
  RSEIDMNDIKAFYQ
    EIDMNDIKA
             FYQKMYGI
             FYQKMYGISLC
             FYQKMYGISLCQA
                KMYGISLC
                KMYGISLCQA
                        QAILDETKGDYEKI
                        QAILDETKGDYEKIL
                        QAILDETKGDYEKILV
                        QAILDETKGDYEKILVA
                        QAILDETKGDYEKILVALC
                        QAILDETKGDYEKILVALCGGN
                          ILDETKGDYEKIL
                          ILDETKGDYEKILV
                          ILDETKGDYEKILVA
                          ILDETKGDYEKILVAL
                          ILDETKGDYEKILVALC
                          ILDETKGDYEKILVALCGGN
                           LDETKGDYEKILV
                           LDETKGDYEKILVALC
                           LDETKGDYEKILVALCGGN
                            DETKGDYEKILV
                               KGDYEKILVALC
                               KGDYEKILVALCGGN
```

Denatured ANXA1 600 sec

```
1        10        20        30        40        50
MAMVSEFLKQAWFIENEEQEYVQTVKSSKGGPGSAVSPYPTFNPSSDVAA
 AMVSEFLKQ
 AMVSEFLKQA
  MVSEFLKQ
  MVSEFLKQA
   VSEFLKQA
VSEFLKQAWFIENEEQEYVQTV
 SEFLKQAWFIENEEQEYVQTV
  EFLKQAWFIENEEQEYVQT
  EFLKQAWFIENEEQEYVQTV
  EFLKQAWFIENEEQEYVQTVKS
     KQAWFIENEEQEYVQTV
       AWFIENEEQ
       AWFIENEEQEYVQT
       AWFIENEEQEYVQTV
       AWFIENEEQEYVQTVKS
        WFIENEEQ
        WFIENEEQEYV
        WFIENEEQEYVQ
        WFIENEEQEYVQT
        WFIENEEQEYVQTV
        WFIENEEQEYVQTVKS
        WFIENEEQEYVQTVKSS
        WFIENEEQEYVQTVKSSKGGPGSA
           ENEEQEYVQTV
                EYVQTVKSSKGGPGSAVSPYPT
                   QTVKSSKGGPGSAVSPYPT
                    TVKSSKGGPGSAVSPYPT
                     VKSSKGGPGSAVSPYPT
                     VKSSKGGPGSAVSPYPTFNPSSD
                     VKSSKGGPGSAVSPYPTFNPSSDVA
                      KSSKGGPGSAVSPYPT
                      KSSKGGPGSAVSPYPTFNPSS
                      KSSKGGPGSAVSPYPTFNPSSD
                      KSSKGGPGSAVSPYPTFNPSSDV
                      KSSKGGPGSAVSPYPTFNPSSDVA
                       SSKGGPGSAVSPYPT
                        SKGGPGSAVSPYPT
                         KGGPGSAVSPYPT
                                    PTFNPSSDVAA
                                     TFNPSSDVAA
                                      FNPSSDVAA
                                      FNPSSDVAA
                                      FNPSSDVAA
                                      FNPSSDVAA
                                      FNPSSDVAA
                                      FNPSSDVAA
                                      FNPSSDVAA
                                       NPSSDVAA
                                        PSSDVAA
                                         SSDVAA
                                              A

51       60        70        80        90        100
LHKAIMVKGVDEATIIDILTKRNNAQRQQIKAAYLQETGKPLDETLKKAL
LHKA
LHKA
L
LHKA
LHKAIM
```

```
LHKAIMV
LHKAIMVK
LHKAIMVKGVDEA
LHKA
LHKA
LHKA
LHKAIMVKGVDEATIIDIL
    IMVKGVDEA
    IMVKGVDEATI
    IMVKGVDEATIIDI
    IMVKGVDEATIIDIL
    IMVKGVDEATIIDILT
    IMVKGVDEATIIDILTK
    IMVKGVDEATIIDILTKR
    IMVKGVDEATIIDILTKRN
    IMVKGVDEATIIDILTKRNNA
    IMVKGVDEATIIDILTKRNNAQ
    IMVKGVDEATIIDILTKRNNAQRQ
     MVKGVDEATIIDIL
      VKGVDEATIIDIL
      VKGVDEATIIDILT
      VKGVDEATIIDILTKRN
       KGVDEATIIDIL
       KGVDEATIIDILT
       KGVDEATIIDILTKRNNAQRQQIKA
               IDILTKRNNAQRQQIKA
                   TKRNNAQRQQIKAAYL
                   TKRNNAQRQQIKAAYLQET
                   TKRNNAQRQQIKAAYLQETGKPLDE
                    KRNNAQRQQIKAAYL
                      NNAQRQQIKAAYL
                      NNAQRQQIKAAYLQET
                       NAQRQQIKAAYL
                       NAQRQQIKAAYLQET
                              KAAYLQET
                                AYLQETGKP
                                AYLQETGKPL
                                AYLQETGKPLD
                                AYLQETGKPLDE
                                AYLQETGKPLDET
                                AYLQETGKPLDETL
                                AYLQETGKPLDETLK
                                AYLQETGKPLDETLKKA
                                AYLQETGKPLDETLKKAL
                                AYLQETGKPLDETLKKAL
                                AYLQETGKPLDETLKKAL
                                AYLQETGKPLDETLKKAL
                                AYLQETGKPLDETLKKAL
                                AYLQETGKPLDETLKKAL
                                AYLQETGKPLDETLKKAL
                                 YLQETGKPLDE
                                 YLQETGKPLDET
                                 YLQETGKPLDETLKKA
                                 YLQETGKPLDETLKKAL
                                 YLQETGKPLDETLKKAL
                                 YLQETGKPLDETLKKAL
                                   QETGKPLDET
                                   QETGKPLDETLKKAL
                                   QETGKPLDETLKKAL
                                    ETGKPLDETLKKAL
                                      GKPLDETLKKAL
                                        PLDETLKKAL
                                             LKKAL
```

```
                                              KKAL
                                                AL
                                                 L


101      110       120       130       140       150
TGHLEEVVLALLKTPAQFDADELRAAMKGLGTDEDTLIEILASRTNKEIR
T
TG
TGH
TGHL
TGHLE
TGHLEEV
T
TG
TGHLEEV
T
TGHLEEV
TGHLEEV
TGHLEEV
TGHLEEV
TGHLEEV
TGHLEEV
TGHLEEV
TGHLEEV
       VLALLKTP
       VLALLKTPA
       VLALLKTPAQ
       VLALLKTPAQF
       VLALLKTPAQFD
       VLALLKTPAQFDA
       VLALLKTPAQFDAD
       VLALLKTPAQFDADE
       VLALLKTPAQFDADEL
       VLALLKTPAQFDADELR
       VLALLKTPAQFDADELRA
       VLALLKTPAQFDADELRAA
       VLALLKTPAQFDADELRAAM
       VLALLKTPAQFDADELRAAMK
       VLALLKTPAQFDADELRAAMKG
        LALLKTPAQFDADEL
        LALLKTPAQFDADELRA
        LALLKTPAQFDADELRAA
         ALLKTPAQFDADEL
         ALLKTPAQFDADELRA
         ALLKTPAQFDADELRAA
         ALLKTPAQFDADELRAAM
          LLKTPAQFDADEL
          LLKTPAQFDADELRA
          LLKTPAQFDADELRAA
           LKTPAQFDADELRA
            KTPAQFDADEL
            KTPAQFDADELRA
            KTPAQFDADELRAA
            KTPAQFDADELRAAM
                QFDADELRA
                QFDADELRAA
                       RAAMKGLGTDEDTLIEI
                       RAAMKGLGTDEDTLIEIL
                       RAAMKGLGTDEDTLIEILA
                       RAAMKGLGTDEDTLIEILASRT
                         AMKGLGTDEDTLIE
                         AMKGLGTDEDTLIEI
```

```
                         AMKGLGTDEDTLIEIL
                         AMKGLGTDEDTLIEILA
                         AMKGLGTDEDTLIEILASRT
                          MKGLGTDEDTLIEI
                          MKGLGTDEDTLIEIL
                          MKGLGTDEDTLIEILASRT
                           KGLGTDEDTLIEIL
                           KGLGTDEDTLIEILA
                                        LASRTNKEIR
                                         ASRTNKEIR
                                         ASRTNKEIR
                                         ASRTNKEIR
                                             NKEIR
                                             NKEIR
                                             NKEIR

151      160       170       180       190       200
DINRVYREELKRDLAKDITSDTSGDFRNALLSLAKGDRSEDFGVNEDLAD
DINRVYREEL
DI
DINRV
DINRVYREEL
DINRV
DINRVYREEL
DINRVYREELKRDLAKDIT
     YREELKRDLAKDITSDT
     YREELKRDLAKDITSDTSGDFRNA
          KRDLAKDITSDT
          KRDLAKDITSDTSGDFRNALL
          KRDLAKDITSDTSGDFRNALLSLA
              AKDITSDTSGDFRNALL
               KDITSDTSGDFRNALL
                  TSDTSGDFRNA
                  TSDTSGDFRNALL
                   SDTSGDFRNA
                   SDTSGDFRNALL
                   SDTSGDFRNALLSL
                   SDTSGDFRNALLSLA
                      SGDFRNALLSLA
                               SLAKGDRSEDFGV
                               SLAKGDRSEDFGVNEDLA
                               SLAKGDRSEDFGVNEDLAD
                               SLAKGDRSEDFGVNEDLAD
                               SLAKGDRSEDFGVNEDLAD
                                LAKGDRSEDFGVNEDLAD
                                  KGDRSEDFGVNEDLAD
                                  KGDRSEDFGVNEDLAD

201      210       220       230       240       250
SDARALYEAGERRKGTDVNVFNTILTTRSYPQLRRVFQKYTKYSKHDMNK
SDA
SDARA
SDARAL
SDARA
SDARA
SDARALYEA
     LYEAGERRKGTDVNVFNT
         GERRKGTDVNVFNT
         GERRKGTDVNVFNTILT
                  NVFNTILTTRSYPQLRRV
                   VFNTILTTRSYPQLRRV
                     NTILTTRSYPQLRRV
                       ILTTRSYPQL
```

```
                       ILTTRSYPQLRRV
                          TRSYPQLRRV
                          TRSYPQLRRVFQ
                                    FQKYTKYSKHDM
                                    FQKYTKYSKHDMNK
                                    FQKYTKYSKHDMNK
                                    FQKYTKYSKHDMNK
                                    FQKYTKYSKHDMNK
                                      KYTKYSKHDMNK
                                         KYSKHDMNK
                                         KYSKHDMNK
                                         KYSKHDMNK
                                            KHDMNK

251      260       270       280       290       300
VLDLELKGDIEKCLTAIVKCATSKPAFFAEKLHQAMKGVGTRHKALIRIM
V
VL
VLDL
VLDLEL
VLDLELKGDIEKC
VL
VLDL
VLDLELKGDIEKCLT
VLDLELKGDIEKC
    ELKGDIEKC
    ELKGDIEKCLTAIVKC
      KGDIEKCLTAIVKC
             LTAIVKCA
             LTAIVKCATSKPA
             LTAIVKCATSKPAFFAEKL
               AIVKCATSKPA
               AIVKCATSKPAFFAEKL
                 VKCATSKPAFFAEKL
                  KCATSKPAFFAEKL
                    ATSKPAFFAEKL
                    ATSKPAFFAEKLH
                    ATSKPAFFAEKLHQ
                    ATSKPAFFAEKLHQA
                    ATSKPAFFAEKLHQAM
                    ATSKPAFFAEKLHQAMKGV
                    ATSKPAFFAEKLHQAMKGVGT
                    ATSKPAFFAEKLHQAMKGVGTRHKA
                     TSKPAFFAEKL
                     TSKPAFFAEKLHQ
                     TSKPAFFAEKLHQAMKGV
                      SKPAFFAEKL
                        PAFFAEKLHQA
                          FFAEKLHQ
                          FFAEKLHQA
                          FFAEKLHQAM
                          FFAEKLHQAMKGV
                          FFAEKLHQAMKGVGTRHKALIRI
                          FFAEKLHQAMKGVGTRHKALIRIM
                          FFAEKLHQAMKGVGTRHKALIRIM
                                HQAMKGVGTRHKAL
                                HQAMKGVGTRHKALI
                                HQAMKGVGTRHKALIRI
                                HQAMKGVGTRHKALIRIM
                                HQAMKGVGTRHKALIRIM
                                HQAMKGVGTRHKALIRIM
                                  AMKGVGTRHKALIRIM
                                   MKGVGTRHKALIRI
```

```
                                   MKGVGTRHKALIRIM
                                   MKGVGTRHKALIRIM
                                   MKGVGTRHKALIRIM
                                     GVGTRHKALIRIM
                                       GTRHKALIRIM
                                              IRIM
                                               RIM
                                                 M
                                                 M
                                                 M
                                                 M
                                                 M

301      310       320       330       340   346
VSRSEIDMNDIKAFYQKMYGISLCQAILDETKGDYEKILVALCGGN
V
V
VS
V
VS
VSRSEIDMNDIKA
VSRSEIDMNDIKA
VSRSEIDMNDI
VSRSEIDMNDIKA
VSRSEIDMNDIKAFYQ
VSRSEIDMNDIKAFYQKM
VSRSEIDMNDIKAFYQKMYGISLC
VSRSEIDMNDI
VSRSEIDMNDIKA
VSRSEIDMNDIKAFYQ
VSRSEIDMNDIKAFYQKM
VSRSEIDMNDIKAFYQKMYGI
VSRSEIDMNDIKAFYQKMYGISLC
 SRSEIDMNDIKA
 SRSEIDMNDIKAFYQ
 SRSEIDMNDIKAFYQKM
 SRSEIDMNDIKAFYQKMYGISLC
  RSEIDMNDIKA
  RSEIDMNDIKAFYQ
    EIDMNDIKA
             FYQKMYGI
             FYQKMYGISLC
             FYQKMYGISLCQA
                KMYGISLC
                KMYGISLCQA
                        QAILDETKGDYEKI
                        QAILDETKGDYEKIL
                        QAILDETKGDYEKILV
                        QAILDETKGDYEKILVA
                        QAILDETKGDYEKILVALC
                        QAILDETKGDYEKILVALCGGN
                          ILDETKGDYEKIL
                          ILDETKGDYEKILV
                          ILDETKGDYEKILVA
                          ILDETKGDYEKILVAL
                          ILDETKGDYEKILVALC
                          ILDETKGDYEKILVALCGGN
                           LDETKGDYEKILV
                           LDETKGDYEKILVALCGGN
                            DETKGDYEKILV
                               KGDYEKILVALC
                               KGDYEKILVALCGGN
```

Supporting data 2

CLUSTAL O (1.2.4) multiple sequence alignment of ANXA1s

```
                             1         10        20        30        40        50        60
P14950|Columba livia p35     MAVVSEFLKQAWFMENLEQECIKCTQCVHGVP-----QQTNFDPSADVVALEKAMTAKGV         55
Q92040|Columba livia p37     MAMVSEFLKQAWFMEHQEQEYIKSVKGGPVVPQ----QQPNFDPSADVVALEKAMTAKGV         56
P24551|Rodentia sp.          MAMVSEFINQACYLEKQEQEYIEIVKSYKGGPAHAVSPYPSFDPSSDVAALHKGIMVNGV         60
P07150|Rattus norvegicus     MAMVSEFLKQACYIEKQEQEYVQAVKSYKGGPGSAVSPYPSFNPSSDVAALHKAIMVKGV         60
P10107|Mus musculus          MAMVSEFLKQARFLENQEQEYVQAVKSYKGGPGSAVSPYPSFNVSSDVAALHKAIMVKGV         60
Q8HZM6|Equus caballus        MSMVSAFLKQAWFIENEEQEYIKAVKGSKGGPGSAVSPYPSFNPSSDVDALHKAITVKGV         60
P19619|Sus scrofa            MAMVSEFLKQAWFIDNEEQEYIKTVKGSKGGPGSAVSPYPTFNPSSDVEASHKAITVKGV         60
P46193|Bos taurus            MAMVSEFLKQAWFIENEEQEYIKTVKGSKGGPGSAVSPYPTFNPSSDVEALHKAITVKGV         60
P14087|Cavia cutleri         MSMVSEFLKQAYFIDNQEQDYVKTVKSSKGGPGSAVSPYPSFDASSDVAALHKAITVKGV         60
P51662|Oryctolagus cuniculus MAMVSEFLKQAWFIDNEEQDYINTVKTYKGGPGSAVSPYPAFNPSSDVAALHQAIMVKGV         60
Q5REL2|Pongo abelii          MAMVSEFLKQAWFIENEEQEYVQTVKSSKGGPGSAVSPYPTFNPSSDVAALHKAIMVKGV         60
A5A6M2|Pan troglodytes       MAMVSEFLKQAWFIENEEQEYVQTVKSSKGGPGSAVSPYPTFNPSSDVAALHKAIMVKGV         60
P04083|Homo sapiens          MAMVSEFLKQAWFIENEEQEYVQTVKSSKGGPGSAVSPYPTFNPSSDVAALHKAIMVKGV         60
                             *::** *::** :::: **: :: .:     *         *: *:** * .:.: .:**

                             61        70        80        90        100       110       120
P14950|Columba livia p35     DEATIIDIMTTRTNAQRPRIKAAYHKAKGKSLEEAMKRVLKSHLEDVVVALLKTPAQFDA         115
Q92040|Columba livia p37     DEATIIDIMTKRTNAQRHRIKAAYQKAKGKSLEEAMKRVLKSHLEDVVVALLKTPAQFDA         116
P24551|Rodentia sp.          DEATILDLLTKRYNAQRHHLKAVYIQETGEPLDETLKKALTGHIQELLLAMIKTPAQFDG         120
P07150|Rattus norvegicus     DEATIIDILTKRTNAQRQQIKAAYLQETGKPLDETLKKALTGHLEEVVLAMLKTPAQFDA         120
P10107|Mus musculus          DEATIIDILTKRTNAQRQQIKAAYLQENGKPLDEVLRKALTGHLEEVVLAMLKTPAQFDA         120
Q8HZM6|Equus caballus        DEATIIEILTKRNNAQRQQIKAAYLQEKGKPLDEALKKALTGHLEDVALALLKTPARFDA         120
P19619|Sus scrofa            DEATIIEIHTKRTNAQRQQIKAAYLQEKGKPLDEALKKALTGHLEEVALALLKTPAQFDA         120
P46193|Bos taurus            DEATIIEILTKRNNAQRQQIKAAYLQEKGKPLDEVLKKALLGHLEEVVLALLKTPAQFDA         120
P14087|Cavia cutleri         DEATIIDILTKRNNAQRQQIKAAYLQEKGKPLDEALKKALTGHLEEVVLALLKTPAQLDA         120
P51662|Oryctolagus cuniculus DEATIIDILTKRNNAQRQQIKAAYLQEKGKPLDEVLKKALTGHLEEVVLALLKTPAQFDA         120
Q5REL2|Pongo abelii          DEATIIDVLTKRNNAQRQQIKAAYLQETGKPLDETLKKALTGHLEEVVLALLKTPAQFDA         120
A5A6M2|Pan troglodytes       DEATIIDILTRRNNAQRQQIKAAYLQETGKPLDETLKKALTGHLEEVVLALLKTPAQFDA         120
P04083|Homo sapiens          DEATIIDILTKRNNAQRQQIKAAYLQETGKPLDETLKKALTGHLEEVVLALLKTPAQFDA         120
                             *****::: * * **** ::**.* : .*: *:*.:::.* .*:::: :*::****::*.

                             121       130       140       150       160       170       180
P14950|Columba livia p35     EELRACMKGHGTDEDTLIEILASRNNKEIREACRYYKEVLKRDLTQDIISDTSGDFQKAL         175
Q92040|Columba livia p37     EELRACMKGLGTDEDTLIEILASRSNKEIREASRYYKEVLKRDLTQDIISDTSGHFQKAL         176
P24551|Rodentia sp.          NELRAAMKAVGTDEETLIEILWTRSNQQIREITSVYREELKKDIAKYQTSDTSGEFRDAL         180
P07150|Rattus norvegicus     DELRAAMKGLGTDEDTLIEILTTRSNQQIREITRVYREELKRDLAKDITSDTSGDFRNAL         180
P10107|Mus musculus          DELRGAMKGLGTDEDTLIEILTTRSNEQIREINRVYREELKRDLAKDITSDTSGDFRKAL         180
Q8HZM6|Equus caballus        DELRAAMKGLGTDEDTLIEILTSRTNKEIREINRVYREELKRDLAKDITSDTSGDFQKAL         180
P19619|Sus scrofa            DELRAAMKGLGTDEDTLNEILASRTNREIREINRVYKEELKRDLAKDITSDTSGDYQKAL         180
P46193|Bos taurus            EELRAAMKGLGTDEDTLNEILASRTNREIREINRVYREELKRDLAKDIASDTSGDYEKAL         180
P14087|Cavia cutleri         DELRAAMKGLGTDEDTLIEILVSRKNREIKEINRVYRDELKRDLAKDITSDTSGDFQKAL         180
P51662|Oryctolagus cuniculus DELRAAMKGLGTDEDTLIEILASRNNKEIREINRVYREELKRDLAKDIASDTSGDFQKAL         180
Q5REL2|Pongo abelii          DELRAAMKGLGTDEDTLIEILASRTNKEIRDINRVYREELKRDLAKDITSDTSGDFRNAL         180
A5A6M2|Pan troglodytes       DELRAAMKGLGTDEDTLIEILASRTNKEIRDINRVYREELKRDLAKDITSDTSGDFRNAL         180
P04083|Homo sapiens          DELRAAMKGLGTDEDTLIEILASRTNKEIRDINRVYREELKRDLAKDITSDTSGDFRNAL         180
                             :***..**. ****:** *** :*.*.:*::    *:: **:*:::   *****.:..**

                             181       190       200       210       220       230       240
P14950|Columba livia p35     VSLAKADRCENPHVNDELAEKDARALYEAGEQKKGTDINVFVTVLTARSYPHS-EVFQKY         234
Q92040|Columba livia p37     VVLAKGDRCEDPHVNDDLADNDARALYEAGEQKKGTDVNVFVTVLTARSYPHLRRVFQKY         236
P24551|Rodentia sp.          LALAKGNRCEDMSVNQDIADTDARALYQAAERRNGTDVNVFNTILTTKKYPHLRNKFQNY         240
P07150|Rattus norvegicus     LALAKGDRCEDMSVNQDLADTDARALYEAGERRKGTDVNVFNTILTTRSYPHLRKVFQNY         240
P10107|Mus musculus          LALAKGDRCQDLSVNQDLADTDARALYEAGERRKGTDVNVFTTILTSRSFPHLRRVFQNY         240
Q8HZM6|Equus caballus        LSLAKGDRSEDFGVNDDLADSDARALYEAGERRKGTDVNVFNTILTTRSYPHLRRVFQMY         240
P19619|Sus scrofa            LSLAKGDRSEDLAINDDLADTDARALYEAGERRKGTDLNVFITILTTRSYLHLRRVFQKY         240
P46193|Bos taurus            LSLAKGDRSEELAVNDDLADSDARALYEAGERRKGTDVNVFITILTTRSYPHLRRVFQKY         240
P14087|Cavia cutleri         LSLAKGDRCEDLSVNDDLADSDARALYEAGERRKGTDVNVFITILTTRSYSHLRRVFQKY         240
P51662|Oryctolagus cuniculus LSLAKGDRSEDFGVNEDLADTDARALYEAGERRKGADVNVFTTILTTRSYLHLRRVFQKY         240
Q5REL2|Pongo abelii          LSLAKGDRSEDFGVNEDLADSDARALYEAGERRKGTDVNVFNTILTTRSYPQLRRVFQKY         240
A5A6M2|Pan troglodytes       LSLAKGDRSEDFGVNEDLADSDARALYEAGERRKGTDVNVFNTILTTRSYPQLRRVFQKY         240
P04083|Homo sapiens          LSLAKGDRSEDFGVNEDLADSDARALYEAGERRKGTDVNVFNTILTTRSYPQLRRVFQKY         240
                             : ***.:*.::  :*:::*:.******:*.*:::*:*:*** *:**::.: :  . ** *

                             241       250       260       270       280       290       300
P14950|Columba livia p35     TKYSKHDMNKAVDMEMKGDIEKCLTALVKCATSKPAFFAEKLHMAMKGFGTQHRDLIRIM         294
Q92040|Columba livia p37     TKYSKHDMNKVVDMELKGDIEKCLTALVKCATSKPAFFAEKLHLAMKGFGTRHKDLIRIM         296
P24551|Rodentia sp.          RKYTEEDMKKALDIELKGQIEKCLTTIAKCGTSTPAFFAEKLYEAMKGAGTRHKTLIRIM         300
P07150|Rattus norvegicus     RKYSQHDMNKALDLELKGDIEKCLTTIVKCATSTPAFFAEKLYEAMKGAGTRHKTLIRIM         300
P10107|Mus musculus          GKYSQHDMNKALDLELKGDIEKCLTTIVKCATSTPAFFAEKLYEAMKGAGTRHKALIRIM         300
Q8HZM6|Equus caballus        TKYSKHDMNKVLDLEMKGDVENCFTAIVKCATSKPMFFAEKLHNAMKGAGTRDKILIRIM         300
P19619|Sus scrofa            SKYSKHDMNKVLDLELKGDIENCLTVVVKCATSKPMFFAEKLHQAMKGNGTRHKTLIRIM         300
P46193|Bos taurus            SKYSKHDMNKVLDLELKGDIEKCLTVIVKCATSQPMFFAEKLHQAMKGIGTRHKTLIRIM         300
P14087|Cavia cutleri         TKYSQHDMNKALDLELKGDIENCLTAIVKCATSTPAFFAEKLHLAMKGAGTRHKALIRIM         300
P51662|Oryctolagus cuniculus SKYSQHDMNKVLDLELKGDIEKCLTAIVQCATCKPAYFAEKLYQAMKGAGTRHKALIRIM         300
Q5REL2|Pongo abelii          TKYSKHDMNKVLDLELKGDIEKCLTAIVKCATSKPAFFAEKLHQAMKGVGTRHKALIRIM         300
A5A6M2|Pan troglodytes       TKYSKHDMNKVLDLELKGDIEKCLTAIVKCATSKPAFFAEKLHQAMKGVGTRHKALIRIM         300
P04083|Homo sapiens          TKYSKHDMNKVLDLELKGDIEKCLTAIVKCATSKPAFFAEKLHQAMKGVGTRHKALIRIM         300
                              **::.**:*.:*:*:**::*:*:*.:.:*.*. * :*****: **** **:.: *****

                             301       310       320       330       340
P14950|Columba livia p35     VSRHEVDMNEIKGYYKKMYGISLCQAIMDELKGGYETILVALCGSDN                      341
Q92040|Columba livia p37     VSRHEVDMNEIKCYYKKMYGISLCQAIMDDLKGDYETILVALCGSDN                      343
P24551|Rodentia sp.          VSRSEIDSDQIKVFYQKKYGVPLCQAILDETKGAYEKILVALEGGN-                      346
P07150|Rattus norvegicus     VSRSEIDMNEIKVFYQKKYGIPLCQAILDETKGDYEKILVALCGGN-                      346
P10107|Mus musculus          VSRSEIDMNEIKVFYQKKYGISLCQAILDETKGDYEKILVALCGGN-                      346
Q8HZM6|Equus caballus        VSRSEVDMNDIKACYQKLYGISLCQAILDETKGDYEKILVALCGRD-                      346
P19619|Sus scrofa            VSRSEIDMNDIKACYQKLYGISLCQAILDETKGDYEKILVALCGGD-                      346
```

```
P46193|Bos taurus             VSRSEIDMNDIKACYQKLYGISLCQAILDETKGDYEKILVALCGRD-      346
P14087|Cavia cutleri          VSRSEIDMNDIKVYYQKMYGISLCQAILDETKGDYEKILVALCGGQ-      346
P51662|Oryctolagus cuniculus  VSRSEVDMNDIKAFYQKKYGVSLCQAILDETKGDYEKILVALCGGN-      346
Q5REL2|Pongo abelii           VSRSEIDMNDIKAFYQKMYGISLCQAILDETKGDYEKILVALCGGN-      346
A5A6M2|Pan troglodytes        VSRSEIDMNDIKAFYQKMYGISLCQAILDETKGDYEKILVALCGGN-      346
P04083|Homo sapiens           VSRSEIDMNDIKAFYQKMYGISLCQAILDETKGDYEKILVALCGGN-      346
                              *** *:* ::**  *:* **: *****:*: ** **.***** * :
```

Supporting data 3

Folded ANXA1 0 sec

```
1         10        20        30        40        50
MAMVSEFLKQAWFIENEEQEYVQTVKSSKGGPGSAVSPYPTFNPSSDVAA
           WFIENEEQEYVQTVKSSKGGPGSA
                           SKGGPGSAVSPYPTFNPSSDVA

51        60        70        80        90        100
LHKAIMVKGVDEATIIDILTKRNNAQRQQIKAAYLQETGKPLDETLKKAL

101       110       120       130       140       150
TGHLEEVVLALLKTPAQFDADELRAAMKGLGTDEDTLIEILASRTNKEIR

151       160       170       180       190       200
DINRVYREELKRDLAKDITSDTSGDFRNALLSLAKGDRSEDFGVNEDLAD

201       210       220       230       240       250
SDARALYEAGERRKGTDVNVFNTILTTRSYPQLRRVFQKYTKYSKHDMNK

251       260       270       280       290       300
VLDLELKGDIEKCLTAIVKCATSKPAFFAEKLHQAMKGVGTRHKALIRIM

301       310       320       330       340   346
VSRSEIDMNDIKAFYQKMYGISLCQAILDETKGDYEKILVALCGGN
```

Folded ANXA1 15 sec

```
1         10        20        30        40        50
MAMVSEFLKQAWFIENEEQEYVQTVKSSKGGPGSAVSPYPTFNPSSDVAA
           WFIENEEQEYVQTV
                   EYVQTVKSSKGGPGSAVSPYPT
                        VKSSKGGPGSAVSPYPT
                         KSSKGGPGSAVSPYPT
                         KSSKGGPGSAVSPYPTFNPSSD
                         KSSKGGPGSAVSPYPTFNPSSDVA
                         KSSKGGPGSAVSPYPTFNPSSDVAA
                           SKGGPGSAVSPYPT
                            KGGPGSAVSPYPT
                                         FNPSSDVAA
                                         FNPSSDVAA
                                         FNPSSDVAA
                                         FNPSSDVAA

51        60        70        80        90        100
LHKAIMVKGVDEATIIDILTKRNNAQRQQIKAAYLQETGKPLDETLKKAL
L
LHKA
LHKAIMVK
LHKAIMVKGVDEAT
    IMVKGVDEATIIDI
                                AYLQETGKP
                                AYLQETGKPL
                                AYLQETGKPLD
                                AYLQETGKPLDE
                                AYLQETGKPLDET
                                AYLQETGKPLDETL
                                AYLQETGKPLDETLK
                                AYLQETGKPLDETLKKAL
                                AYLQETGKPLDETLKKAL
                                AYLQETGKPLDETLKKAL
                                   QETGKPLDETLKKAL

101       110       120       130       140       150
TGHLEEVVLALLKTPAQFDADELRAAMKGLGTDEDTLIEILASRTNKEIR
TG
TGHLEEV
TGHLEEV
       VLALLKTPAQFDADEL
       VLALLKTPAQFDADELRA
       VLALLKTPAQFDADELRAA
                         AMKGLGTDEDTLIEILA

151       160       170       180       190       200
DINRVYREELKRDLAKDITSDTSGDFRNALLSLAKGDRSEDFGVNEDLAD

201       210       220       230       240       250
SDARALYEAGERRKGTDVNVFNTILTTRSYPQLRRVFQKYTKYSKHDMNK

251       260       270       280       290       300
VLDLELKGDIEKCLTAIVKCATSKPAFFAEKLHQAMKGVGTRHKALIRIM

301       310       320       330       340   346
VSRSEIDMNDIKAFYQKMYGISLCQAILDETKGDYEKILVALCGGN
 SRSEIDMNDIKAFYQKMYGISLC
```

Folded ANXA1 30 sec

```
1        10        20        30        40        50
MAMVSEFLKQAWFIENEEQEYVQTVKSSKGGPGSAVSPYPTFNPSSDVAA
 AMVSEFLKQA
          AWFIENEEQEYVQTV
           WFIENEEQEYVQTV
                   EYVQTVKSSKGGPGSAVSPYPT
                        VKSSKGGPGSAVSPYPT
                         KSSKGGPGSAVSPYPT
                         KSSKGGPGSAVSPYPTFNPSSD
                         KSSKGGPGSAVSPYPTFNPSSDVA
                         KSSKGGPGSAVSPYPTFNPSSDVAA
                           SKGGPGSAVSPYPT
                            KGGPGSAVSPYPT
                                         FNPSSDVAA
                                         FNPSSDVAA
                                         FNPSSDVAA
                                         FNPSSDVAA
                                         FNPSSDVAA
                                         FNPSSDVAA

51       60        70        80        90        100
LHKAIMVKGVDEATIIDILTKRNNAQRQQIKAAYLQETGKPLDETLKKAL
L
LHKA
LHKAIMV
LHKAIMVK
LHKAIMVKGVDEA
LHKAIMVKGVDEAT
    IMVKGVDEA
    IMVKGVDEAT
    IMVKGVDEATI
    IMVKGVDEATII
    IMVKGVDEATIIDILTK
    IMVKGVDEATIIDILTKRN
                                AYLQETGKP
                                AYLQETGKPL
                                AYLQETGKPLD
                                AYLQETGKPLDE
                                AYLQETGKPLDET
                                AYLQETGKPLDETL
                                AYLQETGKPLDETLK
                                AYLQETGKPLDETLKKA
                                AYLQETGKPLDETLKKAL
                                AYLQETGKPLDETLKKAL
                                AYLQETGKPLDETLKKAL
                                AYLQETGKPLDETLKKAL
                                AYLQETGKPLDETLKKAL
                                 YLQETGKPLDE
                                 YLQETGKPLDETL
                                 YLQETGKPLDETLKKA
                                 YLQETGKPLDETLKKAL
                                   QETGKPLDETLKKAL
                                      GKPLDETLKKAL
                                        PLDETLKKAL
                                                 L

101      110       120       130       140       150
TGHLEEVVLALLKTPAQFDADELRAAMKGLGTDEDTLIEILASRTNKEIR
TG
TGH
TGHLE
```

```
TGHLEEV
TGHLEEV
TGHLEEV
TGHLEEV
TGHLEEV
TGHLEEV
       VLALLKTPA
       VLALLKTPAQFDA
       VLALLKTPAQFDADEL
       VLALLKTPAQFDADELR
       VLALLKTPAQFDADELRA
       VLALLKTPAQFDADELRAA
       VLALLKTPAQFDADELRAAM
                         AMKGLGTDEDTLIEILA

151      160       170       180       190       200
DINRVYREELKRDLAKDITSDTSGDFRNALLSLAKGDRSEDFGVNEDLAD

201      210       220       230       240       250
SDARALYEAGERRKGTDVNVFNTILTTRSYPQLRRVFQKYTKYSKHDMNK

251      260       270       280       290       300
VLDLELKGDIEKCLTAIVKCATSKPAFFAEKLHQAMKGVGTRHKALIRIM
                    ATSKPAFFAEKL

301      310       320       330       340   346
VSRSEIDMNDIKAFYQKMYGISLCQAILDETKGDYEKILVALCGGN
             FYQKMYGISLCQA
```

Folded ANXA1 60 sec

```
1        10        20        30        40        50
MAMVSEFLKQAWFIENEEQEYVQTVKSSKGGPGSAVSPYPTFNPSSDVAA
 AMVSEFLKQA
     EFLKQAWFIENEEQEYVQTV
          AWFIENEEQEYVQTV
           WFIENEEQ
           WFIENEEQEYVQTV
                   EYVQTVKSSKGGPGSAVSPYPT
                       TVKSSKGGPGSAVSPYPT
                        VKSSKGGPGSAVSPYPT
                        VKSSKGGPGSAVSPYPTFNPSSDVA
                         KSSKGGPGSAVSPYPT
                         KSSKGGPGSAVSPYPTFNPSSD
                         KSSKGGPGSAVSPYPTFNPSSDV
                         KSSKGGPGSAVSPYPTFNPSSDVA
                         KSSKGGPGSAVSPYPTFNPSSDVAA
                           SKGGPGSAVSPYPT
                            KGGPGSAVSPYPT
                                         FNPSSDVAA
                                         FNPSSDVAA
                                         FNPSSDVAA
                                         FNPSSDVAA
                                         FNPSSDVAA
                                         FNPSSDVAA
                                           PSSDVAA
51       60        70        80        90        100
LHKAIMVKGVDEATIIDILTKRNNAQRQQIKAAYLQETGKPLDETLKKAL
L
LHKA
LHKAIMV
LHKAIMVK
LHKAIMVKGVDEA
LHKAIMVKGVDEAT
LHKA
    IMVKGVDEA
    IMVKGVDEAT
    IMVKGVDEATI
    IMVKGVDEATII
    IMVKGVDEATIIDIL
    IMVKGVDEATIIDILT
    IMVKGVDEATIIDILTKRNNAQ
                       NAQRQQIKAAYL
                                AYLQETGKP
                                AYLQETGKPL
                                AYLQETGKPLD
                                AYLQETGKPLDE
                                AYLQETGKPLDET
                                AYLQETGKPLDETL
                                AYLQETGKPLDETLK
                                AYLQETGKPLDETLKKA
                                AYLQETGKPLDETLKKAL
                                AYLQETGKPLDETLKKAL
                                AYLQETGKPLDETLKKAL
                                AYLQETGKPLDETLKKAL
                                AYLQETGKPLDETLKKAL
                                AYLQETGKPLDETLKKAL
                                AYLQETGKPLDETLKKAL
                                 YLQETGKPLDE
                                 YLQETGKPLDET
                                 YLQETGKPLDETL
```

```
                                 YLQETGKPLDETLKKA
                                 YLQETGKPLDETLKKAL
                                   QETGKPLDET
                                   QETGKPLDETLKKAL
                                             LKKAL
                                                 L

101      110       120       130       140       150
TGHLEEVVLALLKTPAQFDADELRAAMKGLGTDEDTLIEILASRTNKEIR
T
TG
TGH
TGHL
TGHLE
TGHLEE
TGHLEEV
TG
TGHLEEV
TGHLEEV
TGHLEEV
TGHLEEV
       VLALLKTP
       VLALLKTPA
       VLALLKTPAQ
       VLALLKTPAQF
       VLALLKTPAQFD
       VLALLKTPAQFDA
       VLALLKTPAQFDAD
       VLALLKTPAQFDADE
       VLALLKTPAQFDADEL
       VLALLKTPAQFDADELR
       VLALLKTPAQFDADELRA
       VLALLKTPAQFDADELRAA
       VLALLKTPAQFDADELRAAM
       VLALLKTPAQFDADELRAAMKGLGT
            KTPAQFDADELRA
            KTPAQFDADELRAA
                       RAAMKGLGTDEDTLIEI
                         AMKGLGTDEDTLIEILA

151      160       170       180       190       200
DINRVYREELKRDLAKDITSDTSGDFRNALLSLAKGDRSEDFGVNEDLAD
                               SLAKGDRSEDFGVNEDLA
```

```
                              SLAKGDRSEDFGVNEDLADSD

201      210       220       230       240       250
SDARALYEAGERRKGTDVNVFNTILTTRSYPQLRRVFQKYTKYSKHDMNK
SDARAL
                       ILTTRSYPQLRRV

251      260       270       280       290       300
VLDLELKGDIEKCLTAIVKCATSKPAFFAEKLHQAMKGVGTRHKALIRIM
                    ATSKPAFFAEKL
                    ATSKPAFFAEKLHQAM
                     TSKPAFFAEKL

301      310       320       330       340   346
VSRSEIDMNDIKAFYQKMYGISLCQAILDETKGDYEKILVALCGGN
                        QAILDETKGDYEKILV
                          ILDETKGDYEKILV
```

Folded ANXA1 90 sec

```
1        10        20        30        40        50
MAMVSEFLKQAWFIENEEQEYVQTVKSSKGGPGSAVSPYPTFNPSSDVAA
 AMVSEFLKQ
 AMVSEFLKQA
  MVSEFLKQA
        KQAWFIENEEQEYVQTV
          AWFIENEEQEYVQTV
           WFIENEEQ
           WFIENEEQEYVQTV
           WFIENEEQEYVQTVKS
                   EYVQTVKSSKGGPGSAVSPYPT
                       TVKSSKGGPGSAVSPYPT
                        VKSSKGGPGSAVSPYPT
                        VKSSKGGPGSAVSPYPTFNPSSDVA
                         KSSKGGPGSAVSPYPT
                         KSSKGGPGSAVSPYPTFNPSSD
                         KSSKGGPGSAVSPYPTFNPSSDV
                         KSSKGGPGSAVSPYPTFNPSSDVA
                         KSSKGGPGSAVSPYPTFNPSSDVAA
                           SKGGPGSAVSPYPT
                            KGGPGSAVSPYPT
                                         FNPSSDVAA
                                         FNPSSDVAA
                                         FNPSSDVAA
                                         FNPSSDVAA
                                         FNPSSDVAA
                                         FNPSSDVAA
                                         FNPSSDVAA
                                           PSSDVAA
51       60        70        80        90        100
LHKAIMVKGVDEATIIDILTKRNNAQRQQIKAAYLQETGKPLDETLKKAL
L
LHKA
LHKAIM
LHKAIMV
LHKAIMVK
LHKAIMVKGVDEA
LHKAIMVKGVDEAT
LHKA
    IMVKGVDEA
    IMVKGVDEAT
    IMVKGVDEATI
    IMVKGVDEATII
    IMVKGVDEATIIDI
    IMVKGVDEATIIDIL
    IMVKGVDEATIIDILT
    IMVKGVDEATIIDILTK
       KGVDEATIIDILTKRNNAQRQQIKA
                   TKRNNAQRQQIKAAYLQETGKPLDE
                       NAQRQQIKAAYL
                                AYLQETGKP
                                AYLQETGKPL
                                AYLQETGKPLD
                                AYLQETGKPLDE
                                AYLQETGKPLDET
                                AYLQETGKPLDETL
                                AYLQETGKPLDETLK
                                AYLQETGKPLDETLKKA
                                AYLQETGKPLDETLKKAL
                                AYLQETGKPLDETLKKAL
```

```
                                AYLQETGKPLDETLKKAL
                                AYLQETGKPLDETLKKAL
                                AYLQETGKPLDETLKKAL
                                AYLQETGKPLDETLKKAL
                                AYLQETGKPLDETLKKAL
                                 YLQETGKPLD
                                 YLQETGKPLDE
                                 YLQETGKPLDET
                                 YLQETGKPLDETL
                                 YLQETGKPLDETLKKA
                                 YLQETGKPLDETLKKAL
                                   QETGKPLDET
                                   QETGKPLDETLKKAL
                                             LKKAL
                                                 L

101      110       120       130       140       150
TGHLEEVVLALLKTPAQFDADELRAAMKGLGTDEDTLIEILASRTNKEIR
T
TG
TGH
TGHL
TGHLE
TGHLEE
TGHLEEV
TG
TGHLEEV
TGHLEEV
TGHLEEV
TGHLEEV
TGHLEEV
       VLALLKTP
       VLALLKTPA
       VLALLKTPAQ
       VLALLKTPAQF
       VLALLKTPAQFD
       VLALLKTPAQFDA
       VLALLKTPAQFDAD
       VLALLKTPAQFDADE
       VLALLKTPAQFDADEL
       VLALLKTPAQFDADELR
       VLALLKTPAQFDADELRA
       VLALLKTPAQFDADELRAA
       VLALLKTPAQFDADELRAAM
       VLALLKTPAQFDADELRAAMKGLGT
         ALLKTPAQFDADEL
          LLKTPAQFDADEL
            KTPAQFDADELRA
            KTPAQFDADELRAA
                       RAAMKGLGTDEDTLIEI
                       RAAMKGLGTDEDTLIEIL
                         AMKGLGTDEDTLIEI
                         AMKGLGTDEDTLIEILA

151      160       170       180       190       200
DINRVYREELKRDLAKDITSDTSGDFRNALLSLAKGDRSEDFGVNEDLAD
                               SLAKGDRSEDFGVNEDLA
```

```
                              SLAKGDRSEDFGVNEDLADSD
                              SLAKGDRSEDFGVNEDLADSD

201      210       220       230       240       250
SDARALYEAGERRKGTDVNVFNTILTTRSYPQLRRVFQKYTKYSKHDMNK
SDARA
SDARAL
                       ILTTRSYPQLRRV
                          TRSYPQLRRV
                                    FQKYTKYSKHDMNK
                                    FQKYTKYSKHDMNK

251      260       270       280       290       300
VLDLELKGDIEKCLTAIVKCATSKPAFFAEKLHQAMKGVGTRHKALIRIM
VLDL
VLDLEL
                    ATSKPAFFAEKL
                    ATSKPAFFAEKLHQ
                    ATSKPAFFAEKLHQA
                     TSKPAFFAEKL
                      SKPAFFAEKL
                           FAEKLHQA
                                HQAMKGVGTRHKALIRIM

301      310       320       330       340   346
VSRSEIDMNDIKAFYQKMYGISLCQAILDETKGDYEKILVALCGGN
VSRSEIDMNDIKA
VSRSEIDMNDIKAFYQKM
             FYQKMYGISLCQA
                        QAILDETKGDYEKILV
                          ILDETKGDYEKILV
                          ILDETKGDYEKILVALC
```

Folded ANXA1 120 sec

```
1        10        20        30        40        50
MAMVSEFLKQAWFIENEEQEYVQTVKSSKGGPGSAVSPYPTFNPSSDVAA
 AMVSEFLKQ
 AMVSEFLKQA
  MVSEFLKQA
        KQAWFIENEEQEYVQTV
          AWFIENEEQEYVQTV
           WFIENEEQ
           WFIENEEQEYVQT
           WFIENEEQEYVQTV
           WFIENEEQEYVQTVKS
           WFIENEEQEYVQTVKSS
              ENEEQEYVQTV
                   EYVQTVKSSKGGPGSAVSPYPT
                       TVKSSKGGPGSAVSPYPT
                        VKSSKGGPGSAVSPYPT
                        VKSSKGGPGSAVSPYPTFNPSSD
                        VKSSKGGPGSAVSPYPTFNPSSDVA
                         KSSKGGPGSAVSPYPT
                         KSSKGGPGSAVSPYPTFNPSS
                         KSSKGGPGSAVSPYPTFNPSSD
                         KSSKGGPGSAVSPYPTFNPSSDV
                         KSSKGGPGSAVSPYPTFNPSSDVA
                         KSSKGGPGSAVSPYPTFNPSSDVAA
                           SKGGPGSAVSPYPT
                            KGGPGSAVSPYPT
                                       PTFNPSSDVAA
                                         FNPSSDVAA
                                         FNPSSDVAA
                                         FNPSSDVAA
                                         FNPSSDVAA
                                         FNPSSDVAA
                                         FNPSSDVAA
                                         FNPSSDVAA
                                         FNPSSDVAA
                                          NPSSDVAA
                                           PSSDVAA

51       60        70        80        90        100
LHKAIMVKGVDEATIIDILTKRNNAQRQQIKAAYLQETGKPLDETLKKAL
LHKA
L
LHKA
LHKAIM
LHKAIMV
LHKAIMVK
LHKAIMVKGVDEA
LHKAIMVKGVDEAT
LHKA
LHKA
    IMVKGVDEA
    IMVKGVDEATI
    IMVKGVDEATIIDI
    IMVKGVDEATIIDIL
    IMVKGVDEATIIDILT
    IMVKGVDEATIIDILTK
    IMVKGVDEATIIDILTKR
    IMVKGVDEATIIDILTKRN
    IMVKGVDEATIIDILTKRNNAQ
       KGVDEATIIDILTKRNNAQRQQIKA
```

```
                   TKRNNAQRQQIKAAYLQET
                   TKRNNAQRQQIKAAYLQETGKPLDE
                       NAQRQQIKAAYL
                       NAQRQQIKAAYLQET
                              KAAYLQET
                                AYLQETGKP
                                AYLQETGKPL
                                AYLQETGKPLD
                                AYLQETGKPLDE
                                AYLQETGKPLDET
                                AYLQETGKPLDETL
                                AYLQETGKPLDETLK
                                AYLQETGKPLDETLKK
                                AYLQETGKPLDETLKKA
                                AYLQETGKPLDETLKKAL
                                AYLQETGKPLDETLKKAL
                                AYLQETGKPLDETLKKAL
                                AYLQETGKPLDETLKKAL
                                AYLQETGKPLDETLKKAL
                                AYLQETGKPLDETLKKAL
                                AYLQETGKPLDETLKKAL
                                AYLQETGKPLDETLKKAL
                                 YLQETGKPLD
                                 YLQETGKPLDE
                                 YLQETGKPLDET
                                 YLQETGKPLDETL
                                 YLQETGKPLDETLKKA
                                 YLQETGKPLDETLKKAL
                                 YLQETGKPLDETLKKAL
                                   QETGKPLDET
                                   QETGKPLDETLKKAL
                                      GKPLDETLKKAL
                                        PLDETLKKAL
                                             LKKAL
                                                 L
                                                 L
101      110       120       130       140       150
TGHLEEVVLALLKTPAQFDADELRAAMKGLGTDEDTLIEILASRTNKEIR
T
TG
TGH
TGHL
TGHLE
TGHLEE
TGHLEEV
TG
TGHLEEV
TGHLEEV
TGHLEEV
TGHLEEV
TGHLEEV
TGHLEEV
TGHLEEVVLA
       VLALLKTP
       VLALLKTPA
       VLALLKTPAQ
       VLALLKTPAQF
       VLALLKTPAQFD
       VLALLKTPAQFDA
       VLALLKTPAQFDAD
       VLALLKTPAQFDADE
       VLALLKTPAQFDADEL
       VLALLKTPAQFDADELR
```

```
       VLALLKTPAQFDADELRA
       VLALLKTPAQFDADELRAA
       VLALLKTPAQFDADELRAAM
       VLALLKTPAQFDADELRAAMKG
       VLALLKTPAQFDADELRAAMKGLGT
         ALLKTPAQFDADEL
         ALLKTPAQFDADELRA
         ALLKTPAQFDADELRAA
          LLKTPAQFDADEL
          LLKTPAQFDADELRA
            KTPAQFDADEL
            KTPAQFDADELRA
            KTPAQFDADELRAA
                       RAAMKGLGTDEDTLIEI
                       RAAMKGLGTDEDTLIEIL
                         AMKGLGTDEDTL
                         AMKGLGTDEDTLIEI
                         AMKGLGTDEDTLIEIL
                         AMKGLGTDEDTLIEILA
                         AMKGLGTDEDTLIEILASRT
                           KGLGTDEDTLIEILA

151      160       170       180       190       200
DINRVYREELKRDLAKDITSDTSGDFRNALLSLAKGDRSEDFGVNEDLAD
                   SDTSGDFRNALL
                               SLAKGDRSEDFGV
                               SLAKGDRSEDFGVNEDLA
                               SLAKGDRSEDFGVNEDLAD
                               SLAKGDRSEDFGVNEDLAD

201      210       220       230       240       250
SDARALYEAGERRKGTDVNVFNTILTTRSYPQLRRVFQKYTKYSKHDMNK
SDARA
SDARAL
                   VFNTILTTRSYPQLRRV
                       ILTTRSYPQLRRV
                          TRSYPQLRRV
                                    FQKYTKYSKHDMNK
                                    FQKYTKYSKHDMNK

251      260       270       280       290       300
VLDLELKGDIEKCLTAIVKCATSKPAFFAEKLHQAMKGVGTRHKALIRIM
V
VLDL
                    ATSKPAFFAEKL
                    ATSKPAFFAEKLHQ
                    ATSKPAFFAEKLHQA
                    ATSKPAFFAEKLHQAM
                    ATSKPAFFAEKLHQAMKGV
                     TSKPAFFAEKL
                      SKPAFFAEKL
                          FFAEKLHQA
                          FFAEKLHQAMKGV
                                HQAMKGVGTRHKALIRIM

301      310       320       330       340   346
VSRSEIDMNDIKAFYQKMYGISLCQAILDETKGDYEKILVALCGGN
VSRSEIDMNDIKA
                        QAILDETKGDYEKILV
                        QAILDETKGDYEKILVALC
                        QAILDETKGDYEKILVALCGGN
                          ILDETKGDYEKILV
                          ILDETKGDYEKILVA
```

ILDETKGDYEKILVALC
ILDETKGDYEKILVALCGGN

Folded ANXA1 300 sec

```
1        10        20        30        40        50
MAMVSEFLKQAWFIENEEQEYVQTVKSSKGGPGSAVSPYPTFNPSSDVAA
 AMVSEFLKQ
 AMVSEFLKQA
 AMVSEFLKQAWFIENEEQEYVQTV
  MVSEFLKQ
  MVSEFLKQA
        KQAWFIENEEQEYVQTV
          AWFIENEEQ
          AWFIENEEQEYVQTV
           WFIENEEQ
           WFIENEEQEYV
           WFIENEEQEYVQT
           WFIENEEQEYVQTV
           WFIENEEQEYVQTVKS
           WFIENEEQEYVQTVKSS
           WFIENEEQEYVQTVKSSKGGPGSA
              ENEEQEYVQTV
                   EYVQTVKSSKGGPGSAVSPYPT
                       TVKSSKGGPGSAVSPYPT
                        VKSSKGGPGSAVSPYPT
                        VKSSKGGPGSAVSPYPTFNPSSD
                        VKSSKGGPGSAVSPYPTFNPSSDVA
                         KSSKGGPGSAVSPYPT
                         KSSKGGPGSAVSPYPTFNPSS
                         KSSKGGPGSAVSPYPTFNPSSD
                         KSSKGGPGSAVSPYPTFNPSSDV
                         KSSKGGPGSAVSPYPTFNPSSDVA
                         KSSKGGPGSAVSPYPTFNPSSDVAA
                          SSKGGPGSAVSPYPT
                           SKGGPGSAVSPYPT
                            KGGPGSAVSPYPT
                                       PTFNPSSDVAA
                                        TFNPSSDVAA
                                         FNPSSDVAA
                                         FNPSSDVAA
                                         FNPSSDVAA
                                         FNPSSDVAA
                                         FNPSSDVAA
                                         FNPSSDVAA
                                         FNPSSDVAA
                                         FNPSSDVAA
                                          NPSSDVAA
                                           PSSDVAA
                                            SSDVAA
                                                 A

51       60        70        80        90        100
LHKAIMVKGVDEATIIDILTKRNNAQRQQIKAAYLQETGKPLDETLKKAL
LHKA
LHKA
L
LHKA
LHKAIM
LHKAIMV
LHKAIMVK
LHKAIMVKGVDEA
LHKAIMVKGVDEAT
LHKA
```

```
LHKA
LHKA
LHKAIMVKGVDEATIIDIL
    IMVKGVDEA
    IMVKGVDEATI
    IMVKGVDEATIIDI
    IMVKGVDEATIIDIL
    IMVKGVDEATIIDILT
    IMVKGVDEATIIDILTK
    IMVKGVDEATIIDILTKR
    IMVKGVDEATIIDILTKRN
    IMVKGVDEATIIDILTKRNNAQ
      VKGVDEATIIDIL
       KGVDEATIIDIL
       KGVDEATIIDILTKRN
       KGVDEATIIDILTKRNNAQRQQIKA
                   TKRNNAQRQQIKAAYL
                   TKRNNAQRQQIKAAYLQET
                   TKRNNAQRQQIKAAYLQETGKPLDE
                      NNAQRQQIKAAYL
                      NNAQRQQIKAAYLQET
                       NAQRQQIKAAYL
                       NAQRQQIKAAYLQET
                              KAAYLQET
                                AYLQETGKP
                                AYLQETGKPL
                                AYLQETGKPLD
                                AYLQETGKPLDE
                                AYLQETGKPLDET
                                AYLQETGKPLDETL
                                AYLQETGKPLDETLK
                                AYLQETGKPLDETLKKA
                                AYLQETGKPLDETLKKAL
                                AYLQETGKPLDETLKKAL
                                AYLQETGKPLDETLKKAL
                                AYLQETGKPLDETLKKAL
                                AYLQETGKPLDETLKKAL
                                AYLQETGKPLDETLKKAL
                                AYLQETGKPLDETLKKAL
                                AYLQETGKPLDETLKKAL
                                 YLQETGKPLD
                                 YLQETGKPLDE
                                 YLQETGKPLDET
                                 YLQETGKPLDETL
                                 YLQETGKPLDETLKKA
                                 YLQETGKPLDETLKKAL
                                 YLQETGKPLDETLKKAL
                                 YLQETGKPLDETLKKAL
                                   QETGKPLDET
                                   QETGKPLDETLKKAL
                                   QETGKPLDETLKKAL
                                      GKPLDETLKKAL
                                             LKKAL
                                                AL
                                                 L
                                                 L

101      110       120       130       140       150
TGHLEEVVLALLKTPAQFDADELRAAMKGLGTDEDTLIEILASRTNKEIR
T
TG
TGH
TGHL
```

```
TGHLE
TGHLEE
TGHLEEV
T
TG
TGHLEEV
T
TGHLEEV
TGHLEEV
TGHLEEV
TGHLEEV
TGHLEEV
TGHLEEVVLA
       VLALLKTP
       VLALLKTPA
       VLALLKTPAQ
       VLALLKTPAQF
       VLALLKTPAQFD
       VLALLKTPAQFDA
       VLALLKTPAQFDAD
       VLALLKTPAQFDADE
       VLALLKTPAQFDADEL
       VLALLKTPAQFDADELR
       VLALLKTPAQFDADELRA
       VLALLKTPAQFDADELRAA
       VLALLKTPAQFDADELRAAM
       VLALLKTPAQFDADELRAAMK
       VLALLKTPAQFDADELRAAMKG
       VLALLKTPAQFDADELRAAMKGLGT
        LALLKTPAQFDADEL
        LALLKTPAQFDADELRA
         ALLKTPAQFDADEL
         ALLKTPAQFDADELRA
         ALLKTPAQFDADELRAA
          LLKTPAQFDADEL
          LLKTPAQFDADELRA
          LLKTPAQFDADELRAA
           LKTPAQFDADELRA
            KTPAQFDADEL
            KTPAQFDADELRA
            KTPAQFDADELRAA
                QFDADELRA
                QFDADELRAA
                       RAAMKGLGTDEDTLIEI
                       RAAMKGLGTDEDTLIEIL
                       RAAMKGLGTDEDTLIEILA
                       RAAMKGLGTDEDTLIEILASRT
                         AMKGLGTDEDTL
                         AMKGLGTDEDTLIE
                         AMKGLGTDEDTLIEI
                         AMKGLGTDEDTLIEIL
                         AMKGLGTDEDTLIEILA
                         AMKGLGTDEDTLIEILASRT
                                        LASRTNKEIR
                                         ASRTNKEIR
                                             NKEIR

151      160       170       180       190       200
DINRVYREELKRDLAKDITSDTSGDFRNALLSLAKGDRSEDFGVNEDLAD
DINRVYREEL
DINRVYREEL
DINRVYREEL
               KDITSDTSGDFRNALL
```

```
                  TSDTSGDFRNALL
                  TSDTSGDFRNALLSLA
                   SDTSGDFRNALL
                   SDTSGDFRNALLSLA
                      SGDFRNALLSLA
                               SLAKGDRSEDFGV
                               SLAKGDRSEDFGVNEDLA
                               SLAKGDRSEDFGVNEDLAD
                               SLAKGDRSEDFGVNEDLAD

201      210       220       230       240       250
SDARALYEAGERRKGTDVNVFNTILTTRSYPQLRRVFQKYTKYSKHDMNK
SDARA
SDARAL
     LYEAGERRKGTDVNVFNT
     LYEAGERRKGTDVNVFNTILT
         GERRKGTDVNVFNTILT
                  NVFNTILTTRSYPQLRRV
                   VFNTILTTRSYPQLRRV
                       ILTTRSYPQLRRV
                          TRSYPQLRRV
                                    FQKYTKYSKHDMNK
                                    FQKYTKYSKHDMNK
                                    FQKYTKYSKHDMNK

251      260       270       280       290       300
VLDLELKGDIEKCLTAIVKCATSKPAFFAEKLHQAMKGVGTRHKALIRIM
V
VL
VLDL
             LTAIVKCA
                    ATSKPAFFAEKL
                    ATSKPAFFAEKLHQ
                    ATSKPAFFAEKLHQA
                    ATSKPAFFAEKLHQAM
                    ATSKPAFFAEKLHQAMKGV
                    ATSKPAFFAEKLHQAMKGVGT
                    ATSKPAFFAEKLHQAMKGVGTRHKA
                     TSKPAFFAEKL
                     TSKPAFFAEKLHQAMKGV
                      SKPAFFAEKL
                          FFAEKLHQ
                          FFAEKLHQA
                          FFAEKLHQAMKGV
                                HQAMKGVGTRHKAL
                                HQAMKGVGTRHKALI
                                HQAMKGVGTRHKALIRIM
                                HQAMKGVGTRHKALIRIM
                                  AMKGVGTRHKALIRIM
                                   MKGVGTRHKALIRIM
                                       GTRHKALIRIM
                                                 M
                                                 M

301      310       320       330       340   346
VSRSEIDMNDIKAFYQKMYGISLCQAILDETKGDYEKILVALCGGN
V
VSRSEIDMNDIKA
VSRSEIDMNDIKAFYQ
VSRSEIDMNDI
VSRSEIDMNDIKA
VSRSEIDMNDIKAFYQ
VSRSEIDMNDIKAFYQKM
```

```
VSRSEIDMNDIKAFYQKMYGI
VSRSEIDMNDIKAFYQKMYGISLC
 SRSEIDMNDIKA
 SRSEIDMNDIKAFYQ
  RSEIDMNDIKA
             FYQKMYGI
             FYQKMYGISLC
             FYQKMYGISLCQA
                        QAILDETKGDYEKI
                        QAILDETKGDYEKILV
                        QAILDETKGDYEKILVA
                        QAILDETKGDYEKILVALC
                        QAILDETKGDYEKILVALCGGN
                          ILDETKGDYEKILV
                          ILDETKGDYEKILVA
                          ILDETKGDYEKILVAL
                          ILDETKGDYEKILVALC
                          ILDETKGDYEKILVALCGGN
                           LDETKGDYEKILV
                           LDETKGDYEKILVALCGGN
                               KGDYEKILVALC
                               KGDYEKILVALCGGN
```

Folded ANXA1 600 sec

```
1        10        20        30        40        50
MAMVSEFLKQAWFIENEEQEYVQTVKSSKGGPGSAVSPYPTFNPSSDVAA
 AMVSEFLKQ
 AMVSEFLKQA
 AMVSEFLKQAWFIENEEQEYVQTV
  MVSEFLKQ
VSEFLKQAWFIENEEQEYVQTV
 SEFLKQAWFIENEEQEYVQTV
  EFLKQAWFIENEEQEYVQT
  EFLKQAWFIENEEQEYVQTV
  EFLKQAWFIENEEQEYVQTVKS
     KQAWFIENEEQEYVQTV
       AWFIENEEQ
       AWFIENEEQEYVQT
       AWFIENEEQEYVQTV
       AWFIENEEQEYVQTVKS
        WFIENEEQ
        WFIENEEQEYV
        WFIENEEQEYVQ
        WFIENEEQEYVQT
        WFIENEEQEYVQTV
        WFIENEEQEYVQTVKS
        WFIENEEQEYVQTVKSS
        WFIENEEQEYVQTVKSSKGGPGSA
           ENEEQEYVQTV
                EYVQTVKSSKGGPGSAVSPYPT
                   QTVKSSKGGPGSAVSPYPT
                    TVKSSKGGPGSAVSPYPT
                     VKSSKGGPGSAVSPYPT
                     VKSSKGGPGSAVSPYPTFNPSSD
                     VKSSKGGPGSAVSPYPTFNPSSDVA
                      KSSKGGPGSAVSPYPT
                      KSSKGGPGSAVSPYPTFNPSS
                      KSSKGGPGSAVSPYPTFNPSSD
                      KSSKGGPGSAVSPYPTFNPSSDV
                      KSSKGGPGSAVSPYPTFNPSSDVA
                      KSSKGGPGSAVSPYPTFNPSSDVAA
                       SSKGGPGSAVSPYPT
                        SKGGPGSAVSPYPT
                         KGGPGSAVSPYPT
                                    PTFNPSSDVAA
                                     TFNPSSDVAA
                                      FNPSSDVAA
                                      FNPSSDVAA
                                      FNPSSDVAA
                                      FNPSSDVAA
                                      FNPSSDVAA
                                      FNPSSDVAA
                                      FNPSSDVAA
                                      FNPSSDVAA
                                      FNPSSDVAA
                                       NPSSDVAA
                                        PSSDVAA
                                         SSDVAA
                                              A

51       60        70        80        90        100
LHKAIMVKGVDEATIIDILTKRNNAQRQQIKAAYLQETGKPLDETLKKAL
LHKA
LHKA
L
```

```
LH
LHKA
LHKAIM
LHKAIMV
LHKAIMVK
LHKAIMVKGVDEA
LHKAIMVKGVDEAT
LHKA
LHKA
LHKA
LHKAIMVKGVDEATIIDIL
    IMVKGVDEA
    IMVKGVDEATI
    IMVKGVDEATIIDI
    IMVKGVDEATIIDIL
    IMVKGVDEATIIDILT
    IMVKGVDEATIIDILTK
    IMVKGVDEATIIDILTKR
    IMVKGVDEATIIDILTKRN
    IMVKGVDEATIIDILTKRNNA
    IMVKGVDEATIIDILTKRNNAQ
      VKGVDEATIIDIL
       KGVDEATIIDIL
       KGVDEATIIDILTKRN
       KGVDEATIIDILTKRNNAQRQQIKA
                   TKRNNAQRQQIKAAYL
                   TKRNNAQRQQIKAAYLQET
                   TKRNNAQRQQIKAAYLQETGKPLDE
                      NNAQRQQIKAAYL
                      NNAQRQQIKAAYLQET
                       NAQRQQIKAAYL
                       NAQRQQIKAAYLQET
                              KAAYLQET
                                AYLQETGKP
                                AYLQETGKPL
                                AYLQETGKPLD
                                AYLQETGKPLDE
                                AYLQETGKPLDET
                                AYLQETGKPLDETL
                                AYLQETGKPLDETLK
                                AYLQETGKPLDETLKKA
                                AYLQETGKPLDETLKKAL
                                AYLQETGKPLDETLKKAL
                                AYLQETGKPLDETLKKAL
                                AYLQETGKPLDETLKKAL
                                AYLQETGKPLDETLKKAL
                                AYLQETGKPLDETLKKAL
                                AYLQETGKPLDETLKKAL
                                AYLQETGKPLDETLKKAL
                                 YLQETGKPLD
                                 YLQETGKPLDE
                                 YLQETGKPLDET
                                 YLQETGKPLDETL
                                 YLQETGKPLDETLKKA
                                 YLQETGKPLDETLKKAL
                                 YLQETGKPLDETLKKAL
                                 YLQETGKPLDETLKKAL
                                   QETGKPLDET
                                   QETGKPLDETLKKAL
                                   QETGKPLDETLKKAL
                                    ETGKPLDETLKKAL
                                      GKPLDETLKKAL
                                        PLDETLKKAL
```

```
                                             LKKAL
                                              KKAL
                                                AL
                                                 L
                                                 L

101      110       120       130       140       150
TGHLEEVVLALLKTPAQFDADELRAAMKGLGTDEDTLIEILASRTNKEIR
T
TG
TGH
TGHL
TGHLE
TGHLEE
TGHLEEV
T
TG
TGHLEEV
T
TGHLEEV
TGHLEEV
TGHLEEV
TGHLEEV
TGHLEEV
TGHLEEV
TGHLEEV
TGHLEEV
TGHLEEVVLA
       VLALLKTP
       VLALLKTPA
       VLALLKTPAQ
       VLALLKTPAQF
       VLALLKTPAQFD
       VLALLKTPAQFDA
       VLALLKTPAQFDAD
       VLALLKTPAQFDADE
       VLALLKTPAQFDADEL
       VLALLKTPAQFDADELR
       VLALLKTPAQFDADELRA
       VLALLKTPAQFDADELRAA
       VLALLKTPAQFDADELRAAM
       VLALLKTPAQFDADELRAAMK
       VLALLKTPAQFDADELRAAMKG
       VLALLKTPAQFDADELRAAMKGLGT
        LALLKTPAQFDADEL
        LALLKTPAQFDADELRA
        LALLKTPAQFDADELRAA
         ALLKTPAQFDADEL
         ALLKTPAQFDADELRA
         ALLKTPAQFDADELRAA
         ALLKTPAQFDADELRAAM
          LLKTPAQFDADEL
          LLKTPAQFDADELRA
          LLKTPAQFDADELRAA
           LKTPAQFDADELRA
            KTPAQFDADEL
            KTPAQFDADELRA
            KTPAQFDADELRAA
            KTPAQFDADELRAAM
                QFDADELRA
                QFDADELRAA
                QFDADELRAAMKGLGTDEDTLIEI
                       RAAMKGLGTDE
```

```
                       RAAMKGLGTDEDTLIEI
                       RAAMKGLGTDEDTLIEIL
                       RAAMKGLGTDEDTLIEILA
                       RAAMKGLGTDEDTLIEILASRT
                         AMKGLGTDEDTL
                         AMKGLGTDEDTLIE
                         AMKGLGTDEDTLIEI
                         AMKGLGTDEDTLIEIL
                         AMKGLGTDEDTLIEILA
                         AMKGLGTDEDTLIEILASRT
                          MKGLGTDEDTLIEI
                          MKGLGTDEDTLIEIL
                           KGLGTDEDTLIEILA
                                        LASRTNKEIR
                                        LASRTNKEIR
                                         ASRTNKEIR
                                         ASRTNKEIR
                                         ASRTNKEIR
                                             NKEIR
                                             NKEIR

151      160       170       180       190       200
DINRVYREELKRDLAKDITSDTSGDFRNALLSLAKGDRSEDFGVNEDLAD
DI
DINRVYREEL
DI
DIN
DINRVYREEL
DINRV
DINRVYREEL
          KRDLAKDITSDTSGDFRNALL
               KDITSDTSGDFRNALL
                  TSDTSGDFRNA
                  TSDTSGDFRNALL
                  TSDTSGDFRNALLSLA
                   SDTSGDFRNA
                   SDTSGDFRNALL
                   SDTSGDFRNALLSL
                   SDTSGDFRNALLSLA
                      SGDFRNALLSLA
                               SLAKGDRSEDFGV
                               SLAKGDRSEDFGVNEDLA
                               SLAKGDRSEDFGVNEDLAD
                               SLAKGDRSEDFGVNEDLAD
                                  KGDRSEDFGVNEDLAD
                                  KGDRSEDFGVNEDLAD

201      210       220       230       240       250
SDARALYEAGERRKGTDVNVFNTILTTRSYPQLRRVFQKYTKYSKHDMNK
SDARA
SDARAL
SDARA
SDARALYEA
     LYEAGERRKGTDVNVFNT
     LYEAGERRKGTDVNVFNTILT
         GERRKGTDVNVFNTILT
                  NVFNTILT
                  NVFNTILTTRSYPQLRRV
                   VFNTILTTRSYPQLRRV
                       ILTTRSYPQL
                       ILTTRSYPQLRRV
                          TRSYPQLRRV
                          TRSYPQLRRVFQ
```

```
                                    FQKYTKYSKHDM
                                    FQKYTKYSKHDMNK
                                    FQKYTKYSKHDMNK
                                    FQKYTKYSKHDMNK
                                    FQKYTKYSKHDMNK
                                         KYSKHDMNK

251      260       270       280       290       300
VLDLELKGDIEKCLTAIVKCATSKPAFFAEKLHQAMKGVGTRHKALIRIM
V
VL
VLDL
VLDLEL
VLDL
    ELKGDIEKC
                    ATSKPAFFAEKL
                    ATSKPAFFAEKLH
                    ATSKPAFFAEKLHQ
                    ATSKPAFFAEKLHQA
                    ATSKPAFFAEKLHQAM
                    ATSKPAFFAEKLHQAMKGV
                    ATSKPAFFAEKLHQAMKGVGT
                    ATSKPAFFAEKLHQAMKGVGTRHKA
                     TSKPAFFAEKL
                     TSKPAFFAEKLHQAMKGV
                          FFAEKLHQ
                          FFAEKLHQA
                          FFAEKLHQAM
                          FFAEKLHQAMKGV
                          FFAEKLHQAMKGVGTRHKALIRIM
                                HQAMKGVGTRHKAL
                                HQAMKGVGTRHKALI
                                HQAMKGVGTRHKALIRI
                                HQAMKGVGTRHKALIRIM
                                HQAMKGVGTRHKALIRIM
                                  AMKGVGTRHKALIRIM
                                   MKGVGTRHKALIRIM
                                     GVGTRHKALIRIM
                                       GTRHKALIRIM
                                              IRIM
                                                 M
                                                 M
                                                 M

301      310       320       330       340   346
VSRSEIDMNDIKAFYQKMYGISLCQAILDETKGDYEKILVALCGGN
V
VSRSEIDMNDIKA
VSRSEIDMNDIKA
VSRSEIDMNDIKAFYQ
VSRSEIDMNDIKAFYQKM
VSRSEIDMNDI
VSRSEIDMNDIKA
VSRSEIDMNDIKAFYQ
VSRSEIDMNDIKAFYQKM
VSRSEIDMNDIKAFYQKMYGI
VSRSEIDMNDIKAFYQKMYGISLC
 SRSEIDMNDIKA
 SRSEIDMNDIKAFYQ
  RSEIDMNDIKA
  RSEIDMNDIKAFYQ
    EIDMNDIKA
             FYQKMYGI
```

```
FYQKMYGISLC
FYQKMYGISLCQA
           QAILDETKGDYEKI
           QAILDETKGDYEKILV
           QAILDETKGDYEKILVA
           QAILDETKGDYEKILVALC
           QAILDETKGDYEKILVALCGGN
             ILDETKGDYEKIL
             ILDETKGDYEKILV
             ILDETKGDYEKILVA
             ILDETKGDYEKILVAL
             ILDETKGDYEKILVALC
             ILDETKGDYEKILVALCGGN
              LDETKGDYEKILV
              LDETKGDYEKILVALCGGN
               DETKGDYEKILV
                  KGDYEKILVALC
                  KGDYEKILVALCGGN
```

Folded ANXA1 1200 sec

```
1        10        20        30        40        50
MAMVSEFLKQAWFIENEEQEYVQTVKSSKGGPGSAVSPYPTFNPSSDVAA
MAMVSEFLKQA
 AMVSEFLKQ
 AMVSEFLKQA
  MVSEFLKQ
  MVSEFLKQA
   VSEFLKQA
   VSEFLKQAWFIENEEQEYVQTV
    SEFLKQAWFIENEEQEYVQTV
     EFLKQAWFIENEEQEYVQT
     EFLKQAWFIENEEQEYVQTV
     EFLKQAWFIENEEQEYVQTVKS
        KQAWFIENEEQEYVQTV
          AWFIENEEQ
          AWFIENEEQEYVQT
          AWFIENEEQEYVQTV
          AWFIENEEQEYVQTVKS
           WFIENEEQ
           WFIENEEQEYV
           WFIENEEQEYVQ
           WFIENEEQEYVQT
           WFIENEEQEYVQTV
           WFIENEEQEYVQTVKS
           WFIENEEQEYVQTVKSS
           WFIENEEQEYVQTVKSSKGGPGSA
              ENEEQEYVQTV
                   EYVQTVKSSKGGPGSAVSPYPT
                      QTVKSSKGGPGSAVSPYPT
                       TVKSSKGGPGSAVSPYPT
                        VKSSKGGPGSAVSPYPT
                        VKSSKGGPGSAVSPYPTFNPSSD
                        VKSSKGGPGSAVSPYPTFNPSSDVA
                         KSSKGGPGSAVSPYPT
                         KSSKGGPGSAVSPYPTFNPSS
                         KSSKGGPGSAVSPYPTFNPSSD
                         KSSKGGPGSAVSPYPTFNPSSDV
                         KSSKGGPGSAVSPYPTFNPSSDVA
                         KSSKGGPGSAVSPYPTFNPSSDVAA
                          SSKGGPGSAVSPYPT
                           SKGGPGSAVSPYPT
                            KGGPGSAVSPYPT
                                       PTFNPSSDVAA
                                        TFNPSSDVAA
                                         FNPSSDVAA
                                         FNPSSDVAA
                                         FNPSSDVAA
                                         FNPSSDVAA
                                         FNPSSDVAA
                                         FNPSSDVAA
                                         FNPSSDVAA
                                         FNPSSDVAA
                                         FNPSSDVAA
                                          NPSSDVAA
                                           PSSDVAA
                                            SSDVAA
                                                 A


51       60        70        80        90        100
LHKAIMVKGVDEATIIDILTKRNNAQRQQIKAAYLQETGKPLDETLKKAL
```

```
LHKA
LHKA
L
LH
LHKA
LHKAIM
LHKAIMV
LHKAIMVK
LHKAIMVKGVDEA
LHKAIMVKGVDEAT
LHKA
LHKA
LHKA
LHKAIMVKGVDEATIIDIL
    IMVKGVDEA
    IMVKGVDEATII
    IMVKGVDEATIIDI
    IMVKGVDEATIIDIL
    IMVKGVDEATIIDILT
    IMVKGVDEATIIDILTK
    IMVKGVDEATIIDILTKR
    IMVKGVDEATIIDILTKRN
    IMVKGVDEATIIDILTKRNNA
    IMVKGVDEATIIDILTKRNNAQ
     MVKGVDEATIIDIL
      VKGVDEATIIDI
      VKGVDEATIIDIL
       KGVDEATIIDIL
       KGVDEATIIDILTKRN
       KGVDEATIIDILTKRNNAQRQQIKA
                   TKRNNAQRQQIKAAYL
                   TKRNNAQRQQIKAAYLQET
                   TKRNNAQRQQIKAAYLQETGKPLDE
                      NNAQRQQIKAAYL
                      NNAQRQQIKAAYLQET
                       NAQRQQIKAAYL
                       NAQRQQIKAAYLQET
                              KAAYLQET
                                AYLQETGKP
                                AYLQETGKPL
                                AYLQETGKPLD
                                AYLQETGKPLDE
                                AYLQETGKPLDET
                                AYLQETGKPLDETL
                                AYLQETGKPLDETLK
                                AYLQETGKPLDETLKKA
                                AYLQETGKPLDETLKKAL
                                AYLQETGKPLDETLKKAL
                                AYLQETGKPLDETLKKAL
                                AYLQETGKPLDETLKKAL
                                AYLQETGKPLDETLKKAL
                                AYLQETGKPLDETLKKAL
                                AYLQETGKPLDETLKKAL
                                AYLQETGKPLDETLKKAL
                                 YLQETGKPLD
                                 YLQETGKPLDE
                                 YLQETGKPLDET
                                 YLQETGKPLDETL
                                 YLQETGKPLDETLKKA
                                 YLQETGKPLDETLKKAL
                                 YLQETGKPLDETLKKAL
                                 YLQETGKPLDETLKKAL
                                   QETGKPLDET
```

```
                                   QETGKPLDETLKKAL
                                   QETGKPLDETLKKAL
                                    ETGKPLDETLKKAL
                                      GKPLDETLKKAL
                                        PLDETLKKAL
                                             LKKAL
                                              KKAL
                                                AL
                                                 L
                                                 L


101      110       120       130       140       150
TGHLEEVVLALLKTPAQFDADELRAAMKGLGTDEDTLIEILASRTNKEIR
T
TG
TGH
TGHL
TGHLE
TGHLEE
TGHLEEV
T
TG
TGHLEEV
T
TGHLEEV
TGHLEEV
TGHLEEV
TGHLEEV
TGHLEEV
TGHLEEV
TGHLEEV
TGHLEEV
TGHLEEVVLA
       VLALLKTP
       VLALLKTPA
       VLALLKTPAQ
       VLALLKTPAQF
       VLALLKTPAQFD
       VLALLKTPAQFDA
       VLALLKTPAQFDAD
       VLALLKTPAQFDADE
       VLALLKTPAQFDADEL
       VLALLKTPAQFDADELR
       VLALLKTPAQFDADELRA
       VLALLKTPAQFDADELRAA
       VLALLKTPAQFDADELRAAM
       VLALLKTPAQFDADELRAAMK
       VLALLKTPAQFDADELRAAMKG
       VLALLKTPAQFDADELRAAMKGLGT
        LALLKTPAQFDADEL
        LALLKTPAQFDADELRA
        LALLKTPAQFDADELRAA
         ALLKTPAQFDADEL
         ALLKTPAQFDADELRA
         ALLKTPAQFDADELRAA
         ALLKTPAQFDADELRAAM
          LLKTPAQFDADEL
          LLKTPAQFDADELRA
          LLKTPAQFDADELRAA
          LLKTPAQFDADELRAAM
           LKTPAQFDADELRA
           LKTPAQFDADELRAA
```

```
            KTPAQFDADEL
            KTPAQFDADELRA
            KTPAQFDADELRAA
            KTPAQFDADELRAAM
                QFDADELRA
                QFDADELRAA
                QFDADELRAAMKGLGTDEDTLIEI
                QFDADELRAAMKGLGTDEDTLIEIL
                       RAAMKGLGTDE
                       RAAMKGLGTDEDTLIEI
                       RAAMKGLGTDEDTLIEIL
                       RAAMKGLGTDEDTLIEILA
                       RAAMKGLGTDEDTLIEILASRT
                         AMKGLGTDEDTL
                         AMKGLGTDEDTLIE
                         AMKGLGTDEDTLIEI
                         AMKGLGTDEDTLIEIL
                         AMKGLGTDEDTLIEILA
                         AMKGLGTDEDTLIEILASRT
                          MKGLGTDEDTLIEI
                          MKGLGTDEDTLIEIL
                          MKGLGTDEDTLIEILASRT
                           KGLGTDEDTLIEILA
                                        LASRTNKEIR
                                        LASRTNKEIR
                                         ASRTNKEIR
                                         ASRTNKEIR
                                         ASRTNKEIR
                                             NKEIR
                                             NKEIR
                                             NKEIR

151      160       170       180       190       200
DINRVYREELKRDLAKDITSDTSGDFRNALLSLAKGDRSEDFGVNEDLAD
DI
DINRVYREEL
DI
DIN
DINRV
DINRVYREEL
DINRV
DINRVYREEL
DINRVYREELKRDLAKDIT
     YREELKRDLAKDITSDT
          KRDLAKDITSDT
          KRDLAKDITSDTSGDFRNALL
          KRDLAKDITSDTSGDFRNALLSLA
               KDITSDTSGDFRNALL
                  TSDTSGDFRNA
                  TSDTSGDFRNALL
                  TSDTSGDFRNALLSLA
                   SDTSGDFRNA
                   SDTSGDFRNALL
                   SDTSGDFRNALLSL
                   SDTSGDFRNALLSLA
                      SGDFRNALLSL
                      SGDFRNALLSLA
                               SLAKGDRSEDFGV
                               SLAKGDRSEDFGVNEDLA
                               SLAKGDRSEDFGVNEDLAD
                               SLAKGDRSEDFGVNEDLAD
                                  KGDRSEDFGVNEDLAD
                                  KGDRSEDFGVNEDLAD
```

```
                                                AD


201      210       220       230       240       250
SDARALYEAGERRKGTDVNVFNTILTTRSYPQLRRVFQKYTKYSKHDMNK
SDA
SDARA
SDARAL
SDARA
SDARALYEA
SDARALYEA
     LYEAGERRKGTDVNVFNT
     LYEAGERRKGTDVNVFNTILT
         GERRKGTDVNVFNTILT
                  NVFNTILT
                  NVFNTILTTRSYPQLRRV
                   VFNTILTTRSYPQLRRV
                       ILTTRSYPQL
                       ILTTRSYPQLRRV
                          TRSYPQLRRV
                          TRSYPQLRRVFQ
                                    FQKYTKYSKHDM
                                    FQKYTKYSKHDMNK
                                    FQKYTKYSKHDMNK
                                    FQKYTKYSKHDMNK
                                    FQKYTKYSKHDMNK
                                      KYTKYSKHDMNK
                                         KYSKHDMNK
                                         KYSKHDMNK
                                         KYSKHDMNK
                                         KYSKHDMNK
                                             KHDMNK


251      260       270       280       290       300
VLDLELKGDIEKCLTAIVKCATSKPAFFAEKLHQAMKGVGTRHKALIRIM
V
VL
VLDL
VLDLEL
VLDLELKGDIEKC
VL
VLDL
VLDLELKGDIEKC
VLDLELKGDIEKCLT
VLDLELKGDIEKC
    ELKGDIEKC
    ELKGDIEKCLTAIVKC
      KGDIEKCLTAIVKC
             LTAIVKCATSKPA
               AIVKCATSKPA
               AIVKCATSKPAFFAEKL
                 VKCATSKPAFFAEKL
                  KCATSKPAFFAEKL
                    ATSKPAFFAEKL
                    ATSKPAFFAEKLH
                    ATSKPAFFAEKLHQ
                    ATSKPAFFAEKLHQA
                    ATSKPAFFAEKLHQAM
                    ATSKPAFFAEKLHQAMKGV
                    ATSKPAFFAEKLHQAMKGVGT
                    ATSKPAFFAEKLHQAMKGVGTRHKA
                     TSKPAFFAEKL
```

```
                     TSKPAFFAEKLHQ
                     TSKPAFFAEKLHQAMKGV
                      SKPAFFAEKL
                          FFAEKLHQ
                          FFAEKLHQA
                          FFAEKLHQAM
                          FFAEKLHQAMKGV
                          FFAEKLHQAMKGVGTRHKALIRI
                          FFAEKLHQAMKGVGTRHKALIRIM
                          FFAEKLHQAMKGVGTRHKALIRIM
                                HQAMKGVGTRHKAL
                                HQAMKGVGTRHKALI
                                HQAMKGVGTRHKALIRI
                                HQAMKGVGTRHKALIRIM
                                HQAMKGVGTRHKALIRIM
                                HQAMKGVGTRHKALIRIM
                                  AMKGVGTRHKALIRIM
                                   MKGVGTRHKALIRIM
                                   MKGVGTRHKALIRIM
                                     GVGTRHKALIRIM
                                       GTRHKALIRIM
                                              IRIM
                                               RIM
                                                 M
                                                 M
                                                 M
                                                 M
                                                 M

301      310       320       330       340   346
VSRSEIDMNDIKAFYQKMYGISLCQAILDETKGDYEKILVALCGGN
V
V
VS
VS
VSRSEIDMNDIKA
VSRSEIDMNDIKA
VSRSEIDMNDI
VSRSEIDMNDIKA
VSRSEIDMNDIKAFYQ
VSRSEIDMNDIKAFYQKM
VSRSEIDMNDIKAFYQKMYGISLC
VSRSEIDMNDI
VSRSEIDMNDIKA
VSRSEIDMNDIKAFY
VSRSEIDMNDIKAFYQ
VSRSEIDMNDIKAFYQKM
VSRSEIDMNDIKAFYQKMYGI
VSRSEIDMNDIKAFYQKMYGISLC
 SRSEIDMNDIKA
 SRSEIDMNDIKAFYQ
 SRSEIDMNDIKAFYQKM
 SRSEIDMNDIKAFYQKMYGISLC
  RSEIDMNDIKA
  RSEIDMNDIKAFYQ
    EIDMNDIKA
             FYQKMYGI
             FYQKMYGISLC
             FYQKMYGISLCQA
                KMYGISLCQA
                        QAILDETKGDYEKI
                        QAILDETKGDYEKIL
```

```
QAILDETKGDYEKILV
QAILDETKGDYEKILVA
QAILDETKGDYEKILVALC
QAILDETKGDYEKILVALCGGN
  ILDETKGDYEKIL
  ILDETKGDYEKILV
  ILDETKGDYEKILVA
  ILDETKGDYEKILVAL
  ILDETKGDYEKILVALC
  ILDETKGDYEKILVALCGGN
   LDETKGDYEKILV
   LDETKGDYEKILVALC
   LDETKGDYEKILVALCGGN
    DETKGDYEKILV
       KGDYEKILVALC
       KGDYEKILVALCGGN
```

Folded ANXA1 2400 sec

```
1        10        20        30        40        50
MAMVSEFLKQAWFIENEEQEYVQTVKSSKGGPGSAVSPYPTFNPSSDVAA
 AMVSEFLKQ
 AMVSEFLKQA
  MVSEFLKQ
  MVSEFLKQA
   VSEFLKQA
   VSEFLKQAWFIENEEQEYVQTV
    SEFLKQAWFIENEEQEYVQTV
     EFLKQAWFIENEEQEYVQT
     EFLKQAWFIENEEQEYVQTV
     EFLKQAWFIENEEQEYVQTVKS
        KQAWFIENEEQEYVQTV
          AWFIENEEQ
          AWFIENEEQEYVQT
          AWFIENEEQEYVQTV
          AWFIENEEQEYVQTVKS
           WFIENEEQ
           WFIENEEQEYV
           WFIENEEQEYVQ
           WFIENEEQEYVQT
           WFIENEEQEYVQTV
           WFIENEEQEYVQTVKS
           WFIENEEQEYVQTVKSS
           WFIENEEQEYVQTVKSSKGGPGSA
              ENEEQEYVQTV
                   EYVQTVKSSKGGPGSAVSPYPT
                      QTVKSSKGGPGSAVSPYPT
                       TVKSSKGGPGSAVSPYPT
                        VKSSKGGPGSAVSPYPT
                        VKSSKGGPGSAVSPYPTFNPSSD
                        VKSSKGGPGSAVSPYPTFNPSSDVA
                         KSSKGGPGSAVSPYPT
                         KSSKGGPGSAVSPYPTFNPSS
                         KSSKGGPGSAVSPYPTFNPSSD
                         KSSKGGPGSAVSPYPTFNPSSDV
                         KSSKGGPGSAVSPYPTFNPSSDVA
                         KSSKGGPGSAVSPYPTFNPSSDVAA
                          SSKGGPGSAVSPYPT
                           SKGGPGSAVSPYPT
                            KGGPGSAVSPYPT
                                       PTFNPSSDVAA
                                        TFNPSSDVAA
                                         FNPSSDVAA
                                         FNPSSDVAA
                                         FNPSSDVAA
                                         FNPSSDVAA
                                         FNPSSDVAA
                                         FNPSSDVAA
                                         FNPSSDVAA
                                         FNPSSDVAA
                                         FNPSSDVAA
                                          NPSSDVAA
                                           PSSDVAA
                                            SSDVAA
                                                 A


51       60        70        80        90        100
LHKAIMVKGVDEATIIDILTKRNNAQRQQIKAAYLQETGKPLDETLKKAL
LHKA
```

```
LHKA
L
LH
LHKA
LHKAIM
LHKAIMV
LHKAIMVK
LHKAIMVKGVDEA
LHKAIMVKGVDEAT
LHKA
LHKA
LHKA
LHKAIMVKGVDEATIIDIL
    IMVKGVDEA
    IMVKGVDEATI
    IMVKGVDEATII
    IMVKGVDEATIIDI
    IMVKGVDEATIIDIL
    IMVKGVDEATIIDILT
    IMVKGVDEATIIDILTK
    IMVKGVDEATIIDILTKR
    IMVKGVDEATIIDILTKRN
    IMVKGVDEATIIDILTKRNNA
    IMVKGVDEATIIDILTKRNNAQ
     MVKGVDEATIIDIL
      VKGVDEATIIDI
      VKGVDEATIIDIL
      VKGVDEATIIDILT
       KGVDEATIIDIL
       KGVDEATIIDILTKRN
       KGVDEATIIDILTKRNNAQRQQIKA
                   TKRNNAQRQQIKAAYL
                   TKRNNAQRQQIKAAYLQET
                   TKRNNAQRQQIKAAYLQETGKPLDE
                    KRNNAQRQQIKAAYL
                      NNAQRQQIKAAYL
                      NNAQRQQIKAAYLQET
                       NAQRQQIKAAYL
                       NAQRQQIKAAYLQET
                              KAAYLQET
                                AYLQETGKP
                                AYLQETGKPL
                                AYLQETGKPLD
                                AYLQETGKPLDE
                                AYLQETGKPLDET
                                AYLQETGKPLDETL
                                AYLQETGKPLDETLK
                                AYLQETGKPLDETLKKA
                                AYLQETGKPLDETLKKAL
                                AYLQETGKPLDETLKKAL
                                AYLQETGKPLDETLKKAL
                                AYLQETGKPLDETLKKAL
                                AYLQETGKPLDETLKKAL
                                AYLQETGKPLDETLKKAL
                                AYLQETGKPLDETLKKAL
                                AYLQETGKPLDETLKKAL
                                 YLQETGKPLD
                                 YLQETGKPLDE
                                 YLQETGKPLDET
                                 YLQETGKPLDETL
                                 YLQETGKPLDETLKKA
                                 YLQETGKPLDETLKKAL
                                 YLQETGKPLDETLKKAL
```

```
                                   QETGKPLDET
                                   QETGKPLDETLKKAL
                                   QETGKPLDETLKKAL
                                    ETGKPLDETLKKAL
                                      GKPLDETLKKA
                                      GKPLDETLKKAL
                                        PLDETLKKAL
                                             LKKAL
                                              KKAL
                                                AL
                                                 L
                                                 L


101      110       120       130       140       150
TGHLEEVVLALLKTPAQFDADELRAAMKGLGTDEDTLIEILASRTNKEIR
T
TG
TGH
TGHL
TGHLE
TGHLEE
TGHLEEV
T
TGHLEEV
T
TGHLEEV
TGHLEEV
TGHLEEV
TGHLEEV
TGHLEEV
TGHLEEV
TGHLEEV
TGHLEEV
TGHLEEVVLA
       VLALLKTP
       VLALLKTPA
       VLALLKTPAQ
       VLALLKTPAQF
       VLALLKTPAQFD
       VLALLKTPAQFDA
       VLALLKTPAQFDAD
       VLALLKTPAQFDADE
       VLALLKTPAQFDADEL
       VLALLKTPAQFDADELR
       VLALLKTPAQFDADELRA
       VLALLKTPAQFDADELRAA
       VLALLKTPAQFDADELRAAM
       VLALLKTPAQFDADELRAAMK
       VLALLKTPAQFDADELRAAMKG
       VLALLKTPAQFDADELRAAMKGLGT
        LALLKTPAQFDADEL
        LALLKTPAQFDADELRA
        LALLKTPAQFDADELRAA
         ALLKTPAQFDADEL
         ALLKTPAQFDADELRA
         ALLKTPAQFDADELRAA
         ALLKTPAQFDADELRAAM
          LLKTPAQFDADEL
          LLKTPAQFDADELRA
          LLKTPAQFDADELRAA
          LLKTPAQFDADELRAAM
           LKTPAQFDADELRA
```

```
           LKTPAQFDADELRAA
            KTPAQFDADEL
            KTPAQFDADELRA
            KTPAQFDADELRAA
            KTPAQFDADELRAAM
                QFDADELRA
                QFDADELRAA
                QFDADELRAAMKGLGTDEDTLIEI
                QFDADELRAAMKGLGTDEDTLIEIL
                       RAAMKGLGTDE
                       RAAMKGLGTDEDTLIEI
                       RAAMKGLGTDEDTLIEIL
                       RAAMKGLGTDEDTLIEILA
                       RAAMKGLGTDEDTLIEILASRT
                         AMKGLGTDED
                         AMKGLGTDEDTL
                         AMKGLGTDEDTLIE
                         AMKGLGTDEDTLIEI
                         AMKGLGTDEDTLIEIL
                         AMKGLGTDEDTLIEILA
                         AMKGLGTDEDTLIEILASRT
                          MKGLGTDEDTLIEI
                          MKGLGTDEDTLIEIL
                          MKGLGTDEDTLIEILASRT
                           KGLGTDEDTLIEI
                           KGLGTDEDTLIEIL
                           KGLGTDEDTLIEILA
                                        LASRTNKEIR
                                        LASRTNKEIR
                                         ASRTNKEIR
                                         ASRTNKEIR
                                         ASRTNKEIR
                                         ASRTNKEIR
                                             NKEIR
                                             NKEIR
                                             NKEIR


151      160       170       180       190       200
DINRVYREELKRDLAKDITSDTSGDFRNALLSLAKGDRSEDFGVNEDLAD
DI
DINRVYREEL
DI
DIN
DINRV
DINRVYREEL
DINRV
DINRVYREEL
DINRVYREELKRDLAKDIT
     YREELKRDLAKDITSDT
          KRDLAKDITSDT
          KRDLAKDITSDTSGDFRNALL
          KRDLAKDITSDTSGDFRNALLSLA
               KDITSDTSGDFRNALL
                  TSDTSGDFRNA
                  TSDTSGDFRNALL
                  TSDTSGDFRNALLSLA
                   SDTSGDFRNA
                   SDTSGDFRNALL
                   SDTSGDFRNALLSL
                   SDTSGDFRNALLSLA
                      SGDFRNALLSL
                      SGDFRNALLSLA
```

```
                               SLAKGDRSEDFGV
                               SLAKGDRSEDFGVNEDLA
                               SLAKGDRSEDFGVNEDLAD
                               SLAKGDRSEDFGVNEDLAD
                               SLAKGDRSEDFGVNEDLAD
                                LAKGDRSEDFGVNEDLAD
                                  KGDRSEDFGVNEDLAD
                                  KGDRSEDFGVNEDLAD
                                                AD

201      210       220       230       240       250
SDARALYEAGERRKGTDVNVFNTILTTRSYPQLRRVFQKYTKYSKHDMNK
SDA
SDARA
SDARAL
SDARA
SDARA
SDARALYEA
SDARALYEA
     LYEAGERRKGTDVNVFNT
     LYEAGERRKGTDVNVFNTILT
         GERRKGTDVNVFNTILT
                  NVFNTILT
                  NVFNTILTTRSYPQLRRV
                   VFNTILTTRSYPQLRRV
                       ILTTRSYPQL
                       ILTTRSYPQLRRV
                          TRSYPQLRRV
                          TRSYPQLRRVFQ
                                    FQKYTKYSKHDM
                                    FQKYTKYSKHDMNK
                                    FQKYTKYSKHDMNK
                                    FQKYTKYSKHDMNK
                                    FQKYTKYSKHDMNK
                                    FQKYTKYSKHDMNK
                                      KYTKYSKHDMNK
                                         KYSKHDMNK
                                         KYSKHDMNK
                                         KYSKHDMNK
                                         KYSKHDMNK
                                             KHDMNK

251      260       270       280       290       300
VLDLELKGDIEKCLTAIVKCATSKPAFFAEKLHQAMKGVGTRHKALIRIM
V
VL
VLDL
VLDLEL
VL
VLDL
VLDLELKGDIEKC
VLDLELKGDIEKCLT
VLDLELKGDIEKC
    ELKGDIEKC
    ELKGDIEKCLTAIVKC
             LTAIVKCA
             LTAIVKCATSKPA
             LTAIVKCATSKPAFFAEKL
               AIVKCATSKPA
               AIVKCATSKPAFFAEKL
                 VKCATSKPAFFAEKL
```

```
                    ATSKPAFFAEKL
                    ATSKPAFFAEKLH
                    ATSKPAFFAEKLHQ
                    ATSKPAFFAEKLHQA
                    ATSKPAFFAEKLHQAM
                    ATSKPAFFAEKLHQAMKGV
                    ATSKPAFFAEKLHQAMKGVGT
                    ATSKPAFFAEKLHQAMKGVGTRHKA
                     TSKPAFFAEKL
                     TSKPAFFAEKLHQ
                     TSKPAFFAEKLHQAMKGV
                      SKPAFFAEKL
                          FFAEKLHQ
                          FFAEKLHQA
                          FFAEKLHQAM
                          FFAEKLHQAMKGV
                          FFAEKLHQAMKGVGTRHKALIRI
                          FFAEKLHQAMKGVGTRHKALIRIM
                          FFAEKLHQAMKGVGTRHKALIRIM
                                HQAMKGVGTRHKAL
                                HQAMKGVGTRHKALI
                                HQAMKGVGTRHKALIRI
                                HQAMKGVGTRHKALIRIM
                                HQAMKGVGTRHKALIRIM
                                HQAMKGVGTRHKALIRIM
                                  AMKGVGTRHKALIRIM
                                   MKGVGTRHKALIRIM
                                     GVGTRHKALIRIM
                                       GTRHKALIRIM
                                              IRIM
                                               RIM
                                                 M
                                                 M
                                                 M
                                                 M
                                                 M

301      310       320       330       340   346
VSRSEIDMNDIKAFYQKMYGISLCQAILDETKGDYEKILVALCGGN
V
V
VS
V
VS
VSRSEIDMNDIKA
VSRSEIDMNDIKA
VSRSEIDMNDI
VSRSEIDMNDIKA
VSRSEIDMNDIKAFYQ
VSRSEIDMNDIKAFYQKM
VSRSEIDMNDIKAFYQKMYGISLC
VSRSEIDMNDI
VSRSEIDMNDIKA
VSRSEIDMNDIKAFY
VSRSEIDMNDIKAFYQ
VSRSEIDMNDIKAFYQKM
VSRSEIDMNDIKAFYQKMYGI
VSRSEIDMNDIKAFYQKMYGISLC
 SRSEIDMNDIKA
 SRSEIDMNDIKAFYQ
 SRSEIDMNDIKAFYQKM
 SRSEIDMNDIKAFYQKMYGISLC
  RSEIDMNDIKA
```

```
RSEIDMNDIKAFYQ
  EIDMNDIKA
           FYQKMYGI
           FYQKMYGISLC
           FYQKMYGISLCQA
              KMYGISLCQA
                      QAILDETKGDYEKI
                      QAILDETKGDYEKIL
                      QAILDETKGDYEKILV
                      QAILDETKGDYEKILVA
                      QAILDETKGDYEKILVALC
                      QAILDETKGDYEKILVALCGGN
                        ILDETKGDYEKIL
                        ILDETKGDYEKILV
                        ILDETKGDYEKILVA
                        ILDETKGDYEKILVAL
                        ILDETKGDYEKILVALC
                        ILDETKGDYEKILVALCGGN
                         LDETKGDYEKILV
                         LDETKGDYEKILVALC
                         LDETKGDYEKILVALCGGN
                          DETKGDYEKILV
                             KGDYEKILVALC
                             KGDYEKILVALCGGN
```

Folded ANXA1 3600 sec

```
1        10        20        30        40        50
MAMVSEFLKQAWFIENEEQEYVQTVKSSKGGPGSAVSPYPTFNPSSDVAA
MAMVSEFLKQA
 AMVSEFLKQ
 AMVSEFLKQA
  MVSEFLKQ
  MVSEFLKQA
   VSEFLKQA
    SEFLKQAWFIENEEQEYVQTV
     EFLKQAWFIENEEQEYVQT
     EFLKQAWFIENEEQEYVQTV
     EFLKQAWFIENEEQEYVQTVKS
        KQAWFIENEEQEYVQTV
          AWFIENEEQ
          AWFIENEEQEYVQT
          AWFIENEEQEYVQTV
          AWFIENEEQEYVQTVKS
           WFIENEEQ
           WFIENEEQEYV
           WFIENEEQEYVQ
           WFIENEEQEYVQT
           WFIENEEQEYVQTV
           WFIENEEQEYVQTVKS
           WFIENEEQEYVQTVKSS
           WFIENEEQEYVQTVKSSKGGPGSA
              ENEEQEYVQTV
                   EYVQTVKSSKGGPGSAVSPYPT
                      QTVKSSKGGPGSAVSPYPT
                       TVKSSKGGPGSAVSPYPT
                        VKSSKGGPGSAVSPYPT
                        VKSSKGGPGSAVSPYPTFNPSSD
                        VKSSKGGPGSAVSPYPTFNPSSDVA
                         KSSKGGPGSAVSPYPT
                         KSSKGGPGSAVSPYPTFNPSS
                         KSSKGGPGSAVSPYPTFNPSSD
                         KSSKGGPGSAVSPYPTFNPSSDV
                         KSSKGGPGSAVSPYPTFNPSSDVA
                         KSSKGGPGSAVSPYPTFNPSSDVAA
                          SSKGGPGSAVSPYPT
                           SKGGPGSAVSPYPT
                            KGGPGSAVSPYPT
                                       PTFNPSSDVAA
                                        TFNPSSDVAA
                                         FNPSSDVAA
                                         FNPSSDVAA
                                         FNPSSDVAA
                                         FNPSSDVAA
                                         FNPSSDVAA
                                         FNPSSDVAA
                                         FNPSSDVAA
                                         FNPSSDVAA
                                          NPSSDVAA
                                           PSSDVAA
                                            SSDVAA
                                                 A


51       60        70        80        90        100
LHKAIMVKGVDEATIIDILTKRNNAQRQQIKAAYLQETGKPLDETLKKAL
LHKA
```

```
LHKA
L
LHKA
LHKAIM
LHKAIMV
LHKAIMVK
LHKAIMVKGVDEA
LHKA
LHKA
LHKA
LHKAIMVKGVDEATIIDIL
    IMVKGVDEA
    IMVKGVDEATI
    IMVKGVDEATII
    IMVKGVDEATIIDI
    IMVKGVDEATIIDIL
    IMVKGVDEATIIDILT
    IMVKGVDEATIIDILTK
    IMVKGVDEATIIDILTKR
    IMVKGVDEATIIDILTKRN
    IMVKGVDEATIIDILTKRNNA
    IMVKGVDEATIIDILTKRNNAQ
     MVKGVDEATIIDIL
      VKGVDEATIIDI
      VKGVDEATIIDIL
      VKGVDEATIIDILT
      VKGVDEATIIDILTKRN
       KGVDEATIIDIL
       KGVDEATIIDILT
       KGVDEATIIDILTKRN
       KGVDEATIIDILTKRNNAQRQQIKA
                   TKRNNAQRQQIKAAYL
                   TKRNNAQRQQIKAAYLQET
                   TKRNNAQRQQIKAAYLQETGKPLDE
                    KRNNAQRQQIKAAYL
                      NNAQRQQIKAAYL
                      NNAQRQQIKAAYLQET
                       NAQRQQIKAAYL
                       NAQRQQIKAAYLQET
                              KAAYLQET
                                AYLQETGKP
                                AYLQETGKPL
                                AYLQETGKPLD
                                AYLQETGKPLDE
                                AYLQETGKPLDET
                                AYLQETGKPLDETL
                                AYLQETGKPLDETLK
                                AYLQETGKPLDETLKKA
                                AYLQETGKPLDETLKKAL
                                AYLQETGKPLDETLKKAL
                                AYLQETGKPLDETLKKAL
                                AYLQETGKPLDETLKKAL
                                AYLQETGKPLDETLKKAL
                                AYLQETGKPLDETLKKAL
                                AYLQETGKPLDETLKKAL
                                AYLQETGKPLDETLKKAL
                                 YLQETGKPLDE
                                 YLQETGKPLDET
                                 YLQETGKPLDETLKKA
                                 YLQETGKPLDETLKKAL
                                 YLQETGKPLDETLKKAL
                                   QETGKPLDET
                                   QETGKPLDETLKKAL
```

```
                                   QETGKPLDETLKKAL
                                    ETGKPLDETLKKAL
                                      GKPLDETLKKA
                                      GKPLDETLKKAL
                                        PLDETLKKAL
                                             LKKAL
                                              KKAL
                                                AL
                                                 L
                                                 L


101      110       120       130       140       150
TGHLEEVVLALLKTPAQFDADELRAAMKGLGTDEDTLIEILASRTNKEIR
T
TG
TGH
TGHL
TGHLE
TGHLEE
TGHLEEV
T
TGHLEEV
T
TGHLEEV
TGHLEEV
TGHLEEV
TGHLEEV
TGHLEEV
TGHLEEV
TGHLEEV
TGHLEEV
TGHLEEVVLA
       VLALLKTP
       VLALLKTPA
       VLALLKTPAQ
       VLALLKTPAQF
       VLALLKTPAQFD
       VLALLKTPAQFDA
       VLALLKTPAQFDAD
       VLALLKTPAQFDADE
       VLALLKTPAQFDADEL
       VLALLKTPAQFDADELR
       VLALLKTPAQFDADELRA
       VLALLKTPAQFDADELRAA
       VLALLKTPAQFDADELRAAM
       VLALLKTPAQFDADELRAAMK
       VLALLKTPAQFDADELRAAMKG
       VLALLKTPAQFDADELRAAMKGLGT
        LALLKTPAQFDADEL
        LALLKTPAQFDADELRA
        LALLKTPAQFDADELRAA
         ALLKTPAQFDADEL
         ALLKTPAQFDADELRA
         ALLKTPAQFDADELRAA
         ALLKTPAQFDADELRAAM
          LLKTPAQFDADEL
          LLKTPAQFDADELRA
          LLKTPAQFDADELRAA
          LLKTPAQFDADELRAAM
           LKTPAQFDADEL
           LKTPAQFDADELRA
           LKTPAQFDADELRAA
```

```
            KTPAQFDADEL
            KTPAQFDADELRA
            KTPAQFDADELRAA
                QFDADELRA
                QFDADELRAA
                QFDADELRAAMKGLGTDEDTLIEIL
                       RAAMKGLGTDE
                       RAAMKGLGTDEDTLIEI
                       RAAMKGLGTDEDTLIEIL
                       RAAMKGLGTDEDTLIEILA
                       RAAMKGLGTDEDTLIEILASRT
                         AMKGLGTDED
                         AMKGLGTDEDTL
                         AMKGLGTDEDTLIE
                         AMKGLGTDEDTLIEI
                         AMKGLGTDEDTLIEIL
                         AMKGLGTDEDTLIEILA
                         AMKGLGTDEDTLIEILASRT
                          MKGLGTDEDTLIEI
                          MKGLGTDEDTLIEIL
                          MKGLGTDEDTLIEILASRT
                           KGLGTDEDTLIEI
                           KGLGTDEDTLIEILA
                                        LASRTNKEIR
                                        LASRTNKEIR
                                        LASRTNKEIR
                                         ASRTNKEIR
                                         ASRTNKEIR
                                         ASRTNKEIR
                                         ASRTNKEIR
                                             NKEIR
                                             NKEIR
                                             NKEIR


151      160       170       180       190       200
DINRVYREELKRDLAKDITSDTSGDFRNALLSLAKGDRSEDFGVNEDLAD
DI
DINRV
DINRVYREEL
DI
DIN
DINRV
DINRVYREEL
DINRV
DINRVYREEL
DINRVYREELKRDLAKDIT
     YREELKRDLAKDITSDT
          KRDLAKDITSDT
          KRDLAKDITSDTSGDFRNALL
          KRDLAKDITSDTSGDFRNALLSLA
              AKDITSDTSGDFRNALL
               KDITSDTSGDFRNALL
                  TSDTSGDFRNA
                  TSDTSGDFRNALL
                   SDTSGDFRNA
                   SDTSGDFRNALL
                   SDTSGDFRNALLSL
                   SDTSGDFRNALLSLA
                      SGDFRNALLSL
                      SGDFRNALLSLA
                               SLAKGDRSEDFGV
                               SLAKGDRSEDFGVNEDLA
```

```
                               SLAKGDRSEDFGVNEDLAD
                               SLAKGDRSEDFGVNEDLAD
                               SLAKGDRSEDFGVNEDLAD
                                LAKGDRSEDFGVNEDLAD
                                  KGDRSEDFGVNEDLAD
                                  KGDRSEDFGVNEDLAD
                                                AD


201      210       220       230       240       250
SDARALYEAGERRKGTDVNVFNTILTTRSYPQLRRVFQKYTKYSKHDMNK
SDA
SDARA
SDARAL
SDARA
SDARA
SDARALYEA
SDARALYEA
     LYEAGERRKGTDVNVFNT
     LYEAGERRKGTDVNVFNTILT
         GERRKGTDVNVFNT
         GERRKGTDVNVFNTILT
                  NVFNTILT
                  NVFNTILTTRSYPQLRRV
                   VFNTILTTRSYPQLRRV
                       ILTTRSYPQL
                       ILTTRSYPQLRRV
                          TRSYPQLRRV
                          TRSYPQLRRVFQ
                                    FQKYTKYSKHDM
                                    FQKYTKYSKHDMNK
                                    FQKYTKYSKHDMNK
                                    FQKYTKYSKHDMNK
                                    FQKYTKYSKHDMNK
                                    FQKYTKYSKHDMNK
                                      KYTKYSKHDMNK
                                         KYSKHDMNK
                                         KYSKHDMNK
                                         KYSKHDMNK
                                         KYSKHDMNK
                                             KHDMNK


251      260       270       280       290       300
VLDLELKGDIEKCLTAIVKCATSKPAFFAEKLHQAMKGVGTRHKALIRIM
V
VL
VLDL
VLDLEL
VLDLELKGDIEKC
VL
VLDL
VLDLELKGDIEKC
VLDLELKGDIEKCLT
VLDLELKGDIEKC
    ELKGDIEKC
    ELKGDIEKCLTAIVKC
      KGDIEKCLTAIVKC
             LTAIVKCATSKPA
               AIVKCATSKPA
               AIVKCATSKPAFFAEKL
                 VKCATSKPAFFAEKL
                    ATSKPAFFAEKL
```

```
                    ATSKPAFFAEKLH
                    ATSKPAFFAEKLHQ
                    ATSKPAFFAEKLHQA
                    ATSKPAFFAEKLHQAM
                    ATSKPAFFAEKLHQAMKGV
                    ATSKPAFFAEKLHQAMKGVGT
                    ATSKPAFFAEKLHQAMKGVGTRHKA
                     TSKPAFFAEKL
                     TSKPAFFAEKLHQ
                     TSKPAFFAEKLHQAMKGV
                      SKPAFFAEKL
                          FFAEKLHQ
                          FFAEKLHQA
                          FFAEKLHQAM
                          FFAEKLHQAMKGV
                          FFAEKLHQAMKGVGTRHKALIRI
                          FFAEKLHQAMKGVGTRHKALIRIM
                          FFAEKLHQAMKGVGTRHKALIRIM
                                HQAMKGVGTRHKAL
                                HQAMKGVGTRHKALI
                                HQAMKGVGTRHKALIRI
                                HQAMKGVGTRHKALIRIM
                                HQAMKGVGTRHKALIRIM
                                HQAMKGVGTRHKALIRIM
                                  AMKGVGTRHKALIRIM
                                   MKGVGTRHKALIRIM
                                   MKGVGTRHKALIRIM
                                   MKGVGTRHKALIRIM
                                   MKGVGTRHKALIRIM
                                     GVGTRHKALIRIM
                                       GTRHKALIRIM
                                              IRIM
                                               RIM
                                                 M
                                                 M
                                                 M
                                                 M
                                                 M

301      310       320       330       340   346
VSRSEIDMNDIKAFYQKMYGISLCQAILDETKGDYEKILVALCGGN
V
V
VS
V
VS
VSRSEIDMNDIKA
VSRSEIDMNDIKA
VSRSEIDMNDI
VSRSEIDMNDIKA
VSRSEIDMNDIKAFYQ
VSRSEIDMNDIKAFYQKM
VSRSEIDMNDIKAFYQKMYGISLC
VSRSEIDMNDI
VSRSEIDMNDIKA
VSRSEIDMNDIKAFY
VSRSEIDMNDIKAFYQ
VSRSEIDMNDIKAFYQKM
VSRSEIDMNDIKAFYQKMYGI
VSRSEIDMNDIKAFYQKMYGISLC
 SRSEIDMNDIKA
 SRSEIDMNDIKAFYQ
```

```
SRSEIDMNDIKAFYQKM
SRSEIDMNDIKAFYQKMYGISLC
 RSEIDMNDIKA
 RSEIDMNDIKAFYQ
 RSEIDMNDIKAFYQKM
   EIDMNDIKA
            FYQKMYGI
            FYQKMYGISLC
            FYQKMYGISLCQA
               KMYGISLC
               KMYGISLCQA
                       QAILDETKGDYEKI
                       QAILDETKGDYEKIL
                       QAILDETKGDYEKILV
                       QAILDETKGDYEKILVA
                       QAILDETKGDYEKILVALC
                       QAILDETKGDYEKILVALCGGN
                         ILDETKGDYEKIL
                         ILDETKGDYEKILV
                         ILDETKGDYEKILVA
                         ILDETKGDYEKILVAL
                         ILDETKGDYEKILVALC
                         ILDETKGDYEKILVALCGGN
                          LDETKGDYEKILV
                          LDETKGDYEKILVALC
                          LDETKGDYEKILVALCGGN
                           DETKGDYEKILV
                              KGDYEKILVALC
                              KGDYEKILVALCGGN
```

Supporting data 4

Folded ANXA1 15 sec 1 μM HTRA1

```
1        10        20        30        40        50
MAMVSEFLKQAWFIENEEQEYVQTVKSSKGGPGSAVSPYPTFNPSSDVAA
                         KSSKGGPGSAVSPYPTFNPSSDVA

51       60        70        80        90        100
LHKAIMVKGVDEATIIDILTKRNNAQRQQIKAAYLQETGKPLDETLKKAL

101      110       120       130       140       150
TGHLEEVVLALLKTPAQFDADELRAAMKGLGTDEDTLIEILASRTNKEIR

151      160       170       180       190       200
DINRVYREELKRDLAKDITSDTSGDFRNALLSLAKGDRSEDFGVNEDLAD

201      210       220       230       240       250
SDARALYEAGERRKGTDVNVFNTILTTRSYPQLRRVFQKYTKYSKHDMNK

251      260       270       280       290       300
VLDLELKGDIEKCLTAIVKCATSKPAFFAEKLHQAMKGVGTRHKALIRIM

301      310       320       330       340   346
VSRSEIDMNDIKAFYQKMYGISLCQAILDETKGDYEKILVALCGGN
```

Folded ANXA1 30 sec 1 μM HTRA1

```
1        10        20        30        40        50
MAMVSEFLKQAWFIENEEQEYVQTVKSSKGGPGSAVSPYPTFNPSSDVAA
           WFIENEEQEYVQTV
                         KSSKGGPGSAVSPYPT

51       60        70        80        90        100
LHKAIMVKGVDEATIIDILTKRNNAQRQQIKAAYLQETGKPLDETLKKAL
                                AYLQETGKPLDETL
                                AYLQETGKPLDETLKKAL

101      110       120       130       140       150
TGHLEEVVLALLKTPAQFDADELRAAMKGLGTDEDTLIEILASRTNKEIR
TGHLEEV
       VLALLKTPAQFDADEL
       VLALLKTPAQFDADELRA

151      160       170       180       190       200
DINRVYREELKRDLAKDITSDTSGDFRNALLSLAKGDRSEDFGVNEDLAD

201      210       220       230       240       250
SDARALYEAGERRKGTDVNVFNTILTTRSYPQLRRVFQKYTKYSKHDMNK

251      260       270       280       290       300
VLDLELKGDIEKCLTAIVKCATSKPAFFAEKLHQAMKGVGTRHKALIRIM

301      310       320       330       340   346
VSRSEIDMNDIKAFYQKMYGISLCQAILDETKGDYEKILVALCGGN
```

Folded ANXA1 15 sec 2 μM HTRA1

```
1        10        20        30        40        50
MAMVSEFLKQAWFIENEEQEYVQTVKSSKGGPGSAVSPYPTFNPSSDVAA

51       60        70        80        90        100
LHKAIMVKGVDEATIIDILTKRNNAQRQQIKAAYLQETGKPLDETLKKAL
                                AYLQETGKPLDETL

101      110       120       130       140       150
TGHLEEVVLALLKTPAQFDADELRAAMKGLGTDEDTLIEILASRTNKEIR

151      160       170       180       190       200
DINRVYREELKRDLAKDITSDTSGDFRNALLSLAKGDRSEDFGVNEDLAD

201      210       220       230       240       250
SDARALYEAGERRKGTDVNVFNTILTTRSYPQLRRVFQKYTKYSKHDMNK

251      260       270       280       290       300
VLDLELKGDIEKCLTAIVKCATSKPAFFAEKLHQAMKGVGTRHKALIRIM

301      310       320       330       340   346
VSRSEIDMNDIKAFYQKMYGISLCQAILDETKGDYEKILVALCGGN
```

Folded ANXA1 30 sec 2 μM HTRA1

```
1        10        20        30        40        50
MAMVSEFLKQAWFIENEEQEYVQTVKSSKGGPGSAVSPYPTFNPSSDVAA
           WFIENEEQEYVQTV
                                         FNPSSDVAA

51       60        70        80        90        100
LHKAIMVKGVDEATIIDILTKRNNAQRQQIKAAYLQETGKPLDETLKKAL
LHKAIMVKGVDEAT
    IMVKGVDEATIIDI
                                AYLQETGKPLDE
                                AYLQETGKPLDETL
                                AYLQETGKPLDETLK
                                AYLQETGKPLDETLKKA
                                AYLQETGKPLDETLKKAL
                                AYLQETGKPLDETLKKAL
                                   QETGKPLDETLKKAL

101      110       120       130       140       150
TGHLEEVVLALLKTPAQFDADELRAAMKGLGTDEDTLIEILASRTNKEIR
TG
TGHLEEV
TGHLEEV
       VLALLKTPAQFDADEL
       VLALLKTPAQFDADELRA
       VLALLKTPAQFDADELRAA

151      160       170       180       190       200
DINRVYREELKRDLAKDITSDTSGDFRNALLSLAKGDRSEDFGVNEDLAD

201      210       220       230       240       250
SDARALYEAGERRKGTDVNVFNTILTTRSYPQLRRVFQKYTKYSKHDMNK

251      260       270       280       290       300
VLDLELKGDIEKCLTAIVKCATSKPAFFAEKLHQAMKGVGTRHKALIRIM

301      310       320       330       340   346
VSRSEIDMNDIKAFYQKMYGISLCQAILDETKGDYEKILVALCGGN
```

Folded ANXA1 15 sec 4 μM HTRA1

```
1        10        20        30        40        50
MAMVSEFLKQAWFIENEEQEYVQTVKSSKGGPGSAVSPYPTFNPSSDVAA
           WFIENEEQEYVQTV
                         KSSKGGPGSAVSPYPTFNPSSDVA

51       60        70        80        90        100
LHKAIMVKGVDEATIIDILTKRNNAQRQQIKAAYLQETGKPLDETLKKAL
    IMVKGVDEATIIDI
                                AYLQETGKPLDETL
                                AYLQETGKPLDETLKKAL
                                AYLQETGKPLDETLKKAL

101      110       120       130       140       150
TGHLEEVVLALLKTPAQFDADELRAAMKGLGTDEDTLIEILASRTNKEIR
TG
TGHLEEV
       VLALLKTPAQFDADELRA

151      160       170       180       190       200
DINRVYREELKRDLAKDITSDTSGDFRNALLSLAKGDRSEDFGVNEDLAD

201      210       220       230       240       250
SDARALYEAGERRKGTDVNVFNTILTTRSYPQLRRVFQKYTKYSKHDMNK

251      260       270       280       290       300
VLDLELKGDIEKCLTAIVKCATSKPAFFAEKLHQAMKGVGTRHKALIRIM

301      310       320       330       340   346
VSRSEIDMNDIKAFYQKMYGISLCQAILDETKGDYEKILVALCGGN
                            DETKGDYEKILV
```

Folded ANXA1 30 sec 4 μM HTRA1

```
1        10        20        30        40        50
MAMVSEFLKQAWFIENEEQEYVQTVKSSKGGPGSAVSPYPTFNPSSDVAA
          AWFIENEEQEYVQTV
           WFIENEEQEYVQTV
                        VKSSKGGPGSAVSPYPT
                         KSSKGGPGSAVSPYPT
                         KSSKGGPGSAVSPYPTFNPSSDVA
                            KGGPGSAVSPYPT
                                         FNPSSDVAA
                                         FNPSSDVAA
                                         FNPSSDVAA

51       60        70        80        90        100
LHKAIMVKGVDEATIIDILTKRNNAQRQQIKAAYLQETGKPLDETLKKAL
LHKA
LHKAIMVK
LHKAIMVKGVDEAT
    IMVKGVDEATIIDI
                                AYLQETGKPLD
                                AYLQETGKPLDE
                                AYLQETGKPLDET
                                AYLQETGKPLDETL
                                AYLQETGKPLDETLK
                                AYLQETGKPLDETLKKA
                                AYLQETGKPLDETLKKAL
                                AYLQETGKPLDETLKKAL
                                AYLQETGKPLDETLKKAL
                                   QETGKPLDETLKKAL

101      110       120       130       140       150
TGHLEEVVLALLKTPAQFDADELRAAMKGLGTDEDTLIEILASRTNKEIR
TG
TGHLEEV
TGHLEEV
       VLALLKTPAQFDADEL
       VLALLKTPAQFDADELRA
       VLALLKTPAQFDADELRAA
                         AMKGLGTDEDTLIEIL
                         AMKGLGTDEDTLIEILA

151      160       170       180       190       200
DINRVYREELKRDLAKDITSDTSGDFRNALLSLAKGDRSEDFGVNEDLAD

201      210       220       230       240       250
SDARALYEAGERRKGTDVNVFNTILTTRSYPQLRRVFQKYTKYSKHDMNK

251      260       270       280       290       300
VLDLELKGDIEKCLTAIVKCATSKPAFFAEKLHQAMKGVGTRHKALIRIM

301      310       320       330       340   346
VSRSEIDMNDIKAFYQKMYGISLCQAILDETKGDYEKILVALCGGN
```

Supporting data 5

Example for how UMSAP calculates the relative frequency of cleavages

In a MS experiment the following peptides were identified. They all share the same P1 site at V231

A - RGHYV
B - GHYV
C - DFRTGH
D - DFRTGHKL
E - DFRTGHKLM
F - DFRTGHKLMT

Average intensities. 0 values mean that the peptide was not detected in the given time point or the intensity values are not significantly different to the control experiments.

Time Point	A	B	C	D	E	F
5 min	2	0	0	3	3	10
15 min	4	1	0	0	6	5
30 min	8	3	5	0	0	1

The first non-zero average intensity along the time points is taken as reference for the calculation of the relative intensity.

	Relative Intensities						Relative cleavage frequency
Time-point	A	B	C	D	E	F	P1 (V231)
5 min	2/2 = 1	0/1 = 0	0/5 = 0	3/3 = 1	3/3 = 1	10/10 = 1	1+0+0+1+1+1 = 4
15 min	4/2 = 2	1/1 = 1	0/5 = 0	0/3 = 0	6/3 = 2	5/10 = 0.5	2+1+0+0+2+0.5 = 5.5
30 min	8/2 = 4	3/1 = 1	5/5 = 1	0/3 = 0	0/3 = 0	1/10 = 0.1	4+1+1+0+0+0.1 = 6.1

Supporting data 6

Native SUS SCROFA MDH (P00346) sequence

```
MLSALARPAGAALRRSFSTSAQNNAKVAVLGASGGIGQPLSLLLKNSPLVSRLTLYDIAH
TPGVAADLSHIETRATVKGYLGPEQLPDCLKGCDVVVIPAGVPRKPGMTRDDLFNTNATI
VATLTAACAQHCPDAMICIISNPVNSTIPITAEVFKKHGVYNPNKIFGVTTLDIVRANAF
VAELKGLDPARVSVPVIGGHAGKTIIPLISQCTPKVDFPQDQLSTLTGRIQEAGTEVVKA
KAGAGSATLSMAYAGARFVFSLVDAMNGKEGVVECSFVKSQETDCPYFSTPLLLGKKGIE
KNLGIGKISPFEEKMIAEAIPELKASIKKGEEFVKNMK
```

Denatured MDH 0 sec

```
1        10        20        30        40        50
MLSALARPAGAALRRSFSTSAQNNAKVAVLGASGGIGQPLSLLLKNSPLV
                        AKVAVLGASGGIGQPLSL
                        AKVAVLGASGGIGQPLSLLLKN
                        AKVAVLGASGGIGQPLSLLLKNSPL
                        AKVAVLGASGGIGQPLSLLLKNSPLV
                                                LV

51       60        70        80        90        100
SRLTLYDIAHTPGVAADLSHIETRATVKGYLGPEQLPDCLKGCDVVVIPA
SR
SRLTLYD
         HTPGVAADLSHIETR

101      110       120       130       140       150
GVPRKPGMTRDDLFNTNATIVATLTAACAQHCPDAMICIISNPVNSTIPI

151      160       170       180       190       200
TAEVFKKHGVYNPNKIFGVTTLDIVRANAFVAELKGLDPARVSVPVIGGH
                                         VSVPVIGGH

201      210       220       230       240       250
AGKTIIPLISQCTPKVDFPQDQLSTLTGRIQEAGTEVVKAKAGAGSATLS
AGK

251      260       270       280       290       300
MAYAGARFVFSLVDAMNGKEGVVECSFVKSQETDCPYFSTPLLLGKKGIE

301      310       320       330     338
KNLGIGKISPFEEKMIAEAIPELKASIKKGEEFVKNMK
 NLGIGKISPFEEK
  LGIGKISPFEE
               IAEAIPELKASIKKGEEFVKNMK
```

Denatured MDH 15 sec

```
1        10        20        30        40        50
MLSALARPAGAALRRSFSTSAQNNAKVAVLGASGGIGQPLSLLLKNSPLV
                        AKVAVLGASGGIGQPLSLLLKN
                        AKVAVLGASGGIGQPLSLLLKNSPL
                        AKVAVLGASGGIGQPLSLLLKNSPLV
                         KVAVLGASGGIGQPLSLLLKN
                          VAVLGASGGIGQPLSLLLKNSPLV
                                 GGIGQPLSLLLK
                                    GQPLSLLLKNSPLV
                                      QPLSLLLK
                                       PLSLLLK
                                       PLSLLLKNSPLV
                                        LSLLLKNSPL
                                         SLLLKNSPLV
                                              NSPLV
                                              NSPLV
                                              NSPLV

51       60        70        80        90        100
SRLTLYDIAHTPGVAADLSHIETRATVKGYLGPEQLPDCLKGCDVVVIPA
SR
SR
SR
SR
SR
SR
SRLTLYDIAHTPGVAADLSHIETR
SRLTLYDIAHTPGVAADLSHIETRATVK
  LTLYDIAHTPGVA
  LTLYDIAHTPGVAADLSHIET
  LTLYDIAHTPGVAADLSHIETR
  LTLYDIAHTPGVAADLSHIETRATVK
   TLYDIAHTPGVAADLSHIETR
       IAHTPGVAADLSHIETR
         HTPGVAADLSHIETR
          TPGVAADLSHIETR
             VAADLSHIETR
               ADLSHIETR
                        ATVKGYLGPEQLPD
                        ATVKGYLGPEQLPDCLK

101      110       120       130       140       150
GVPRKPGMTRDDLFNTNATIVATLTAACAQHCPDAMICIISNPVNSTIPI
                                          PVNSTIPI

151      160       170       180       190       200
TAEVFKKHGVYNPNKIFGVTTLDIVRANAFVAELKGLDPARVSVPVIGGH
TAEVFKK
                          ANAFVAELK
                          ANAFVAELKGLDPAR
                                   GLDPARVSVPVIGGH
                                         VSVPVIGGH
                                          SVPVIGGH
                                           VPVIGGH
                                            PVIGGH

201      210       220       230       240       250
AGKTIIPLISQCTPKVDFPQDQLSTLTGRIQEAGTEVVKAKAGAGSATLS
AGK
AGK
AGK
AGK
```

```
201      210       220       230       240       250
AGKTIIPLISQCTPKVDFPQDQLSTLTGRIQEAGTEVVKAKAGAGSATLS
AGK
   TIIPLISQCTPKVDFPQDQLSTLTGR
               VDFPQDQLSTLTGR
               VDFPQDQLSTLTGRIQEAGTEVVK
               VDFPQDQLSTLTGRIQEAGTEVVKAK
                    DQLSTLTGR
                             IQEAGTEVVK
                             IQEAGTEVVKA
                             IQEAGTEVVKAK
                             IQEAGTEVVKAKAGAGSATLS
                              QEAGTEVVK
                              QEAGTEVVKAK
                               EAGTEVVKAK
                                AGTEVVKAK
                                       AKAGAGSATLS
                                       AKAGAGSATLS
                                        KAGAGSATLS
                                         AGAGSATLS
                                         AGAGSATLS
                                          GAGSATLS
                                           AGSATLS
                                            GSATLS
                                             SATLS
                                              ATLS
                                               TLS
                                                LS

251      260       270       280       290       300
MAYAGARFVFSLVDAMNGKEGVVECSFVKSQETDCPYFSTPLLLGKKGIE
MAYAGAR
MAYAGAR
MAYAGAR
MAY
MAYAGAR
MAYAGAR
MAYAGAR
MAYAGAR
MAYAGAR
MAYAGAR
MAYAGAR
MAYAGAR
MAYAGAR
                             SQETDCPYFSTPLLLGK
                                              KGIE
                                              KGIE
                                              KGIE
                                              KGIE
                                               GIE
                                               GIE
                                               GIE
                                               GIE
                                               GIE
                                               GIE
                                                IE
                                                 E

301      310       320       330     338
KNLGIGKISPFEEKMIAEAIPELKASIKKGEEFVKNMK
KNLGIGK
KNLGIGKISPFEEK
KNLGIGKISPFEEKMIAEAIPELK
KNLGIGKISPFEEKMIAEAIPELKASIK
```

```
301      310       320       330     338
KNLGIGKISPFEEKMIAEAIPELKASIKKGEEFVKNMK
KNLGIG
KNLGIGK
KNLGIGKISPFEE
KNLGIGKISPFEEK
KNLGIGKISPFEEKMIAEAIPELK
KNLGIGKISPFEEKMIAEAIPELKASIK
KNLGIGK
KNLGIGKISPFEEK
KNLGIGK
 NLGIGKISPFEE
 NLGIGKISPFEEK
 NLGIGKISPFEEKMIAEAIPELK
 NLGIGKISPFEEKMIAEAIPELKASIK
 NLGIGKISPFEEKMIAEAIPELKASIKKGEEFVK
 NLGIGKISPFEEKMIAEAIPELKASIKKGEEFVKNM
  LGIGKISPFEE
  LGIGKISPFEEK
   GIGKISPFEEK
       ISPFEEK
       ISPFEEKMIAEAIPELK
       ISPFEEKMIAEAIPELKASIK
       ISPFEEKMIAEAIPELKASIKKGEEFVK
       ISPFEEKMIAEAIPELKASIKKGEEFVKNM
       ISPFEEKMIAEAIPELKASIKKGEEFVKNMK
         PFEEKMIAEAIPELKASIKKGEEFVKNMK
              MIAEAIPELK
              MIAEAIPELKASIK
              MIAEAIPELKASIKKGE
              MIAEAIPELKASIKKGEEFVK
              MIAEAIPELKASIKKGEEFVKNM
              MIAEAIPELKASIKKGEEFVKNMK
                AEAIPELKASIKKGEEFVKNMK
                    PELKASIKKGEEFVKNMK
                        ASIKKGEEFVK
                        ASIKKGEEFVKNM
                        ASIKKGEEFVKNMK
                         SIKKGEEFVK
                         SIKKGEEFVKNMK
                          IKKGEEFVKNMK
                           KKGEEFVKNMK
                            KGEEFVK
                            KGEEFVKNM
                            KGEEFVKNMK
                             GEEFVKNM
                             GEEFVKNMK
                              EEFVKNMK
```

Denatured MDH 30 sec

```
1        10        20        30        40        50
MLSALARPAGAALRRSFSTSAQNNAKVAVLGASGGIGQPLSLLLKNSPLV
                        AKVAVLGASGGIGQP
                        AKVAVLGASGGIGQPLSL
                        AKVAVLGASGGIGQPLSLLLKN
                        AKVAVLGASGGIGQPLSLLLKNSPL
                        AKVAVLGASGGIGQPLSLLLKNSPLV
                          VAVLGASGGIGQPLSLLLK
                          VAVLGASGGIGQPLSLLLKNSPLV
                                SGGIGQPLSLLLK
                                 GGIGQPLSLLLK
                                    GQPLSLLLKNSPLV
                                     QPLSLLL
                                     QPLSLLLKNSPLV
                                      PLSLLLK
                                      PLSLLLKNSPLV
                                       LSLLLKNSPL
                                       LSLLLKNSPLV
                                        SLLLKNSPLV
                                         LLLKNSPLV
                                             NSPLV
                                             NSPLV
                                             NSPLV
                                             NSPLV
                                             NSPLV
                                             NSPLV
                                               PLV
                                                LV

51       60        70        80        90        100
SRLTLYDIAHTPGVAADLSHIETRATVKGYLGPEQLPDCLKGCDVVVIPA
SR
SR
SR
SR
SR
SR
SR
SR
SR
SRLT
SRLTLYD
SRLTLYDIAHTPGVAADLSHIET
SRLTLYDIAHTPGVAADLSHIETR
SRLTLYDIAHTPGVAADLSHIETRATVK
SRLTLYDIAHTPGVAADLSHIETR
SRLTLYD
  LTLYDIAHTPG
  LTLYDIAHTPGVA
  LTLYDIAHTPGVAAD
  LTLYDIAHTPGVAADLSHIET
  LTLYDIAHTPGVAADLSHIETR
  LTLYDIAHTPGVAADLSHIETRATVK
   TLYDIAHTPGVAADLSHIETR
    LYDIAHTPGVA
    LYDIAHTPGVAADLSHIETR
    LYDIAHTPGVAADLSHIETRATVK
     YDIAHTPGVAADLSHIETR
       IAHTPGVAADLSHIETR
         HTPGVAADLSHIETR
            GVAADLSHIETR
             VAADLSHIETR
```

```
51       60        70        80        90        100
SRLTLYDIAHTPGVAADLSHIETRATVKGYLGPEQLPDCLKGCDVVVIPA
               ADLSHIETR
                        ATVKGYLGPEQLPD
                        ATVKGYLGPEQLPDCLK
                        ATVKGYLGPEQLPDCLKGCDVVVIPA

101      110       120       130       140       150
GVPRKPGMTRDDLFNTNATIVATLTAACAQHCPDAMICIISNPVNSTIPI
GVPR
                                          PVNSTIPI

151      160       170       180       190       200
TAEVFKKHGVYNPNKIFGVTTLDIVRANAFVAELKGLDPARVSVPVIGGH
TAEVFKK
      KHGVYNPNKIFGVTTLDIVR
       HGVYNPNKIFGVTTLDIVR
       HGVYNPNKIFGVTTLDIVRANAFVAELK
        GVYNPNKIFGVTTLDIVR
                          ANAFVAELK
                          ANAFVAELKGLD
                          ANAFVAELKGLDPAR
                            AFVAELK
                                   GLDPARVSVPVIGGH
                                         VSVPVIGGH
                                         VSVPVIGGH
                                          SVPVIGGH
                                           VPVIGGH
                                            PVIGGH

201      210       220       230       240       250
AGKTIIPLISQCTPKVDFPQDQLSTLTGRIQEAGTEVVKAKAGAGSATLS
AGK
AGK
AGKTIIPLISQCTPK
AGK
AGK
AGK
   TIIPLISQCTPKVDFPQDQLSTLTGR
   TIIPLISQCTPKVDFPQDQLSTLTGRIQEAGTEVVK
               VDFPQDQLSTLTGR
               VDFPQDQLSTLTGRIQEAGTEVVK
               VDFPQDQLSTLTGRIQEAGTEVVKAK
                 FPQDQLSTLTGR
                  PQDQLSTLTGR
                             IQEAGTEVVK
                             IQEAGTEVVKA
                             IQEAGTEVVKAK
                             IQEAGTEVVKAKAGAGSATLS
                              QEAGTEVVK
                              QEAGTEVVKAK
                               EAGTEVVKAK
                               EAGTEVVKAKAGAGSATLS
                                AGTEVVKAK
                                       AKAGAGSATLS
                                       AKAGAGSATLS
                                        KAGAGSATLS
                                         AGAGSATLS
                                         AGAGSATLS
                                         AGAGSATLS
                                          GAGSATLS
                                           AGSATLS
                                            GSATLS
```

```
201      210       220       230       240       250
AGKTIIPLISQCTPKVDFPQDQLSTLTGRIQEAGTEVVKAKAGAGSATLS
                                             SATLS
                                              ATLS
                                               TLS
                                                LS
                                                 S

251      260       270       280       290       300
MAYAGARFVFSLVDAMNGKEGVVECSFVKSQETDCPYFSTPLLLGKKGIE
MAYAGAR
MAYAGAR
MAYAGAR
MAYAGAR
MAY
MAYAGA
MAYAGAR
MAYAGAR
MAYAGAR
MAYAGAR
MAYAGAR
MAYAGAR
MAYAGAR
MAYAGAR
MAYAGAR
MAYAGAR
       FVFSLVDAMNGK
       FVFSLVDAMNGKEGVVECSFVK
                   EGVVECSFVK
                             SQETDCPYFSTPLLLGK
                             SQETDCPYFSTPLLLGKK
                             SQETDCPYFSTPLLLGKKGIE
                                     FSTPLLLGKK
                                              KGIE
                                              KGIE
                                              KGIE
                                              KGIE
                                              KGIE
                                               GIE
                                               GIE
                                               GIE
                                               GIE
                                               GIE
                                               GIE
                                               GIE
                                               GIE
                                                IE
                                                 E
                                                 E

301      310       320       330     338
KNLGIGKISPFEEKMIAEAIPELKASIKKGEEFVKNMK
K
KNLGIGK
KNLGIGKISPFEEK
KNLGIGKISPFEEKMIA
KNLGIGKISPFEEKMIAEAIPELK
KNLGIGKISPFEEKMIAEAIPELKASIK
KNLGIG
KNLGIGK
KNLGIGKISPF
KNLGIGKISPFEE
KNLGIGKISPFEEK
KNLGIGKISPFEEKM
```

```
301      310       320       330     338
KNLGIGKISPFEEKMIAEAIPELKASIKKGEEFVKNMK
KNLGIGKISPFEEKMIAEAIPELK
KNLGIGKISPFEEKMIAEAIPELKASIK
KNLGIGK
KNLGIGK
KNLGIGKISPFEEK
KNLGIGK
 NLGIGKISPF
 NLGIGKISPFEE
 NLGIGKISPFEEK
 NLGIGKISPFEEKM
 NLGIGKISPFEEKMIA
 NLGIGKISPFEEKMIAEAIPELK
 NLGIGKISPFEEKMIAEAIPELKASIK
 NLGIGKISPFEEKMIAEAIPELKASIKKGEEFVK
 NLGIGKISPFEEKMIAEAIPELKASIKKGEEFVKNM
  LGIGKISPFE
  LGIGKISPFEE
  LGIGKISPFEEK
   GIGKISPFEEK
    IGKISPFEEK
     GKISPFEEK
      KISPFEEK
      KISPFEEKM
       ISPFEEK
       ISPFEEKM
       ISPFEEKMIA
       ISPFEEKMIAE
       ISPFEEKMIAEAIPELK
       ISPFEEKMIAEAIPELKASIK
       ISPFEEKMIAEAIPELKASIKKGEEFVK
       ISPFEEKMIAEAIPELKASIKKGEEFVKNM
       ISPFEEKMIAEAIPELKASIKKGEEFVKNMK
         PFEEKMIAEAIPELKASIK
         PFEEKMIAEAIPELKASIKKGEEFVK
              MIAEAIPELK
              MIAEAIPELKASIK
              MIAEAIPELKASIKKG
              MIAEAIPELKASIKKGE
              MIAEAIPELKASIKKGEEFVK
              MIAEAIPELKASIKKGEEFVKNM
              MIAEAIPELKASIKKGEEFVKNMK
               IAEAIPELKASIKKGEEFVKNMK
                AEAIPELK
                AEAIPELKASIK
                AEAIPELKASIKKGEEFVKNMK
                 EAIPELKASIK
                  AIPELKASIK
                  AIPELKASIKKGEEFVKNMK
                    PELKASIKKGEEFVKNMK
                        ASIKKGEEFV
                        ASIKKGEEFVK
                        ASIKKGEEFVKNMK
                         SIKKGEEFVK
                         SIKKGEEFVKNM
                         SIKKGEEFVKNMK
                          IKKGEEFVKNMK
                           KKGEEFVKNMK
                            KGEEFVK
                            KGEEFVKN
                            KGEEFVKNM
                            KGEEFVKNMK
                             GEEFVKNM
```

```
301      310       320       330     338
KNLGIGKISPFEEKMIAEAIPELKASIKKGEEFVKNMK
                             GEEFVKNMK
                              EEFVKNMK
```

Denatured MDH 45 sec

```
1        10        20        30        40        50
MLSALARPAGAALRRSFSTSAQNNAKVAVLGASGGIGQPLSLLLKNSPLV
                        AKVAVLGASGGIG
                        AKVAVLGASGGIGQP
                        AKVAVLGASGGIGQPLSL
                        AKVAVLGASGGIGQPLSLLLKN
                        AKVAVLGASGGIGQPLSLLLKNSPLV
                          VAVLGASGGIGQPLSLLLK
                          VAVLGASGGIGQPLSLLLKNSPLV
                              GASGGIGQPLSLLLK
                              GASGGIGQPLSLLLKNSPLV
                                SGGIGQPLSLLLK
                                 GGIGQPLSLLLK
                                    GQPLSLLLKNSPLV
                                     QPLSLLL
                                      PLSLLLK
                                      PLSLLLKNSPLV
                                       LSLLLKNSPL
                                       LSLLLKNSPLV
                                        SLLLKNSPLV
                                         LLLKNSPLV
                                             NSPLV
                                             NSPLV
                                             NSPLV
                                             NSPLV
                                             NSPLV
                                             NSPLV
                                             NSPLV
                                             NSPLV
                                             NSPLV
                                               PLV
                                                LV
                                                 V

51       60        70        80        90        100
SRLTLYDIAHTPGVAADLSHIETRATVKGYLGPEQLPDCLKGCDVVVIPA
SR
SR
SR
SR
SR
SR
SR
SR
SR
SRLT
SRLTLYD
SRLTLYDIA
SRLTLYDIAHTPG
SRLTLYDIAHTPGVA
SRLTLYDIAHTPGVAADLSHIET
SRLTLYDIAHTPGVAADLSHIETR
SRLTLYDIAHTPGVAADLSHIETRATVK
SRLTLYDIAHTPGVAADLSHIETR
SRLTLYDIAHTPGVAADLSHIETR
SRLTLYD
SRLTLYDIAHTPGVAADLSHIETR
  LTLYDIAHTP
  LTLYDIAHTPG
  LTLYDIAHTPGVA
  LTLYDIAHTPGVAA
  LTLYDIAHTPGVAAD
```

```
51       60        70        80        90        100
SRLTLYDIAHTPGVAADLSHIETRATVKGYLGPEQLPDCLKGCDVVVIPA
  LTLYDIAHTPGVAADLSHIET
  LTLYDIAHTPGVAADLSHIETR
  LTLYDIAHTPGVAADLSHIETRATVK
   TLYDIAHTPGVAADLSHIETR
   TLYDIAHTPGVAADLSHIETRATVK
    LYDIAHTPGVA
    LYDIAHTPGVAADLSHIETR
    LYDIAHTPGVAADLSHIETRATVK
     YDIAHTPGVAADLSHIETR
     YDIAHTPGVAADLSHIETRATVK
      DIAHTPGVAADLSHIETR
       IAHTPGVAADLSHIETR
         HTPGVAADLSHIETR
           PGVAADLSHIETR
            GVAADLSHIETR
             VAADLSHIETR
              AADLSHIETR
               ADLSHIETR
                        ATVKGYLGPEQLPD
                        ATVKGYLGPEQLPDCLK
                        ATVKGYLGPEQLPDCLKGCDVVVIPA
                        ATVKGYLGPEQLPDCLKGCDVVVIPA

101      110       120       130       140       150
GVPRKPGMTRDDLFNTNATIVATLTAACAQHCPDAMICIISNPVNSTIPI
GVPR
GVPRKPGMTR
                                          PVNSTIPI

151      160       170       180       190       200
TAEVFKKHGVYNPNKIFGVTTLDIVRANAFVAELKGLDPARVSVPVIGGH
TAEVFKK
      KHGVYNPNKIFGVTTLDIVR
       HGVYNPNKIFGVTTLDIVR
       HGVYNPNKIFGVTTLDIVRANAFVAELK
        GVYNPNKIFGVTTLDIVR
         VYNPNKIFGVTTLDIVR
           NPNKIFGVTTLDIVR
              KIFGVTTLDIVR
                          ANAFVAELK
                          ANAFVAELKGLD
                          ANAFVAELKGLDPAR
                          ANAFVAELKGLDPARVSVPVIGGH
                            AFVAELKGLDPAR
                              VAELKGLDPAR
                                   GLDPARVSVPVIGGH
                                   GLDPARVSVPVIGGH
                                      PARVSVPVIGGH
                                         VSVPVIGGH
                                         VSVPVIGGH
                                          SVPVIGGH
                                           VPVIGGH
                                            PVIGGH

201      210       220       230       240       250
AGKTIIPLISQCTPKVDFPQDQLSTLTGRIQEAGTEVVKAKAGAGSATLS
AGK
AGK
AGKTIIPLISQCTPK
AGK
AGK
AGKTIIPLISQCTPK
```

```
201      210       220       230       240       250
AGKTIIPLISQCTPKVDFPQDQLSTLTGRIQEAGTEVVKAKAGAGSATLS
AGK
AGK
AGK
   TIIPLISQCTPK
   TIIPLISQCTPKVDFPQDQLSTLTGR
   TIIPLISQCTPKVDFPQDQLSTLTGRIQEAGTEVVK
      PLISQCTPK
               VDFPQDQLSTLTGR
               VDFPQDQLSTLTGRIQEAGTEVVK
               VDFPQDQLSTLTGRIQEAGTEVVKAK
                 FPQDQLSTLTGR
                 FPQDQLSTLTGRIQEAGTEVVKAK
                    DQLSTLTGR
                     QLSTLTGR
                          TGRIQEAGTEVVK
                          TGRIQEAGTEVVKAK
                          TGRIQEAGTEVVKAKAGAGSATLS
                             IQEAGTEVVK
                             IQEAGTEVVKA
                             IQEAGTEVVKAK
                             IQEAGTEVVKAKAGAGSATLS
                              QEAGTEVVK
                              QEAGTEVVKAK
                               EAGTEVVKAK
                               EAGTEVVKAKAGAGSATLS
                                AGTEVVKAK
                                AGTEVVKAKAGAGSATLS
                                 GTEVVKAKAGAGSATLS
                                    VVKAKAGAGSATLS
                                       AKAGAGSATLS
                                       AKAGAGSATLS
                                        KAGAGSATLS
                                         AGAGSATLS
                                         AGAGSATLS
                                         AGAGSATLS
                                         AGAGSATLS
                                          GAGSATLS
                                           AGSATLS
                                            GSATLS
                                             SATLS
                                              ATLS
                                               TLS
                                                LS
                                                 S

251      260       270       280       290       300
MAYAGARFVFSLVDAMNGKEGVVECSFVKSQETDCPYFSTPLLLGKKGIE
MAYAGAR
MAYAGAR
MAYAGAR
MAYAGAR
MAYAGAR
MAYAGAR
MAYAGAR
MAYAGAR
MAY
MAYA
MAYAGA
MAYAGAR
MAYAGAR
MAYAGAR
MAYAGAR
```

```
251      260       270       280       290       300
MAYAGARFVFSLVDAMNGKEGVVECSFVKSQETDCPYFSTPLLLGKKGIE
MAYAGAR
MAYAGAR
MAYAGAR
MAYAGAR
MAYAGAR
MAYAGAR
       FVFSLVDAMNGK
       FVFSLVDAMNGKE
       FVFSLVDAMNGKEGVVECSFVK
       FVFSLVDAMNGKEGVVECSFVKSQETD
                   EGVVECSFVK
                             SQETDCPYFSTPLLLGK
                             SQETDCPYFSTPLLLGKK
                             SQETDCPYFSTPLLLGKKGIE
                             SQETDCPYFSTPLLLGKKGIE
                             SQETDCPYFSTPLLLGKKGIE
                               ETDCPYFSTPLLLGK
                                TDCPYFSTPLLLGK
                                   PYFSTPLLLGK
                                   PYFSTPLLLGKK
                                    YFSTPLLLGK
                                     FSTPLLLGK
                                     FSTPLLLGKK
                                            GKKGIE
                                              KGIE
                                              KGIE
                                              KGIE
                                              KGIE
                                              KGIE
                                              KGIE
                                              KGIE
                                              KGIE
                                               GIE
                                               GIE
                                               GIE
                                               GIE
                                               GIE
                                               GIE
                                               GIE
                                               GIE
                                               GIE
                                                IE
                                                IE
                                                 E
                                                 E
                                                 E

301      310       320       330     338
KNLGIGKISPFEEKMIAEAIPELKASIKKGEEFVKNMK
K
KNLGIGK
KNLGIGKISPFEEK
KNLGIGKISPFEEK
KNLGIGK
KNLGIGKISPF
KNLGIGKISPFEEK
KNLGIGKISPFEEKM
KNLGIGKISPFEEKMIA
KNLGIGKISPFEEKMIAE
KNLGIGKISPFEEKMIAEAIPELK
KNLGIGKISPFEEKMIAEAIPELKASIK
KNLGIG
```

```
301      310       320       330     338
KNLGIGKISPFEEKMIAEAIPELKASIKKGEEFVKNMK
KNLGIGK
KNLGIGKISPF
KNLGIGKISPFEE
KNLGIGKISPFEEK
KNLGIGKISPFEEKM
KNLGIGKISPFEEKMIAE
KNLGIGKISPFEEKMIAEAIPELK
KNLGIGKISPFEEKMIAEAIPELKASIK
KNLGIGK
KNLGIGKISPFEEK
KNLGIGK
KNLGIGKISPFEEK
KNLGIGKISPFEEKMIAEAIPELKASIK
KNLGIGK
 NLGIGKISPF
 NLGIGKISPFEE
 NLGIGKISPFEEK
 NLGIGKISPFEEKM
 NLGIGKISPFEEKMIA
 NLGIGKISPFEEKMIAEAIPELK
 NLGIGKISPFEEKMIAEAIPELKASIK
 NLGIGKISPFEEKMIAEAIPELKASIKK
 NLGIGKISPFEEKMIAEAIPELKASIKKGEEFVK
 NLGIGKISPFEEKMIAEAIPELKASIKKGEEFVKNM
  LGIGKISPFE
  LGIGKISPFEE
  LGIGKISPFEEK
   GIGKISPFEEK
   GIGKISPFEEKMIAEAIPELK
   GIGKISPFEEKMIAEAIPELKASIK
    IGKISPFEEK
     GKISPFEEK
      KISPFEEK
      KISPFEEKM
      KISPFEEKMIA
      KISPFEEKMIAE
       ISPFEEK
       ISPFEEKM
       ISPFEEKMIA
       ISPFEEKMIAE
       ISPFEEKMIAEAIPELK
       ISPFEEKMIAEAIPELKASIK
       ISPFEEKMIAEAIPELKASIKKGEEFVK
       ISPFEEKMIAEAIPELKASIKKGEEFVKNM
       ISPFEEKMIAEAIPELKASIKKGEEFVKNMK
        SPFEEKMIAEAIPELKASIK
         PFEEKMIAEAIPELK
         PFEEKMIAEAIPELKASIK
         PFEEKMIAEAIPELKASIKKGEEFVK
           EEKMIAEAIPELKASIKKGEEFVKNMK
              MIAEAIPELK
              MIAEAIPELKA
              MIAEAIPELKASIK
              MIAEAIPELKASIKK
              MIAEAIPELKASIKKG
              MIAEAIPELKASIKKGE
              MIAEAIPELKASIKKGEEFVK
              MIAEAIPELKASIKKGEEFVKNM
              MIAEAIPELKASIKKGEEFVKNMK
               IAEAIPELKASIK
               IAEAIPELKASIKKGEEFVKNMK
                AEAIPELK
```

```
301      310       320       330     338
KNLGIGKISPFEEKMIAEAIPELKASIKKGEEFVKNMK
                AEAIPELKASIK
                AEAIPELKASIKKGEEFVK
                AEAIPELKASIKKGEEFVKNMK
                 EAIPELK
                 EAIPELKASIK
                 EAIPELKASIKKGEEFVKNMK
                  AIPELKASIK
                  AIPELKASIKKGEEFVK
                  AIPELKASIKKGEEFVKNMK
                    PELKASIK
                    PELKASIKKGEEFVK
                    PELKASIKKGEEFVKNMK
                     ELKASIKKGEEFVKNMK
                        ASIKKGEEFV
                        ASIKKGEEFVK
                        ASIKKGEEFVKNMK
                         SIKKGEEFVK
                         SIKKGEEFVKNM
                         SIKKGEEFVKNMK
                          IKKGEEFVKNM
                          IKKGEEFVKNMK
                           KKGEEFVKNMK
                            KGEEFVK
                            KGEEFVKN
                            KGEEFVKNM
                            KGEEFVKNMK
                             GEEFVKNM
                             GEEFVKNMK
                              EEFVKNMK
```

Denatured MDH 60 sec

```
1        10        20        30        40        50
MLSALARPAGAALRRSFSTSAQNNAKVAVLGASGGIGQPLSLLLKNSPLV
                        AKVAVLGASGGIGQPLSL
                        AKVAVLGASGGIGQPLSLLLKN
                        AKVAVLGASGGIGQPLSLLLKNSPLV
                         KVAVLGASGGIGQPLSLLLKN
                          VAVLGASGGIGQPLSLLLK
                          VAVLGASGGIGQPLSLLLKNSPLV
                            VLGASGGIGQPLSLLLK
                              GASGGIGQPLSLLLK
                              GASGGIGQPLSLLLKNSPLV
                                SGGIGQPLSLLLK
                                 GGIGQPLSLLLK
                                 GGIGQPLSLLLKNSPLV
                                    GQPLSLLLKNSPLV
                                     QPLSLLL
                                      PLSLLLK
                                      PLSLLLKNSPLV
                                       LSLLLKNSPL
                                       LSLLLKNSPLV
                                        SLLLKNSPLV
                                         LLLKNSPLV
                                             NSPLV
                                             NSPLV
                                             NSPLV
                                             NSPLV
                                             NSPLV
                                             NSPLV
                                             NSPLV
                                             NSPLV
                                              SPLV
                                               PLV
                                                 V

51       60        70        80        90        100
SRLTLYDIAHTPGVAADLSHIETRATVKGYLGPEQLPDCLKGCDVVVIPA
SR
SR
SR
SR
SR
SR
SR
SR
SR
SR
SRLTLYD
SRLTLYDIA
SRLTLYDIAHTPG
SRLTLYDIAHTPGVA
SRLTLYDIAHTPGVAADLSHIET
SRLTLYDIAHTPGVAADLSHIETR
SRLTLYDIAHTPGVAADLSHIETRATVK
SRLTLYDIAHTPGVAADLSHIETR
SRLTLYDIAHTPGVAADLSHIETR
SRLTLYDIAHTPGVAADLSHIETR
  LTLYDIAH
  LTLYDIAHTP
  LTLYDIAHTPG
  LTLYDIAHTPGVA
  LTLYDIAHTPGVAA
```

```
51       60        70        80        90        100
SRLTLYDIAHTPGVAADLSHIETRATVKGYLGPEQLPDCLKGCDVVVIPA
  LTLYDIAHTPGVAAD
  LTLYDIAHTPGVAADLSHIET
  LTLYDIAHTPGVAADLSHIETR
  LTLYDIAHTPGVAADLSHIETRATVK
   TLYDIAHTPGVAADLSHIETR
   TLYDIAHTPGVAADLSHIETRATVK
    LYDIAHTPGVA
    LYDIAHTPGVAADLSHIETR
    LYDIAHTPGVAADLSHIETRATVK
     YDIAHTPGVAADLSHIETR
      DIAHTPGVAADLS
      DIAHTPGVAADLSHIETR
       IAHTPGVAADLSHIETR
         HTPGVAADLSHIETR
          TPGVAADLSHIETR
           PGVAADLSHIETR
            GVAADLSHIETR
             VAADLSHIETR
               ADLSHIETR
                        ATVKGYLGPEQLPD
                        ATVKGYLGPEQLPDCLK
                        ATVKGYLGPEQLPDCLKGCDVVVIPA

101      110       120       130       140       150
GVPRKPGMTRDDLFNTNATIVATLTAACAQHCPDAMICIISNPVNSTIPI
GVPR
                                          PVNSTIPI

151      160       170       180       190       200
TAEVFKKHGVYNPNKIFGVTTLDIVRANAFVAELKGLDPARVSVPVIGGH
TAEVFKK
      KHGVYNPNKIFGVTTLDIVR
       HGVYNPNKIFGVTTLDIVR
       HGVYNPNKIFGVTTLDIVRANAFVAELK
        GVYNPNKIFGVTTLDIVR
         VYNPNKIFGVTTLDIVR
           NPNKIFGVTTLDIVR
              KIFGVTTLDIVR
                          ANAFVAELK
                          ANAFVAELKGLD
                          ANAFVAELKGLDPAR
                          ANAFVAELKGLDPARVSVPVIGGH
                          ANAFVAELKGLDPARVSVPVIGGH
                            AFVAELKGLDPAR
                              VAELKGLDPAR
                               AELKGLDPAR
                                  KGLDPARVSVPVIGGH
                                   GLDPARVSVPVIGGH
                                   GLDPARVSVPVIGGH
                                     DPARVSVPVIGGH
                                         VSVPVIGGH
                                         VSVPVIGGH
                                          SVPVIGGH
                                           VPVIGGH
                                            PVIGGH

201      210       220       230       240       250
AGKTIIPLISQCTPKVDFPQDQLSTLTGRIQEAGTEVVKAKAGAGSATLS
AGK
AGK
AG
AGK
```

```
201      210       220       230       240       250
AGKTIIPLISQCTPKVDFPQDQLSTLTGRIQEAGTEVVKAKAGAGSATLS
AGKTIIPLISQCTPK
AGK
AGK
AGKTIIPLISQCTPK
AGK
AGK
AGK
   TIIPLISQCTPK
   TIIPLISQCTPKVD
   TIIPLISQCTPKVDFPQDQLSTLTGR
   TIIPLISQCTPKVDFPQDQLSTLTGRIQEAGTEVVK
      PLISQCTPK
               VDFPQDQLSTLTGR
               VDFPQDQLSTLTGRIQEAGTEVVK
               VDFPQDQLSTLTGRIQEAGTEVVKAK
                 FPQDQLSTLTGR
                 FPQDQLSTLTGRIQEAGTEVVKAK
                  PDQLSTLTGR
                     QLSTLTGR
                          TGRIQEAGTEVVK
                          TGRIQEAGTEVVKAK
                             IQEAGTEVVK
                             IQEAGTEVVKA
                             IQEAGTEVVKAK
                             IQEAGTEVVKAKAGAGSATLS
                              QEAGTEVVK
                              QEAGTEVVKAK
                               EAGTEVVKAK
                               EAGTEVVKAKAGAGSATLS
                                AGTEVVKAK
                                AGTEVVKAKAGAGSATLS
                                       AKAGAGSATLS
                                       AKAGAGSATLS
                                       AKAGAGSATLS
                                        KAGAGSATLS
                                         AGAGSATLS
                                         AGAGSATLS
                                         AGAGSATLS
                                         AGAGSATLS
                                          GAGSATLS
                                           AGSATLS
                                            GSATLS
                                             SATLS
                                              ATLS
                                               TLS
                                                LS
                                                 S
```

```
251      260       270       280       290       300
MAYAGARFVFSLVDAMNGKEGVVECSFVKSQETDCPYFSTPLLLGKKGIE
MAYAGAR
MAYAGAR
MAY
MAYAGAR
MAYAGAR
MAY
MAYA
MAYAGAR
MAY
MAYA
MAYAGA
MAYAGAR
```

```
251      260       270       280       290       300
MAYAGARFVFSLVDAMNGKEGVVECSFVKSQETDCPYFSTPLLLGKKGIE
MAYAGAR
MAYAGAR
MAYAGAR
MAYAGAR
MAYAGAR
MAYAGAR
MAYAGAR
MAYAGAR
MAYAGAR
MAYAGAR
MAYAGAR
       FVFSLVDAMNGK
       FVFSLVDAMNGKEGVVECSFVK
       FVFSLVDAMNGKEGVVECSFVKSQETD
                   EGVVECSFVK
                             SQETDCPYFSTPLLLGK
                             SQETDCPYFSTPLLLGKK
                             SQETDCPYFSTPLLLGKKGIE
                             SQETDCPYFSTPLLLGKKGIE
                               ETDCPYFSTPLLLGK
                               ETDCPYFSTPLLLGKK
                                TDCPYFSTPLLLGK
                                TDCPYFSTPLLLGKK
                                  CPYFSTPLLLGK
                                   PYFSTPLLLGK
                                   PYFSTPLLLGKK
                                    YFSTPLLLGK
                                     FSTPLLLGK
                                     FSTPLLLGKK
                                      STPLLLGKKGIE
                                         LLGKKGIE
                                            GKKGIE
                                              KGIE
                                              KGIE
                                              KGIE
                                              KGIE
                                              KGIE
                                              KGIE
                                              KGIE
                                               GIE
                                               GIE
                                               GIE
                                               GIE
                                               GIE
                                               GIE
                                               GIE
                                               GIE
                                                IE
                                                IE
                                                 E
                                                 E

301      310       320       330     338
KNLGIGKISPFEEKMIAEAIPELKASIKKGEEFVKNMK
K
KNLGIGK
K
KNLGIGK
KNLGIGKISPFEEK
KNLGIGKISPFEEKMIAEAIPELKASIKKGEEFVKNMK
KNLGIGK
KNLGIGKISPF
```

```
301      310       320       330     338
KNLGIGKISPFEEKMIAEAIPELKASIKKGEEFVKNMK
KNLGIGKISPFEEK
KNLGIGKISPFEEKMIA
KNLGIGKISPFEEKMIAE
KNLGIGKISPFEEKMIAEAIPELK
KNLGIGKISPFEEKMIAEAIPELKASIK
KNLGIG
KNLGIGK
KNLGIGKISPF
KNLGIGKISPFEE
KNLGIGKISPFEEK
KNLGIGKISPFEEKM
KNLGIGKISPFEEKMIAE
KNLGIGKISPFEEKMIAEAIPELK
KNLGIGKISPFEEKMIAEAIPELKASIK
KNLGIGK
KNLGIGKISPFEEK
KNLGIGK
KNLGIGKISPFEEK
KNLGIGK
KNLGIGKISPFEEK
 NLGIGKISPF
 NLGIGKISPFEE
 NLGIGKISPFEEK
 NLGIGKISPFEEKM
 NLGIGKISPFEEKMIA
 NLGIGKISPFEEKMIAEAIPELK
 NLGIGKISPFEEKMIAEAIPELKASIK
 NLGIGKISPFEEKMIAEAIPELKASIKK
 NLGIGKISPFEEKMIAEAIPELKASIKKGEEFVK
 NLGIGKISPFEEKMIAEAIPELKASIKKGEEFVKNM
  LGIGKISPFE
  LGIGKISPFEE
  LGIGKISPFEEK
   GIGKISPFEEK
   GIGKISPFEEKMIAEAIPELK
   GIGKISPFEEKMIAEAIPELKASIK
    IGKISPFEEK
     GKISPFEEK
      KISPFEEK
       ISPFEEK
       ISPFEEKM
       ISPFEEKMIA
       ISPFEEKMIAE
       ISPFEEKMIAEAIPELK
       ISPFEEKMIAEAIPELKASIK
       ISPFEEKMIAEAIPELKASIKKGEEFVK
       ISPFEEKMIAEAIPELKASIKKGEEFVKNM
       ISPFEEKMIAEAIPELKASIKKGEEFVKNMK
        SPFEEKMIAEAIPELKASIK
         PFEEKMIAEAIPELK
         PFEEKMIAEAIPELKASIK
         PFEEKMIAEAIPELKASIKKGEEFVK
              MIAEAIPELK
              MIAEAIPELKASIK
              MIAEAIPELKASIKK
              MIAEAIPELKASIKKG
              MIAEAIPELKASIKKGE
              MIAEAIPELKASIKKGEEFVK
              MIAEAIPELKASIKKGEEFVKNM
              MIAEAIPELKASIKKGEEFVKNMK
               IAEAIPELKASIK
               IAEAIPELKASIKKGEEFVKNMK
```

```
301      310       320       330     338
KNLGIGKISPFEEKMIAEAIPELKASIKKGEEFVKNMK
                AEAIPELK
                AEAIPELKASIK
                AEAIPELKASIKKGEEFVK
                AEAIPELKASIKKGEEFVKNMK
                 EAIPELK
                 EAIPELKASIK
                 EAIPELKASIKKGEEFVK
                 EAIPELKASIKKGEEFVKNMK
                  AIPELKASIK
                  AIPELKASIKKGEEFVK
                  AIPELKASIKKGEEFVKNMK
                   IPELKASIK
                    PELKASIK
                    PELKASIKKGEEFVK
                    PELKASIKKGEEFVKNMK
                        ASIKKGEEFV
                        ASIKKGEEFVK
                        ASIKKGEEFVKNMK
                         SIKKGEEFVK
                         SIKKGEEFVKNM
                         SIKKGEEFVKNMK
                          IKKGEEFVKNM
                          IKKGEEFVKNMK
                           KKGEEFVKNM
                           KKGEEFVKNMK
                            KGEEFVK
                            KGEEFVKN
                            KGEEFVKNM
                            KGEEFVKNMK
                             GEEFVKNM
                             GEEFVKNMK
                              EEFVKNMK
```

Denatured MDH 120 sec

```
1        10        20        30        40        50
MLSALARPAGAALRRSFSTSAQNNAKVAVLGASGGIGQPLSLLLKNSPLV
                        AKVAVLGASGGIG
                        AKVAVLGASGGIGQP
                        AKVAVLGASGGIGQPL
                        AKVAVLGASGGIGQPLSL
                        AKVAVLGASGGIGQPLSLLLKN
                        AKVAVLGASGGIGQPLSLLLKNSPLV
                         KVAVLGASGGIGQPLSLLLKN
                          VAVLGASGGIGQPLSLLLK
                          VAVLGASGGIGQPLSLLLKNSPLV
                            VLGASGGIGQPLSLLLK
                             LGASGGIGQPLSLLLK
                              GASGGIGQPLSLLLK
                              GASGGIGQPLSLLLKNSPLV
                                SGGIGQPLSLLLK
                                 GGIGQPLSLLLK
                                 GGIGQPLSLLLKNSPLV
                                    GQPLSLLLKNSPLV
                                     QPLSLLL
                                     QPLSLLLKNSPLV
                                      PLSLLLK
                                      PLSLLLKNSPLV
                                       LSLLLKNSPL
                                       LSLLLKNSPLV
                                        SLLLKNSPLV
                                         LLLKNSPLV
                                             NSPLV
                                             NSPLV
                                             NSPLV
                                             NSPLV
                                             NSPLV
                                             NSPLV
                                             NSPLV
                                             NSPLV
                                              SPLV
                                               PLV
                                                LV
                                                LV
                                                 V

51       60        70        80        90        100
SRLTLYDIAHTPGVAADLSHIETRATVKGYLGPEQLPDCLKGCDVVVIPA
SR
SR
SR
SR
SR
SR
SR
SR
SR
SR
SR
SRLTLYD
SRLTLYDIA
SRLTLYDIAHTPG
SRLTLYDIAHTPGVA
SRLTLYDIAHTPGVAADLSHIET
SRLTLYDIAHTPGVAADLSHIETR
SRLTLYDIAHTPGVAADLSHIETRATVK
SRLTLYDIAHTPGVAADLSHIETR
```

```
51       60        70        80        90        100
SRLTLYDIAHTPGVAADLSHIETRATVKGYLGPEQLPDCLKGCDVVVIPA
SRLTLYDIAHTPGVAADLSHIETR
SRLTLYD
SRLTLYDIAHTPGVAADLSHIETR
SRLTLYDIAHTPGVAADLSHIETR
  LTLYDIAH
  LTLYDIAHTP
  LTLYDIAHTPG
  LTLYDIAHTPGVA
  LTLYDIAHTPGVAA
  LTLYDIAHTPGVAAD
  LTLYDIAHTPGVAADLS
  LTLYDIAHTPGVAADLSHIET
  LTLYDIAHTPGVAADLSHIETR
  LTLYDIAHTPGVAADLSHIETRATVK
   TLYDIAHTPGVAADLSHIETR
   TLYDIAHTPGVAADLSHIETRATVK
    LYDIAHTPGVA
    LYDIAHTPGVAADLSHIETR
    LYDIAHTPGVAADLSHIETRATVK
     YDIAHTPGVAADLSHIETR
     YDIAHTPGVAADLSHIETRATVK
      DIAHTPGVAADLS
      DIAHTPGVAADLSHIETR
      DIAHTPGVAADLSHIETRATVK
       IAHTPGVAADLSHIETR
         HTPGVAADLSHIETR
           PGVAADLSHIETR
            GVAADLSHIETR
             VAADLSHIETR
              AADLSHIETR
               ADLSHIETR
                        ATVKGYLGPEQLPD
                        ATVKGYLGPEQLPDCLK
                        ATVKGYLGPEQLPDCLKGCDVVVIPA
                        ATVKGYLGPEQLPDCLKGCDVVVIPA
                            GYLGPEQLPDCLKGCDVVVIPA

101      110       120       130       140       150
GVPRKPGMTRDDLFNTNATIVATLTAACAQHCPDAMICIISNPVNSTIPI
GVPR
GVPRKPGMTR
GVPRKPGMTR
                                          PVNSTIPI

151      160       170       180       190       200
TAEVFKKHGVYNPNKIFGVTTLDIVRANAFVAELKGLDPARVSVPVIGGH
TAEVFKK
      KHGVYNPNKIFGVTTLDIVR
       HGVYNPNKIFGVTTLDIVR
       HGVYNPNKIFGVTTLDIVRANAFVAELK
       HGVYNPNKIFGVTTLDIVRANAFVAELKGLDPAR
        GVYNPNKIFGVTTLDIVR
         VYNPNKIFGVTTLDIVR
           NPNKIFGVTTLDIVR
              KIFGVTTLDIVR
               IFGVTTLDIVR
                          ANAFVAELK
                          ANAFVAELKGLD
                          ANAFVAELKGLDPAR
                          ANAFVAELKGLDPARVSVPVIGGH
                           NAFVAELKGLDPAR
                            AFVAELK
```

```
151      160       170       180       190       200
TAEVFKKHGVYNPNKIFGVTTLDIVRANAFVAELKGLDPARVSVPVIGGH
                            AFVAELKGLDPAR
                            AFVAELKGLDPARVSVPVIGGH
                              VAELKGLDPAR
                              VAELKGLDPARVSVPVIGGH
                               AELKGLDPAR
                                   GLDPARVSVPVIGGH
                                   GLDPARVSVPVIGGH
                                     DPARVSVPVIGGH
                                         VSVPVIGGH
                                         VSVPVIGGH
                                           VPVIGGH
                                            PVIGGH
                                            PVIGGH

201      210       220       230       240       250
AGKTIIPLISQCTPKVDFPQDQLSTLTGRIQEAGTEVVKAKAGAGSATLS
AGK
AGK
AGK
AGK
AGKTIIPLISQCTPK
AGK
AGK
AGKTIIPLISQCTPK
AGK
AGK
AGKTIIPLISQCTPK
   TIIPLISQCTPK
   TIIPLISQCTPKVD
   TIIPLISQCTPKVDFPQDQLSTLTGR
   TIIPLISQCTPKVDFPQDQLSTLTGRIQEAGTEVVK
     IPLISQCTPK
      PLISQCTPK
      PLISQCTPKVDFPQDQLSTLTGR
          QCTPKVDFPQDQLSTLTGR
               VDFPQDQLSTLTGR
               VDFPQDQLSTLTGRIQEAGTEVVK
               VDFPQDQLSTLTGRIQEAGTEVVKAK
                 FPQDQLSTLTGR
                 FPQDQLSTLTGRIQEAGTEVVK
                 FPQDQLSTLTGRIQEAGTEVVKAK
                  PDQLSTLTGR
                  PQDQLSTLTGRIQEAGTEVVKAK
                    DQLSTLTGR
                     QLSTLTGR
                          TGRIQEAGTEVVK
                          TGRIQEAGTEVVKAK
                             IQEAGTEVV
                             IQEAGTEVVK
                             IQEAGTEVVKA
                             IQEAGTEVVKAK
                             IQEAGTEVVKAKAGAGSATLS
                              QEAGTEVVK
                              QEAGTEVVKAK
                               EAGTEVVKAK
                               EAGTEVVKAKAGAGSATLS
                                AGTEVVKAK
                                       AKAGAGSATLS
                                       AKAGAGSATLS
                                       AKAGAGSATLS
                                        KAGAGSATLS
                                         AGAGSATLS
```

```
   201      210       220       230       240       250
AGKTIIPLISQCTPKVDFPQDQLSTLTGRIQEAGTEVVKAKAGAGSATLS
                                         AGAGSATLS
                                         AGAGSATLS
                                         AGAGSATLS
                                          GAGSATLS
                                           AGSATLS
                                            GSATLS
                                             SATLS
                                              ATLS
                                               TLS
                                                LS
                                                 S

251      260       270       280       290       300
MAYAGARFVFSLVDAMNGKEGVVECSFVKSQETDCPYFSTPLLLGKKGIE
MAYAGAR
MAYAGAR
MAY
MAYAGAR
MAYAGAR
MAY
MAYA
MAYAGAR
MAY
MAYA
MAYAGA
MAYAGAR
MAYAGAR
MAYAGAR
MAYAGAR
MAYAGAR
MAYAGAR
MAYAGAR
MAYAGAR
MAYAGAR
MAYAGAR
       FVFSLVDAMNGK
       FVFSLVDAMNGKEGVVECSFVK
       FVFSLVDAMNGKEGVVECSFVKSQETD
                 GKEGVVECSFVK
                   EGVVECSFVK
                   EGVVECSFVKSQETD
                   EGVVECSFVKSQETDCPYFSTPLLLGK
                   EGVVECSFVKSQETDCPYFSTPLLLGKK
                             SQETDCPYFSTPLLLGK
                             SQETDCPYFSTPLLLGKK
                             SQETDCPYFSTPLLLGKKGIE
                             SQETDCPYFSTPLLLGKKGIE
                             SQETDCPYFSTPLLLGKKGIE
                               ETDCPYFSTPLLLGK
                               ETDCPYFSTPLLLGKK
                                TDCPYFSTPLLLGK
                                TDCPYFSTPLLLGKK
                                  CPYFSTPLLLGK
                                  CPYFSTPLLLGKK
                                   PYFSTPLLLGK
                                   PYFSTPLLLGKK
                                   PYFSTPLLLGKKGIE
                                    YFSTPLLLGK
                                     FSTPLLLGK
                                     FSTPLLLGKK
                                     FSTPLLLGKKGIE
                                      STPLLLGKKGIE
```

```
251      260       270       280       290       300
MAYAGARFVFSLVDAMNGKEGVVECSFVKSQETDCPYFSTPLLLGKKGIE
                                       TPLLLGKKGIE
                                        PLLLGKKGIE
                                          LLGKKGIE
                                          LLGKKGIE
                                           LGKKGIE
                                            GKKGIE
                                              KGIE
                                              KGIE
                                              KGIE
                                              KGIE
                                              KGIE
                                              KGIE
                                              KGIE
                                              KGIE
                                              KGIE
                                               GIE
                                               GIE
                                               GIE
                                               GIE
                                               GIE
                                               GIE
                                               GIE
                                               GIE
                                               GIE
                                               GIE
                                                IE
                                                IE
                                                 E
                                                 E
                                                 E

301      310       320       330     338
KNLGIGKISPFEEKMIAEAIPELKASIKKGEEFVKNMK
K
KNLGIGK
KNLGIGKISPFEEK
K
K
K
K
K
KNLGIGK
KNLGIGKISPFEEK
KNLGIGK
KNLGIGKISPFEEK
KNLGIGK
KNLGIGKISPF
KNLGIGKISPFEEK
KNLGIGKISPFEEKMIA
KNLGIGKISPFEEKMIAE
KNLGIGKISPFEEKMIAEAIPELK
KNLGIGKISPFEEKMIAEAIPELKASIK
KNLGIGKISPFEEKMIAEAIPELKASIKK
KNLGIG
KNLGIGK
KNLGIGKISPF
KNLGIGKISPFE
KNLGIGKISPFEE
KNLGIGKISPFEEK
KNLGIGKISPFEEKM
KNLGIGKISPFEEKMIAE
KNLGIGKISPFEEKMIAEAIPELK
```

```
301      310       320       330     338
KNLGIGKISPFEEKMIAEAIPELKASIKKGEEFVKNMK
KNLGIGKISPFEEKMIAEAIPELKASIK
KNLGIGK
KNLGIGKISPFEEK
KNLGIGK
KNLGIGKISPFEEK
KNLGIGKISPFEEKMIAEAIPELKASIK
KNLGIGK
KNLGIGKISPFEEK
 NLGIGKISPF
 NLGIGKISPFEE
 NLGIGKISPFEEK
 NLGIGKISPFEEKM
 NLGIGKISPFEEKMIA
 NLGIGKISPFEEKMIAEAIPELK
 NLGIGKISPFEEKMIAEAIPELKASIK
 NLGIGKISPFEEKMIAEAIPELKASIKK
 NLGIGKISPFEEKMIAEAIPELKASIKKGEEFVK
 NLGIGKISPFEEKMIAEAIPELKASIKKGEEFVKNM
  LGIGKISPFE
  LGIGKISPFEE
  LGIGKISPFEEK
   GIGKISPFEEK
   GIGKISPFEEKMIAEAIPELK
   GIGKISPFEEKMIAEAIPELKASIK
    IGKISPFEEK
     GKISPFEEK
     GKISPFEEKMIAEAIPELK
      KISPFEEK
      KISPFEEKM
      KISPFEEKMIA
       ISPFEEK
       ISPFEEKM
       ISPFEEKMIA
       ISPFEEKMIAE
       ISPFEEKMIAEAIPELK
       ISPFEEKMIAEAIPELKASIK
       ISPFEEKMIAEAIPELKASIKKGEEFVK
       ISPFEEKMIAEAIPELKASIKKGEEFVKNM
       ISPFEEKMIAEAIPELKASIKKGEEFVKNMK
        SPFEEKMIAEAIPELKASIK
        SPFEEKMIAEAIPELKASIK
        SPFEEKMIAEAIPELKASIKKGEEFVK
         PFEEKMIAEAIPELK
         PFEEKMIAEAIPELKASIK
         PFEEKMIAEAIPELKASIKKGEEFVKNMK
           EEKMIAEAIPELK
             KMIAEAIPELK
              MIAEAIPELK
              MIAEAIPELKA
              MIAEAIPELKASIK
              MIAEAIPELKASIKK
              MIAEAIPELKASIKKG
              MIAEAIPELKASIKKGE
              MIAEAIPELKASIKKGEE
              MIAEAIPELKASIKKGEEFVK
              MIAEAIPELKASIKKGEEFVKNM
              MIAEAIPELKASIKKGEEFVKNMK
               IAEAIPELKASIK
               IAEAIPELKASIKKGEEFVK
               IAEAIPELKASIKKGEEFVKNMK
                AEAIPELK
                AEAIPELKASIK
```

```
301      310       320       330     338
KNLGIGKISPFEEKMIAEAIPELKASIKKGEEFVKNMK
                AEAIPELKASIKKGEEFVK
                AEAIPELKASIKKGEEFVKNMK
                 EAIPELK
                 EAIPELKASIK
                 EAIPELKASIKKGEEFVK
                 EAIPELKASIKKGEEFVKNMK
                  AIPELKASIK
                  AIPELKASIKKGEEFVK
                  AIPELKASIKKGEEFVKNMK
                   IPELKASIK
                   IPELKASIKKGEEFVK
                   IPELKASIKKGEEFVKNMK
                    PELKASIK
                    PELKASIKK
                    PELKASIKKGEEFVK
                    PELKASIKKGEEFVKNMK
                     ELKASIKKGEEFVKNMK
                       KASIKKGEEFVKNMK
                        ASIKKGEEFV
                        ASIKKGEEFVK
                        ASIKKGEEFVKNMK
                         SIKKGEEFVK
                         SIKKGEEFVKNM
                         SIKKGEEFVKNMK
                          IKKGEEFVK
                          IKKGEEFVKNM
                          IKKGEEFVKNMK
                           KKGEEFVK
                           KKGEEFVKNMK
                            KGEEFVK
                            KGEEFVKN
                            KGEEFVKNM
                            KGEEFVKNMK
                             GEEFVKNM
                             GEEFVKNMK
                              EEFVKNMK
```

Denatured MDH 300 sec

```
1        10        20        30        40        50
MLSALARPAGAALRRSFSTSAQNNAKVAVLGASGGIGQPLSLLLKNSPLV
                        AKVAVLGASGGIG
                        AKVAVLGASGGIGQP
                        AKVAVLGASGGIGQPL
                        AKVAVLGASGGIGQPLSLLL
                        AKVAVLGASGGIGQPLSLLLKNSPLV
                         KVAVLGASGGIG
                         KVAVLGASGGIGQPLSLLLKN
                          VAVLGASGGIGQPLSLLLK
                          VAVLGASGGIGQPLSLLLKNSPLV
                            VLGASGGIGQPLSLLLK
                              GASGGIGQPLSLLLK
                                SGGIGQPLSLLLK
                                 GGIGQPLSLLLK
                                 GGIGQPLSLLLKNSPLV
                                    GQPLSLLLKNSPLV
                                     QPLSLLL
                                     QPLSLLLKNSPLV
                                      PLSLLLK
                                      PLSLLLKNSPLV
                                       LSLLLKNSPL
                                       LSLLLKNSPLV
                                        SLLLKNSPLV
                                         LLLKNSPLV
                                             NSPLV
                                             NSPLV
                                             NSPLV
                                             NSPLV
                                             NSPLV
                                             NSPLV
                                             NSPLV
                                              SPLV
                                               PLV
                                                LV
                                                 V

51       60        70        80        90        100
SRLTLYDIAHTPGVAADLSHIETRATVKGYLGPEQLPDCLKGCDVVVIPA
SR
SR
SR
SR
SR
SR
SR
SR
SR
SR
SR
SRLTLYD
SRLTLYDIA
SRLTLYDIAHTPG
SRLTLYDIAHTPGVAADLSHIET
SRLTLYDIAHTPGVAADLSHIETR
SRLTLYDIAHTPGVAADLSHIETRATVK
SRLTLYDIAHTPGVAADLSHIETR
SRLTLYDIAHTPGVAADLSHIETR
SRLTLYDIAHTPGVAADLSHIETR
SRLTLYDIAHTPGVAADLSHIETR
  LTLYDIAH
  LTLYDIAHTP
```

```
51       60        70        80        90        100
SRLTLYDIAHTPGVAADLSHIETRATVKGYLGPEQLPDCLKGCDVVVIPA
  LTLYDIAHTPG
  LTLYDIAHTPGVA
  LTLYDIAHTPGVAA
  LTLYDIAHTPGVAAD
  LTLYDIAHTPGVAADLSHIET
  LTLYDIAHTPGVAADLSHIETR
  LTLYDIAHTPGVAADLSHIETRATVK
   TLYDIAHTPGVAADLSHIETR
   TLYDIAHTPGVAADLSHIETRATVK
    LYDIAHTPGVA
    LYDIAHTPGVAADLSHIETR
    LYDIAHTPGVAADLSHIETRATVK
     YDIAHTPGVAADLSHIETR
     YDIAHTPGVAADLSHIETRATVK
      DIAHTPGVAADLS
      DIAHTPGVAADLSHIETR
       IAHTPGVAADLSHIETR
        AHTPGVAADLSHIETR
         HTPGVAADLSHIETR
          TPGVAADLSHIETR
           PGVAADLSHIETR
            GVAADLSHIETR
             VAADLSHIETR
              AADLSHIETR
               ADLSHIETR
                        ATVKGYLGPEQLPD
                        ATVKGYLGPEQLPDCLK
                        ATVKGYLGPEQLPDCLKGCDVVVIPA
                        ATVKGYLGPEQLPDCLKGCDVVVIPA
                            GYLGPEQLPDCLK
                            GYLGPEQLPDCLKGCDVVVIPA
                            GYLGPEQLPDCLKGCDVVVIPA
                               GPEQLPDCLK
                                         GCDVVVIPA

101      110       120       130       140       150
GVPRKPGMTRDDLFNTNATIVATLTAACAQHCPDAMICIISNPVNSTIPI
GVPR
GVPRKPGMTR
GVPR
GVPRKPGMTR
GVPR
                                          PVNSTIPI

151      160       170       180       190       200
TAEVFKKHGVYNPNKIFGVTTLDIVRANAFVAELKGLDPARVSVPVIGGH
TAEVFKK
      KHGVYNPNKIFGVTTLDIVR
      KHGVYNPNKIFGVTTLDIVRANAFVAELKGLDPAR
       HGVYNPNKIFGVTTLDIVR
       HGVYNPNKIFGVTTLDIVRANAFVAELK
       HGVYNPNKIFGVTTLDIVRANAFVAELKGLDPAR
        GVYNPNKIFGVTTLDIVR
         VYNPNKIFGVTTLDIVR
           NPNKIFGVTTLDIVR
              KIFGVTTLDIVR
               IFGVTTLDIVR
                          ANAFVAELK
                          ANAFVAELKGLD
                          ANAFVAELKGLDPAR
                          ANAFVAELKGLDPARVSVPVIGGH
                           NAFVAELKGLDPAR
```

```
151      160       170       180       190       200
TAEVFKKHGVYNPNKIFGVTTLDIVRANAFVAELKGLDPARVSVPVIGGH
                           NAFVAELKGLDPAR
                           NAFVAELKGLDPARVSVPVIGGH
                            AFVAELK
                            AFVAELKGLDPAR
                            AFVAELKGLDPARVSVPVIGGH
                              VAELKGLDPARVSVPVIGGH
                               AELKGLDPAR
                                   GLDPARVSVPVIGGH
                                   GLDPARVSVPVIGGH
                                     DPARVSVPVIGGH
                                      PARVSVPVIGGH
                                         VSVPVIGGH
                                         VSVPVIGGH
                                          SVPVIGGH
                                          SVPVIGGH
                                           VPVIGGH
                                            PVIGGH
                                            PVIGGH

201      210       220       230       240       250
AGKTIIPLISQCTPKVDFPQDQLSTLTGRIQEAGTEVVKAKAGAGSATLS
AGK
AGK
AGK
AGK
AGK
AGKTIIPLISQCTPK
AGK
AGK
AGK
AGKTIIPLISQCTPK
AGK
AGKTIIPLISQCTPK
AGK
AGK
AGK
   TIIPLISQ
   TIIPLISQCTPK
   TIIPLISQCTPKVD
   TIIPLISQCTPKVDFPQDQLSTLTGR
   TIIPLISQCTPKVDFPQDQLSTLTGRIQEAGTEVVK
     IPLISQCTPK
      PLISQCTPK
      PLISQCTPKVDFPQDQLSTLTGR
        ISQCTPKVDFPQDQLSTLTGR
          QCTPKVDFPQDQLSTLTGR
               VDFPQDQLSTLTGR
               VDFPQDQLSTLTGRIQEAGTEVVK
               VDFPQDQLSTLTGRIQEAGTEVVKAK
                 FPQDQLSTLTGR
                 FPQDQLSTLTGRIQEAGTEVVK
                 FPQDQLSTLTGRIQEAGTEVVKAK
                  PDQLSTLTGR
                  PQDQLSTLTGRIQEAGTEVVKAK
                    DQLSTLTGR
                     QLSTLTGR
                          TGRIQEAGTEVVK
                          TGRIQEAGTEVVKAK
                            RIQEAGTEVVKAK
                             IQEAGTEVV
                             IQEAGTEVVK
                             IQEAGTEVVKA
```

```
201       210       220       230       240       250
AGKTIIPLISQCTPKVDFPQDQLSTLTGRIQEAGTEVVKAKAGAGSATLS
                             IQEAGTEVVKAK
                             IQEAGTEVVKAKAGAGSATLS
                              QEAGTEVVK
                              QEAGTEVVKAK
                               EAGTEVVK
                               EAGTEVVKAK
                               EAGTEVVKAKAGAGSATLS
                                AGTEVVK
                                AGTEVVKAK
                                       AKAGAGSATLS
                                       AKAGAGSATLS
                                       AKAGAGSATLS
                                       AKAGAGSATLS
                                        KAGAGSATLS
                                         AGAGSATLS
                                         AGAGSATLS
                                         AGAGSATLS
                                         AGAGSATLS
                                          GAGSATLS
                                           AGSATLS
                                            GSATLS
                                             SATLS
                                              ATLS
                                               TLS
                                                LS
                                                 S

251       260       270       280       290       300
MAYAGARFVFSLVDAMNGKEGVVECSFVKSQETDCPYFSTPLLLGKKGIE
MAYAGAR
MAYAGAR
MAY
MAYAGA
MAYAGAR
MAYAGAR
MAY
MAYA
MAYAGA
MAYAGAR
MAYAGAR
MAYAGAR
MAYAGAR
MAYAGAR
MAYAGAR
MAYAGAR
MAYAGAR
MAYAGAR
MAYAGAR
       FVFSLVDAMNGK
       FVFSLVDAMNGKEGVVECSFVK
       FVFSLVDAMNGKEGVVECSFVKSQETD
                 GKEGVVECSFVK
                   EGVVECSFVK
                   EGVVECSFVKSQETD
                   EGVVECSFVKSQETDCPYFSTPLLLGK
                   EGVVECSFVKSQETDCPYFSTPLLLGKK
                             SQETDCPYFSTPLLLGK
                             SQETDCPYFSTPLLLGKK
                             SQETDCPYFSTPLLLGKKGIE
                             SQETDCPYFSTPLLLGKKGIE
                             SQETDCPYFSTPLLLGKKGIE
                              QETDCPYFSTPLLLGKK
```

```
251      260       270       280       290       300
MAYAGARFVFSLVDAMNGKEGVVECSFVKSQETDCPYFSTPLLLGKKGIE
                               ETDCPYFSTPLLLGK
                               ETDCPYFSTPLLLGKK
                               ETDCPYFSTPLLLGKKGIE
                                TDCPYFSTPLLLGKK
                                  CPYFSTPLLLGK
                                  CPYFSTPLLLGKK
                                   PYFSTPLLLGK
                                   PYFSTPLLLGKK
                                   PYFSTPLLLGKKGIE
                                    YFSTPLLLGK
                                    YFSTPLLLGKKGIE
                                     FSTPLLLGK
                                     FSTPLLLGKK
                                     FSTPLLLGKKGIE
                                      STPLLLGKKGIE
                                       TPLLLGKKGIE
                                        PLLLGKKGIE
                                          LLGKKGIE
                                          LLGKKGIE
                                           LGKKGIE
                                            GKKGIE
                                              KGIE
                                              KGIE
                                              KGIE
                                              KGIE
                                              KGIE
                                              KGIE
                                              KGIE
                                              KGIE
                                              KGIE
                                               GIE
                                               GIE
                                               GIE
                                               GIE
                                               GIE
                                               GIE
                                               GIE
                                               GIE
                                               GIE
                                               GIE
                                                IE
                                                IE
                                                 E
                                                 E
                                                 E
                                                 E

301      310       320       330     338
KNLGIGKISPFEEKMIAEAIPELKASIKKGEEFVKNMK
K
KNLGIGK
KNLGIGKISPFEEK
K
K
K
K
K
K
K
KNLGIGK
KNLGIGKISPFEEK
KNLGIGK
```

```
301      310       320       330     338
KNLGIGKISPFEEKMIAEAIPELKASIKKGEEFVKNMK
KNLGIGKISPFEEK
KNLGIGK
KNLGIGKISPF
KNLGIGKISPFEEK
KNLGIGKISPFEEKM
KNLGIGKISPFEEKMIA
KNLGIGKISPFEEKMIAE
KNLGIGKISPFEEKMIAEAIPELK
KNLGIGKISPFEEKMIAEAIPELKASIK
KNLGIGKISPFEEKMIAEAIPELKASIKK
KNLGIG
KNLGIGK
KNLGIGKISPF
KNLGIGKISPFE
KNLGIGKISPFEE
KNLGIGKISPFEEK
KNLGIGKISPFEEKM
KNLGIGKISPFEEKMIAE
KNLGIGKISPFEEKMIAEAIPELK
KNLGIGKISPFEEKMIAEAIPELKASIK
KNLGIGK
KNLGIGKISPFEEK
KNLGIGK
KNLGIGKISPFEEK
KNLGIGKISPFEEKMIAEAIPELK
KNLGIGKISPFEEKMIAEAIPELKASIK
KNLGIGK
KNLGIGKISPFEEK
 NLGIGKISPF
 NLGIGKISPFEE
 NLGIGKISPFEEK
 NLGIGKISPFEEKM
 NLGIGKISPFEEKMIA
 NLGIGKISPFEEKMIAE
 NLGIGKISPFEEKMIAEAIPELK
 NLGIGKISPFEEKMIAEAIPELKASIK
 NLGIGKISPFEEKMIAEAIPELKASIKK
 NLGIGKISPFEEKMIAEAIPELKASIKKGEEFVK
 NLGIGKISPFEEKMIAEAIPELKASIKKGEEFVKNM
  LGIGKISPFE
  LGIGKISPFEE
  LGIGKISPFEEK
  LGIGKISPFEEKMIAEAIPELKASIK
   GIGKISPFEEK
   GIGKISPFEEKMIAEAIPELK
   GIGKISPFEEKMIAEAIPELKASIK
   GIGKISPFEEKMIAEAIPELKASIKKGEEFVK
    IGKISPFEEK
    IGKISPFEEKMIAEAIPELK
     GKISPFEEK
     GKISPFEEKMIAEAIPELK
      KISPFEEK
      KISPFEEKM
      KISPFEEKMIA
      KISPFEEKMIAE
       ISPFEEK
       ISPFEEKM
       ISPFEEKMIA
       ISPFEEKMIAE
       ISPFEEKMIAEAIPELK
       ISPFEEKMIAEAIPELKASIK
       ISPFEEKMIAEAIPELKASIKK
```

```
301      310       320       330     338
KNLGIGKISPFEEKMIAEAIPELKASIKKGEEFVKNMK
       ISPFEEKMIAEAIPELKASIKKGEEFVK
       ISPFEEKMIAEAIPELKASIKKGEEFVKNM
       ISPFEEKMIAEAIPELKASIKKGEEFVKNMK
        SPFEEKMIAEAIPELK
        SPFEEKMIAEAIPELKASIK
        SPFEEKMIAEAIPELKASIKKGEEFVK
        SPFEEKMIAEAIPELKASIKKGEEFVK
        SPFEEKMIAEAIPELKASIKKGEEFVKNMK
         PFEEKMIAEAIPELK
         PFEEKMIAEAIPELKASIK
         PFEEKMIAEAIPELKASIKKGEEFVKNMK
          FEEKMIAEAIPELK
           EEKMIAEAIPELK
            EKMIAEAIPELKASIK
             KMIAEAIPELK
             KMIAEAIPELKASIK
             KMIAEAIPELKASIKKGEEFVK
              MIAEAIPELK
              MIAEAIPELKA
              MIAEAIPELKASI
              MIAEAIPELKASIK
              MIAEAIPELKASIKK
              MIAEAIPELKASIKKG
              MIAEAIPELKASIKKGE
              MIAEAIPELKASIKKGEEFV
              MIAEAIPELKASIKKGEEFVK
              MIAEAIPELKASIKKGEEFVKNM
              MIAEAIPELKASIKKGEEFVKNMK
               IAEAIPELK
               IAEAIPELKASIK
               IAEAIPELKASIKKGEEFVK
               IAEAIPELKASIKKGEEFVKNMK
                AEAIPELK
                AEAIPELKASIK
                AEAIPELKASIKK
                AEAIPELKASIKKGEEFVK
                AEAIPELKASIKKGEEFVKNMK
                 EAIPELK
                 EAIPELKASIK
                 EAIPELKASIKKGEEFVK
                 EAIPELKASIKKGEEFVKNMK
                  AIPELKASIK
                  AIPELKASIKKGEEFVK
                  AIPELKASIKKGEEFVKNMK
                   IPELKASIK
                   IPELKASIKKGEEFVK
                   IPELKASIKKGEEFVKNMK
                    PELKASIK
                    PELKASIKK
                    PELKASIKKGEEFVK
                    PELKASIKKGEEFVKNMK
                     ELKASIKKGEEFVKNMK
                        ASIKKGEEFV
                        ASIKKGEEFVK
                        ASIKKGEEFVKNM
                        ASIKKGEEFVKNMK
                         SIKKGEEFVK
                         SIKKGEEFVKNM
                         SIKKGEEFVKNMK
                          IKKGEEFVK
                          IKKGEEFVKNM
                          IKKGEEFVKNMK
```

```
301      310       320       330     338
KNLGIGKISPFEEKMIAEAIPELKASIKKGEEFVKNMK
                           KKGEEFVK
                           KKGEEFVKNMK
                            KGEEFVK
                            KGEEFVKN
                            KGEEFVKNM
                            KGEEFVKNMK
                             GEEFVKNM
                             GEEFVKNMK
                              EEFVKNMK
```

Denatured MDH 600 sec

```
1        10        20        30        40        50
MLSALARPAGAALRRSFSTSAQNNAKVAVLGASGGIGQPLSLLLKNSPLV
                        AKVAVLGASGGIG
                        AKVAVLGASGGIGQPL
                        AKVAVLGASGGIGQPLSLLL
                        AKVAVLGASGGIGQPLSLLLKNSPLV
                          VAVLGASGGIGQPLSLLLK
                          VAVLGASGGIGQPLSLLLKNSPLV
                            VLGASGGIGQPLSLLLK
                             LGASGGIGQPLSLLLK
                              GASGGIGQPLSLLLK
                                SGGIGQPLSLLLK
                                 GGIGQPLSLLLK
                                    GQPLSLLLKNSPLV
                                     QPLSLLLK
                                      PLSLLLK
                                      PLSLLLKNSPLV
                                       LSLLLKNSPLV
                                        SLLLKNSPLV
                                         LLLKNSPLV
                                             NSPLV
                                             NSPLV
                                             NSPLV
                                             NSPLV
                                             NSPLV
                                             NSPLV
                                               PLV
                                                LV

51       60        70        80        90        100
SRLTLYDIAHTPGVAADLSHIETRATVKGYLGPEQLPDCLKGCDVVVIPA
SR
SR
SR
SR
SR
SR
SR
SR
SR
SRLT
SRLTLYD
SRLTLYDIAHTPGVAADLSHIET
SRLTLYDIAHTPGVAADLSHIETR
SRLTLYDIAHTPGVAADLSHIETRATVK
SRLTLYDIAHTPGVAADLSHIETR
SRLTLYDIAHTPGVAADLSHIETR
  LTLYDIAH
  LTLYDIAHTP
  LTLYDIAHTPG
  LTLYDIAHTPGVA
  LTLYDIAHTPGVAA
  LTLYDIAHTPGVAAD
  LTLYDIAHTPGVAADLS
  LTLYDIAHTPGVAADLSHIET
  LTLYDIAHTPGVAADLSHIETR
  LTLYDIAHTPGVAADLSHIETRATVK
   TLYDIAHTPGVAADLSHIETR
   TLYDIAHTPGVAADLSHIETRATVK
    LYDIAHTPGVA
    LYDIAHTPGVAADLSHIETR
    LYDIAHTPGVAADLSHIETRATVK
```

```
51       60        70        80        90        100
SRLTLYDIAHTPGVAADLSHIETRATVKGYLGPEQLPDCLKGCDVVVIPA
     YDIAHTPGVAADLSHIETR
     YDIAHTPGVAADLSHIETRATVK
      DIAHTPGVAADLSHIETR
      DIAHTPGVAADLSHIETRATVK
       IAHTPGVAADLSHIETR
        AHTPGVAADLSHIETR
         HTPGVAADLSHIETR
          TPGVAADLSHIETR
           PGVAADLSHIETR
            GVAADLSHIETR
             VAADLSHIETR
              AADLSHIETR
               ADLSHIETR
                        ATVKGYLGPEQLPD
                        ATVKGYLGPEQLPDCLK
                        ATVKGYLGPEQLPDCLKGCDVVVIPA
                        ATVKGYLGPEQLPDCLKGCDVVVIPA
                            GYLGPEQLPDCLK
                            GYLGPEQLPDCLKGCDVVVIPA
                            GYLGPEQLPDCLKGCDVVVIPA
                               GPEQLPDCLK
                                PEQLPDCLK
                                         GCDVVVIPA
                                                PA

101      110       120       130       140       150
GVPRKPGMTRDDLFNTNATIVATLTAACAQHCPDAMICIISNPVNSTIPI
GVPR
GVPRKPGMTR
GVPR
GVPRKPGMTR
GVPR
GVPRKPGMTR
                                          PVNSTIPI

151      160       170       180       190       200
TAEVFKKHGVYNPNKIFGVTTLDIVRANAFVAELKGLDPARVSVPVIGGH
TAEVFKK
      KHGVYNPNKIFGVTTLDIVR
      KHGVYNPNKIFGVTTLDIVRANAFVAELKGLDPAR
       HGVYNPNK
       HGVYNPNKIFGVTTLDIVR
       HGVYNPNKIFGVTTLDIVRANAFVAELK
       HGVYNPNKIFGVTTLDIVRANAFVAELKGLDPAR
        GVYNPNKIFGVTTLDIVR
         VYNPNKIFGVTTLDIVR
           NPNKIFGVTTLDIVR
              KIFGVTTLDIVR
               IFGVTTLDIVR
                   TTLDIVR
                          ANAFVAELK
                          ANAFVAELKGLD
                          ANAFVAELKGLDPAR
                          ANAFVAELKGLDPARVSVPVIGGH
                           NAFVAELKGLDPAR
                           NAFVAELKGLDPARVSVPVIGGH
                            AFVAELK
                            AFVAELKGLDPAR
                            AFVAELKGLDPARVSVPVIGGH
                              VAELKGLDPAR
                              VAELKGLDPARVSVPVIGGH
                               AELKGLDPAR
```

```
151      160       170       180       190       200
TAEVFKKHGVYNPNKIFGVTTLDIVRANAFVAELKGLDPARVSVPVIGGH
                               AELKGLDPARVSVPVIGGH
                                   GLDPARVSVPVIGGH
                                   GLDPARVSVPVIGGH
                                     DPARVSVPVIGGH
                                      PARVSVPVIGGH
                                         VSVPVIGGH
                                         VSVPVIGGH
                                          SVPVIGGH
                                          SVPVIGGH
                                           VPVIGGH
                                            PVIGGH
                                            PVIGGH
                                              IGGH

201      210       220       230       240       250
AGKTIIPLISQCTPKVDFPQDQLSTLTGRIQEAGTEVVKAKAGAGSATLS
AGK
AGK
AGK
AGK
AGK
AGK
AGKTIIPLISQCTPK
AGK
AGK
AGK
AGKTIIPLISQCTPK
AGK
AGKTIIPLISQCTPK
AGK
AGK
AGKTIIPLISQCTPK
AGK
   TIIPLISQ
   TIIPLISQCTPK
   TIIPLISQCTPKVD
   TIIPLISQCTPKVDFPQDQLSTLTGR
   TIIPLISQCTPKVDFPQDQLSTLTGRIQEAGTEVVK
     IPLISQCTPK
      PLISQCTPK
      PLISQCTPKVDFPQDQLSTLTGR
        ISQCTPKVDFPQDQLSTLTGR
          QCTPKVDFPQDQLSTLTGR
               VDFPQDQLSTLTGR
               VDFPQDQLSTLTGRIQEAGTEVVK
               VDFPQDQLSTLTGRIQEAGTEVVKAK
                 FPQDQLSTLTGR
                 FPQDQLSTLTGRIQEAGTEVVK
                 FPQDQLSTLTGRIQEAGTEVVKAK
                  PDQLSTLTGR
                  PQDQLSTLTGRIQEAGTEVVKAK
                    DQLSTLTGR
                     QLSTLTGR
                          TGRIQEAGTEVVK
                          TGRIQEAGTEVVKAK
                            RIQEAGTEVVKAK
                             IQEAGTEVV
                             IQEAGTEVVK
                             IQEAGTEVVKA
                             IQEAGTEVVKAK
                             IQEAGTEVVKAKAGAGSATLS
                              QEAGTEVVK
```

```
201      210       220       230       240       250
AGKTIIPLISQCTPKVDFPQDQLSTLTGRIQEAGTEVVKAKAGAGSATLS
                              QEAGTEVVKAK
                               EAGTEVVK
                               EAGTEVVKAK
                               EAGTEVVKAKAGAGSATLS
                                AGTEVVK
                                AGTEVVKAK
                                 GTEVVKAK
                                       AKAGAGSATLS
                                       AKAGAGSATLS
                                       AKAGAGSATLS
                                       AKAGAGSATLS
                                        KAGAGSATLS
                                         AGAGSATLS
                                         AGAGSATLS
                                         AGAGSATLS
                                         AGAGSATLS
                                          GAGSATLS
                                           AGSATLS
                                            GSATLS
                                             SATLS
                                              ATLS
                                               TLS
                                                LS
                                                 S

251      260       270       280       290       300
MAYAGARFVFSLVDAMNGKEGVVECSFVKSQETDCPYFSTPLLLGKKGIE
MAYAGAR
MAYAGAR
MAY
MAYAGA
MAYAGAR
MAYAGAR
MAY
MAYA
MAYAGA
MAYAGAR
MAYAGAR
MAYAGAR
MAYAGAR
MAYAGAR
MAYAGAR
MAYAGAR
MAYAGAR
MAYAGAR
MAYAGAR
       FVFSLVDAMNGK
       FVFSLVDAMNGKEGVVECSFVK
       FVFSLVDAMNGKEGVVECSFVKSQETD
         FSLVDAMNGK
          SLVDAMNGKEGVVECSFVK
                 GKEGVVECSFVK
                   EGVVECSFVK
                   EGVVECSFVKSQETD
                   EGVVECSFVKSQETDCPYFSTPLLLGK
                   EGVVECSFVKSQETDCPYFSTPLLLGKK
                      VECSFVK
                            KSQETDCPYFSTPLLLGK
                             SQETDCPYFSTPLLLGK
                             SQETDCPYFSTPLLLGKK
                             SQETDCPYFSTPLLLGKKGIE
                             SQETDCPYFSTPLLLGKKGIE
```

```
251      260       270       280       290       300
MAYAGARFVFSLVDAMNGKEGVVECSFVKSQETDCPYFSTPLLLGKKGIE
                             SQETDCPYFSTPLLLGKKGIE
                              QETDCPYFSTPLLLGKK
                               ETDCPYFSTPLLLGK
                               ETDCPYFSTPLLLGKK
                               ETDCPYFSTPLLLGKKGIE
                                TDCPYFSTPLLLGKK
                                 DCPYFSTPLLLGKK
                                  CPYFSTPLLLGK
                                  CPYFSTPLLLGKK
                                   PYFSTPLLLGKK
                                   PYFSTPLLLGKKGIE
                                   PYFSTPLLLGKKGIE
                                    YFSTPLLLGKKGIE
                                     FSTPLLLGK
                                     FSTPLLLGKK
                                     FSTPLLLGKKGIE
                                      STPLLLGKKGIE
                                       TPLLLGKKGIE
                                        PLLLGKKGIE
                                          LLGKKGIE
                                          LLGKKGIE
                                           LGKKGIE
                                              KGIE
                                              KGIE
                                              KGIE
                                              KGIE
                                              KGIE
                                              KGIE
                                              KGIE
                                              KGIE
                                              KGIE
                                              KGIE
                                               GIE
                                               GIE
                                               GIE
                                               GIE
                                               GIE
                                               GIE
                                               GIE
                                               GIE
                                               GIE
                                               GIE
                                               GIE
                                                IE
                                                IE
                                                 E
                                                 E
                                                 E
                                                 E

301      310       320       330     338
KNLGIGKISPFEEKMIAEAIPELKASIKKGEEFVKNMK
K
KNLGIGK
KNLGIGKISPFEEK
K
K
KNLGIGKISPFEEK
K
K
K
```

```
301      310       320       330     338
KNLGIGKISPFEEKMIAEAIPELKASIKKGEEFVKNMK
K
K
KNLGIGK
KNLGIGKISPFEEK
KNLGIG
KNLGIGK
KNLGIGKISPF
KNLGIGKISPFEE
KNLGIGKISPFEEK
KNLGIGKISPFEEKM
KNLGIGKISPFEEKMIA
KNLGIGKISPFEEKMIAE
KNLGIGKISPFEEKMIAEAIPELK
KNLGIGKISPFEEKMIAEAIPELKASIK
KNLGIGKISPFEEKMIAEAIPELKASIKK
KNLGIG
KNLGIGK
KNLGIGKISPF
KNLGIGKISPFEE
KNLGIGKISPFEEK
KNLGIGKISPFEEKM
KNLGIGKISPFEEKMIAE
KNLGIGKISPFEEKMIAEAIPELK
KNLGIGKISPFEEKMIAEAIPELKASIK
KNLGIGK
KNLGIGKISPFEEK
KNLGIGK
KNLGIGKISPFEEK
KNLGIGKISPFEEKMIAEAIPELK
KNLGIGKISPFEEKMIAEAIPELKASIK
KNLGIGK
KNLGIGKISPFEEK
 NLGIGKISPF
 NLGIGKISPFEE
 NLGIGKISPFEEK
 NLGIGKISPFEEKM
 NLGIGKISPFEEKMIA
 NLGIGKISPFEEKMIAE
 NLGIGKISPFEEKMIAEAIPELK
 NLGIGKISPFEEKMIAEAIPELKASIK
 NLGIGKISPFEEKMIAEAIPELKASIKK
 NLGIGKISPFEEKMIAEAIPELKASIKKGEEFVK
 NLGIGKISPFEEKMIAEAIPELKASIKKGEEFVKNM
  LGIGKISPFE
  LGIGKISPFEE
  LGIGKISPFEEK
  LGIGKISPFEEKMIAEAIPELKASIK
   GIGKISPFEEK
   GIGKISPFEEKMIAEAIPELK
   GIGKISPFEEKMIAEAIPELKASIK
   GIGKISPFEEKMIAEAIPELKASIKKGEEFVK
    IGKISPFEEK
    IGKISPFEEKMIAEAIPELK
     GKISPFEEK
     GKISPFEEKMIAEAIPELK
      KISPFEEK
      KISPFEEKM
      KISPFEEKMIA
      KISPFEEKMIAE
       ISPFEEK
       ISPFEEKM
       ISPFEEKMIA
```

```
301      310       320       330     338
KNLGIGKISPFEEKMIAEAIPELKASIKKGEEFVKNMK
       ISPFEEKMIAE
       ISPFEEKMIAEAIPELK
       ISPFEEKMIAEAIPELKASIK
       ISPFEEKMIAEAIPELKASIKK
       ISPFEEKMIAEAIPELKASIKKGEEFVK
       ISPFEEKMIAEAIPELKASIKKGEEFVKNM
       ISPFEEKMIAEAIPELKASIKKGEEFVKNMK
        SPFEEKMIAEAIPELK
        SPFEEKMIAEAIPELKASIK
        SPFEEKMIAEAIPELKASIKKGEEFVK
         PFEEKMIAEAIPELK
         PFEEKMIAEAIPELKASIK
         PFEEKMIAEAIPELKASIKKGEEFVK
         PFEEKMIAEAIPELKASIKKGEEFVKNMK
          FEEKMIAEAIPELK
          FEEKMIAEAIPELKASIK
           EEKMIAEAIPELK
             KMIAEAIPELK
             KMIAEAIPELKASIK
             KMIAEAIPELKASIKKGEEFVK
              MIAEAIPELK
              MIAEAIPELKA
              MIAEAIPELKASI
              MIAEAIPELKASIK
              MIAEAIPELKASIKK
              MIAEAIPELKASIKKG
              MIAEAIPELKASIKKGE
              MIAEAIPELKASIKKGEEFV
              MIAEAIPELKASIKKGEEFVK
              MIAEAIPELKASIKKGEEFVKNM
              MIAEAIPELKASIKKGEEFVKNMK
               IAEAIPELK
               IAEAIPELKASIK
               IAEAIPELKASIKKGEEFVK
               IAEAIPELKASIKKGEEFVKNMK
                AEAIPELK
                AEAIPELKASIK
                AEAIPELKASIKK
                AEAIPELKASIKKGEEFVK
                AEAIPELKASIKKGEEFVKNMK
                 EAIPELK
                 EAIPELKASIK
                 EAIPELKASIKKGEEFVK
                 EAIPELKASIKKGEEFVKNMK
                  AIPELKASIK
                  AIPELKASIKKGEEFVK
                  AIPELKASIKKGEEFVKNMK
                   IPELKASIK
                   IPELKASIKKGEEFVK
                   IPELKASIKKGEEFVKNMK
                    PELKASIK
                    PELKASIKK
                    PELKASIKKGEEFVK
                    PELKASIKKGEEFVKNMK
                     ELKASIKKGEEFVKNMK
                        ASIKKGEEFV
                        ASIKKGEEFVK
                        ASIKKGEEFVKNM
                        ASIKKGEEFVKNMK
                         SIKKGEEFVK
                         SIKKGEEFVKNM
                         SIKKGEEFVKNMK
```

```
301      310       320       330     338
KNLGIGKISPFEEKMIAEAIPELKASIKKGEEFVKNMK
                          IKKGEEFVK
                          IKKGEEFVKNM
                          IKKGEEFVKNMK
                           KKGEEFVK
                           KKGEEFVKNMK
                            KGEEFVK
                            KGEEFVKN
                            KGEEFVKNM
                            KGEEFVKNMK
                             GEEFVKNM
                             GEEFVKNMK
                              EEFVKNMK
```

Supporting data 7

CLUSTAL O (1.2.4) multiple sequence alignment of MDHs

```
                                                   1      .         .         .         .         50
 1 tr|A0A1P8BBQ0|ARABIDOPSIS THALIANA   -------MEFRGDANQRIARISAHLTPQMEAKNSVIGRENCRAKGGNPGF
 2 tr|A0A8M1PNU6|DANIO RERIO             -------------MFSRVARPT-------------ASLVRSLSTSSQNNA
 3 tr|A0A6I9JAA7|CHRYSOCHLORIS ASIATICA -------------MLSALARP------------AGATLRRGFSTSVQNNA
 4 tr|A0A8C7EKE8|NEOVISON VISON          -------------MFSALARP------------AGAALRRSFSTSAQKNA
 5 sp|Q5NVR2|PONGO ABELII                -------------MLSALARP------------ASAVLRRSFSTSAQNNA
 6 tr|H2QUS9|PAN TROGLODYTES             -------------MLSALARP------------ASAALRRSFSTSAQNNA
 7 sp|P40926|HOMO SAPIENS                -------------MLSALARP------------ASAALRRSFSTSAQNNA
 8 tr|F6TYW4|EQUUS CABALLUS              -------------MLSAFARP------------AGAALRRSLSTSAQNNA
 9 sp|P00346|SUS SCROFA                  -------------MLSALARP------------AGAALRRSFSTSAQNNA
10 sp|Q32LG3|BOS TAURUS                  -------------MLSALARP------------AGAALRRSFSTSAQNNA
11 sp|P08249|MUS MUSCULUS                -------------MLSALARP------------AGAALRRSFSTSAQNNA
12 sp|P04636|RATTUS NORVEGICUS           -------------MLSALARP------------VGAALRRSFSTSAQNNA
13 tr|A0A8C4WQE8|GOPHERUS EVGOODEI       ------------MLLARLSGPS-----------AAAALRRGIATSAQSNA
14 tr|A0A8C8VKR3|PELUSIOS CASTANEUS      ------------MLLSRLS--R-----------AAAALRRGLATSAQSNA
15 tr|A0A8B9BBN8|ANSER BRACHYRHYNCHUS    MKSKESVVKSPGSLLQSLAK---------------RLFSAFLSFFPQNNA
16 tr|A0A7L2UU21|BALAENICEPS REX         -------------MLSRLARP------------AAAALRRGLATTAQTNA
17 tr|E1BVT3|GALLUS GALLUS               -------------MLSRLARP------------AA-VLCRGLATSAQNNA
18 tr|A0A850X7V7|PIAYA CAYANA            ---------------------------------------------SPQHNA
19 tr|A0A218V2K7|LONCHURA STRIATA        -------------MLSRLSTA------------TAAALRRGIATSAQNNA
20 tr|A0A8C9MRZ4|SERINUS CANARIA         -------------PLCILSVG------------IQASLR---ERGLDNHA
                                                                                :

                                         51     .         .         .         .         100
 1 tr|A0A1P8BBQ0|ARABIDOPSIS THALIANA   KVAILGAAGGIGQSLSLLMKMNPLVSLLHLYDVVNAPGVTADVSHMDTGA
 2 tr|A0A8M1PNU6|DANIO RERIO             KVAVLGASGGIGQPLSLLLKNSPLVSELSLFDIAHTPGVAADLSHIETRA
 3 tr|A0A6I9JAA7|CHRYSOCHLORIS ASIATICA KVAVLGASGGIGQPLSLLLKNSPLVSRLTLYDIAHTPGVAADLSHIETRA
 4 tr|A0A8C7EKE8|NEOVISON VISON          KVAVLGASGGIGQPLALLLKNSPLVSRLTLYDIAHTPGVAADLSHIETRA
 5 sp|Q5NVR2|PONGO ABELII                KVAVLGASGGIGQPLSLLLKNSPLVSRLTLYDIAHTPGVAADLSHIETKA
 6 tr|H2QUS9|PAN TROGLODYTES             KVAVLGASGGIGQPLSLLLKNSPLVSRLTLYDIAHTPGVAADLSHIETKA
 7 sp|P40926|HOMO SAPIENS                KVAVLGASGGIGQPLSLLLKNSPLVSRLTLYDIAHTPGVAADLSHIETKA
 8 tr|F6TYW4|EQUUS CABALLUS              KVAVLGASGGIGQPLSLLLKNSPLVSRLTLYDIAHTPGVAADLSHIETRA
 9 sp|P00346|SUS SCROFA                  KVAVLGASGGIGQPLSLLLKNSPLVSRLTLYDIAHTPGVAADLSHIETRA
10 sp|Q32LG3|BOS TAURUS                  KVAVLGASGGIGQPLSLLLKNSPLVSRLTLYDIAHTPGVAADLSHIETRA
11 sp|P08249|MUS MUSCULUS                KVAVLGASGGIGQPLSLLLKNSPLVSRLTLYDIAHTPGVAADLSHIETRA
12 sp|P04636|RATTUS NORVEGICUS           KVAVLGASGGIGQPLSLLLKNSPLVSRLTLYDIAHTPGVAADLSHIETRA
13 tr|A0A8C4WQE8|GOPHERUS EVGOODEI       KVAVLGASGGIGQPLSLLLKNSPLVSNLSLYDIAHTPGVAADLSHIETRA
14 tr|A0A8C8VKR3|PELUSIOS CASTANEUS      KVAVLGASGGIGQPLSLLLKNSPLVSHLNLYDIAHTPGVAADLSHIETRA
15 tr|A0A8B9BBN8|ANSER BRACHYRHYNCHUS    KVAVLGASGGIGQPLSLLLKNSPLVSRLSLYDIAHTPGVAADLSHIETRA
16 tr|A0A7L2UU21|BALAENICEPS REX         KVAVLGASGGIGQPLSLLLKNSPLVSRLSLYDIAHTPGVAADLSHIETRA
17 tr|E1BVT3|GALLUS GALLUS               KVAVLGASGGIGQPLSLLLKNSPLVSRLTLYDIAHTPGVAADLSHIETRA
18 tr|A0A850X7V7|PIAYA CAYANA            KVAVLGASGGIGQPLSLLLKNSPLVSRLSLYDIAHTPGVAADLSHIETRA
19 tr|A0A218V2K7|LONCHURA STRIATA        KVAVLGASGGIGQPLSLLLKNSPLVSKLSLYDIAHTPGVAADLSHIETRA
20 tr|A0A8C9MRZ4|SERINUS CANARIA         KVAVLGASGGIGQPLSLLLKNSPLVSKLSLYDIAHTPGVAADLSHIETKA
                                         ***:***:***** *:**:* .**** * *:*:.::***:**:**::* *

                                         101    .         .         .         .         150
 1 tr|A0A1P8BBQ0|ARABIDOPSIS THALIANA   VVRGFLGAKQLEDALTGMDLVIIPAGIPRKPGMTRDDLFKINAGIVKTLC
 2 tr|A0A8M1PNU6|DANIO RERIO             HVKGYIGADQLGDALKGCEVVVIPAGVPRKPGMTRDDLFNTNATIVATLV
 3 tr|A0A6I9JAA7|CHRYSOCHLORIS ASIATICA NVKGYLGPDQLPDCLKGCDVVVIPAGVPRKPGMTRDDLFNTNATIVATLA
 4 tr|A0A8C7EKE8|NEOVISON VISON          TVKGYLGPEQLPDCLKGCDVVVIPAGVPRKPGMTRDDLFNTNASIVATLT
 5 sp|Q5NVR2|PONGO ABELII                TVKGYLGPEQLPDCLKGCDVVVIPAGVPRKPGMTRDDLFNTNATIVATLT
 6 tr|H2QUS9|PAN TROGLODYTES             AVKGYLGPEQLPDCLKGCDVVVIPAGVPRKPGMTRDDLFNTNATIVATLT
 7 sp|P40926|HOMO SAPIENS                AVKGYLGPEQLPDCLKGCDVVVIPAGVPRKPGMTRDDLFNTNATIVATLT
 8 tr|F6TYW4|EQUUS CABALLUS              TVKGYLGPEQLPDCLKGCDVVVIPAGVPRKPGMTRDDLFNTNATIVATLT
 9 sp|P00346|SUS SCROFA                  TVKGYLGPEQLPDCLKGCDVVVIPAGVPRKPGMTRDDLFNTNATIVATLT
10 sp|Q32LG3|BOS TAURUS                  TVKGYLGPEQLPDCLKGCDVVVIPAGVPRKPGMTRDDLFNTNATIVATLT
11 sp|P08249|MUS MUSCULUS                NVKGYLGPEQLPDCLKGCDVVVIPAGVPRKPGMTRDDLFNTNATIVATLT
12 sp|P04636|RATTUS NORVEGICUS           NVKGYLGPEQLPDCLKGCDVVVIPAGVPRKPGMTRDDLFNTNATIVATLT
13 tr|A0A8C4WQE8|GOPHERUS EVGOODEI       NVKGFIGSEQLPDCLKGCEVVVIPAGVPRKPGMTRDDLFNTNATIVANLT
14 tr|A0A8C8VKR3|PELUSIOS CASTANEUS      EVKGFIGPEQLPECLKGCEVVVIPAGVPRKPGMTRDDLFNTNATIVANLT
15 tr|A0A8B9BBN8|ANSER BRACHYRHYNCHUS    TVKGFLGPEQLPECLKGCDVVVIPAGVPRKPGMTRDDLFNTNASIVATLT
16 tr|A0A7L2UU21|BALAENICEPS REX         NVKGFLGPEQLPECLKGCDVVVIPAGVPRKPGMTRDDLFNTNASIVATLT
17 tr|E1BVT3|GALLUS GALLUS               NVKGFLGPEQLPECLKGCDVVVIPAGVPRKPGMTRDDLFNTNASIVATLT
18 tr|A0A850X7V7|PIAYA CAYANA            NVKGFLGPEQLPECLKGCDVVVIPAGVPRKPGMTRDDLFNTNASIVATLT
19 tr|A0A218V2K7|LONCHURA STRIATA        NVKGFMGPEQLPECLKGCDVVVIPAGVPRKPGMTRDDLFNTNASIVASLT
20 tr|A0A8C9MRZ4|SERINUS CANARIA         SVKGFMGPEQLPECLKGCDVVVIPAGVPRKPGMTRDDLFNTNASIVASLT
                                          *:*::* .** :.*.* ::*:****:************: ** ** .*

                                         151    .         .         .         .         200
 1 tr|A0A1P8BBQ0|ARABIDOPSIS THALIANA   EGVAKCCPNAIVNLISNPVNSTVPIAAEVFKKAGTYDPKKLLGVTTLDVA
 2 tr|A0A8M1PNU6|DANIO RERIO             DGCARHCPQAMICIISNPVNSTIPITSEVMKKHGVYNPNKIFGVTTLDIV
 3 tr|A0A6I9JAA7|CHRYSOCHLORIS ASIATICA AACAQHCPEAMVCVIANPVNSTIPITAEVFKKHGVYNPNKIFGVTTLDIV
 4 tr|A0A8C7EKE8|NEOVISON VISON          AACAQHCPEAMICIISNPVNSTIPIATEVFKKHGAYNPNKIFGVTTLDIV
 5 sp|Q5NVR2|PONGO ABELII                SACAQHCPEAMICVIANPVNSTIPITAEVFKKHGVYNPNKIFGVTTLDIV
 6 tr|H2QUS9|PAN TROGLODYTES             AACAQHCPEAMICIIANPVNSTIPITAEVFKKHGVYNPNKIFGVTTLDIV
```

```
 7 sp|P40926|HOMO SAPIENS               AACAQHCPEAMICVIANPVNSTIPITAEVFKKHGVYNPNKIFGVTTLDIV
 8 tr|F6TYW4|EQUUS CABALLUS             AACAQHCPEAMVCIISNPVNSTIPITAEVFKKHGVYNPDKIFGVTTLDIV
 9 sp|P00346|SUS SCROFA                 AACAQHCPDAMICIISNPVNSTIPITAEVFKKHGVYNPNKIFGVTTLDIV
10 sp|Q32LG3|BOS TAURUS                 AACAQHCPEAMICIISNPVNSTIPITAEVFKKHGVYNPNKIFGVTTLDIV
11 sp|P08249|MUS MUSCULUS               AACAQHCPEAMVCIIANPVNSTIPITAEVFKKHGVYNPNKIFGVTTLDIV
12 sp|P04636|RATTUS NORVEGICUS          AACAQHCPEAMICIISNPVNSTIPITAEVFKKHGVYNPNKIFGVTTLDIV
13 tr|A0A8C4WQE8|GOPHERUS EVGOODEI      TACAKHCPEAMICIIANPVNSTIPITSEIFKKHGVYNPNRIFGVTTLDIV
14 tr|A0A8C8VKR3|PELUSIOS CASTANEUS     AACAKHSPEAMICIISNPVNSTIPITSEVFKKHGVYNPNRIFGVTTLDIV
15 tr|A0A8B9BBN8|ANSER BRACHYRHYNCHUS   TACAKHCPEAMICIISNPVNSTIPITSEVFKKHGVYNPNRIFGVTTLDIV
16 tr|A0A7L2UU21|BALAENICEPS REX        SACAKHCPEAMICIISNPVNSTIPITSEVFKKHGVYNPNRIFGVTTLDIV
17 tr|E1BVT3|GALLUS GALLUS              TACAKHCPEAMICIISNPVNSTIPITSEVFKKHGVYNPNRIFGVTTLDIV
18 tr|A0A850X7V7|PIAYA CAYANA           TACAKHCPEAMICIISNPVNSTIPITSEVFKKHGVYNPNRIFGVTTLDIV
19 tr|A0A218V2K7|LONCHURA STRIATA       TACAKHCPEAMICIISNPVNSTIPITSEVFKKHGVYNPNKIFGVTTLDIV
20 tr|A0A8C9MRZ4|SERINUS CANARIA        TACAKHCPEAMICIISNPVNSTIPITSEVFKKHGVYNPNKIFGVTTLDIV
                                         . *: .*:*:: :*:******:**::*::** *.*:*.:::******:.

                                        201      .         .         .         .         250
 1 tr|A0A1P8BBQ0|ARABIDOPSIS THALIANA   RANTFA-EVLGLDPREVDVPVVGGHAGVTILPLLSQVKPPSSFTPQEIEY
 2 tr|A0A8M1PNU6|DANIO RERIO            RANTFVAELKGLDPARVNVPVVGGHAGITIIPLISQCTPKVEFPADQLSA
 3 tr|A0A6I9JAA7|CHRYSOCHLORIS ASIATICA RANTFVAELKGLDPARVNVPVIGGHAGKTIIPLISQCTPKVDFPQDQLTA
 4 tr|A0A8C7EKE8|NEOVISON VISON         RANTFIAELKGLDPARVNVPVIGGHAGKTIIPLISQCTPKVDLPQDQLTA
 5 sp|Q5NVR2|PONGO ABELII               RANTFVAELKGLDPARVNVPVIGGHAGKTIIPLISQCTPKVDFPQDQLTA
 6 tr|H2QUS9|PAN TROGLODYTES            RANTFVAELKGLDPARVNVPVIGGHAGKTIIPLISQCTPKVDFPQDQLTA
 7 sp|P40926|HOMO SAPIENS               RANTFVAELKGLDPARVNVPVIGGHAGKTIIPLISQCTPKVDFPQDQLTA
 8 tr|F6TYW4|EQUUS CABALLUS             RANTFVAELKGLDPARVHVPVIGGHAGKTIIPVISQCTPKVDFPQDQLTT
 9 sp|P00346|SUS SCROFA                 RANAFVAELKGLDPARVSVPVIGGHAGKTIIPLISQCTPKVDFPQDQLST
10 sp|Q32LG3|BOS TAURUS                 RANAFVAELKDLDPARVNVPVIGGHAGKTIIPLISQCTPKVEFPQDQLTT
11 sp|P08249|MUS MUSCULUS               RANTFVAELKGLDPARVNVPVIGGHAGKTIIPLISQCTPKVDFPQDQLAT
12 sp|P04636|RATTUS NORVEGICUS          RANTFVAELKGLDPARVNVPVIGGHAGKTIIPLISQCTPKVDFPQDQLAT
13 tr|A0A8C4WQE8|GOPHERUS EVGOODEI      RANTFVAELKGLDPARVNVPVIGGHAGKTIIPLISQCTPKVEFPQDQLET
14 tr|A0A8C8VKR3|PELUSIOS CASTANEUS     RANTFVAELKGLDPARVNVPVVGGHAGKTIIPLISQCTPKVEFPQDQLET
15 tr|A0A8B9BBN8|ANSER BRACHYRHYNCHUS   RANTFVAELKGLDPARVSVPVIGGHAGKTIIPLISQCTPKVDFPQDQLEK
16 tr|A0A7L2UU21|BALAENICEPS REX        RANTFVAELKGLDPARVSVPVIGGHAGKTIIPLISQCTPKVDFPQDQLEK
17 tr|E1BVT3|GALLUS GALLUS              RANTFVAELKGLDPARVSVPVIGGHAGKTIIPLISQCTPKVDFPQDQLEK
18 tr|A0A850X7V7|PIAYA CAYANA           RANTFVAELKGLDPARVSVPVIGGHAGKTIIPLISQCTPKVDFPQDQLEK
19 tr|A0A218V2K7|LONCHURA STRIATA       RANTFVAELKGLDPARVTVPVIGGHAGKTIIPLISQCTPKVEFPQDQLEK
20 tr|A0A8C9MRZ4|SERINUS CANARIA        RANTFVAELKGLDPARVTVPVVGGHAGKTIIPLISQCTPKVEFPQDQLEK
                                        ****:*  *: .*** .* ***:**** **:*::** .* .:  :::

                                        251      .         .         .         .         300
 1 tr|A0A1P8BBQ0|ARABIDOPSIS THALIANA   LTNRIQNGGTEVVEAKAGAGSATLSMAYAAAKFADACLRGLRGDANVVEC
 2 tr|A0A8M1PNU6|DANIO RERIO            LTGRIQEAGTEVVKAKAGAGSATLSMAYAGARFTFSLLDAMNGKEGVVEC
 3 tr|A0A6I9JAA7|CHRYSOCHLORIS ASIATICA LTGRIQEAGTEVVKAKAGAGSATLSMAYAGARFVFSLVDAMNGKQGVIEC
 4 tr|A0A8C7EKE8|NEOVISON VISON         VTGRIQEAGTEVVKAKAGAGSATLSMAYAGARFVFSLVDAMNGKEGVVEC
 5 sp|Q5NVR2|PONGO ABELII               LTGRIQEAGTEVVKAKAGAGSATLSMAYAGARFVFSLVDAMNGKEGVVEC
 6 tr|H2QUS9|PAN TROGLODYTES            LTGRIQEAGTEVVKAKAGAGSATLSMAYAGARFVFSLVDAMNGKEGVVEC
 7 sp|P40926|HOMO SAPIENS               LTGRIQEAGTEVVKAKAGAGSATLSMAYAGARFVFSLVDAMNGKEGVVEC
 8 tr|F6TYW4|EQUUS CABALLUS             LIGRIQEAGTEVVKAKAGAGSATLSMAYAGARFVFSLVDAMNGKEGVVEC
 9 sp|P00346|SUS SCROFA                 LTGRIQEAGTEVVKAKAGAGSATLSMAYAGARFVFSLVDAMNGKEGVVEC
10 sp|Q32LG3|BOS TAURUS                 LTGRIQEAGTEVVKAKAGAGSATLSMAYAGARFVFSLVDAMNGKEGVVEC
11 sp|P08249|MUS MUSCULUS               LTGRIQEAGTEVVKAKAGAGSATLSMAYAGARFVFSLVDAMNGKEGVVEC
12 sp|P04636|RATTUS NORVEGICUS          LTGRIQEAGTEVVKAKAGAGSATLSMAYAGARFVFSLVDAMNGKEGVIEC
13 tr|A0A8C4WQE8|GOPHERUS EVGOODEI      LTGRIQEAGTEVVKAKAGAGSATLSMAYAGARFVFSVLDAMNGKEGVIEC
14 tr|A0A8C8VKR3|PELUSIOS CASTANEUS     LTGRIQEAGTEVVKAKAGAGSATLSMAYAGARFVFSVLDAMNGKEGVIEC
15 tr|A0A8B9BBN8|ANSER BRACHYRHYNCHUS   LTGRIQEAGTEVVKAKAGAGSATLSMAYAGARFVFSLVDAMNGKEGVIEC
16 tr|A0A7L2UU21|BALAENICEPS REX        LTGRIQEAGTEVVKAKAGAGSATLSMAYAGARFVFSLVDAMNGKEGVIEC
17 tr|E1BVT3|GALLUS GALLUS              LTGRIQEAGTEVVKAKAGAGSATLSMAYAGARFVFSLVDAMNGKEGVIEC
18 tr|A0A850X7V7|PIAYA CAYANA           LTGRIQEAGTEVVKAKAGAGSATLSMAYAGARFVFSLVDAMNGKEGVIEC
19 tr|A0A218V2K7|LONCHURA STRIATA       LTARIQEAGTEVVQAKAGAGSATLSMAYAGARFVFSLLDAMSGKQGVVEC
20 tr|A0A8C9MRZ4|SERINUS CANARIA        LTARIQEAGTEVVQAKAGAGSATLSMAYAGARFAFSLLEAMSGKQGVVEC
                                        :  ***:.*****:***************.*:*. : : .: *. .*:**

                                        301      .         .         .         .         350
 1 tr|A0A1P8BBQ0|ARABIDOPSIS THALIANA   SFVASQVTELAFFATKVRLGRTGAEEVYQLGPLNEYERIGLEKAKDELAG
 2 tr|A0A8M1PNU6|DANIO RERIO            SFVRSEETECKYFSTPLLLGKNGIEKNLGLGKLSAFEEKLVADAMTELKG
 3 tr|A0A6I9JAA7|CHRYSOCHLORIS ASIATICA SFVKSQETDCTYFSTPLVLGKKGLEKNMGIGKVSSFEEKMIAEAIPELKA
 4 tr|A0A8C7EKE8|NEOVISON VISON         SFVKSQEADCGYFSTPLLLGKKGIEKNLGIGKISPFEEKMIAEAIPELKA
 5 sp|Q5NVR2|PONGO ABELII               SFVKSQETECTYFSTPLLLGKKGIEKNLGIGKVSSFEEKMISDAIPELKA
 6 tr|H2QUS9|PAN TROGLODYTES            SFVKSQETECTYFSTPLLLGKKGIEKNLGIGKVSSFEEKMISDAIPELKA
 7 sp|P40926|HOMO SAPIENS               SFVKSQETECTYFSTPLLLGKKGIEKNLGIGKVSSFEEKMISDAIPELKA
 8 tr|F6TYW4|EQUUS CABALLUS             SFVKSQETDCPYFSTPLLLGKKGIEKNLGLGKLSSFEEKMIAEALPELKA
 9 sp|P00346|SUS SCROFA                 SFVKSQETDCPYFSTPLLLGKKGIEKNLGIGKISPFEEKMIAEAIPELKA
10 sp|Q32LG3|BOS TAURUS                 SFVKSQETDCPYFSTPLLLGKKGIEKNLGIGKVSPFEEKMIAEAIPELKA
11 sp|P08249|MUS MUSCULUS               SFVQSKETECTYFSTPLLLGKKGLEKNLGIGKITPFEEKMIAEAIPELKA
12 sp|P04636|RATTUS NORVEGICUS          SFVQSKETECTYFSTPLLLGKKGLEKNLGIGKITPFEEKMIAEAIPELKA
13 tr|A0A8C4WQE8|GOPHERUS EVGOODEI      SFVRSEETECPYFSTPLLLGKNGIEKNLGIGKISPYEEKMIAEAMAELKA
14 tr|A0A8C8VKR3|PELUSIOS CASTANEUS     SFVRSEETECPYFSTPLLLGKNGIEKNLGIGKISPYEEKMITEAMSELKA
15 tr|A0A8B9BBN8|ANSER BRACHYRHYNCHUS   SFVRSEETESPYFSTPLLLGKNGIEKNLGIGKITPFEEKMVAEAMSELKA
```

```
16 tr|A0A7L2UU21|BALAENICEPS REX        AFVRSEETESPYFSTPLLLGKNGIEKNLGMGKISPFEEKMVAEAMSELKA
17 tr|E1BVT3|GALLUS GALLUS              SFVRSEETESPYFSTPLLLGKNGIEKNLGIGKITPFEEKMVAEAMAELKA
18 tr|A0A850X7V7|PIAYA CAYANA           AFVRSEETESPYFSTPLLLGKNGIEKNLGIGKISPFEEKMVAEAMAELKA
19 tr|A0A218V2K7|LONCHURA STRIATA       AFVRSDVTEVPYFSTPLQLGKKGIEKNLGLGKLSPFEEKMVAAAMSELKG
20 tr|A0A8C9MRZ4|SERINUS CANARIA        AFVRSDVTEVPYFSTPLQLGKKGIEKNLGLGKLSPFEEKMVAAAMSELKG
                                        :** *. ::  :*:* : **:.* *:   :* :. :*.  :  *  ** .

                                        351      .  363
 1 tr|A0A1P8BBQ0|ARABIDOPSIS THALIANA   SIQKGVEFIRK--
 2 tr|A0A8M1PNU6|DANIO RERIO            SIKKGEDFVANMK
 3 tr|A0A6I9JAA7|CHRYSOCHLORIS ASIATICA SIKKGEDFVKNMK
 4 tr|A0A8C7EKE8|NEOVISON VISON         SIKKGEDFVKNMK
 5 sp|Q5NVR2|PONGO ABELII               SIKKGEDFVKTLK
 6 tr|H2QUS9|PAN TROGLODYTES            SIKKGEDFVKTLK
 7 sp|P40926|HOMO SAPIENS               SIKKGEDFVKTLK
 8 tr|F6TYW4|EQUUS CABALLUS             SIKKGEEFVKSMK
 9 sp|P00346|SUS SCROFA                 SIKKGEEFVKNMK
10 sp|Q32LG3|BOS TAURUS                 SIKKGEEFVKNMK
11 sp|P08249|MUS MUSCULUS               SIKKGEDFVKNMK
12 sp|P04636|RATTUS NORVEGICUS          SIKKGEDFVKNMK
13 tr|A0A8C4WQE8|GOPHERUS EVGOODEI      SIKKGEEFAKSMK
14 tr|A0A8C8VKR3|PELUSIOS CASTANEUS     SIKKGEEFVKSMK
15 tr|A0A8B9BBN8|ANSER BRACHYRHYNCHUS   SIKKGEDFAKNFK
16 tr|A0A7L2UU21|BALAENICEPS REX        SIKKGEEFAKNFK
17 tr|E1BVT3|GALLUS GALLUS              SIKKGEDFAKNFK
18 tr|A0A850X7V7|PIAYA CAYANA           SIKKGEEFAKNFK
19 tr|A0A218V2K7|LONCHURA STRIATA       SIKKGEEFAKNFK
20 tr|A0A8C9MRZ4|SERINUS CANARIA        SIKKGEEFAKNFK
                                        **:** :*
```

Supporting data 8

Folded MDH 15 sec

```
1        10        20        30        40        50
MLSALARPAGAALRRSFSTSAQNNAKVAVLGASGGIGQPLSLLLKNSPLV
                        AKVAVLGASGGIGQPLSLLLKNSPLV
                                             NSPLV

51       60        70        80        90        100
SRLTLYDIAHTPGVAADLSHIETRATVKGYLGPEQLPDCLKGCDVVVIPA
SR
SR
  LTLYDIAHTPGVAADLSHIETR
  LTLYDIAHTPGVAADLSHIETRATVK
   TLYDIAHTPGVAADLSHIETR
    LYDIAHTPGVAADLSHIETR

101      110       120       130       140       150
GVPRKPGMTRDDLFNTNATIVATLTAACAQHCPDAMICIISNPVNSTIPI

151      160       170       180       190       200
TAEVFKKHGVYNPNKIFGVTTLDIVRANAFVAELKGLDPARVSVPVIGGH
                          ANAFVAELK
                          ANAFVAELKGLDPAR
                                         VSVPVIGGH

201      210       220       230       240       250
AGKTIIPLISQCTPKVDFPQDQLSTLTGRIQEAGTEVVKAKAGAGSATLS
AGK
               VDFPQDQLSTLTGR
                             IQEAGTEVVK
                             IQEAGTEVVKAK
                              QEAGTEVVKAK
                               EAGTEVVKAK
                                       AKAGAGSATLS
                                         AGAGSATLS
                                           AGSATLS
                                           GSATLS
                                             SATLS

251      260       270       280       290       300
MAYAGARFVFSLVDAMNGKEGVVECSFVKSQETDCPYFSTPLLLGKKGIE
MAYAGAR
MAYAGAR
MAYAGAR
MAYAGAR
MAYAGAR
       FVFSLVDAMNGK
                                              KGIE
                                              KGIE
                                               GIE
                                               GIE
                                               GIE

301      310       320       330     338
KNLGIGKISPFEEKMIAEAIPELKASIKKGEEFVKNMK
KNLGIGK
KNLGIGKISPFEEK
KNLGIGK
KNLGIGKISPFEEK
NLGIGKISPFEEK
NLGIGKISPFEEKMIAEAIPELKASIK
```

```
       ISPFEEK
       ISPFEEKMIAEAIPELK
       ISPFEEKMIAEAIPELKASIK
301      310       320       330     338
KNLGIGKISPFEEKMIAEAIPELKASIKKGEEFVKNMK
              MIAEAIPELK
              MIAEAIPELKASIK
              MIAEAIPELKASIKKGEEFVKNMK
                        ASIKKGEEFVK
                        ASIKKGEEFVKNMK
                            KGEEFVK
                            KGEEFVKNMK
                             GEEFVKNMK
```

Folded MDH 30 sec

```
1        10        20        30        40        50
MLSALARPAGAALRRSFSTSAQNNAKVAVLGASGGIGQPLSLLLKNSPLV
                        AKVAVLGASGGIGQPLSLLLKNSPLV
                          VAVLGASGGIGQPLSLLLK
                                             NSPLV

51       60        70        80        90        100
SRLTLYDIAHTPGVAADLSHIETRATVKGYLGPEQLPDCLKGCDVVVIPA
SR
SR
  LTLYDIAHTPGVA
  LTLYDIAHTPGVAADLSHIETR
  LTLYDIAHTPGVAADLSHIETRATVK
   TLYDIAHTPGVAADLSHIETR
    LYDIAHTPGVAADLSHIETR
     YDIAHTPGVAADLSHIETR
          TPGVAADLSHIETR
               ADLSHIETR

101      110       120       130       140       150
GVPRKPGMTRDDLFNTNATIVATLTAACAQHCPDAMICIISNPVNSTIPI

151      160       170       180       190       200
TAEVFKKHGVYNPNKIFGVTTLDIVRANAFVAELKGLDPARVSVPVIGGH
               IFGVTTLDIVR
                          ANAFVAELK
                          ANAFVAELKGLDPAR
                          ANAFVAELKGLDPARVSVPVIGGH
                                  KGLDPARVSVPVIGGH
                                   GLDPARVSVPVIGGH
                                         VSVPVIGGH
                                         VSVPVIGGH

201      210       220       230       240       250
AGKTIIPLISQCTPKVDFPQDQLSTLTGRIQEAGTEVVKAKAGAGSATLS
AGK
AG
AGK
AGK
AGKTIIPLISQCTPK
   TIIPLISQCTPK
               VDFPQDQLSTLTGR
               VDFPQDQLSTLTGRIQEAGTEVVK
               VDFPQDQLSTLTGRIQEAGTEVVKAK
                 FPQDQLSTLTGR
                             IQEAGTEVVK
                             IQEAGTEVVKAK
                              QEAGTEVVKAK
                               EAGTEVVKAK
                                       AKAGAGSATLS
                                         AGAGSATLS
                                           AGSATLS
                                            GSATLS
                                             SATLS

251      260       270       280       290       300
MAYAGARFVFSLVDAMNGKEGVVECSFVKSQETDCPYFSTPLLLGKKGIE
MAYAGAR
MAYAGAR
MAYAGAR
MAYAGAR
MAYAGAR
```

```
251      260       270       280       290       300
MAYAGARFVFSLVDAMNGKEGVVECSFVKSQETDCPYFSTPLLLGKKGIE
       FVFSLVDAMNGK
                             SQETDCPYFSTPLLLGKK
                                              KGIE
                                              KGIE
                                              KGIE
                                              KGIE
                                               GIE
                                               GIE
                                               GIE
                                               GIE
                                                 E

301      310       320       330     338
KNLGIGKISPFEEKMIAEAIPELKASIKKGEEFVKNMK
KNLGIGK
KNLGIGKISPFEEK
KNLGIGKISPFEEKMIAEAIPELK
KNLGIGKISPFEEKMIAEAIPELKASIK
KNLGIGK
KNLGIGKISPFEEK
KNLGIGKISPFEEKMIAEAIPELK
KNLGIGKISPFEEKMIAEAIPELKASIK
 NLGIGKISPFEEK
 NLGIGKISPFEEKMIAEAIPELK
 NLGIGKISPFEEKMIAEAIPELKASIK
   GIGKISPFEEK
       ISPFEEK
       ISPFEEKMIAEAIPELK
       ISPFEEKMIAEAIPELKASIK
       ISPFEEKMIAEAIPELKASIKKGEEFVK
              MIAEAIPELK
              MIAEAIPELKASIK
              MIAEAIPELKASIKKGEEFVK
              MIAEAIPELKASIKKGEEFVKNMK
                        ASIKKGEEFVK
                        ASIKKGEEFVKNMK
                            KGEEFVK
                            KGEEFVKNMK
                             GEEFVKNMK
```

Folded MDH 60 sec

```
1        10        20        30        40        50
MLSALARPAGAALRRSFSTSAQNNAKVAVLGASGGIGQPLSLLLKNSPLV
                        AKVAVLGASGGIGQPL
                        AKVAVLGASGGIGQPLSLLLKNSPLV
                          VAVLGASGGIGQPLSLLLK
                          VAVLGASGGIGQPLSLLLKNSPLV
                                             NSPLV
                                             NSPLV
                                               PLV

51       60        70        80        90        100
SRLTLYDIAHTPGVAADLSHIETRATVKGYLGPEQLPDCLKGCDVVVIPA
SR
SR
SR
SRLTLYDIAHTPGVAADLSHIETR
SRLTLYDIAHTPGVAADLSHIETR
  LTLYDIAHTPGVA
  LTLYDIAHTPGVAADLSHIETR
  LTLYDIAHTPGVAADLSHIETRATVK
   TLYDIAHTPGVAADLSHIETR
    LYDIAHTPGVAADLSHIETR
     YDIAHTPGVAADLSHIETR
      DIAHTPGVAADLSHIETR
          TPGVAADLSHIETR
               ADLSHIETR
                        ATVKGYLGPEQLPDCLK

101      110       120       130       140       150
GVPRKPGMTRDDLFNTNATIVATLTAACAQHCPDAMICIISNPVNSTIPI

151      160       170       180       190       200
TAEVFKKHGVYNPNKIFGVTTLDIVRANAFVAELKGLDPARVSVPVIGGH
      KHGVYNPNKIFGVTTLDIVR
       HGVYNPNKIFGVTTLDIVR
               IFGVTTLDIVR
                          ANAFVAELK
                          ANAFVAELKGLDPAR
                          ANAFVAELKGLDPARVSVPVIGGH
                                  KGLDPARVSVPVIGGH
                                   GLDPARVSVPVIGGH
                                         VSVPVIGGH
                                         VSVPVIGGH
                                          SVPVIGGH


201      210       220       230       240       250
AGKTIIPLISQCTPKVDFPQDQLSTLTGRIQEAGTEVVKAKAGAGSATLS
AGK
AG
AGK
AGK
AGKTIIPLISQCTPK
AGK
   TIIPLISQCTPK
   TIIPLISQCTPKVDFPQDQLSTLTGR
               VDFPQDQLSTLTGR
               VDFPQDQLSTLTGRIQEAGTEVVK
               VDFPQDQLSTLTGRIQEAGTEVVKAK
                 FPQDQLSTLTGR
```

```
201      210       220       230       240       250
AGKTIIPLISQCTPKVDFPQDQLSTLTGRIQEAGTEVVKAKAGAGSATLS
                             IQEAGTEVVK
                             IQEAGTEVVKAK
                              QEAGTEVVK
                              QEAGTEVVKAK
                               EAGTEVVKAK
                                AGTEVVKAK
                                       AKAGAGSATLS
                                         AGAGSATLS
                                          GAGSATLS
                                           AGSATLS
                                            GSATLS
                                             SATLS

251      260       270       280       290       300
MAYAGARFVFSLVDAMNGKEGVVECSFVKSQETDCPYFSTPLLLGKKGIE
MAYAGAR
MAYAGAR
MAYAGAR
MAYAGAR
MAYAGAR
MAYAGAR
       FVFSLVDAMNGK
       FVFSLVDAMNGKEGVVECSFVK
                             SQETDCPYFSTPLLLGK
                             SQETDCPYFSTPLLLGKK
                                              KGIE
                                              KGIE
                                              KGIE
                                              KGIE
                                               GIE
                                               GIE
                                               GIE
                                               GIE
                                                 E

301      310       320       330     338
KNLGIGKISPFEEKMIAEAIPELKASIKKGEEFVKNMK
K
KNLGIGK
KNLGIGKISPFEEK
KNLGIGKISPFEEKMIAEAIPELK
KNLGIGKISPFEEKMIAEAIPELKASIK
KNLGIGK
KNLGIGKISPFEEK
KNLGIGKISPFEEKMIAEAIPELK
KNLGIGKISPFEEKMIAEAIPELKASIK
KNLGIGKISPFEEK
 NLGIGKISPFEEK
 NLGIGKISPFEEKMIAEAIPELK
 NLGIGKISPFEEKMIAEAIPELKASIK
 NLGIGKISPFEEKMIAEAIPELKASIKKGEEFVK
   GIGKISPFEEK
       ISPFEEK
       ISPFEEKMIAEAIPELK
       ISPFEEKMIAEAIPELKASIK
         PFEEKMIAEAIPELKASIK
              MIAEAIPELK
              MIAEAIPELKASIK
              MIAEAIPELKASIKKGEEFVK
              MIAEAIPELKASIKKGEEFVKNMK
                        ASIKKGEEFVK
                        ASIKKGEEFVKNMK
```

```
301      310       320       330     338
KNLGIGKISPFEEKMIAEAIPELKASIKKGEEFVKNMK
                            KGEEFVK
                            KGEEFVKNMK
                             GEEFVKNMK
```

Folded MDH 120 sec

```
1         10        20        30        40        50
MLSALARPAGAALRRSFSTSAQNNAKVAVLGASGGIGQPLSLLLKNSPLV
                        AKVAVLGASGGIGQPL
                        AKVAVLGASGGIGQPLSLLLKNSPLV
                          VAVLGASGGIGQPLSLLLK
                          VAVLGASGGIGQPLSLLLKNSPLV
                                    GQPLSLLLKNSPLV
                                      PLSLLLKNSPLV
                                             NSPLV
                                             NSPLV
                                               PLV

51        60        70        80        90        100
SRLTLYDIAHTPGVAADLSHIETRATVKGYLGPEQLPDCLKGCDVVVIPA
SR
SR
SR
SR
SR
SRLTLYDIAHTPGVAADLSHIETR
SRLTLYDIAHTPGVAADLSHIETRATVK
  LTLYDIAHTPG
  LTLYDIAHTPGVA
  LTLYDIAHTPGVAAD
  LTLYDIAHTPGVAADLSHIET
  LTLYDIAHTPGVAADLSHIETR
  LTLYDIAHTPGVAADLSHIETRATVK
   TLYDIAHTPGVAADLSHIETR
    LYDIAHTPGVAADLSHIETR
     YDIAHTPGVAADLSHIETR
      DIAHTPGVAADLSHIETR
          TPGVAADLSHIETR
           PGVAADLSHIETR
               ADLSHIETR
                        ATVKGYLGPEQLPDCLK
                            GYLGPEQLPDCLK
                            GYLGPEQLPDCLKGCDVVVIPA

101       110       120       130       140       150
GVPRKPGMTRDDLFNTNATIVATLTAACAQHCPDAMICIISNPVNSTIPI
GVPR

151       160       170       180       190       200
TAEVFKKHGVYNPNKIFGVTTLDIVRANAFVAELKGLDPARVSVPVIGGH
     KHHGVYNPNKIFGVTTLDIVR
       HGVYNPNKIFGVTTLDIVR
               IFGVTTLDIVR
                          ANAFVAELK
                          ANAFVAELKGLD
                          ANAFVAELKGLDPAR
                          ANAFVAELKGLDPARVSVPVIGGH
                            AFVAELKGLDPAR
                              VAELKGLDPAR
                                  KGLDPARVSVPVIGGH
                                   GLDPARVSVPVIGGH
                                         VSVPVIGGH
                                         VSVPVIGGH
                                          SVPVIGGH
```

```
201      210       220       230       240       250
AGKTIIPLISQCTPKVDFPQDQLSTLTGRIQEAGTEVVKAKAGAGSATLS
AGK
AG
AGK
AGK
AGKTIIPLISQCTPK
AGK
   TIIPLISQCTPK
   TIIPLISQCTPKVDFPQDQLSTLTGR
               VDFPQDQLSTLTGR
               VDFPQDQLSTLTGRIQEAGTEVVK
               VDFPQDQLSTLTGRIQEAGTEVVKAK
                 FPQDQLSTLTGR
                  PQDQLSTLTGR
                             IQEAGTEVVK
                             IQEAGTEVVKAK
                              QEAGTEVVK
                              QEAGTEVVKAK
                               EAGTEVVKAK
                                AGTEVVKAK
                                       AKAGAGSATLS
                                         AGAGSATLS
                                         AGAGSATLS
                                           AGSATLS
                                            GSATLS
                                             SATLS

251      260       270       280       290       300
MAYAGARFVFSLVDAMNGKEGVVECSFVKSQETDCPYFSTPLLLGKKGIE
MAYAGAR
MAYAGAR
MAYAGARF
MAYAGAR
MAYAGAR
MAYAGAR
       FVFSLVDAMNGK
       FVFSLVDAMNGKEGVVECSFVK
                   EGVVECSFVK
                             SQETDCPYFSTPLLLGK
                             SQETDCPYFSTPLLLGKK
                             SQETDCPYFSTPLLLGKKGIE
                                     FSTPLLLGKK
                                              KGIE
                                              KGIE
                                              KGIE
                                              KGIE
                                               GIE
                                               GIE
                                               GIE
                                               GIE
                                                 E

301      310       320       330     338
KNLGIGKISPFEEKMIAEAIPELKASIKKGEEFVKNMK
K
KNLGIGKISPFEEK
KNLGIGKISPFEEKMIAEAIPELK
KNLGIGKISPFEEKMIAEAIPELKASIK
KNLGIGK
KNLGIGKISPFEEK
KNLGIGKISPFEEKMIAEAIPELK
KNLGIGKISPFEEKMIAEAIPELKASIK
KNLGIGKISPFEEK
```

```
301      310       320       330     338
KNLGIGKISPFEEKMIAEAIPELKASIKKGEEFVKNMK
 NLGIGKISPFEEK
 NLGIGKISPFEEKMIAEAIPELK
 NLGIGKISPFEEKMIAEAIPELKASIK
  LGIGKISPFEEK
   GIGKISPFEEK
   GIGKISPFEEKMIAEAIPELK
    IGKISPFEEK
     GKISPFEEK
       ISPFEEK
       ISPFEEKMIAEAIPELK
       ISPFEEKMIAEAIPELKASIK
       PFEEKMIAEAIPELK
       PFEEKMIAEAIPELKASIK
              MIAEAIPELK
              MIAEAIPELKASIK
              MIAEAIPELKASIKKGEEFVK
              MIAEAIPELKASIKKGEEFVKNMK
                AEAIPELKASIK
                    PELKASIK
                        ASIKKGEEFVK
                        ASIKKGEEFVKNMK
                            KGEEFVK
                            KGEEFVKNMK
                             GEEFVKNMK
```

Supporting data 9

Folded MDH digested with 2 nM trypsin 0 sec

```
1        10        20        30        40        50
MLSALARPAGAALRRSFSTSAQNNAKVAVLGASGGIGQPLSLLLKNSPLV

51       60        70        80        90        100
SRLTLYDIAHTPGVAADLSHIETRATVKGYLGPEQLPDCLKGCDVVVIPA

101      110       120       130       140       150
GVPRKPGMTRDDLFNTNATIVATLTAACAQHCPDAMICIISNPVNSTIPI

151      160       170       180       190       200
TAEVFKKHGVYNPNKIFGVTTLDIVRANAFVAELKGLDPARVSVPVIGGH

201      210       220       230       240       250
AGKTIIPLISQCTPKVDFPQDQLSTLTGRIQEAGTEVVKAKAGAGSATLS

251      260       270       280       290       300
MAYAGARFVFSLVDAMNGKEGVVECSFVKSQETDCPYFSTPLLLGKKGIE
                                              KGIE

301      310       320       330     338
KNLGIGKISPFEEKMIAEAIPELKASIKKGEEFVKNMK
KNLGIGKISPFEEK
```

Folded MDH 15 sec

```
1        10        20        30        40        50
MLSALARPAGAALRRSFSTSAQNNAKVAVLGASGGIGQPLSLLLKNSPLV
                        AKVAVLGASGGIGQPLSLLLKNSPLV
                                             NSPLV


51      60        70        80        90        100
SRLTLYDIAHTPGVAADLSHIETRATVKGYLGPEQLPDCLKGCDVVVIPA
SR
SR
  LTLYDIAHTPGVAADLSHIETR
  LTLYDIAHTPGVAADLSHIETRATVK
    LYDIAHTPGVAADLSHIETR


101      110       120       130       140       150
GVPRKPGMTRDDLFNTNATIVATLTAACAQHCPDAMICIISNPVNSTIPI

151      160       170       180       190       200
TAEVFKKHGVYNPNKIFGVTTLDIVRANAFVAELKGLDPARVSVPVIGGH
                          ANAFVAELK
                          ANAFVAELKGLDPAR
                                         VSVPVIGGH

201      210       220       230       240       250
AGKTIIPLISQCTPKVDFPQDQLSTLTGRIQEAGTEVVKAKAGAGSATLS
AGK
               VDFPQDQLSTLTGR
                             IQEAGTEVVK
                             IQEAGTEVVKAK
                              QEAGTEVVKAK
                               EAGTEVVKAK
                                       AKAGAGSATLS
                                         AGAGSATLS
                                           AGSATLS
                                            GSATLS
                                             SATLS

251      260       270       280       290       300
MAYAGARFVFSLVDAMNGKEGVVECSFVKSQETDCPYFSTPLLLGKKGIE
MAYAGAR
MAYAGAR
MAYAGAR
MAYAGAR
MAYAGAR
                                              KGIE
                                              KGIE
                                               GIE
                                               GIE
                                               GIE

301      310       320       330     338
KNLGIGKISPFEEKMIAEAIPELKASIKKGEEFVKNMK
KNLGIGK
KNLGIGKISPFEE
KNLGIGK
KNLGIGKISPFEEK
KNLGIGKISPFEEKMIAEAIPELK
 NLGIGKISPFEEK
      ISPFEEK
      ISPFEEKMIAEAIPELK
      ISPFEEKMIAEAIPELKASIK
```

```
301      310       320       330     338
KNLGIGKISPFEEKMIAEAIPELKASIKKGEEFVKNMK
     ISPFEEKMIAEAIPELKASIK
             MIAEAIPELK
             MIAEAIPELKASIK
             MIAEAIPELKASIKKGEEFVKNMK
                       ASIKKGEEFVK
                       ASIKKGEEFVKNMK
                           KGEEFVK
                           KGEEFVKNMK
                            GEEFVKNMK
```

Folded MDH 30 sec

```
1        10        20        30        40        50
MLSALARPAGAALRRSFSTSAQNNAKVAVLGASGGIGQPLSLLLKNSPLV
                        AKVAVLGASGGIGQPLSLLLKNSPLV
                                             NSPLV

51       60        70        80        90        100
SRLTLYDIAHTPGVAADLSHIETRATVKGYLGPEQLPDCLKGCDVVVIPA
SR
SR
  LTLYDIAHTPGVA
  LTLYDIAHTPGVAADLSHIETR
  LTLYDIAHTPGVAADLSHIETRATVK
   TLYDIAHTPGVAADLSHIETR
    LYDIAHTPGVAADLSHIETR
     YDIAHTPGVAADLSHIETR

101      110       120       130       140       150
GVPRKPGMTRDDLFNTNATIVATLTAACAQHCPDAMICIISNPVNSTIPI

151      160       170       180       190       200
TAEVFKKHGVYNPNKIFGVTTLDIVRANAFVAELKGLDPARVSVPVIGGH
               IFGVTTLDIVR
                          ANAFVAELK
                          ANAFVAELKGLDPAR
                                         VSVPVIGGH

201      210       220       230       240       250
AGKTIIPLISQCTPKVDFPQDQLSTLTGRIQEAGTEVVKAKAGAGSATLS
AGK
   TIIPLISQCTPK
               VDFPQDQLSTLTGR
               VDFPQDQLSTLTGRIQEAGTEVVK
               VDFPQDQLSTLTGRIQEAGTEVVKAK
                             IQEAGTEVVK
                             IQEAGTEVVKAK
                              QEAGTEVVKAK
                               EAGTEVVKAK
                                       AKAGAGSATLS
                                         AGAGSATLS
                                           AGSATLS
                                            GSATLS
                                             SATLS

251      260       270       280       290       300
MAYAGARFVFSLVDAMNGKEGVVECSFVKSQETDCPYFSTPLLLGKKGIE
MAYAGAR
MAYAGAR
MAYAGAR
MAYAGAR
MAYAGAR
       FVFSLVDAMNGK
                             SQETDCPYFSTPLLLGKK
                                              KGIE
                                              KGIE
                                               GIE
                                               GIE
                                               GIE

301      310       320       330     338
KNLGIGKISPFEEKMIAEAIPELKASIKKGEEFVKNMK
KNLGIGK
KNLGIGKISPFEEK
```

```
301      310       320       330     338
KNLGIGKISPFEEKMIAEAIPELKASIKKGEEFVKNMK
KNLGIGK
KNLGIGKISPFEEK
KNLGIGKISPFEEKMIAEAIPELK
 NLGIGKISPFEEK
 NLGIGKISPFEEKMIAEAIPELK
 NLGIGKISPFEEKMIAEAIPELKASIK
   GIGKISPFEEK
       ISPFEEK
       ISPFEEKMIAEAIPELK
       ISPFEEKMIAEAIPELKASIK
              MIAEAIPELK
              MIAEAIPELKASIK
              MIAEAIPELKASIKKGEEFVK
              MIAEAIPELKASIKKGEEFVKNMK
                        ASIKKGEEFVK
                        ASIKKGEEFVKNMK
                            KGEEFVK
                            KGEEFVKNMK
                             GEEFVKNMK
```

Supporting data 10

Overview

ACE project	Title
ACE_0653-01	Time-resolved analysis of proteolytic products of ANXA1 (concentration constant 0 or 20 μM, time points 0, 15, 30 sec) by recombinant HTRA1 (concentrations: 0, 1, 2 or 4 μM)
ACE_0653-02	Time-resolved analysis of proteolytic products of ANXA1 (concentration constant 0 or 20 μM, time points 0, 15, 30, 60, 90, 120, 300, 600, 1200, 2400 or 3600 sec) by recombinant HTRA1 (concentrations: 0 or 20 μM)
ACE_0653-03	Time-resolved analysis of proteolytic products of ANXA1-denat (concentration constant 0 or 20 μM, time points 0, 15, 30, 60, 90, 120, 300 sec) by recombinant HTRA1 (concentrations: 0 or 20 μM)
ACE_0793-01	Time-resolved analysis of proteolytic products of MDH (concentration constant 0 or 20 µM, time points 0, 15, 30, 45, 60, 90, 120, 300, 600, 1200, 1800 or 7200 sec) by Trypsin (concentrations: 0 or 0.004 µM)
ACE_0793-02	Time-resolved analysis of proteolytic products of MDH-denat (concentration constant 0 or 20 µM, time points 0, 15, 30, 45, 60, 120, 300, 600 sec) by Trypsin (concentrations: 0 or 0.004 µM)
ACE_0793-03	Time-resolved analysis of proteolytic products of MDH (concentration constant 0 or 20 µM, time points 0, 15, 30 sec) by Trypsin (concentrations: 0 or 0.002 µM)

ANXA1 = folded ANXA1; ANXA1-denat = chemically denatured ANXA1
MDH = folded MDH; MDH-denat = chemically denatured MDH

ACE_0653-01/02/03

File legend

ACE ID	Organism	Organ/ cell line	Treatment/ experimental setup
ACE_0653-01_MK01	Human (overexpressed in *E. coli*)	Recomb. protein	HTRA1 0 μM + ANXA1 20 μM, 0 sec, supernatant after acetone precipitation
ACE_0653-01_MK02	Human (overexpressed in *E. coli*)	Recomb. protein	HTRA1 0 μM + ANXA1 20 μM, 0 sec, supernatant after acetone precipitation
ACE_0653-01_MK03	Human (overexpressed in *E. coli*)	Recomb. protein	HTRA1 0 μM + ANXA1 20 μM, 0 sec, supernatant after acetone precipitation
ACE_0653-01_MK04	Human (overexpressed in *E. coli*)	Recomb. protein	HTRA1 0 μM + ANXA1 20 μM, 0 sec, supernatant after acetone precipitation
ACE_0653-01_MK05	Human (overexpressed in *E. coli*)	Recomb. protein	HTRA1 0 μM + ANXA1 20 μM, 30 sec, supernatant after acetone precipitation
ACE_0653-01_MK06	Human (overexpressed in *E. coli*)	Recomb. protein	HTRA1 0 μM + ANXA1 20 μM, 30 sec, supernatant after acetone precipitation
ACE_0653-01_MK07	Human (overexpressed in *E. coli*)	Recomb. protein	HTRA1 0 μM + ANXA1 20 μM, 30 sec, supernatant after acetone precipitation
ACE_0653-01_MK08	Human (overexpressed in *E. coli*)	Recomb. protein	HTRA1 0 μM + ANXA1 20 μM, 30 sec, supernatant after acetone precipitation
ACE_0653-01_MK09	Human (overexpressed in *E. coli*)	Recomb. protein	HTRA1 4 μM + ANXA1 0 μM, 0 sec, supernatant after acetone precipitation
ACE_0653-01_MK10	Human (overexpressed in *E. coli*)	Recomb. protein	HTRA1 4 μM + ANXA1 0 μM, 0 sec, supernatant after acetone precipitation
ACE_0653-01_MK11	Human (overexpressed in *E. coli*)	Recomb. protein	HTRA1 4 μM + ANXA1 0 μM, 0 sec, supernatant after acetone precipitation
ACE_0653-01_MK12	Human (overexpressed in *E. coli*)	Recomb. protein	HTRA1 4 μM + ANXA1 0 μM, 0 sec, supernatant after acetone precipitation
ACE_0653-01_MK13	Human (overexpressed in *E. coli*)	Recomb. protein	HTRA1 4 μM + ANXA1 0 μM, 30 sec, supernatant after acetone precipitation
ACE_0653-01_MK14	Human (overexpressed in *E. coli*)	Recomb. protein	HTRA1 4 μM + ANXA1 0 μM, 30 sec, supernatant after acetone precipitation
ACE_0653-01_MK15	Human (overexpressed in *E. coli*)	Recomb. protein	HTRA1 4 μM + ANXA1 0 μM, 30 sec, supernatant after acetone precipitation
ACE_0653-01_MK16	Human (overexpressed in *E. coli*)	Recomb. protein	HTRA1 4 μM + ANXA1 0 μM, 30 sec, supernatant after acetone precipitation
ACE_0653-01_MK17	Human (overexpressed in *E. coli*)	Recomb. protein	HTRA1 1 μM + ANXA1 20 μM, 0 sec, supernatant after acetone precipitation
ACE_0653-01_MK18	Human (overexpressed in *E. coli*)	Recomb. protein	HTRA1 1 μM + ANXA1 20 μM, 0 sec, supernatant after acetone precipitation
ACE_0653-01_MK19	Human (overexpressed in *E. coli*)	Recomb. protein	HTRA1 1 μM + ANXA1 20 μM, 0 sec, supernatant after acetone precipitation
ACE_0653-01_MK20	Human (overexpressed in *E. coli*)	Recomb. protein	HTRA1 1 μM + ANXA1 20 μM, 0 sec, supernatant after acetone precipitation
ACE_0653-01_MK21	Human (overexpressed in *E. coli*)	Recomb. protein	HTRA1 1 μM + ANXA1 20 μM, 15 sec, supernatant after acetone precipitation
ACE_0653-01_MK22	Human (overexpressed in *E. coli*)	Recomb. protein	HTRA1 1 μM + ANXA1 20 μM, 15 sec, supernatant after acetone precipitation
ACE_0653-01_MK23	Human (overexpressed in *E. coli*)	Recomb. protein	HTRA1 1 μM + ANXA1 20 μM, 15 sec, supernatant after acetone precipitation
ACE_0653-01_MK24	Human (overexpressed in *E. coli*)	Recomb. protein	HTRA1 1 μM + ANXA1 20 μM, 15 sec, supernatant after acetone precipitation
ACE_0653-01_MK25	Human (overexpressed in *E. coli*)	Recomb. protein	HTRA1 1 μM + ANXA1 20 μM, 30 sec, supernatant after acetone precipitation
ACE_0653-01_MK26	Human (overexpressed in *E. coli*)	Recomb. protein	HTRA1 1 μM + ANXA1 20 μM, 30 sec, supernatant after acetone precipitation
ACE_0653-01_MK27	Human (overexpressed in *E. coli*)	Recomb. protein	HTRA1 1 μM + ANXA1 20 μM, 30 sec, supernatant after acetone precipitation
ACE_0653-01_MK28	Human (overexpressed in *E. coli*)	Recomb. protein	HTRA1 1 μM + ANXA1 20 μM, 30 sec, supernatant after acetone precipitation
ACE_0653-01_MK29	Human (overexpressed in *E. coli*)	Recomb. protein	HTRA1 2 μM + ANXA1 20 μM, 0 sec, supernatant after acetone precipitation
ACE_0653-01_MK30	Human (overexpressed in *E. coli*)	Recomb. protein	HTRA1 2 μM + ANXA1 20 μM, 0 sec, supernatant after acetone precipitation
ACE_0653-01_MK31	Human (overexpressed in *E. coli*)	Recomb. protein	HTRA1 2 μM + ANXA1 20 μM, 0 sec, supernatant after acetone precipitation
ACE_0653-01_MK32	Human (overexpressed in *E. coli*)	Recomb. protein	HTRA1 2 μM + ANXA1 20 μM, 0 sec, supernatant after acetone precipitation
ACE_0653-01_MK33	Human (overexpressed in *E. coli*)	Recomb. protein	HTRA1 2 μM + ANXA1 20 μM, 15 sec, supernatant after acetone precipitation
ACE_0653-01_MK34	Human (overexpressed in *E. coli*)	Recomb. protein	HTRA1 2 μM + ANXA1 20 μM, 15 sec, supernatant after acetone precipitation
ACE_0653-01_MK35	Human (overexpressed in *E. coli*)	Recomb. protein	HTRA1 2 μM + ANXA1 20 μM, 15 sec, supernatant after acetone precipitation
ACE_0653-01_MK36	Human (overexpressed in *E. coli*)	Recomb. protein	HTRA1 2 μM + ANXA1 20 μM, 15 sec, supernatant after acetone precipitation
ACE_0653-01_MK37	Human (overexpressed in *E. coli*)	Recomb. protein	HTRA1 2 μM + ANXA1 20 μM, 30 sec, supernatant after acetone precipitation
ACE_0653-01_MK38	Human (overexpressed in *E. coli*)	Recomb. protein	HTRA1 2 μM + ANXA1 20 μM, 30 sec, supernatant after acetone precipitation
ACE_0653-01_MK39	Human (overexpressed in *E. coli*)	Recomb. protein	HTRA1 2 μM + ANXA1 20 μM, 30 sec, supernatant after acetone precipitation

ACE_0653-01_MK40	Human (overexpressed in *E. coli*)	Recomb. protein	HTRA1 2 μM + ANXA1 20 μM, 30 sec, supernatant after acetone precipitation
ACE_0653-01_MK41	Human (overexpressed in *E. coli*)	Recomb. protein	HTRA1 4 μM + ANXA1 20 μM, 0 sec, supernatant after acetone precipitation
ACE_0653-01_MK42	Human (overexpressed in *E. coli*)	Recomb. protein	HTRA1 4 μM + ANXA1 20 μM, 0 sec, supernatant after acetone precipitation
ACE_0653-01_MK43	Human (overexpressed in *E. coli*)	Recomb. protein	HTRA1 4 μM + ANXA1 20 μM, 0 sec, supernatant after acetone precipitation
ACE_0653-01_MK44	Human (overexpressed in *E. coli*)	Recomb. protein	HTRA1 4 μM + ANXA1 20 μM, 0 sec, supernatant after acetone precipitation
ACE_0653-01_MK45	Human (overexpressed in *E. coli*)	Recomb. protein	HTRA1 4 μM + ANXA1 20 μM, 15 sec, supernatant after acetone precipitation
ACE_0653-01_MK46	Human (overexpressed in *E. coli*)	Recomb. protein	HTRA1 4 μM + ANXA1 20 μM, 15 sec, supernatant after acetone precipitation
ACE_0653-01_MK47	Human (overexpressed in *E. coli*)	Recomb. protein	HTRA1 4 μM + ANXA1 20 μM, 15 sec, supernatant after acetone precipitation
ACE_0653-01_MK48	Human (overexpressed in *E. coli*)	Recomb. protein	HTRA1 4 μM + ANXA1 20 μM, 15 sec, supernatant after acetone precipitation
ACE_0653-01_MK49	Human (overexpressed in *E. coli*)	Recomb. protein	HTRA1 4 μM + ANXA1 20 μM, 30 sec, supernatant after acetone precipitation
ACE_0653-01_MK50	Human (overexpressed in *E. coli*)	Recomb. protein	HTRA1 4 μM + ANXA1 20 μM, 30 sec, supernatant after acetone precipitation
ACE_0653-01_MK51	Human (overexpressed in *E. coli*)	Recomb. protein	HTRA1 4 μM + ANXA1 20 μM, 30 sec, supernatant after acetone precipitation
ACE_0653-01_MK52	Human (overexpressed in *E. coli*)	Recomb. protein	HTRA1 4 μM + ANXA1 20 μM, 30 sec, supernatant after acetone precipitation
ACE_0653-02_MK053	Human (overexpressed in *E. coli*)	Recomb. protein	HTRA1 0 μM + ANXA1 20 μM, 0 sec, supernatant after acetone precipitation
ACE_0653-02_MK054	Human (overexpressed in *E. coli*)	Recomb. protein	HTRA1 0 μM + ANXA1 20 μM, 0 sec, supernatant after acetone precipitation
ACE_0653-02_MK055	Human (overexpressed in *E. coli*)	Recomb. protein	HTRA1 0 μM + ANXA1 20 μM, 0 sec, supernatant after acetone precipitation
ACE_0653-02_MK056	Human (overexpressed in *E. coli*)	Recomb. protein	HTRA1 0 μM + ANXA1 20 μM, 0 sec, supernatant after acetone precipitation
ACE_0653-02_MK057	Human (overexpressed in *E. coli*)	Recomb. protein	HTRA1 0 μM + ANXA1 20 μM, 3600 sec, supernatant after acetone precipitation
ACE_0653-02_MK058	Human (overexpressed in *E. coli*)	Recomb. protein	HTRA1 0 μM + ANXA1 20 μM, 3600 sec, supernatant after acetone precipitation
ACE_0653-02_MK059	Human (overexpressed in *E. coli*)	Recomb. protein	HTRA1 0 μM + ANXA1 20 μM, 3600 sec, supernatant after acetone precipitation
ACE_0653-02_MK060	Human (overexpressed in *E. coli*)	Recomb. protein	HTRA1 0 μM + ANXA1 20 μM, 3600 sec, supernatant after acetone precipitation
ACE_0653-02_MK061	Human (overexpressed in *E. coli*)	Recomb. protein	HTRA1 20 μM + ANXA1 0 μM, 0 sec, supernatant after acetone precipitation
ACE_0653-02_MK062	Human (overexpressed in *E. coli*)	Recomb. protein	HTRA1 20 μM + ANXA1 0 μM, 0 sec, supernatant after acetone precipitation
ACE_0653-02_MK063	Human (overexpressed in *E. coli*)	Recomb. protein	HTRA1 20 μM + ANXA1 0 μM, 0 sec, supernatant after acetone precipitation
ACE_0653-02_MK064	Human (overexpressed in *E. coli*)	Recomb. protein	HTRA1 20 μM + ANXA1 0 μM, 0 sec, supernatant after acetone precipitation
ACE_0653-02_MK065	Human (overexpressed in *E. coli*)	Recomb. protein	HTRA1 20 μM + ANXA1 0 μM, 3600 sec, supernatant after acetone precipitation
ACE_0653-02_MK066	Human (overexpressed in *E. coli*)	Recomb. protein	HTRA1 20 μM + ANXA1 0 μM, 3600 sec, supernatant after acetone precipitation
ACE_0653-02_MK067	Human (overexpressed in *E. coli*)	Recomb. protein	HTRA1 20 μM + ANXA1 0 μM, 3600 sec, supernatant after acetone precipitation
ACE_0653-02_MK068	Human (overexpressed in *E. coli*)	Recomb. protein	HTRA1 20 μM + ANXA1 0 μM, 3600 sec, supernatant after acetone precipitation
ACE_0653-02_MK069	Human (overexpressed in *E. coli*)	Recomb. protein	HTRA1 20 μM + ANXA1 20 μM, 0 sec, supernatant after acetone precipitation
ACE_0653-02_MK070	Human (overexpressed in *E. coli*)	Recomb. protein	HTRA1 20 μM + ANXA1 20 μM, 0 sec, supernatant after acetone precipitation
ACE_0653-02_MK071	Human (overexpressed in *E. coli*)	Recomb. protein	HTRA1 20 μM + ANXA1 20 μM, 0 sec, supernatant after acetone precipitation
ACE_0653-02_MK072	Human (overexpressed in *E. coli*)	Recomb. protein	HTRA1 20 μM + ANXA1 20 μM, 0 sec, supernatant after acetone precipitation
ACE_0653-02_MK073	Human (overexpressed in *E. coli*)	Recomb. protein	HTRA1 20 μM + ANXA1 20 μM, 15 sec, supernatant after acetone precipitation
ACE_0653-02_MK074	Human (overexpressed in *E. coli*)	Recomb. protein	HTRA1 20 μM + ANXA1 20 μM, 15 sec, supernatant after acetone precipitation
ACE_0653-02_MK075	Human (overexpressed in *E. coli*)	Recomb. protein	HTRA1 20 μM + ANXA1 20 μM, 15 sec, supernatant after acetone precipitation
ACE_0653-02_MK076	Human (overexpressed in *E. coli*)	Recomb. protein	HTRA1 20 μM + ANXA1 20 μM, 15 sec, supernatant after acetone precipitation
ACE_0653-02_MK077	Human (overexpressed in *E. coli*)	Recomb. protein	HTRA1 20 μM + ANXA1 20 μM, 30 sec, supernatant after acetone precipitation
ACE_0653-02_MK078	Human (overexpressed in *E. coli*)	Recomb. protein	HTRA1 20 μM + ANXA1 20 μM, 30 sec, supernatant after acetone precipitation
ACE_0653-02_MK079	Human (overexpressed in *E. coli*)	Recomb. protein	HTRA1 20 μM + ANXA1 20 μM, 30 sec, supernatant after acetone precipitation
ACE_0653-02_MK080	Human (overexpressed in *E. coli*)	Recomb. protein	HTRA1 20 μM + ANXA1 20 μM, 30 sec, supernatant after acetone precipitation
ACE_0653-02_MK081	Human (overexpressed in *E. coli*)	Recomb. protein	HTRA1 20 μM + ANXA1 20 μM, 60 sec, supernatant after acetone precipitation
ACE_0653-02_MK082	Human (overexpressed in *E. coli*)	Recomb. protein	HTRA1 20 μM + ANXA1 20 μM, 60 sec, supernatant after acetone precipitation

ACE_0653-02_MK083	Human (overexpressed in *E. coli*)	Recomb. protein	HTRA1 20 μM + ANXA1 20 μM, 60 sec, supernatant after acetone precipitation
ACE_0653-02_MK084	Human (overexpressed in *E. coli*)	Recomb. protein	HTRA1 20 μM + ANXA1 20 μM, 60 sec, supernatant after acetone precipitation
ACE_0653-02_MK085	Human (overexpressed in *E. coli*)	Recomb. protein	HTRA1 20 μM + ANXA1 20 μM, 90 sec, supernatant after acetone precipitation
ACE_0653-02_MK086	Human (overexpressed in *E. coli*)	Recomb. protein	HTRA1 20 μM + ANXA1 20 μM, 90 sec, supernatant after acetone precipitation
ACE_0653-02_MK087	Human (overexpressed in *E. coli*)	Recomb. protein	HTRA1 20 μM + ANXA1 20 μM, 90 sec, supernatant after acetone precipitation
ACE_0653-02_MK088	Human (overexpressed in *E. coli*)	Recomb. protein	HTRA1 20 μM + ANXA1 20 μM, 90 sec, supernatant after acetone precipitation
ACE_0653-02_MK089	Human (overexpressed in *E. coli*)	Recomb. protein	HTRA1 20 μM + ANXA1 20 μM, 120 sec, supernatant after acetone precipitation
ACE_0653-02_MK090	Human (overexpressed in *E. coli*)	Recomb. protein	HTRA1 20 μM + ANXA1 20 μM, 120 sec, supernatant after acetone precipitation
ACE_0653-02_MK091	Human (overexpressed in *E. coli*)	Recomb. protein	HTRA1 20 μM + ANXA1 20 μM, 120 sec, supernatant after acetone precipitation
ACE_0653-02_MK092	Human (overexpressed in *E. coli*)	Recomb. protein	HTRA1 20 μM + ANXA1 20 μM, 120 sec, supernatant after acetone precipitation
ACE_0653-02_MK093	Human (overexpressed in *E. coli*)	Recomb. protein	HTRA1 20 μM + ANXA1 20 μM, 300 sec, supernatant after acetone precipitation
ACE_0653-02_MK094	Human (overexpressed in *E. coli*)	Recomb. protein	HTRA1 20 μM + ANXA1 20 μM, 300 sec, supernatant after acetone precipitation
ACE_0653-02_MK095	Human (overexpressed in *E. coli*)	Recomb. protein	HTRA1 20 μM + ANXA1 20 μM, 300 sec, supernatant after acetone precipitation
ACE_0653-02_MK096	Human (overexpressed in *E. coli*)	Recomb. protein	HTRA1 20 μM + ANXA1 20 μM, 300 sec, supernatant after acetone precipitation
ACE_0653-02_MK097	Human (overexpressed in *E. coli*)	Recomb. protein	HTRA1 20 μM + ANXA1 20 μM, 600 sec, supernatant after acetone precipitation
ACE_0653-02_MK098	Human (overexpressed in *E. coli*)	Recomb. protein	HTRA1 20 μM + ANXA1 20 μM, 600 sec, supernatant after acetone precipitation
ACE_0653-02_MK099	Human (overexpressed in *E. coli*)	Recomb. protein	HTRA1 20 μM + ANXA1 20 μM, 600 sec, supernatant after acetone precipitation
ACE_0653-02_MK100	Human (overexpressed in *E. coli*)	Recomb. protein	HTRA1 20 μM + ANXA1 20 μM, 600 sec, supernatant after acetone precipitation
ACE_0653-02_MK101	Human (overexpressed in *E. coli*)	Recomb. protein	HTRA1 20 μM + ANXA1 20 μM, 1200 sec, supernatant after acetone precipitation
ACE_0653-02_MK102	Human (overexpressed in *E. coli*)	Recomb. protein	HTRA1 20 μM + ANXA1 20 μM, 1200 sec, supernatant after acetone precipitation
ACE_0653-02_MK103	Human (overexpressed in *E. coli*)	Recomb. protein	HTRA1 20 μM + ANXA1 20 μM, 1200 sec, supernatant after acetone precipitation
ACE_0653-02_MK104	Human (overexpressed in *E. coli*)	Recomb. protein	HTRA1 20 μM + ANXA1 20 μM, 1200 sec, supernatant after acetone precipitation
ACE_0653-02_MK105	Human (overexpressed in *E. coli*)	Recomb. protein	HTRA1 20 μM + ANXA1 20 μM, 2400 sec, supernatant after acetone precipitation
ACE_0653-02_MK106	Human (overexpressed in *E. coli*)	Recomb. protein	HTRA1 20 μM + ANXA1 20 μM, 2400 sec, supernatant after acetone precipitation
ACE_0653-02_MK107	Human (overexpressed in *E. coli*)	Recomb. protein	HTRA1 20 μM + ANXA1 20 μM, 2400 sec, supernatant after acetone precipitation
ACE_0653-02_MK108	Human (overexpressed in *E. coli*)	Recomb. protein	HTRA1 20 μM + ANXA1 20 μM, 2400 sec, supernatant after acetone precipitation
ACE_0653-02_MK109	Human (overexpressed in *E. coli*)	Recomb. protein	HTRA1 20 μM + ANXA1 20 μM, 3600 sec, supernatant after acetone precipitation
ACE_0653-02_MK110	Human (overexpressed in *E. coli*)	Recomb. protein	HTRA1 20 μM + ANXA1 20 μM, 3600 sec, supernatant after acetone precipitation
ACE_0653-02_MK111	Human (overexpressed in *E. coli*)	Recomb. protein	HTRA1 20 μM + ANXA1 20 μM, 3600 sec, supernatant after acetone precipitation
ACE_0653-02_MK112	Human (overexpressed in *E. coli*)	Recomb. protein	HTRA1 20 μM + ANXA1 20 μM, 3600 sec, supernatant after acetone precipitation
ACE_0653-03_MK113	Human (overexpressed in *E. coli*)	Recomb. protein	HTRA1 0 μM + “denat.” ANXA1 20 μM, 0 sec, supernatant after acetone precipitation
ACE_0653-03_MK114	Human (overexpressed in *E. coli*)	Recomb. protein	HTRA1 0 μM + “denat.” ANXA1 20 μM, 0 sec, supernatant after acetone precipitation
ACE_0653-03_MK115	Human (overexpressed in *E. coli*)	Recomb. protein	HTRA1 0 μM + “denat.” ANXA1 20 μM, 0 sec, supernatant after acetone precipitation
ACE_0653-03_MK116	Human (overexpressed in *E. coli*)	Recomb. protein	HTRA1 0 μM + “denat.” ANXA1 20 μM, 0 sec, supernatant after acetone precipitation
ACE_0653-03_MK117	Human (overexpressed in *E. coli*)	Recomb. protein	HTRA1 0 μM + “denat.” ANXA1 20 μM, 600 sec, supernatant after acetone precipitation
ACE_0653-03_MK118	Human (overexpressed in *E. coli*)	Recomb. protein	HTRA1 0 μM + “denat.” ANXA1 20 μM, 600 sec, supernatant after acetone precipitation
ACE_0653-03_MK119	Human (overexpressed in *E. coli*)	Recomb. protein	HTRA1 0 μM + “denat.” ANXA1 20 μM, 600 sec, supernatant after acetone precipitation
ACE_0653-03_MK120	Human (overexpressed in *E. coli*)	Recomb. protein	HTRA1 0 μM + “denat.” ANXA1 20 μM, 600 sec, supernatant after acetone precipitation
ACE_0653-03_MK121	Human (overexpressed in *E. coli*)	Recomb. protein	HTRA1 20 μM + “denat.” ANXA1 0 μM, 0 sec, supernatant after acetone precipitation
ACE_0653-03_MK122	Human (overexpressed in *E. coli*)	Recomb. protein	HTRA1 20 μM + “denat.” ANXA1 0 μM, 0 sec, supernatant after acetone precipitation
ACE_0653-03_MK123	Human (overexpressed in *E. coli*)	Recomb. protein	HTRA1 20 μM + “denat.” ANXA1 0 μM, 0 sec, supernatant after acetone precipitation
ACE_0653-03_MK124	Human (overexpressed in *E. coli*)	Recomb. protein	HTRA1 20 μM + “denat.” ANXA1 0 μM, 0 sec, supernatant after acetone precipitation
ACE_0653-03_MK125	Human (overexpressed in *E. coli*)	Recomb. protein	HTRA1 20 μM + “denat.” ANXA1 0 μM, 3600 sec, supernatant after acetone precipitation

ACE_0653-03_MK126	Human (overexpressed in *E. coli*)	Recomb. protein	HTRA1 20 μM + "denat." ANXA1 0 μM, 3600 sec, supernatant after acetone precipitation
ACE_0653-03_MK127	Human (overexpressed in *E. coli*)	Recomb. protein	HTRA1 20 μM + "denat." ANXA1 0 μM, 3600 sec, supernatant after acetone precipitation
ACE_0653-03_MK128	Human (overexpressed in *E. coli*)	Recomb. protein	HTRA1 20 μM + "denat." ANXA1 0 μM, 3600 sec, supernatant after acetone precipitation
ACE_0653-03_MK129	Human (overexpressed in *E. coli*)	Recomb. protein	HTRA1 20 μM + "denat." ANXA1 20 μM, 0 sec, supernatant after acetone precipitation
ACE_0653-03_MK130	Human (overexpressed in *E. coli*)	Recomb. protein	HTRA1 20 μM + "denat." ANXA1 20 μM, 0 sec, supernatant after acetone precipitation
ACE_0653-03_MK131	Human (overexpressed in *E. coli*)	Recomb. protein	HTRA1 20 μM + "denat." ANXA1 20 μM, 0 sec, supernatant after acetone precipitation
ACE_0653-03_MK132	Human (overexpressed in *E. coli*)	Recomb. protein	HTRA1 20 μM + "denat." ANXA1 20 μM, 0 sec, supernatant after acetone precipitation
ACE_0653-03_MK133	Human (overexpressed in *E. coli*)	Recomb. protein	HTRA1 20 μM + "denat." ANXA1 20 μM, 15 sec, supernatant after acetone precipitation
ACE_0653-03_MK134	Human (overexpressed in *E. coli*)	Recomb. protein	HTRA1 20 μM + "denat." ANXA1 20 μM, 15 sec, supernatant after acetone precipitation
ACE_0653-03_MK135	Human (overexpressed in *E. coli*)	Recomb. protein	HTRA1 20 μM + "denat." ANXA1 20 μM, 15 sec, supernatant after acetone precipitation
ACE_0653-03_MK136	Human (overexpressed in *E. coli*)	Recomb. protein	HTRA1 20 μM + "denat." ANXA1 20 μM, 15 sec, supernatant after acetone precipitation
ACE_0653-03_MK137	Human (overexpressed in *E. coli*)	Recomb. protein	HTRA1 20 μM + "denat." ANXA1 20 μM, 30 sec, supernatant after acetone precipitation
ACE_0653-03_MK138	Human (overexpressed in *E. coli*)	Recomb. protein	HTRA1 20 μM + "denat." ANXA1 20 μM, 30 sec, supernatant after acetone precipitation
ACE_0653-03_MK139	Human (overexpressed in *E. coli*)	Recomb. protein	HTRA1 20 μM + "denat." ANXA1 20 μM, 30 sec, supernatant after acetone precipitation
ACE_0653-03_MK140	Human (overexpressed in *E. coli*)	Recomb. protein	HTRA1 20 μM + "denat." ANXA1 20 μM, 30 sec, supernatant after acetone precipitation
ACE_0653-03_MK141	Human (overexpressed in *E. coli*)	Recomb. protein	HTRA1 20 μM + "denat." ANXA1 20 μM, 60 sec, supernatant after acetone precipitation
ACE_0653-03_MK142	Human (overexpressed in *E. coli*)	Recomb. protein	HTRA1 20 μM + "denat." ANXA1 20 μM, 60 sec, supernatant after acetone precipitation
ACE_0653-03_MK143	Human (overexpressed in *E. coli*)	Recomb. protein	HTRA1 20 μM + "denat." ANXA1 20 μM, 60 sec, supernatant after acetone precipitation
ACE_0653-03_MK144	Human (overexpressed in *E. coli*)	Recomb. protein	HTRA1 20 μM + "denat." ANXA1 20 μM, 60 sec, supernatant after acetone precipitation
ACE_0653-03_MK145	Human (overexpressed in *E. coli*)	Recomb. protein	HTRA1 20 μM + "denat." ANXA1 20 μM, 90 sec, supernatant after acetone precipitation
ACE_0653-03_MK146	Human (overexpressed in *E. coli*)	Recomb. protein	HTRA1 20 μM + "denat." ANXA1 20 μM, 90 sec, supernatant after acetone precipitation
ACE_0653-03_MK147	Human (overexpressed in *E. coli*)	Recomb. protein	HTRA1 20 μM + "denat." ANXA1 20 μM, 90 sec, supernatant after acetone precipitation
ACE_0653-03_MK148	Human (overexpressed in *E. coli*)	Recomb. protein	HTRA1 20 μM + "denat." ANXA1 20 μM, 90 sec, supernatant after acetone precipitation
ACE_0653-03_MK149	Human (overexpressed in *E. coli*)	Recomb. protein	HTRA1 20 μM + "denat." ANXA1 20 μM, 120 sec, supernatant after acetone precipitation
ACE_0653-03_MK150	Human (overexpressed in *E. coli*)	Recomb. protein	HTRA1 20 μM + "denat." ANXA1 20 μM, 120 sec, supernatant after acetone precipitation
ACE_0653-03_MK151	Human (overexpressed in *E. coli*)	Recomb. protein	HTRA1 20 μM + "denat." ANXA1 20 μM, 120 sec, supernatant after acetone precipitation
ACE_0653-03_MK152	Human (overexpressed in *E. coli*)	Recomb. protein	HTRA1 20 μM + "denat." ANXA1 20 μM, 120 sec, supernatant after acetone precipitation
ACE_0653-03_MK153	Human (overexpressed in *E. coli*)	Recomb. protein	HTRA1 20 μM + "denat." ANXA1 20 μM, 300 sec, supernatant after acetone precipitation
ACE_0653-03_MK154	Human (overexpressed in *E. coli*)	Recomb. protein	HTRA1 20 μM + "denat." ANXA1 20 μM, 300 sec, supernatant after acetone precipitation
ACE_0653-03_MK155	Human (overexpressed in *E. coli*)	Recomb. protein	HTRA1 20 μM + "denat." ANXA1 20 μM, 300 sec, supernatant after acetone precipitation
ACE_0653-03_MK156	Human (overexpressed in *E. coli*)	Recomb. protein	HTRA1 20 μM + "denat." ANXA1 20 μM, 300 sec, supernatant after acetone precipitation
ACE_0653-03_MK157	Human (overexpressed in *E. coli*)	Recomb. protein	HTRA1 20 μM + "denat." ANXA1 20 μM, 600 sec, supernatant after acetone precipitation
ACE_0653-03_MK158	Human (overexpressed in *E. coli*)	Recomb. protein	HTRA1 20 μM + "denat." ANXA1 20 μM, 600 sec, supernatant after acetone precipitation
ACE_0653-03_MK159	Human (overexpressed in *E. coli*)	Recomb. protein	HTRA1 20 μM + "denat." ANXA1 20 μM, 600 sec, supernatant after acetone precipitation
ACE_0653-03_MK160	Human (overexpressed in *E. coli*)	Recomb. protein	HTRA1 20 μM + "denat." ANXA1 20 μM, 600 sec, supernatant after acetone precipitation

LC Settings

MS device	Orbitrap Elite
LC device	Evosep One
Ion source	Thermo Nanospray Flex
**Analytical column**	EV1064 Analytical Column – 60 & 100 samples/day
Column diameter	Length (L_C) = 8 cm; ID = 100; OD = PEEK; emitter EV-1086 Stainless steel emitter
Stationary phase	Dr Maisch C18 AQ, 3 μm beads
Particle diameter (d_p)	3 μm
Pore size	120 Å
Column ID	AC_EVO-003
**Solvents**	A: 0.1% FA in UPLC water<br>B: 0.1% FA in UPLC ACN
Gradient	21 min gradient

MS Settings

**Project**	**MS**	**general**	**MS1**	**MS2**	**MS2**	**MS3**	**Comments; special settings**
ACE_0653	Elite	Tune v2.7.0.1112 SP2<br>Gradient: 21 min	Analyzer: FT<br>Res.: 60000<br>SR: 300 - 1500<br>AGC: 3× 10^6<br>AcT: 50<br>RF: 30<br>SF: --<br>DDM: NS/15	Analyzer: IT<br>Res./ScR: -/rapid<br>SR: Auto<br>AGC: 1× 10^4<br>AcT: 60<br>CS: +2 and higher<br>IsM: IT<br>IsW: 2.0<br>Frag.: CID<br>NCE: 35			Classic orbitrap experiment: MS1 in Orbitrap at high resolution and data dependent MS2 in Iontrap at rapid scan rate. Dynamic exclusion enabled (exclude after n times=1; Exclusion duration (s)= 30; mass tolerance= ± 100 ppm), exclusion list size: 500

FT= Fourier Transform (Orbitrap); **IT**= Iontrap; **Q**= Quadrupol; **Res.**= max. Resolution at 200 m/z (Lumos) or 400 m/z (Elite) [FWHM (full width at half maximum)]; **ScR**= scan rate for measurements in the IT; **SR**= scan range [m/z]; **AGC**= automatic gain control, max number of acquired ions per measurement; **AcT**= max. Ion acquisition time [ms]; **CS**= charge states used for fragmentation; **IsM**= Isolation mode (Q or IT), MS2 isolation and further is only done in IT; **IsW**= Isolation window [m/z], value followed by scan mode the isolation is based on (MS1, MS2 …) **Frag.**= Fragmentation method; **HCD**= Higher-energy collisional dissociation; **CID**= Collision-induced dissociation; **ETD**= Electron-transfer dissociation**; EThcD=** Electron-Transfer/Higher-Energy Collision Dissociation; **sHCD**= stepped HCD**; NCE**= normalized collision energy; **cycles**: number of MSn recorded or max cycle time; **RF**= RF Lens [%]; **SF**= Source Fragmentation [V]; **DDM**: Data dependent Mode (cycle time in seconds, CT/[s] or number of scans, NS); **NS**= Number of data dependent scans

MaxQuant Search

Program & version	MaxQuant v2.0.2.0
Search engine	Andromeda
Settings	Basically default; LFQ and MBR were turned on. Normalization was turned off
Static modification	none
Digestion mode	unspecific
Dynamic modification	Acetyl (N-term); Oxidation (M)
Modification included in quantification	Oxidation (M)
Databases	1. Contaminants<br>2. ACE_0653_UP000000625_83333.fasta

The unfiltered results of the MaxQuant Search can be found here:

ACE_0653_all_in_modificationSpecificPep...	1/13/2022 10:03 AM	Text Document	6,024 KB
ACE_0653_all_in_parameters.txt	1/13/2022 10:03 AM	Text Document	4 KB
ACE_0653_all_in_peptides.txt	1/13/2022 10:03 AM	Text Document	7,183 KB
ACE_0653_all_in_proteinGroups.txt	1/13/2022 10:03 AM	Text Document	2,253 KB
ACE_0653_all_in_summary.txt	1/13/2022 10:04 AM	Text Document	42 KB
ACE_0653_all_in_tables.pdf	1/13/2022 10:03 AM	Adobe Acrobat D...	179 KB

ACE_0793-01/02/03

File legend

ACE ID	Organism	Organ/ cell line	Treatment/ experimental setup
ACE_0793-01_MK01	*S. scofa*	Pancreas/heart	Trypsin 0 µM + MDH 20 µM, 0 sec, supernatant after acetone precipitation
ACE_0793-01_MK02	*S. scofa*	Pancreas/heart	Trypsin 0 µM + MDH 20 µM, 0 sec, supernatant after acetone precipitation
ACE_0793-01_MK03	*S. scofa*	Pancreas/heart	Trypsin 0 µM + MDH 20 µM, 0 sec, supernatant after acetone precipitation
ACE_0793-01_MK04	*S. scofa*	Pancreas/heart	Trypsin 0 µM + MDH 20 µM, 0 sec, supernatant after acetone precipitation
ACE_0793-01_MK05	*S. scofa*	Pancreas/heart	Trypsin 0 µM + MDH 20 µM, 7200 sec, supernatant after acetone precipitation
ACE_0793-01_MK06	*S. scofa*	Pancreas/heart	Trypsin 0 µM + MDH 20 µM, 7200 sec, supernatant after acetone precipitation
ACE_0793-01_MK07	*S. scofa*	Pancreas/heart	Trypsin 0 µM + MDH 20 µM, 7200 sec, supernatant after acetone precipitation
ACE_0793-01_MK08	*S. scofa*	Pancreas/heart	Trypsin 0 µM + MDH 20 µM, 7200 sec, supernatant after acetone precipitation
ACE_0793-01_MK09	*S. scofa*	Pancreas/heart	Trypsin 20 µM + MDH 0 µM, 0 sec, supernatant after acetone precipitation
ACE_0793-01_MK10	*S. scofa*	Pancreas/heart	Trypsin 0.004 µM + MDH 0 µM, 0 sec, supernatant after acetone precipitation
ACE_0793-01_MK11	*S. scofa*	Pancreas/heart	Trypsin 0.004 µM + MDH 0 µM, 0 sec, supernatant after acetone precipitation
ACE_0793-01_MK12	*S. scofa*	Pancreas/heart	Trypsin 0.004 µM + MDH 0 µM, 0 sec, supernatant after acetone precipitation
ACE_0793-01_MK13	*S. scofa*	Pancreas/heart	Trypsin 0.004 µM + MDH 0 µM, 7200 sec, supernatant after acetone precipitation
ACE_0793-01_MK14	*S. scofa*	Pancreas/heart	Trypsin 0.004 µM + MDH 0 µM, 7200 sec, supernatant after acetone precipitation
ACE_0793-01_MK15	*S. scofa*	Pancreas/heart	Trypsin 0.004 µM + MDH 0 µM, 7200 sec, supernatant after acetone precipitation
ACE_0793-01_MK16	*S. scofa*	Pancreas/heart	Trypsin 0.004 µM + MDH 0 µM, 7200 sec, supernatant after acetone precipitation
ACE_0793-01_MK17	*S. scofa*	Pancreas/heart	Trypsin 0.004 µM + MDH 20 µM, 0 sec, supernatant after acetone precipitation
ACE_0793-01_MK18	*S. scofa*	Pancreas/heart	Trypsin 0.004 µM + MDH 20 µM, 0 sec, supernatant after acetone precipitation
ACE_0793-01_MK19	*S. scofa*	Pancreas/heart	Trypsin 0.004 µM + MDH 20 µM, 0 sec, supernatant after acetone precipitation
ACE_0793-01_MK20	*S. scofa*	Pancreas/heart	Trypsin 0.004 µM + MDH 20 µM, 0 sec, supernatant after acetone precipitation
ACE_0793-01_MK21	*S. scofa*	Pancreas/heart	Trypsin 0.004 µM + MDH 20 µM, 15 sec, supernatant after acetone precipitation
ACE_0793-01_MK22	*S. scofa*	Pancreas/heart	Trypsin 0.004 µM + MDH 20 µM, 15 sec, supernatant after acetone precipitation
ACE_0793-01_MK23	*S. scofa*	Pancreas/heart	Trypsin 0.004 µM + MDH 20 µM, 15 sec, supernatant after acetone precipitation
ACE_0793-01_MK24	*S. scofa*	Pancreas/heart	Trypsin 0.004 µM + MDH 20 µM, 15 sec, supernatant after acetone precipitation
ACE_0793-01_MK25	*S. scofa*	Pancreas/heart	Trypsin 0.004 µM + MDH 20 µM, 30 sec, supernatant after acetone precipitation
ACE_0793-01_MK26	*S. scofa*	Pancreas/heart	Trypsin 0.004 µM + MDH 20 µM, 30 sec, supernatant after acetone precipitation
ACE_0793-01_MK27	*S. scofa*	Pancreas/heart	Trypsin 0.004 µM + MDH 20 µM, 30 sec, supernatant after acetone precipitation
ACE_0793-01_MK28	*S. scofa*	Pancreas/heart	Trypsin 0.004 µM + MDH 20 µM, 30 sec, supernatant after acetone precipitation
ACE_0793-01_MK29	*S. scofa*	Pancreas/heart	Trypsin 0.004 µM + MDH 20 µM, 45 sec, supernatant after acetone precipitation
ACE_0793-01_MK30	*S. scofa*	Pancreas/heart	Trypsin 0.004 µM + MDH 20 µM, 45 sec, supernatant after acetone precipitation
ACE_0793-01_MK31	*S. scofa*	Pancreas/heart	Trypsin 0.004 µM + MDH 20 µM, 45 sec, supernatant after acetone precipitation
ACE_0793-01_MK32	*S. scofa*	Pancreas/heart	Trypsin 0.004 µM + MDH 20 µM, 45 sec, supernatant after acetone precipitation
ACE_0793-01_MK33	*S. scofa*	Pancreas/heart	Trypsin 0.004 µM + MDH 20 µM, 60 sec, supernatant after acetone precipitation
ACE_0793-01_MK34	*S. scofa*	Pancreas/heart	Trypsin 0.004 µM + MDH 20 µM, 60 sec, supernatant after acetone precipitation
ACE_0793-01_MK35	*S. scofa*	Pancreas/heart	Trypsin 0.004 µM + MDH 20 µM, 60 sec, supernatant after acetone precipitation
ACE_0793-01_MK36	*S. scofa*	Pancreas/heart	Trypsin 0.004 µM + MDH 20 µM, 60 sec, supernatant after acetone precipitation
ACE_0793-01_MK37	*S. scofa*	Pancreas/heart	Trypsin 0.004 µM + MDH 20 µM, 90 sec, supernatant after acetone precipitation
ACE_0793-01_MK38	*S. scofa*	Pancreas/heart	Trypsin 0.004 µM + MDH 20 µM, 90 sec, supernatant after acetone precipitation
ACE_0793-01_MK39	*S. scofa*	Pancreas/heart	Trypsin 0.004 µM + MDH 20 µM, 90 sec, supernatant after acetone precipitation

ACE_0793-01_MK40	*S. scofa*	Pancreas/heart	Trypsin 0.004 µM + MDH 20 µM, 90 sec, supernatant after acetone precipitation
ACE_0793-01_MK41	*S. scofa*	Pancreas/heart	Trypsin 0.004 µM + MDH 20 µM, 120 sec, supernatant after acetone precipitation
ACE_0793-01_MK42	*S. scofa*	Pancreas/heart	Trypsin 0.004 µM + MDH 20 µM, 120 sec, supernatant after acetone precipitation
ACE_0793-01_MK43	*S. scofa*	Pancreas/heart	Trypsin 0.004 µM + MDH 20 µM, 120 sec, supernatant after acetone precipitation
ACE_0793-01_MK44	*S. scofa*	Pancreas/heart	Trypsin 0.004 µM + MDH 20 µM, 120 sec, supernatant after acetone precipitation
ACE_0793-01_MK45	*S. scofa*	Pancreas/heart	Trypsin 0.004 µM + MDH 20 µM, 300 sec, supernatant after acetone precipitation
ACE_0793-01_MK46	*S. scofa*	Pancreas/heart	Trypsin 0.004 µM + MDH 20 µM, 300 sec, supernatant after acetone precipitation
ACE_0793-01_MK47	*S. scofa*	Pancreas/heart	Trypsin 0.004 µM + MDH 20 µM, 300 sec, supernatant after acetone precipitation
ACE_0793-01_MK48	*S. scofa*	Pancreas/heart	Trypsin 0.004 µM + MDH 20 µM, 300 sec, supernatant after acetone precipitation
ACE_0793-01_MK49	*S. scofa*	Pancreas/heart	Trypsin 0.004 µM + MDH 20 µM, 600 sec, supernatant after acetone precipitation
ACE_0793-01_MK50	*S. scofa*	Pancreas/heart	Trypsin 0.004 µM + MDH 20 µM, 600 sec, supernatant after acetone precipitation
ACE_0793-01_MK51	*S. scofa*	Pancreas/heart	Trypsin 0.004 µM + MDH 20 µM, 600 sec, supernatant after acetone precipitation
ACE_0793-01_MK52	*S. scofa*	Pancreas/heart	Trypsin 0.004 µM + MDH 20 µM, 600 sec, supernatant after acetone precipitation
ACE_0793-01_MK53	*S. scofa*	Pancreas/heart	Trypsin 0.004 µM + MDH 20 µM, 1200 sec, supernatant after acetone precipitation
ACE_0793-01_MK54	*S. scofa*	Pancreas/heart	Trypsin 0.004 µM + MDH 20 µM, 1200 sec, supernatant after acetone precipitation
ACE_0793-01_MK55	*S. scofa*	Pancreas/heart	Trypsin 0.004 µM + MDH 20 µM, 1200 sec, supernatant after acetone precipitation
ACE_0793-01_MK56	*S. scofa*	Pancreas/heart	Trypsin 0.004 µM + MDH 20 µM, 1200 sec, supernatant after acetone precipitation
ACE_0793-01_MK57	*S. scofa*	Pancreas/heart	Trypsin 0.004 µM + MDH 20 µM, 1800 sec, supernatant after acetone precipitation
ACE_0793-01_MK58	*S. scofa*	Pancreas/heart	Trypsin 0.004 µM + MDH 20 µM, 1800 sec, supernatant after acetone precipitation
ACE_0793-01_MK59	*S. scofa*	Pancreas/heart	Trypsin 0.004 µM + MDH 20 µM, 1800 sec, supernatant after acetone precipitation
ACE_0793-01_MK60	*S. scofa*	Pancreas/heart	Trypsin 0.004 µM + MDH 20 µM, 1800 sec, supernatant after acetone precipitation
ACE_0793-01_MK61	*S. scofa*	Pancreas/heart	Trypsin 0.004 µM + MDH 20 µM, 7200 sec, supernatant after acetone precipitation
ACE_0793-01_MK62	*S. scofa*	Pancreas/heart	Trypsin 0.004 µM + MDH 20 µM, 7200 sec, supernatant after acetone precipitation
ACE_0793-01_MK63	*S. scofa*	Pancreas/heart	Trypsin 0.004 µM + MDH 20 µM, 7200 sec, supernatant after acetone precipitation
ACE_0793-01_MK64	*S. scofa*	Pancreas/heart	Trypsin 0.004 µM + MDH 20 µM, 7200 sec, supernatant after acetone precipitation
ACE_0793-02_MK01	S. scrofa	Pancreas/heart	Trypsin 0 µM + “denat.” MDH 20 µM, 0 sec, supernatant after acetone precipitation
ACE_0793-02_MK02	S. scrofa	Pancreas/heart	Trypsin 0 µM + “denat.” MDH 20 µM, 0 sec, supernatant after acetone precipitation
ACE_0793-02_MK03	S. scrofa	Pancreas/heart	Trypsin 0 µM + “denat.” MDH 20 µM, 0 sec, supernatant after acetone precipitation
ACE_0793-02_MK04	S. scrofa	Pancreas/heart	Trypsin 0 µM + “denat.” MDH 20 µM, 0 sec, supernatant after acetone precipitation
ACE_0793-02_MK05	S. scrofa	Pancreas/heart	Trypsin 0 µM + “denat.” MDH 20 µM, 600 sec, supernatant after acetone precipitation
ACE_0793-02_MK06	S. scrofa	Pancreas/heart	Trypsin 0 µM + “denat.” MDH 20 µM, 600 sec, supernatant after acetone precipitation
ACE_0793-02_MK07	S. scrofa	Pancreas/heart	Trypsin 0 µM + “denat.” MDH 20 µM, 600 sec, supernatant after acetone precipitation
ACE_0793-02_MK08	S. scrofa	Pancreas/heart	Trypsin 0 µM + “denat.” MDH 20 µM, 600 sec, supernatant after acetone precipitation
ACE_0793-02_MK09	S. scrofa	Pancreas/heart	Trypsin 0.004 µM + “denat.” MDH 0 µM, 0 sec, supernatant after acetone precipitation
ACE_0793-02_MK10	S. scrofa	Pancreas/heart	Trypsin 0.004 µM + “denat.” MDH 0 µM, 0 sec, supernatant after acetone precipitation
ACE_0793-02_MK11	S. scrofa	Pancreas/heart	Trypsin 0.004 µM + “denat.” MDH 0 µM, 0 sec, supernatant after acetone precipitation
ACE_0793-02_MK12	S. scrofa	Pancreas/heart	Trypsin 0.004 µM + “denat.” MDH 0 µM, 0 sec, supernatant after acetone precipitation
ACE_0793-02_MK13	S. scrofa	Pancreas/heart	Trypsin 0.004 µM + “denat.” MDH 0 µM, 600 sec, supernatant after acetone precipitation
ACE_0793-02_MK14	S. scrofa	Pancreas/heart	Trypsin 0.004 µM + “denat.” MDH 0 µM, 600 sec, supernatant after acetone precipitation
ACE_0793-02_MK15	S. scrofa	Pancreas/heart	Trypsin 0.004 µM + “denat.” MDH 0 µM, 600 sec, supernatant after acetone precipitation
ACE_0793-02_MK16	S. scrofa	Pancreas/heart	Trypsin 0.004 µM + “denat.” MDH 0 µM, 600 sec, supernatant after acetone precipitation
ACE_0793-02_MK17	S. scrofa	Pancreas/heart	Trypsin 0.004 µM + “denat.” MDH 20 µM, 0 sec, supernatant after acetone precipitation
ACE_0793-02_MK18	S. scrofa	Pancreas/heart	Trypsin 0.004 µM + “denat.” MDH 20 µM, 0 sec, supernatant after acetone precipitation

ACE_0793-02_MK19	S. scrofa	Pancreas/heart	Trypsin 0.004 µM + “denat.” MDH 20 µM, 0 sec, supernatant after acetone precipitation
ACE_0793-02_MK20	S. scrofa	Pancreas/heart	Trypsin 0.004 µM + “denat.” MDH 20 µM, 0 sec, supernatant after acetone precipitation
ACE_0793-02_MK21	S. scrofa	Pancreas/heart	Trypsin 0.004 µM + “denat.” MDH 20 µM, 15 sec, supernatant after acetone precipitation
ACE_0793-02_MK22	S. scrofa	Pancreas/heart	Trypsin 0.004 µM + “denat.” MDH 20 µM, 15 sec, supernatant after acetone precipitation
ACE_0793-02_MK23	S. scrofa	Pancreas/heart	Trypsin 0.004 µM + “denat.” MDH 20 µM, 15 sec, supernatant after acetone precipitation
ACE_0793-02_MK24	S. scrofa	Pancreas/heart	Trypsin 0.004 µM + “denat.” MDH 20 µM, 15 sec, supernatant after acetone precipitation
ACE_0793-02_MK25	S. scrofa	Pancreas/heart	Trypsin 0.004 µM + “denat.” MDH 20 µM, 30 sec, supernatant after acetone precipitation
ACE_0793-02_MK26	S. scrofa	Pancreas/heart	Trypsin 0.004 µM + “denat.” MDH 20 µM, 30 sec, supernatant after acetone precipitation
ACE_0793-02_MK27	S. scrofa	Pancreas/heart	Trypsin 0.004 µM + “denat.” MDH 20 µM, 30 sec, supernatant after acetone precipitation
ACE_0793-02_MK28	S. scrofa	Pancreas/heart	Trypsin 0.004 µM + “denat.” MDH 20 µM, 30 sec, supernatant after acetone precipitation
ACE_0793-02_MK29	S. scrofa	Pancreas/heart	Trypsin 0.004 µM + “denat.” MDH 20 µM, 45 sec, supernatant after acetone precipitation
ACE_0793-02_MK30	S. scrofa	Pancreas/heart	Trypsin 0.004 µM + “denat.” MDH 20 µM, 45 sec, supernatant after acetone precipitation
ACE_0793-02_MK31	S. scrofa	Pancreas/heart	Trypsin 0.004 µM + “denat.” MDH 20 µM, 45 sec, supernatant after acetone precipitation
ACE_0793-02_MK32	S. scrofa	Pancreas/heart	Trypsin 0.004 µM + “denat.” MDH 20 µM, 45 sec, supernatant after acetone precipitation
ACE_0793-02_MK33	S. scrofa	Pancreas/heart	Trypsin 0.004 µM + “denat.” MDH 20 µM, 60 sec, supernatant after acetone precipitation
ACE_0793-02_MK34	S. scrofa	Pancreas/heart	Trypsin 0.004 µM + “denat.” MDH 20 µM, 60 sec, supernatant after acetone precipitation
ACE_0793-02_MK35	S. scrofa	Pancreas/heart	Trypsin 0.004 µM + “denat.” MDH 20 µM, 60 sec, supernatant after acetone precipitation
ACE_0793-02_MK36	S. scrofa	Pancreas/heart	Trypsin 0.004 µM + “denat.” MDH 20 µM, 60 sec, supernatant after acetone precipitation
ACE_0793-02_MK37	S. scrofa	Pancreas/heart	Trypsin 0.004 µM + “denat.” MDH 20 µM, 120 sec, supernatant after acetone precipitation
ACE_0793-02_MK38	S. scrofa	Pancreas/heart	Trypsin 0.004 µM + “denat.” MDH 20 µM, 120 sec, supernatant after acetone precipitation
ACE_0793-02_MK39	S. scrofa	Pancreas/heart	Trypsin 0.004 µM + “denat.” MDH 20 µM, 120 sec, supernatant after acetone precipitation
ACE_0793-02_MK40	S. scrofa	Pancreas/heart	Trypsin 0.004 µM + “denat.” MDH 20 µM, 120 sec, supernatant after acetone precipitation
ACE_0793-02_MK41	S. scrofa	Pancreas/heart	Trypsin 0.004 µM + “denat.” MDH 20 µM, 300 sec, supernatant after acetone precipitation
ACE_0793-02_MK42	S. scrofa	Pancreas/heart	Trypsin 0.004 µM + “denat.” MDH 20 µM, 300 sec, supernatant after acetone precipitation
ACE_0793-02_MK43	S. scrofa	Pancreas/heart	Trypsin 0.004 µM + “denat.” MDH 20 µM, 300 sec, supernatant after acetone precipitation
ACE_0793-02_MK44	S. scrofa	Pancreas/heart	Trypsin 0.004 µM + “denat.” MDH 20 µM, 300 sec, supernatant after acetone precipitation
ACE_0793-02_MK45	S. scrofa	Pancreas/heart	Trypsin 0.004 µM + “denat.” MDH 20 µM, 600 sec, supernatant after acetone precipitation
ACE_0793-02_MK46	S. scrofa	Pancreas/heart	Trypsin 0.004 µM + “denat.” MDH 20 µM, 600 sec, supernatant after acetone precipitation
ACE_0793-02_MK47	S. scrofa	Pancreas/heart	Trypsin 0.004 µM + “denat.” MDH 20 µM, 600 sec, supernatant after acetone precipitation
ACE_0793-02_MK48	S. scrofa	Pancreas/heart	Trypsin 0.004 µM + “denat.” MDH 20 µM, 600 sec, supernatant after acetone precipitation
ACE_0793-03_MK01	*S. scorfa*	Pancreas/heart	Trypsin 0 µM + MDH 20 µM, 0 sec, supernatant after acetone precipitation
ACE_0793-03_MK02	*S. scorfa*	Pancreas/heart	Trypsin 0 µM + MDH 20 µM, 0 sec, supernatant after acetone precipitation
ACE_0793-03_MK03	*S. scorfa*	Pancreas/heart	Trypsin 0 µM + MDH 20 µM, 0 sec, supernatant after acetone precipitation
ACE_0793-03_MK04	*S. scorfa*	Pancreas/heart	Trypsin 0 µM + MDH 20 µM, 0 sec, supernatant after acetone precipitation
ACE_0793-03_MK05	*S. scorfa*	Pancreas/heart	Trypsin 0 µM + MDH 20 µM, 120 sec, supernatant after acetone precipitation
ACE_0793-03_MK06	*S. scorfa*	Pancreas/heart	Trypsin 0 µM + MDH 20 µM, 120 sec, supernatant after acetone precipitation
ACE_0793-03_MK07	*S. scorfa*	Pancreas/heart	Trypsin 0 µM + MDH 20 µM, 120 sec, supernatant after acetone precipitation
ACE_0793-03_MK08	*S. scorfa*	Pancreas/heart	Trypsin 0 µM + MDH 20 µM, 120 sec, supernatant after acetone precipitation
ACE_0793-03_MK29	*S. scorfa*	Pancreas/heart	Trypsin 0.002 µM + MDH 20 µM, 0 sec, supernatant after acetone precipitation
ACE_0793-03_MK30	*S. scorfa*	Pancreas/heart	Trypsin 0.002 µM + MDH 20 µM, 0 sec, supernatant after acetone precipitation
ACE_0793-03_MK31	*S. scorfa*	Pancreas/heart	Trypsin 0.002 µM + MDH 20 µM, 0 sec, supernatant after acetone precipitation
ACE_0793-03_MK32	*S. scorfa*	Pancreas/heart	Trypsin 0.002 µM + MDH 20 µM, 0 sec, supernatant after acetone precipitation
ACE_0793-03_MK33	*S. scorfa*	Pancreas/heart	Trypsin 0.002 µM + MDH 20 µM, 15 sec, supernatant after acetone precipitation

ACE_0793-03_MK34	*S. scorfa*	Pancreas/heart	Trypsin 0.002 µM + MDH 20 µM, 15 sec, supernatant after acetone precipitation
ACE_0793-03_MK35	*S. scorfa*	Pancreas/heart	Trypsin 0.002 µM + MDH 20 µM, 15 sec, supernatant after acetone precipitation
ACE_0793-03_MK36	*S. scorfa*	Pancreas/heart	Trypsin 0.002 µM + MDH 20 µM, 15 sec, supernatant after acetone precipitation
ACE_0793-03_MK37	*S. scorfa*	Pancreas/heart	Trypsin 0.002 µM + MDH 20 µM, 30 sec, supernatant after acetone precipitation
ACE_0793-03_MK38	*S. scorfa*	Pancreas/heart	Trypsin 0.002 µM + MDH 20 µM, 30 sec, supernatant after acetone precipitation
ACE_0793-03_MK39	*S. scorfa*	Pancreas/heart	Trypsin 0.002 µM + MDH 20 µM, 30 sec, supernatant after acetone precipitation
ACE_0793-03_MK40	*S. scorfa*	Pancreas/heart	Trypsin 0.002 µM + MDH 20 µM, 30 sec, supernatant after acetone precipitation

LC

Settings

MS device	Orbitrap Elite
LC device	Evosep One
Ion source	Thermo Nanospray Flex
**Analytical column**	EV1064 Analytical Column – 60 & 100 samples/day
Column diameter	Length (L_C) = 8 cm; ID = 100; OD = PEEK; emitter EV-1086 Stainless steel emitter
Stationary phase	Dr Maisch C18 AQ, 3 μm beads
Particle diameter (d_p)	3 μm
Pore size	120 Å
Column ID	AC_EVO-008
**Solvents**	A: 0.1% FA in UPLC water<br>B: 0.1% FA in UPLC ACN
Gradient	21 min gradient

MS Settings

**Project**	**MS**	**general**	**MS1**	**MS2**	**MS2**	**MS3**	**Comments; special settings**
ACE_0793	Lumos	Tune v3.5.3881.18<br>Xcalibur v4.5.445.18<br>Gradient: 21 min	Analyzer: FT<br>Res.: 120000<br>SR: 375 - 1600<br>AGC: Standard<br>AcT: Auto<br>RF: 30<br>SF: --<br>DDM: CT/3sec	Analyzer: IT<br>Res./ScR: -/rapid<br>SR: Auto<br>AGC: Standard<br>AcT: Auto<br>CS: +2 to +6<br>IsM: Q<br>IsW: 1.6<br>Frag.: HCD<br>NCE: 32			classic orbitrap experiment: MS1 in Orbitrap at high resolution and data dependent MS2 in Iontrap at turbo scan rate. Dynamic exclusion enabled (exclude after n times=1; Exclusion duration (s)= 20; mass tolerance= ± 10ppm)<br>intensity threshold: 5×10^3

FT= Fourier Transform (Orbitrap); **IT**= Iontrap; **Q**= Quadrupol; **Res.**= max. Resolution at 200 m/z (Lumos) or 400 m/z (Elite) [FWHM (full width at half maximum)]; **ScR**= scan rate for measurements in the IT; **SR**= scan range [m/z]; **AGC**= automatic gain control, max number of acquired ions per measurement; **AcT**= max. Ion acquisition time [ms]; **CS**= charge states used for fragmentation; **IsM**= Isolation mode (Q or IT), MS2 isolation and further is only done in IT; **IsW**= Isolation window [m/z], value followed by scan mode the isolation is based on (MS1, MS2 …) **Frag.**= Fragmentation method; **HCD**= Higher-energy collisional dissociation; **CID**= Collision-induced dissociation; **ETD**= Electron-transfer dissociation**; EThcD=** Electron-Transfer/Higher-Energy Collision Dissociation; **sHCD**= stepped HCD**; NCE**= normalized collision energy; **cycles**: number of MSn recorded or max cycle time; **RF**= RF Lens [%]; **SF**= Source Fragmentation [V]; **DDM**: Data dependent Mode (cycle time in seconds, CT/[s] or number of scans, NS); **NS**= Number of data dependent scans

MaxQuant Search

Program & version	MaxQuant v2.0.3.0
Search engine	Andromeda
settings	Basically default; LFQ and MBR were turned on
Static modification	none
Digestion mode	unspecific
Dynamic modification	Acetyl (N-term); Oxidation (M)
Modification included in quantification	Oxidation (M)
Databases	1. ACE_793_SOI_plus_con.fasta

The unfiltered results of the MaxQuant Search can be found here:

ACE_0793-01_Sec._7.1._MQ01_unspecific_modificationSpecificPeptides.txt	13/07/2023 11:44	Textdokument	629 KB
ACE_0793-01_Sec._7.1._MQ01_unspecific_Oxidation (M)Sites.txt	13/07/2023 11:44	Textdokument	76 KB
ACE_0793-01_Sec._7.1._MQ01_unspecific_parameters.txt	13/07/2023 11:44	Textdokument	4 KB
ACE_0793-01_Sec._7.1._MQ01_unspecific_peptides.txt	13/07/2023 11:44	Textdokument	720 KB
ACE_0793-01_Sec._7.1._MQ01_unspecific_proteinGroups.txt	13/07/2023 11:44	Textdokument	298 KB
ACE_0793-01_Sec._7.1._MQ01_unspecific_summary.txt	13/07/2023 11:44	Textdokument	18 KB
ACE_0793-01_Sec._7.1._MQ01_unspecific_tables.pdf	13/07/2023 11:44	Firefox PDF Docu...	94 KB

ACE_0793-02_Sec._7.1._MQ01_unspecific_modificationSpecificPeptides.txt	13/07/2023 11:44	Textdokument	539 KB
ACE_0793-02_Sec._7.1._MQ01_unspecific_Oxidation (M)Sites.txt	13/07/2023 11:44	Textdokument	77 KB
ACE_0793-02_Sec._7.1._MQ01_unspecific_parameters.txt	13/07/2023 11:44	Textdokument	4 KB
ACE_0793-02_Sec._7.1._MQ01_unspecific_peptides.txt	13/07/2023 11:44	Textdokument	595 KB
ACE_0793-02_Sec._7.1._MQ01_unspecific_proteinGroups.txt	13/07/2023 11:44	Textdokument	251 KB
ACE_0793-02_Sec._7.1._MQ01_unspecific_summary.txt	13/07/2023 11:44	Textdokument	14 KB
ACE_0793-02_Sec._7.1._MQ01_unspecific_tables.pdf	13/07/2023 11:44	Firefox PDF Docu...	80 KB

ACE_0793-03_Sec._7.1._MQ01_modificationSpecificPeptides.txt	13/07/2023 11:44	Textdokument	231 KB
ACE_0793-03_Sec._7.1._MQ01_unspecific_Oxidation (M)Sites.txt	13/07/2023 11:44	Textdokument	69 KB
ACE_0793-03_Sec._7.1._MQ01_unspecific_parameters.txt	13/07/2023 11:44	Textdokument	4 KB
ACE_0793-03_Sec._7.1._MQ01_unspecific_peptides.txt	13/07/2023 11:44	Textdokument	291 KB
ACE_0793-03_Sec._7.1._MQ01_unspecific_proteinGroups.txt	13/07/2023 11:44	Textdokument	119 KB
ACE_0793-03_Sec._7.1._MQ01_unspecific_summary.txt	13/07/2023 11:44	Textdokument	17 KB
ACE_0793-03_Sec._7.1._MQ01_unspecific_tables.pdf	13/07/2023 11:44	Firefox PDF Docu...	91 KB
